# Host-initiated microbial association leads to stable ectosymbiosis in an ecological model

**DOI:** 10.1371/journal.pcbi.1014699

**Published:** 2026-09-02

**Authors:** Nandakishor Krishnan, István Zachar, Ádám Kun, Chaitanya S. Gokhale, József Garay

**Affiliations:** 1 Institute of Evolution, HUN-REN Centre for Ecological Research, Budapest, Hungary; 2 Doctoral School of Biology, Institute of Biology, Eötvös Loránd University, Budapest, Hungary; 3 Department of Plant Systematics, Ecology and Theoretical Biology, Institute of Biology, Eötvös Loránd University, Budapest, Hungary; 4 Parmenides Center for the Conceptual Foundations of Science, Parmenides Foundation, Pöcking, Germany; 5 Chair for Computational and Theoretical Biology, Julius-Maximilians University, Würzburg, Germany; 6 Department of Theoretical Biology, Max Planck Institute for Evolutionary Biology, Plön, Germany; University of Illinois at Urbana-Champaign, UNITED STATES OF AMERICA

## Abstract

Microbial symbiosis is widespread among metabolically coupled cells; it presumably gave rise to mitochondria. However, how such symbioses emerge, evolve, and stabilize are unknown, particularly in the prokaryotic domain where endosymbiosis is virtually nonexistent. Yet there is growing evidence suggesting that mitochondria originated from such a metabolically driven prokaryotic partnership rather than phagocytotic predation. While prokaryotes almost ubiquitously engage in metabolic syntrophy, it is unknown whether syntrophy alone can enable stable physical associations that could pave the road toward physical integration. Here, we tested the hypothesis that syntrophy can transition into stable ectosymbiosis, using an ecological mathematical model. Starting from an existing syntrophic partnership between free-living hosts and symbionts, we demonstrate that population-level obligate ectosymbiosis can emerge and stabilize, even in unilateral syntrophy where only the symbiont consumes a host-produced metabolite. A key assumption is that the hosts’ by-product inhibits their growth when it accumulates. By consuming the toxic by-product, the symbiont locally reduces hosts’ self-inhibition at the contact surface, manifesting as a private benefit providing selective advantage. Our results show that due to the direct and indirect benefits, the ectosymbiotic consortium is stable against free-living forms and the consortial cooperation is ecologically selected for. Furthermore, solid metabolic coupling promotes population-level obligacy, ultimately excluding free-living individuals under stricter conditions. Our results support the hypothesis that cooperative, syntrophic microbes (particularly prokaryotes) are capable of forming stable, physical, and species-specific ectosymbiosis through inhibition reduction, providing a plausible first step toward potential, gradual endosymbiotic integration. Our work bridges the gap between models of microbial cooperation between free-living species and models that assume already-concluded, fully integrated endosymbiosis under multilevel selection.

## Author summary

Metabolic interactions among microbes are integral to their associations ranging from cross-feeding between free-living individuals to physically integrated cells. While metabolic coupling among free-living microbes is ubiquitous, the conditions that enable and facilitate a more intimate physical connection or even integration remain unclear. This is mostly because we do not know examples that demonstrate how the process unfolds and we lack intermediates of the most important and perhaps the first of such integrations: mitochondria. With the potential benefits of forming a higher unit of selection by prolonged association, we hypothesize that an evolutionary path could exist from free-living syntrophic partners toward obligate endosymbiosis through the intermediate stage of syntrophic ectosymbiosis. Here we provide a theoretical model that demonstrates that host-initiated ectosymbiosis can be stable, even when the cross-feeding with the bound symbionts is unilateral. Our results demonstrate that the physical association with a syntrophic partner is selected for as a mechanism to survive a self-produced toxic metabolite. Self-inhibition turns out to be crucial as the feeding symbiont reducing it effectively provides the necessary benefit for the host. Our model provides a potential mechanism of an ecological driver that could hypothetically lead to the endosymbiotic origin of mitochondria among prokaryotic partners (alphaproteobacterium and Asgard archaeon), without assuming a direct metabolic benefit for the host.

## Introduction

Microbes, especially prokaryotes, almost ubiquitously engage in metabolic cooperation at every habitat they occupy. They establish cooperation through various metabolites they produce or enable in their neighborhood via their metabolisms. They can evolve dependencies on biofunctions of each other on a diverse scale, ranging from pairwise to multipartite associations [1] and from direct cross-feeding to protection mutualism [2–4]. Syntrophy is an umbrella term to denote metabolic interaction in which constituent partners rely on one another (bi- or unilateral) for growth-sustaining metabolites [5–9]. The microbial syntrophic relationship facilitates a synergy for associations and communities among them [6,10–12]. Permanent and obligate pairwise metabolic cooperation may even evolve into physically linked symbioses, where one partner is the long-term ectosymbiont of the other (*Chlorochromatium* [13–15] or epibiotic γ-proteobacteria on *Giganthauma karukerense*, a Thaumarchaeota [16]). When a syntrophic partnership ensures better-than-random probability of co-inheritance of partners, group selection may select for the pair instead of free-living individuals. As a result, a new evolutionary unit may evolve under a new level of selection, ultimately leading to a major evolutionary transition [17,18]. Importantly, this need not happen when one partner is physically integrated into the other (even in case of ectosymbiosis), but when they become obligately dependent on each other.

According to mainstream hypotheses [19–23], the origin of the eukaryotic cell, a major transition itself, was the result of such a metabolic cooperation between free-living cells that turned obligate and irreversible, one partner ultimately ending up within the other. While the ancestral partners are pointed out with increasing confidence from the bacterial and archaeal domains [19,21,23–26], there is no consensus on many critical points of the origin [27]. Their initial interaction, benefits, and mechanism of inclusion are still unknown, and the omics data trickling in can rarely differentiate among contending hypotheses, leaving room for much speculation, often without merit (most, if not all, hypotheses remain to be validated [28]). The issue is further complicated by the fact that eukaryogenesis happened more than a billion years ago with elusive traces and without second examples. There is neither another origin of the nucleus nor another merger between two prokaryotes being on par with the success and stability of the ancestral mitochondrion and its host. Despite a few known cases of ectosymbiotic prokaryotic partnerships [29,30], we do not know how these partnerships were initiated, stabilized, and coevolved. As a result, it remains unclear how obligate symbioses of syntrophic partners may ensue. Without directly observable cases of prokaryotic ecto- or endosymbioses emerging from metabolic cooperation (natural or artificial), theoretical modelling remains the only approach to identify the potential driving factors that can lead to stable symbiotic consortium in a syntrophically interacting community. Yet the field of eukaryogenesis severely lacks appropriate modelling to back up the various theories, and research has notoriously been hampered by the lack of models to test the hypotheses.

One particularly long-lasting debate concerns the original interaction of eukaryogenetic parties, specifically the nature and formation of the initial interaction of the host and the future mitochondrion. Arguably, how the symbiont got inside the host is extremely important. While phagotrophy or parasitic invasion cannot yet be excluded, here we focus on metabolic syntrophy only (that is available to prokaryotes too). More specifically, we focus on a step that likely preceded physical integration. We propose that a stable ectosymbiosis evolved out of a syntrophic system before the inclusion of the symbiont (also see [31]), contrary to the possibility of direct internalization as in, e.g., phagotrophic farming [32]. While the metabolic coupling of the ancestral parties was and still is the focus of considerable research [21,28,33–37], syntrophic theories agree on one aspect: the metabolite-mediated cooperation between parties provided the selective advantage for the pair over free-living individuals. It is well established through modelling that metabolic cooperation can lead to microbial mutualism [38–46], with models covering many specific metabolic interaction types (also see [2,47]). Yet, to our knowledge, there are no models that study how metabolic cooperation (syntrophy) may lead to stable, obligate physical integration, something that seems to be an absolutely crucial step on the road to endosymbiosis. The lack of such models or data urges us to investigate the hypothesis of syntrophy leading to endosymbiosis. Our model is motivated by the various syntrophic microbial cooperation cases known, with a focus on eukaryogenesis. We concentrate on a mechanism to bridge microbial models that assume free-living species in a community (even if part of biofilms [48]) with those that deal with upfront physical integration [32,49].

We introduce a theoretical model to test the specific hypothesis that metabolic syntrophy can lead to physical ectosymbiosis under multilevel selection. We assume a worst-case scenario of a physically independent (free-living) pair of *hosts* and *symbionts* belonging to different species in unilateral and obligate syntrophy. The symbiont feeds on the metabolic product of the host. Rising concentration of the hosts’ metabolite (accumulated in the habitat due to the whole host population) is toxic to the hosts, inhibiting their growth. There are many examples among microbes where a product inhibits growth and its consumption (effectively reducing the concentration in the vicinity) by other species provides cooperative help, e.g., *Pseudomonas* sp. produces methanol that inhibits its growth while *Hyphomicrobium* sp. can utilize methanol [6,50]. Apparently, cross-feeding and metabolic inhibition affecting microbial consortia are prevalent [51–53].

Our model considers the invasion of a (costly) mutant host that can bind its free-living symbiont partners on its cell surface, forming ectosymbiotic association. In the ectosymbiotic consortium, symbionts on the host surface effectively reduce the free surface area of the host’s plasma membrane. This has two direct effects on the host: reduced surface implies constrained resource consumption, and it reduces exposure to the inhibitory effect of its (and conspecifics’) metabolic product. Most autotrophic microbes (especially prokaryotes) perform bioenergetics through their plasma membranes, which means that their bioenergetic production is directly dependent on cell surface area over which essential resources are taken up [54]. Having partners attached to the surface imposes a severe trade-off, and it is not trivial when it is beneficial to pay the cost of reduced consumption despite reduced self-inhibition. Accordingly, we consider that the consortial host’s external surface area occupied by ectosymbionts is (and could have been) crucial in the emergence of the stable, obligate ectosymbiotic consortium.

## Results

We tested our hypothesis within a model where metabolically cooperating species may form physical associations, increasing the contact surface of partners. We analyzed whether a mutant host, with the capability to capture or bind symbionts on its external cell surface, can invade a system resided by free-living host and symbiont species. Our methodology relies on density-dependent ecological (two-species) dynamics determining mutant invasion, aided by metabolite concentration dynamics. Under this assumption, we checked whether physical coupling is selected for, given the possibility of the host species to associate with free-living symbionts. Alternatively, we check whether such association becomes a hindrance due to the increased cost realized in reduced consumption rate for the mutant host. If the host is better off with increased consumption and baseline metabolic partnership, we expect that physical coupling will not fix in the population and metabolically dependent species continue their free-living lifestyle (as extant syntrophic prokaryotes do).

**Fig 1:**
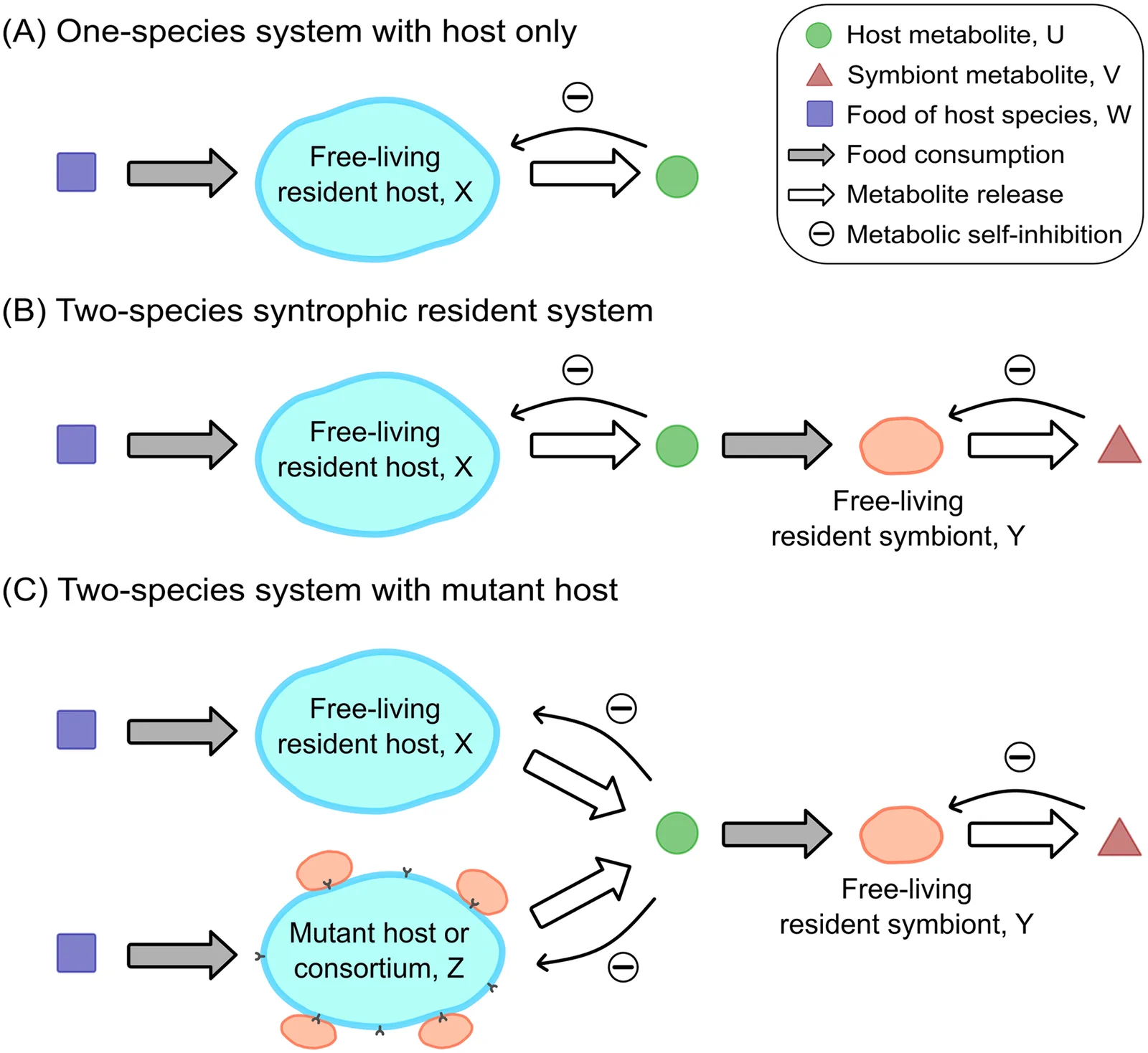
The three evolutionary steps toward ectosymbiotic consortium formation. **(A)** Initially, there is a single host species producing a metabolite that exerts self-inhibition (metabolic growth inhibition on the producer). **(B)** When the symbiont species is added to the system, it alleviates the effect on the host by consuming its self-inhibiting product, establishing unilateral syntrophy. The symbiont in turn produces a self-inhibiting metabolite too. **(C)** Mutant hosts capable of forming consortium may appear and invade the population of symbiotic syntrophs, leading to explicit ectosymbiosis. Mutant hosts always form consortium; the two terms are interchangeably used in the model descriptions and equations.

We model the ecological scenario leading to the formation of ectosymbiotic consortium from physically independent (free-living) individuals of two distinct species in three steps. We start with a system with a single species (host), enduring self-inhibition of growth by its metabolic product (Fig 1A). Next, we consider a system with a new species (symbiont), enjoying benefits of an obligate syntrophic relationship with the host species (Fig 1B). A stable ecological coexistence of the two free-living species in this system guarantees the maintenance of syntrophy between them. Finally, we check whether a mutant host capable of initiating ectosymbiosis with the free-living symbionts (forming a two-species ectosymbiotic consortium) can invade the syntrophic resident system of free-living individuals (Fig 1C). The mutant population newly introduced to the system adds a dimension to the existing resident system, extending the ecological dynamics. The outcomes of ecological selection are based on the stability of the fixed points of the resident-mutant dynamical system [55–58]. We focus on the fixation of the ectosymbiotic consortium in the system based on the stability of the fixed point with viable mutant host population density. The conditions for the *substitution* [56] of the resident host by its mutant phenotype reveal the potential driving factors behind a stable ectosymbiotic consortium. Fig 1 is a schematic representation of the three ecosystems we consider. For details of the individual systems, see Methods and S1 Appendix.

**Fig 2:**
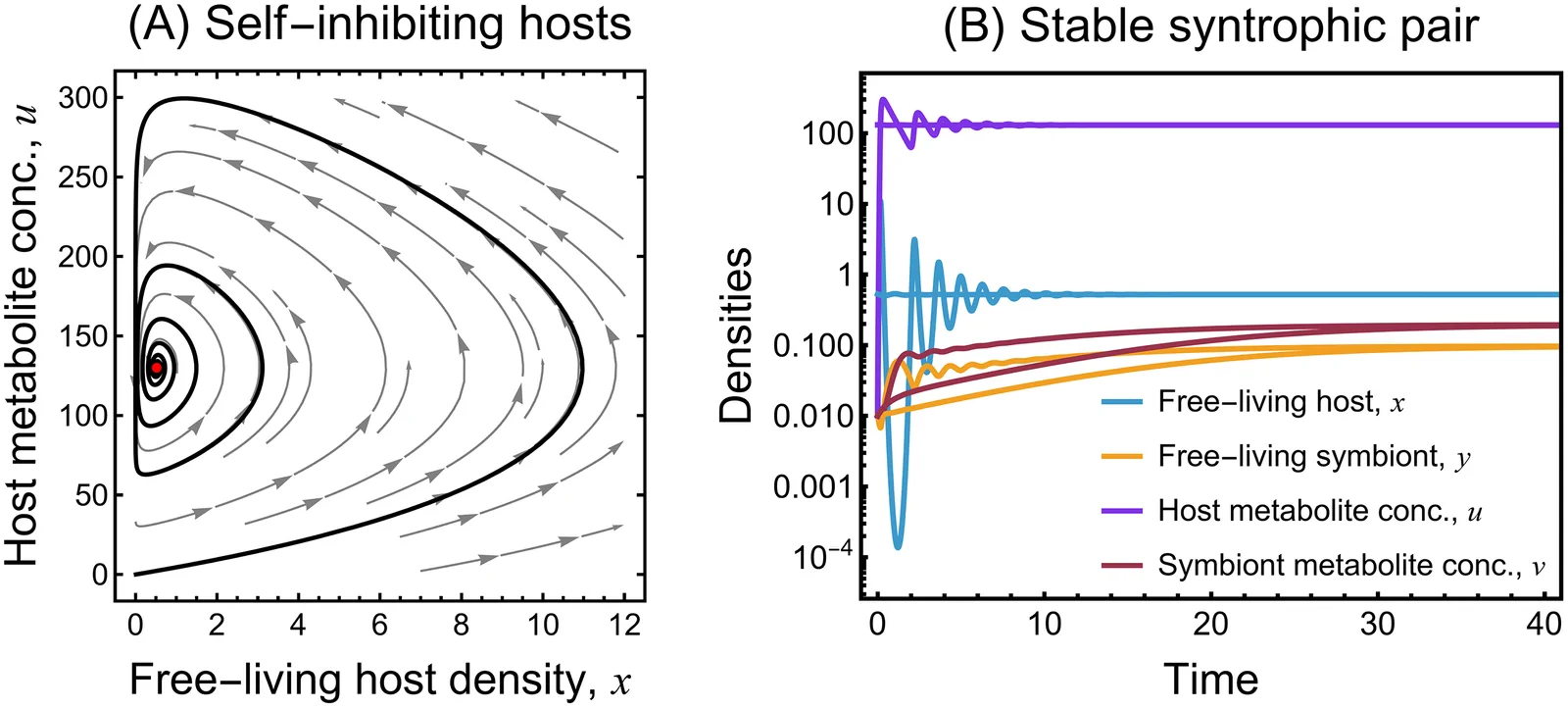
Stability of self-inhibiting host species and coexistence of host-symbiont pair. **(A)** Phase portrait of the one-species system with the trajectory (colored black) starting from an arbitrary point in the neighborhood of the trivial fixed point. The dynamics stabilizes at the unique interior fixed point $\left(x^{*},\;u^{*}\right)=(\text{0}.\text{5}\text{2},\;\text{1}\text{3}\text{0})$ (red point). The trivial fixed point (0, 0) is always unstable. **(B)** Solution of the two-species resident system showing the stability of the unique interior fixed point $\left(x^{+},\;y^{+},\;u^{+},\;v^{+}\right)=(\text{0}.\text{5}\text{2},\;\text{0}.\text{0}\text{9}\text{7},\;\text{1}\text{3}\text{0},\;\text{0}.\text{1}\text{9}\text{5})$, where both X and Y survive. For arbitrary points close to the trivial (0, 0, 0, 0) and one-species resident $\left(x^{*},\;\text{0},\;u^{*},\;\text{0}\right)$ fixed points as the initial conditions, the dynamics stabilizes at $\left(x^{+},\;y^{+},\;u^{+},\;v^{+}\right)$, while (0, 0, 0, 0) and $\left(x^{*},\;\text{0},\;u^{*},\;\text{0}\right)$ are unstable. Arbitrary values of the parameters are as listed in Table 1. See Appendix A1 and A2 in S1 Appendix for more details on stability analysis of the two systems. Host and symbiont can stably coexist if symbiont depends on host’s product and metabolic self-inhibition limits population growth.

The one-species resident dynamical system (Eq 3) of host population density and metabolite concentration is analyzed for the stability of its unique interior fixed point, ensuring limited growth and stabilization of the host (species X) population. The unique interior fixed point exists if and only if the growth rate of species X is less than the maximal growth inhibition (otherwise, the population density explodes). The interior fixed point, if it exists, is unconditionally stable (see Fig 2A). This shows that metabolic self-inhibition is sufficient to prevent population explosion and stabilize population growth.

According to the fixed-point analysis of the two-species resident system (Eq 4), either the host X survives alone (as in the previous one-species system), or the host X and the symbiont Y (both free-living) coexist. Unlike the host species, the symbiont cannot survive entirely on its own (i.e., free of X) as it depends on the host metabolite U for food. Note that this coexistence is not ectosymbiotic as there is no physical attachment between the host and the symbiont yet. The existence of the interior fixed point of the two-species system depends on how the host’s life history parameters are within bounds governed by the symbiont’s life history parameters (see Appendix A2 in S1 Appendix). The trivial and one-species fixed points are always unstable; while the interior fixed point, if it exists, is always stable unconditionally (see Fig 2B). Apparently, the presence of a syntrophic species destabilizes the previously stable one-species system; the syntrophic pair dominates the host-only system. Hence, metabolite-induced self-inhibition coupled with unidirectional syntrophy can lead to the ecological coexistence of the free-living host and symbiont species (also see [38]).

**Fig 3:**
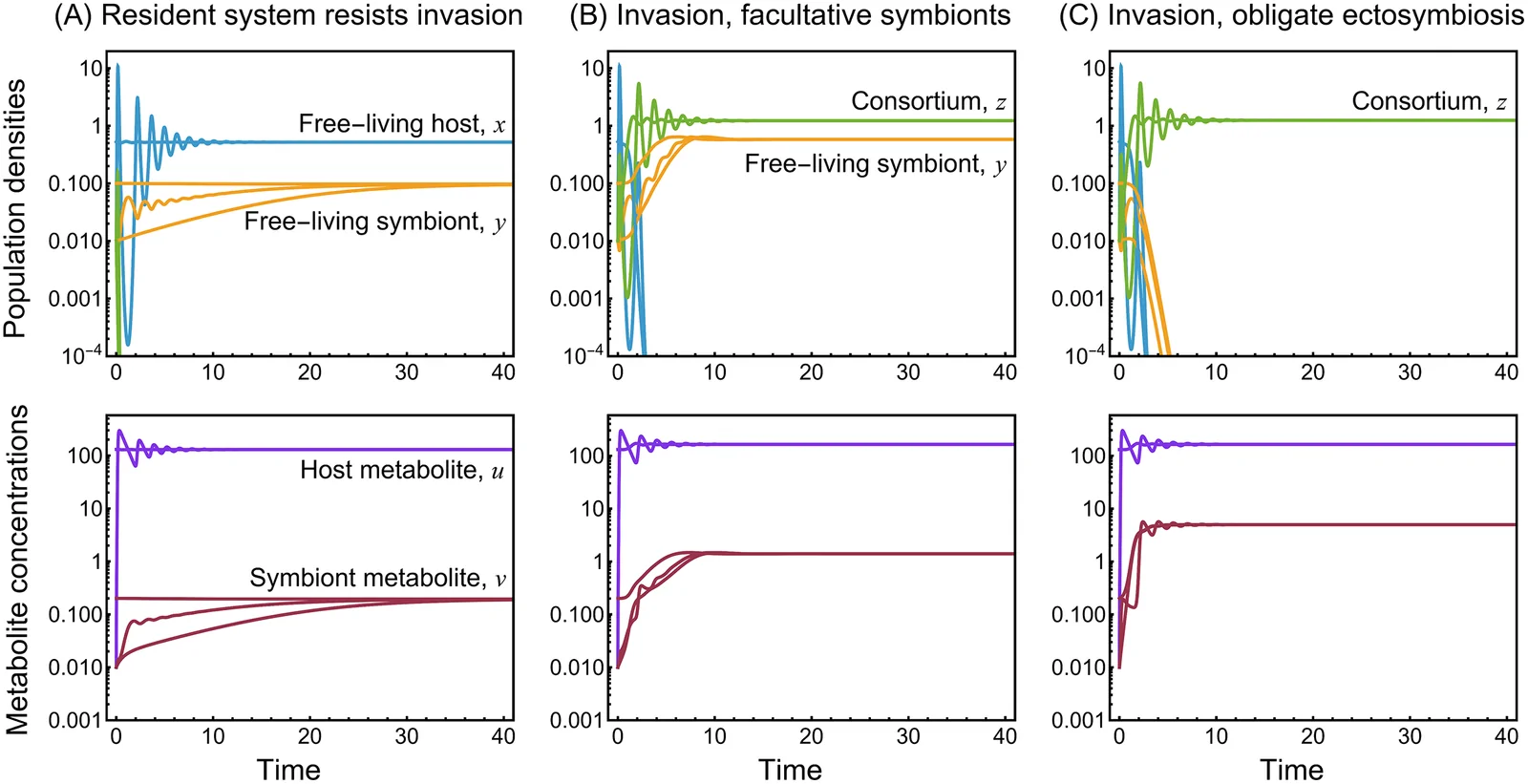
Solutions of population densities and metabolite concentrations in the resident-mutant dynamics. The parameters and the arbitrary values are listed in Table 1. The initial conditions are arbitrary points close to the trivial $E_{\text{0}}(\text{0},\;\text{0},\;\text{0},\;\text{0},\;\text{0})$, one-species resident $E_{\text{1}}\left(x^{*},\;\text{0},\;\text{0},\;u^{*},\;\text{0}\right)$, and two-species resident $E_{\text{2}}\left(x^{+},\;y^{+},\;\text{0},\;u^{+},\;v^{+}\right)$ equilibria. **(A)** The dynamics stabilizes at $E_{\text{2}}=\left(x^{+},\;y^{+},\;\text{0},\;u^{+},\;v^{+}\right)=(\text{0}.\text{5}\text{2},\;\text{0}.\text{0}\text{9}\text{7},\;\text{0},\;\text{1}\text{3}\text{0},\;\text{0}.\text{1}\text{9}\text{5})$ for s=0.2, $\beta_{\text{Z}}=\text{0}.\text{2}$, and $k_{\text{YZ}}=\text{0}.\text{0}\text{5}$. **(B)** The dynamics stabilizes at $E_{\text{P}}=\left(\text{0},\;\hat{y},\;\hat{z},\;\hat{u},\;\hat{v}\right)=(\text{0},\;\text{0}.\text{5}\text{7}\text{9},\;\text{1}.\text{2}\text{2}\text{4},\;\text{1}\text{6}\text{4},\;\text{1}.\text{4}\text{0}\text{3})$ for s=0.4, $\beta_{\text{Z}}=\text{0}.\text{2}$, and $k_{\text{YZ}}=\text{0}.\text{0}\text{5}$. **(C)** The dynamics stabilizes at $E_{\text{M}}=\left(\text{0},\;\text{0},\;\overset{―}{z},\;\overset{―}{u},\;\overset{―}{v}\right)=(\text{0},\;\text{0},\;\text{1}.\text{2}\text{4}\text{4},\;\text{1}\text{6}\text{4},\;\text{4}.\text{9}\text{7}\text{7})$ for s=0.4, $\beta_{\text{Z}}=\text{4}$, and $k_{\text{YZ}}=\text{0}.\text{0}\text{6}$. See Appendix A3 in S1 Appendix for more details on stability analysis. Increased surface coverage stabilizes consortium, and the effective syntrophy parameters of the ectosymbionts promote population-level obligacy leading to exclusion of free-living individuals. The ectosymbiotic consortium can tolerate higher concentrations of metabolites. Mutant invasion (stable $E_{\text{M}}$ or $E_{\text{P}}$) depends on the geometric benefit from inhibition reduction, and effective syntrophic parameters lead to extinction of the free-living individuals (stable $E_{\text{M}}$).

The resident-mutant system (Eq 7) with the mutant host can have five ecologically feasible fixed points. The trivial fixed point $E_{\text{0}}$ and the one-species fixed point $E_{\text{1}}$ are always unstable. The consortium-only fixed point $E_{\text{M}}$, where the two species survive only as the ectosymbiotic consortium formed by the mutant host, exists if and only if the mutant growth rate is less than the maximal growth inhibition and is conditionally stable. A fixed point $E_{\text{P}}$ with symbiont species in both free-living and ectosymbiotic forms could also exist conditionally and be stable. The mutant invasion condition depends on the growth rates and growth inhibition factors of the consortial (Z) and free-living (X) hosts. The two-species resident fixed point $E_{\text{2}}$, where the consortium-forming mutant host perishes, is stable if the invasion condition is not satisfied. For more details on the existence and stability conditions of the fixed points, see Appendix A3 in S1 Appendix. Fig 3 gives the solutions of the resident-mutant system corresponding to the stability of three possible fixed points.

**Table 1:** Variables and parameters of the model. The parameter values are qualitatively compatible with the model descriptions and are used to visualize and verify analytical results. The values are based on internal model feasibility rather than any empirical data. All our analytical results are valid for any range of biologically feasible parameter values. See Supporting Information S1 Appendix and S1 Code for information on choice of arbitrary parameter values, conditions determining parameter bounds and feasible ranges, and numerical analyses.

Variables and parameters	Meaning	Value set definition	Arbitrary parameter values
x(t)	Population density of free-living host X	$R_{\geq \text{0}}$	
y(t)	Population density of free-living symbiont Y	$R_{\geq \text{0}}$	
z(t)	Population density of ectosymbiotic consortium (or mutant host) Z	$R_{\geq \text{0}}$	
u(t)	Concentration of host metabolic product U in the habitat	$R_{\geq \text{0}}$	
v(t)	Concentration of symbiont metabolic product V in the habitat	$R_{\geq \text{0}}$	
w	Constant concentration of food W available to the hosts	$R_{+}$	500
Ω	Volume of the habitat or medium	$R_{+}$	10
$k_{\text{X}}$	Consumption rate of W by free-living host	(0, 1)	0.5
$k_{\text{Y}}$	Consumption rate of U by free-living symbiont	(0, 1)	0.1
$k_{\text{Z}}$	Consumption rate of W by ectosymbiotic consortium	(0, 1)	0.3
$k_{\text{YZ}}$	Consumption rate of U by ectosymbionts	(0, 1)	0.05, 0.06
$l_{\text{X}}$	Cost of living of free-living host	$R_{+}$	4
$l_{\text{Y}}$	Cost of living of free-living symbiont	$R_{+}$	1.2
$l_{\text{Z}}$	Cost of living of ectosymbiotic consortium	$R_{+}$	5
$c_{\text{X}}$	Cost of reproduction of free-living host	$R_{+}$	3
$c_{\text{Y}}$	Cost of reproduction of free-living symbiont	$R_{+}$	0.3
$c_{\text{Z}}$	Cost of reproduction of ectosymbiotic consortium	$R_{+}$	4
$d_{\text{X}}$	Intrinsic death rate of free-living host	(0, 1)	0.33
$d_{\text{Y}}$	Intrinsic death rate of free-living symbiont	(0, 1)	0.1
$d_{\text{Z}}$	Intrinsic death rate of ectosymbiotic consortium	(0, 1)	0.4
$a_{\text{X}};b_{\text{X}}$	Coefficients of self-inhibition by U on free-living host	$R_{+};\;(\text{0},\;\text{1})$	1; 0.01
$a_{\text{Y}};b_{\text{Y}}$	Coefficients of self-inhibition by V on free-living symbiont	$R_{+};\;(\text{0},\;\text{1})$	1.5; 0.015
s	Surface impact on growth inhibition by U on host (per ectosymbiont)	(0, 1)	0.2, 0.4
n	Average number of ectosymbionts in a consortium	$R_{+}$	2
$p_{\text{Z}}$	Surface impact on consumption of W by the consortium	(0, 1)	0.2
$p_{\text{YZ}}$	Surface impact on consumption of U by the ectosymbionts	(0, 1)	0.5, 0.6
$\beta_{\text{Y}}$	Production coefficient of symbiont metabolite V by free-living symbiont	$R_{+}$	2
$\beta_{\text{Z}}$	Production coefficient of symbiont metabolite V by the consortium	$R_{+}$	0.2, 4
$\delta_{\text{U}}$	Decay coefficient of host metabolite U	$R_{+}$	1
$\delta_{\text{V}}$	Decay coefficient of symbiont metabolite V	$R_{+}$	1

### Emergence of consortium

**Fig 4:**
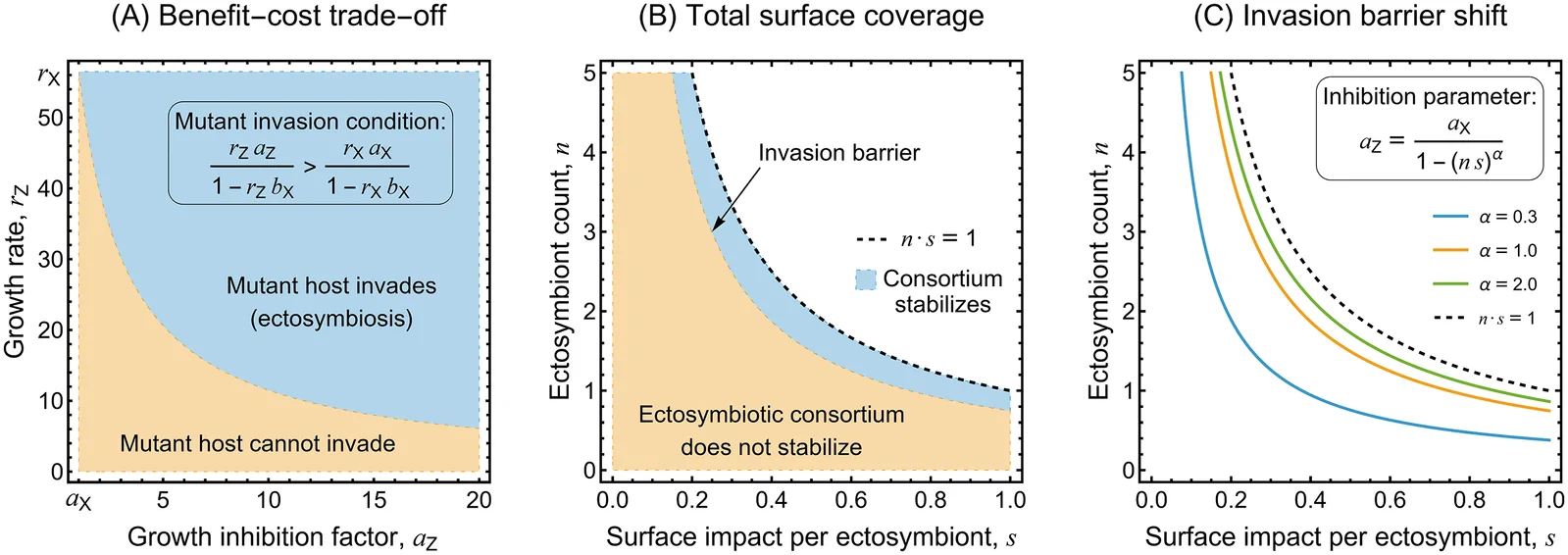
Condition for stable consortium. Region plots representing parameter values corresponding to consortium-forming mutant invasion. $r_{\text{X}}$ is the growth rate of the resident hosts, and $a_{\text{X}}$ and $b_{\text{X}}$ are their growth inhibition parameters. For parameter values, see Table 1. **(A)** The invasion condition is based on the growth rate and growth inhibition factor of the mutant host (Appendix A3 in S1 Appendix). The ectosymbiotic consortium can potentially fixate on account of the benefit of reduced self-inhibition due to the occupied external surface area of the hosts. **(B)** Average ectosymbiont count on a mutant host (n) is plotted against surface impact on the growth inhibition of the mutant host induced per ectosymbiont on its surface (s). High surface coverage promotes consortium fixation. **(C)** Shift in invasion boundary for varied dependence of growth inhibition on occupied surface is plotted. Mutant invasion persists regardless of the linear geometry of inhibition reduction; the feasible region shifts quantitatively with non-linear (α≠1) and biophysically plausible scaling.

The mutant invasion condition guaranteeing a viable consortium is satisfied by high values of mutant growth inhibition factor compared to that of the resident hosts. In other words, reduced growth inhibition (or high values of a, i.e., $a_{\text{Z}}$ relative to $a_{\text{X}}$) can facilitate the fixation of consortium as represented in Fig 4. If the invasion condition (Appendix A3 in S1 Appendix) is not satisfied, the consortium-forming mutant host cannot invade, and the two-species resident equilibrium remains stable; the resident syntrophic system is thus resistant to invasion by the mutant host (i.e., coexistence of free-living species is stable against ectosymbiosis). Now note that the higher the contact surface per ectosymbiont (s) and ectosymbiont count (n), the higher the total surface coverage by the ectosymbionts. As a result, reduced exposure to toxic metabolites or equivalently reduced growth inhibition is realized when the total surface area of contact is high. Fig 4B shows the parameter space in terms of s and n where the consortium can stabilize based on higher surface coverage. Note that our results are regardless of linear dependence of self-inhibition on surface coverage (Fig 4C). In essence, a mutation in the host that reduces the net growth inhibition by sufficiently protecting it from the self-inhibitory effects of its own metabolites can fixate if these benefits outweigh the costs suffered in the form of a reduced growth rate (Fig 4A). Inhibition reduction in the hosts, due to surface coverage while in ectosymbiosis, can be a driving factor in stabilizing the symbiotic consortium, despite the immediate reduction of energetically and metabolically active cell membrane surface (also note that the dependence of host’s benefit and cost on the surface coverage factor are separately adjustable in the model). Additionally, the consortial host cannot invade if the self-inhibitory metabolic effect is replaced by a spontaneous mechanism for limiting population growth: intraspecies competition where the host phenotypes differ only in their costs (Eq 8; also see Appendix A5 in S1 Appendix). Self-inhibition and its reduction thus play a key role in stabilizing the consortium.

The significance of contact surface in reducing growth inhibition is a reasonable incentive behind the emergence of the ectosymbiotic consortium. Furthermore, this also points to the possible evolution of enhanced invagination or membrane protrusions as mechanisms to further increase contact surface. Such mechanisms likely precede direct internalization of the syntrophic ectosymbionts by the host, which can eventually pave the way for endosymbiosis, despite entailing a necessary forfeit of free surface area as species growth rate depends on active (unoccupied) cell surface. Note that the idea generally applies to any microbial syntrophy, not just for the presumed syntrophic origin of mitochondria. Accordingly, we predict that microbial syntrophic partnerships (when the above condition of invasion is met) evolve to increase surface contact area (and also time spent attached to host) through modulating cell-shape, invagination, engulfment, or other means (also see [35]). Recently discovered syntrophic Asgards of complex-morphologies [19] suggest that their cellular protrusions may serve exactly this purpose. Our idea is potentially testable in vitro with naturally occurring or artificially assembled symbioses.

### Emergence of population-level obligate ectosymbiosis

Based on the condition for the exclusive stability of the consortium-only equilibrium $E_{\text{M}}$ (Appendix A3 in S1 Appendix), the lower the release of the metabolite U into the environment by the consortium, the higher the chance of consortial fixation. A high rate of host metabolite consumption by the ectosymbionts in the consortium ($k_{\text{YZ}}$) reduces the release of metabolite U into the habitat and favors the stability of the $E_{\text{M}}$ equilibrium by lowering metabolite release. Similarly, a higher production rate ($\beta_{\text{Z}}$) of the symbiont metabolite V by the consortium can also facilitate the exclusive stability of $E_{\text{M}}$. In other words, if the ectosymbionts are metabolically active and effectively consume and metabolize U produced by the consortial host, the consortium-only equilibrium is stable. In this case, the free-living symbiont density collapses due to the constrained availability of resource U in the environment and consequent competitive exclusion. Hence, fixed point $E_{\text{P}}$ with the facultative existence of the symbiont species (i.e., free-living and ectosymbiotic forms) is not viable. Additionally, in the consortium-only equilibrium, the symbionts are only present in their ectosymbiotic form; the free-living ones go extinct. This means that the stability of the fixed point $E_{\text{M}}$ also ensures (and forces) a single mode of living for the symbionts, thereby developing a population-level obligate ectosymbiosis with the host. High dilution of host metabolite in the habitat (as a result of large habitat volume) also promotes the extinction of free-living types (Fig A1 and Appendix A4.1 in S1 Appendix). The ectosymbionts, with direct access to the metabolite, are at an advantage here and could thrive regardless of the dilution. In essence, effective utilization of host metabolite and subsequent release of symbiont metabolite by the ectosymbionts promote obligate ectosymbiosis with their mutant host. Note that mutant invasion and facultative ectosymbiosis can happen over a wide range of parameter conditions. However, population-level obligate ectosymbiosis requires stricter and ideal conditions, for instance, synchronization in host-symbiont replication and perfect symbiont inheritance (respectively explained in Appendix A4.2 and A4.3 in S1 Appendix).

### Improved metabolic tolerance of consortium

The mutant hosts in the consortium-only state of the system (stable $E_{\text{M}}$) can tolerate higher concentrations of the host metabolite compared to the resident equilibrium (see Fig 3). The ectosymbionts protect the mutant host from the toxic metabolite in its environment (produced by host and its conspecifics), increasing their host’s metabolic tolerance. Previously, we observed that the consortial hosts can invade the resident system of free-living species if their ectosymbionts can sufficiently help them reduce the effect of metabolic self-inhibition. Also, such a mutant host could form obligate consortium if the dependent ectosymbionts are highly metabolically active. The fixation of the obligate ectosymbiotic consortium is enabled by inhibition reduction and a syntrophically solid relationship. The ectosymbionts protect the hosts by effectively consuming the host metabolite and reducing exposure to it, thereby enhancing the hosts’ tolerance.

## Discussion

We have modelled the scenario where a host species (a prokaryote, possibly an Asgard archaeon) develops the means to physically bind individuals of the free-living symbiont species on its plasma membrane to create a consortium. We were interested in whether this interaction can lead to stable coexistence of the consortium-forming host and its symbiont to the point where free-living phenotypes are lost from the population. In our model, the host produces a waste molecule that limits its own growth if accumulating in the vicinity. This molecule, however, is utilized by the symbiont species that can metabolize the inhibitive product; thus, alleviating its effects on the host individual. The syntrophic association is ecologically stable even though the host species was previously viable on its own. The mutant host and the symbiont can form a physically associated, intimate relationship by the host binding the symbiont to its outer surface. The ectosymbiont not only lowers the concentration of the toxic by-product by taking it up but also protects the host from the same inhibitive metabolite produced by the host’s conspecifics in the neighborhood. The magnitude of this effect is the key determinant of the possible outcomes of ecological selection in our model. While this cooperative help has explicit benefits, it also comes with a larger metabolic cost and reduced metabolically active cell surface. The three possible outcomes are as follows: 1) ectosymbiosis does not stabilize, i.e., the consortium-forming mutant host cannot invade; 2) ectosymbiosis stabilizes with both free-living and ectosymbiotic symbionts surviving; and 3) only ectosymbiotic consortium survives, resulting in population-level obligate ectosymbiosis. Note that we do not claim that these are necessarily evolutionary endpoints, however these are possibilities of stabilization of ecological dynamics. We do not assert any mechanisms on how the mutant emerges or that the invasively successful mutant host could resist invasion by a new mutant later. We showed that the ectosymbiotic mutant could invade and persist against already stable free-living hosts, ultimately replacing the resident population. This suggests that ecologically stable ectosymbiosis from free-living parties can serve as the first step or starting point in the potential (hypothetical) evolutionary trajectory enabling physical integration. Here, inhibition combined with metabolic syntrophy acts as the ecological driver. Subsequent mutants with better integration (e.g., host capable of invaginating the ectosymbiont) could be predicted to stabilize in the same environmental setup based on their selective advantage in reducing inhibition, leading to successive replacements under selection. We found that stable ectosymbiotic consortium can emerge if the net inhibition reduction by the ectosymbionts is large enough to compensate for the inferior growth rate of the consortium-forming phenotype compared to the free-living resident hosts.

Our model assumes that the mutant host has a novel mechanism expressed on its external surface to bind symbiotic cells. While in biofilms, associated prokaryotes stay together because of the matrix they produce [59]; there are many dedicated mechanisms for prokaryotic cells to attach to each other. For example, archaea can attach to surfaces or to other cells by hami- [60], flagellae- or pili-like structures [61–63]. In biofilms of *Pyrococcus furiosus* and *Methanopyrus kandleri*, apart from pili, a yet unknown contact molecule acts as binder [64]. Bacterial dynamin-like proteins 1 (DLP1) bind to membranes, and the DLP1/DLP2 complex can tether two membranes together [65]. Coiled-coil structures with globular heads at their end can connect two membranes at a distance [66] (they are mostly involved in the functioning of the Golgi apparatus). Based on the above biological examples, it is not far-fetched to assume a small change in a surface protein (or a cytosolic protein that begins to be expressed on the membrane) of the host initiates a more intimate connection with a partner cell.

The connection with the host reduces the active cell surface, thus the connection must come with a metabolic price. Most prokaryotes feed through their plasma membranes, and the surface area affects their metabolic production. In prokaryotes, especially in autotrophs, growth rate is strongly dependent on cell surface area [67]. However, we also point out that our model is not specific on molecular surface structures in binding partner cells. As a matter of fact, any surface mechanism that keeps partner cells attached to the host suffices. For example, membrane invaginations or protrusions can serve to capture the partner cells and keep them attached to the host. While there exist such mechanisms [19,26,68], admittedly, it is unknown if they are capable of ensuring long-term coexistence of parties. Such mechanisms, if they were ever involved in establishing obligate ectosymbioses, are beneficial over simple surface attachments due to at least two reasons. First, they may be able to compensate for the loss of active cell surface for the host; second, they may better ensure vertical inheritance if the symbiont is enclosed such that it cannot escape. Either way, the model indicates the significance of the area of surface contact between parties in the evolution of ectosymbioses.

Our model ignores the individual dynamics (reproduction, death, escape, etc.) of the attached ectosymbionts. While this is a must for the model to remain tractable, it is also justifiable. We assume that as the host cell reproduces, it doubles its genome and materials. This means that with every division, the cell membrane is also doubled in size. Accordingly, we assume that as the host increases its surface area, the number of attachment points also increases. Immediately after division, a cell surface of S can hold n ectosymbionts; while immediately before division, we assume that 2S can hold 2n ectosymbionts. Assuming that the membrane is fluid, attachment points are distributed evenly on the surface; as a result, daughter cells after division receive equal amounts: n ectosymbionts. Any excess replication of the ectosymbionts when attached to the surface is ignored as, if offspring cannot fit over the host, it is released to the external symbiont pool which is assumed to be much larger. Admittedly these are simplifications, but this approach does not require synchronized cell cycles of host and symbiont. Note that we do not assume perfect vertical inheritance as we do not specify if the same symbionts are present always or occupy specific attachment points, they may come and leave independently and randomly. The important point is that a number of them are always attached to the host after a random assortment, implementing thus a poor man’s passive vertical inheritance. An altered model considering unsynchronized host-symbiont cell replication was numerically analyzed with a symbiont escape function added to the free-living symbiont dynamics. Given the symbionts are relatively smaller to the hosts in terms of size, it is reasonable to assume a faster symbiont replication as opposed to synchronized host-symbiont cell-cycles. Analysis of the altered model suggested that obligate ectosymbiosis with ecological exclusion of the free-living types remains an outcome only if the release or escape factor is negligibly low (Fig A2 and Appendix A4.2 in S1 Appendix). This approach indirectly captures the ectosymbiont dynamics and indicates that, in our model setting, synchronized host-symbiont replication is needed for the consortium-only outcome. Furthermore, by relaxing the assumption of perfect and completely reliable vertical inheritance of symbionts from the consortial hosts to their offspring, we observed that high reliability or accuracy is necessary to guarantee the stability of the consortium (Fig A3 and Appendix A4.3 in S1 Appendix). Increasing the accuracy of vertical inheritance above a critical value also ensures decline of the free-living populations, and the consortium-only outcome is obtained only if the symbionts are perfectly inherited. Consortium is stably maintained only if the ectosymbionts are adequately inherited to its daughter cells during division (i.e., group selection is in effect). Further analysis could include a binomial treatment of symbiont attachment-detachment dynamics and discrete ectosymbiont subpopulations with their spatial distribution (variance in ectosymbiont count). However, this is beyond the scope of this paper, requiring an entirely different model.

In our model, the different host phenotypes (consortial and non-consortial) do not compete for any resource (neither for their primary food nor for symbionts). Any such competition would further help the consortial host in channeling away free symbionts, reducing external symbiont population. This in turn is expected to increase inhibition for the non-consortial hosts that rely explicitly on free-living symbionts for reducing the toxic metabolite concentration. However, since the free-living symbionts are assumed to be abundant compared to the hosts, the symbiont acquisition (or privatization of the public good) by the consortial hosts does not harm the non-consortial hosts notably. A tragedy-of-the-commons situation for the non-consortial hosts does not emerge. The ectosymbionts on a mutant host modify only the host’s own local self-inhibition; they do not redistribute or affect the benefit from symbiont pool or ectosymbiotic shielding to other hosts (either resident or mutant). The privatization aspect also does not affect the mutant’s invasion. This is because a host’s self-inhibition depends only on the external metabolite concentration and is not directly modified by any interference from other hosts (except via their contribution to the metabolite bulk). Additionally, even with a finite symbiont supply, invasion of the mutant, consortial hosts could happen (see S1 Code). Our results also show that there is no mixed coexistence where both host phenotypes can survive.

In a previous work [43], a theoretical model with a unidirectional syntrophic relationship of two physically independent host and symbiont species, where the population growth was limited by intraspecies density-dependent competition instead of self-inhibition, was considered. Similar to the study in this paper, the possible invasion of a mutant forming an ectosymbiotic consortium was analyzed; however, there it was assumed that the mutation emerges in the symbiont species and not in the host, serving as a crude model of the parasitic invasion theory [37,69]. It was observed that the metabolic activity and the additional costs to the mutant symbiont (compared to the resident) determine the stability of the consortium [43]. However, mutant symbionts, while replacing the residents, survived in both free-living and ectosymbiotic forms. No obligate association of the ectosymbiotic consortium emerged in the model [43]. However, in this paper, we have shown that an obligate ectosymbiotic association with ecological exclusion of free-living types can stabilize. Note that our model requires differentiation between the usage of *obligacy* in four contexts: 1) obligate metabolic relationship where partners cannot survive without other’s metabolite, 2) obligate physical attachment where partners cannot replicate without the other, 3) population-level obligate ectosymbiosis where partners cannot exist in free-living form, and 4) genome-level obligate symbiosis where partners complement for gene loss in the other. In the model, the stable resident equilibrium corresponds to an obligate metabolic dependence, and the stable consortium-only equilibrium corresponds to population-level obligate ectosymbiosis where the free-living individuals are extinct. Though our model does not consider an explicit mechanism to capture obligate physical attachment, population (ecological) dynamics and extinction of free-living symbionts at the stable consortium-only equilibrium effectively ensure a scenario where symbionts cannot replicate without the host (however, note that this does not imply any loss of autonomy or genotype-level obligacy). We have not included an obligacy in genotype-level dependence, considering that this could coevolve following exclusive, prolonged coexistence of the host-symbiont ectosymbiotic consortium. This is why we present our general model based on syntrophy and ectosymbiosis as potentially a pre-requisite (or first step) to eukaryogenetic endosymbiosis [20,21,33,34], without considering physical inclusion or vertical inheritance. Here, coevolutionary adaptations are a consequence of group-selection of the consortium as a single unit. We have not modelled or demonstrated the later transitions; instead, we focused only on the first transition for a better understanding of the ecological driver and direction of selection. Eukaryogenesis here serves only as a paradigm of a stable physical integration of free-living partners. Our results primarily demonstrate the ecological conditions for a consortium-forming (ectosymbiotic or early integration) trait to invade, without assuming any mechanisms to physically engulf the partner.

Despite significant advances in research, the origin of the eukaryotic cell still remains an enigma; most importantly, the basis of the permanent association and the mechanism of entry of the bacterial partner remains unknown. A common idea in most theories about the origin is that syntrophy *somehow* led to the endosymbiosis of the early partners. This idea is prevalent in many forms [19–23,26,31,33–35,37], despite the fact that no syntrophic prokaryotes are known yet that have concluded their merger by provably forming a higher level unit of evolution. Most of the known endosymbionts of unicells are known to have entered their host via the phagocytotic mechanism, e.g., all plastids [70–73]. However, prokaryotes lack a homologous mechanism which may account for the lack of endosymbioses among them. While there is at least one known analogous endocytic mechanism in prokaryotes [74], it is neither on par with eukaryotic phagotrophy nor is it known to lead to (or facilitate) endosymbiotic integration; most importantly, there are no such mechanisms known in Asgards, the closest archaeal relatives of eukaryotes [75]. While the last eukaryotic common ancestor might have already had phagocytotic capabilities [76,77], we do not yet know if it evolved before or after the integration of the mitochondrial ancestor (also see [27,78–80]). Some Asgards, though possess rudimentary cytoskeleton and relevant proteins [19,81–83], have not shown endocytic capabilities so far [79]. Consequently, as of now, we still don’t know the mechanism of entry of the bacterial symbiont into the ancestor of eukaryotes. The phagotrophic scenario [84–86] was already explored in a model by Zachar et al. [32]. If, however, we assume phagotrophy was a late eukaryotic invention after mitochondria (e.g., [87]), then we must account for an alternative mechanism on how the host has captured and internalized its symbiont. Furthermore, we must point out that metabolic dependence, capture (or binding), and internalization are distinct steps that the different scenarios explain in different orders.

Besides phagotrophy, the two main contenders of endosymbiotic inclusion are parasitic invasion [69] and syntrophic engulfment [19,31,88]. While we note that *engulfment* is not a very well-defined term, there are nevertheless at least two reasons why syntrophy seems to be the more likely candidate for initial interaction of ancestral parties. First, parasitic infection is a disadvantage to the host that is unlikely to be selected for; contrarily, a neutral or even beneficial metabolic interaction seems to be a better starting point, especially if it is already mutual at the start [34–36,75,89]. Second, metabolic syntrophy is ubiquitous among prokaryotes to the point where it is intriguing why it has never triggered more endosymbioses that we know of [4,30]. Here, we focused on the latter theory, exploring the possibility that if metabolic dependence, at least unilateral, has already evolved between the parties, then physical attachment can lead to obligate partnership. We have tested the hypothesis that ectosymbiosis can emerge from metabolic cooperation.

A particularly relevant hypothesis to discuss here is the inside-out origin of eukaryotes [31,88], also consistent with the entangle-engulf-endogenize hypothesis [19]. This scenario assumes an initially metabolic interaction, likely mutually dependent and beneficial for both parties, that evolved to more intimate physical entanglement. Ultimate integration happened when the host’s protrusions evaginated and enclosed the symbiont(s) and ultimately fused with itself, endogenizing the captured partners. There is no proof yet that such a fusing mechanism happens or could work in prokaryotes; the basic idea is the same as in all syntrophic scenarios: *somehow*, one partner was endogenized without an explicit phagocytotic mechanism. Without trying to explain the last bit, here we focused on how the metabolic interaction of two free-living species can trigger obligate membrane-bound ectosymbiosis. Our approach aligns with hypotheses assuming either an already-nucleated (though non-phagotrophic) or a prokaryotic host.

Our model explicitly assumes that genetic dependence has already evolved in the sense that symbionts depend on host cell products (however, hosts need not depend on symbiont as it can live freely, though with increased self-inhibition). These assumptions are well-supported by real-world examples. Association between prokaryotes, particularly between Archaea and Bacteria is ubiquitous [90]. The association can often be syntrophic, where the inhibitive waste product of one species is consumed by another [39,41,91–93]. For example, photosynthetic *Chlorobi* bacteria attach to a larger *β*-proteobacterium to form epibiotic consortia [13–15], or filamentous, sulphur-oxidizing γ-proteobacteria of the genus *Thiothrix* associates with coccoid euryarchaeota forming pearl-like structures [94–96]. In the latter, the bacterial partner possibly gives metabolites to the archaea and shields them from the aerobic environment. In this system, it is the bacteria which shields a multitude of smaller archaea. However, small, sulfur-oxidizing γ-proteobacterial cells can also be seen covering the surface of the giant archaeal cells of *Giganthauma karukerense* [16]. This arrangement is morphologically identical to the one proposed in our model. Even more similar examples are discovered among syntrophic Asgards too [19,75,97,98].

A special type of cross-feeding is the detoxification of inhibitory molecules, and the previous examples hint not only about potential metabolic interactions (syntrophy) and their benefits, but also about the adverse effect of products on one partner that can be eliminated by the other. For example, *Escherichia coli* produces acetate as a by-product of metabolizing glucose which, by changing the anionic environment, hinders its growth [99]. Another example, yeast (*Saccharomyces cerevisiae*) produces alcohol as a by-product of breaking down glucose; yet excess concentration of alcohol becomes toxic, inducing severe stress for the yeast [100]. *Bacillus cereus* MLY1 strain can degrade the polluting compound tetrahydrofuran; however, the resulting acidic environment makes it very inefficient. In the presence of *Rhodococcus ruber* YYL, metabolic cross-feeding, especially the removal of acidic compounds by *R. ruber*, makes the co-culture more efficient at biodegrading tetrahydrofuran [51].

The fully obligate ectosymbiosis (an outcome of our model) enables and may ultimately lead to vertical inheritance of the symbiont, thus creating a higher-level unit of selection (this transition remains to be seen either in the lab or in simulations). Moreover, successful host-symbiont integration also depends on the timing of evolution of the relevant innovations, for instance, the synchronization in replication, perfect (or effective) vertical inheritance, and the dependence between the hosts and symbionts in consortium [101]. With the fixation of the new evolutionary unit, selection for better-integrated interactions and novel features can become achievable. An upcoming paper is taking the modelling a step further toward physical inclusion, discussing the possibility of improved contact surface (as in membrane invaginations and protrusions) as a mechanism to counter growth inhibition.

## Methods

### Population growth rate

Metabolic consumption is key to our model and the population’s intrinsic growth is metabolite dependent. Hence, we utilize the Malthusian growth formulation [43], based on uniform and continuous resource consumption and cell replication of an asexually reproducing unicellular organism, which is defined as follows:


$$\begin{array}{c} \begin{array}{c} r=\frac{k\Pi -l}{c}\text{ln}\text{2}-d, \end{array} \end{array}$$ (1)

where k is the constant rate of resource consumption, Π the available resource concentration, l the cost of living or portion of per capita consumption required for cell sustenance, and d the intrinsic death rate. We assume resource flux depends linearly on the metabolite concentration. The organism is assumed to replicate (e.g., binary splitting [102]) upon acquiring a threshold of biomass represented as cost of reproduction c. Note that here population growth equation considering certain life history traits along with linear consumption results in scaling (ln2 factor in Eq 1) based on a branching process [43].

The organism, while benefiting from consuming the available resource, in turn produces a metabolic waste. The concentration of the metabolic product of the organism negatively impacts its growth, i.e., metabolite is self-inhibitory to the organism (e.g., alcohol produced by yeast [100]). Accordingly, we model the net growth rate of the producer declining with rising concentration of the produced metabolite (growth inhibition), bounding growth and potentially stabilizing the population.

### One-species system: self-inhibition by metabolite production

First, we analyze the behavior of a single-species system to examine whether growth inhibition by accumulated metabolite stabilizes its producers’ population as expected (Fig 1A). We assume a species X (would-be host) acquires their primary resource W from a replenishing source that maintains a constant available concentration w in the environment. Based on the description of the growth rate, $r_{\text{X}}=\frac{k_{\text{X}}w-l_{\text{X}}}{c_{\text{X}}}\text{ln}\text{2}-d_{\text{X}}$ is the per capita growth rate of species X. The organism’s metabolic product (or waste) U is released into the external environment at a rate proportional to its food uptake. The cumulative metabolite in the environment is toxic to the organism, restricting their population growth according to a non-linear function motivated by the Monod equation [103–105]:


$$\begin{array}{c} \gamma (u)=\frac{u}{a+bu}, \end{array}$$ (2)

where u is the total variable concentration of the released product in the environment, 1/b defines the value of maximal inhibition, and a/b is the half-saturation constant. We vary these factors to differentiate the metabolite-specific growth inhibition for different species. We also assume that the metabolite U decays at a constant rate ($\delta_{\text{U}}$). We propose the following dynamical model of a single host species X (with population density x) and metabolite U using a set of non-linear differential equations:


$$\begin{array}{c} \begin{array}{c} \begin{array}{c} \dot{x}=x\left(r_{\text{X}}-\frac{u}{a_{\text{X}}+b_{\text{X}}u}\right),\;\\ \dot{u}=k_{\text{X}}wx-\delta_{\text{U}}u, \end{array} \end{array} \end{array}$$ (3)

where all the parameters are strictly positive. More details in Appendix A1 in S1 Appendix. Note here that the intrinsic growth rate term is presented independent of any inhibitory effect.

### Two-species resident system: Syntrophy stabilizes

Now consider a second species Y (symbiont) forming a unidirectional and obligate metabolic relationship with species X (host) (Fig 1B). The symbiont Y consumes the metabolic product U of X. We assume that the metabolite U externalized by the host is diluted or evenly distributed in the habitat having volume Ω; hence, $k_{\text{Y}}u/\Omega$ is the effective per capita consumption of metabolite U in the medium by the free-living symbionts. Here, u/Ω is the net external concentration of U available to the free-living symbionts. The term reflects dilution of the metabolite, where the volume parameter is introduced to represent that the external metabolite is uniformly dispersed over the whole habitat volume. The scaling establishes that the resource concentration available to the symbionts is effectively lower unless they are in direct contact with the hosts. However, for the hosts, w represents the available concentration without the need for dilution factor scaling as the host food source is assumed to be continuously replenishing and constant. $r_{\text{Y}}(u)=\frac{k_{\text{Y}}\frac{u}{\Omega }-l_{\text{Y}}}{c_{\text{Y}}}\text{ln}\text{2}-d_{\text{Y}}$ is the metabolite-dependent per capita growth rate of species Y. The symbiont releases its metabolic product V into the environment at a constant rate ($\beta_{\text{Y}}$). Just as U inhibits X, V is inhibitory to the free-living symbiont growth. V also decays at a constant rate $\delta_{\text{V}}$. We denote y as the population density of free-living species Y and v as the concentration of the metabolite V in the habitat. The two-species extended system of the syntrophic pair is as follows:


$$\begin{array}{c} \begin{array}{c} \dot{x}=x\left(r_{\text{X}}-\frac{u}{a_{\text{X}}+b_{\text{X}}u}\right)\;,\\ \dot{y}=y\left(r_{\text{Y}}(u)-\frac{v}{a_{\text{Y}}+b_{\text{Y}}v}\right)\;,\\ \dot{u}=k_{\text{X}}wx-k_{\text{Y}}\frac{u}{\Omega }y-\delta_{\text{U}}u,\\ \dot{v}=\beta_{\text{Y}}y-\delta_{\text{V}}v, \end{array} \end{array}$$ (4)

where all the parameters are strictly positive. More details in Appendix A2 in S1 Appendix. The resource-consumer dynamics here assumes no timescale separation between the species’ population densities and the metabolite concentration dynamics (also compare with [38]). The syntrophic resident (monomorphic) system is analyzed to check for the stable coexistence of the host-symbiont pair.

### Mutant host invasion: Ectosymbiosis emerges

Next, we consider a mutant phenotype of the host invading the two-species syntrophic resident system (Fig 1C). Mutant invasions are usually rare, and we assume that the mutant emerges at an ecologically stable state of the resident system [57,58] and back mutation is unlikely. As a result, it is reasonable to assume that all mutant hosts could have descended from one mutant individual. The mutant hosts (denoted Z) have novel structures on their external surface to bind the free-living symbionts and initiate ectosymbiosis. The mutant host along with the ectosymbionts (*consortium*) is considered a higher-level organization (also see [17,18,106,107]). The mutant hosts are assumed to always form (and exist only in) consortium; hence, the two terms are interchangeable in our context. We also consider that the free-living symbiont pool is considerably larger than the host population. The consortium replicates based on the host characteristics, and its offspring remain in the same population group owing to a random assortment of symbionts on the host surface before and after replication. For the sake of simplicity, we do not explicitly model the ectosymbiont dynamics, particularly interspecific interactions leading to their formation, replication, and dissociation (more explanation in Discussion).

The mutant hosts feed on resource W and produce the self-inhibitory metabolite U as the resident hosts. The mutation to associate symbionts with the plasma membrane is, however, assumed to be costly, i.e., the costs of living and reproduction of the consortium-forming host Z are higher than that of the free-living resident X ($l_{\text{Z}}>l_{\text{X}}$ and $c_{\text{Z}}>c_{\text{X}}$). Additionally, accounting for the effect of the symbiont metabolite on the consortium’s net growth rate (due to the ectosymbionts), we consider a higher intrinsic death rate for the consortium ($d_{\text{Z}}>d_{\text{X}}$) to indirectly reflect that the consortium is worse off in that aspect. Otherwise, we assume the mutant host is identical to the resident. The presence of symbionts on the surface benefits the mutant host by providing them protection and reducing the local concentration of toxic metabolites released by X and Z. However, the presence of ectosymbionts also constrains the uptake of resource W by Z. Thus, the consumption rate of the consortial mutant host is lower than that of the free-living resident host ($k_{\text{Z}}<k_{\text{X}}$). The rationale behind these two effects is detailed next.

### Surface coverage causes inhibition reduction

The attached ectosymbionts reduce the free external surface area of their mutant host. As a result, there is an effective reduction in the exposure to the metabolite U in the environment. While in ectosymbiosis, contact reduces the self-inhibition on the mutant hosts induced by the external concentration of U. Let us denote by s∈(0,1) the surface area of contact (as a measure of impact on the growth inhibition of the host by metabolite U) per ectosymbiotic cell attached and the ectosymbiont count by n∈[1, 1/s). Without loss of generality, we consider unity as the host’s total external surface area. Here, 0<s<ns<1, where ns correspond to the impact of the total coverage by the n attached symbionts and we assume that complete surface coverage is never achieved. The consortial mutant host thus has a geometric benefit in terms of its reduced exposure to the self-inhibitory metabolite (compared to the free-living residents), which can be represented as follows:


$$\begin{array}{c} \gamma_{\text{Z}}(u)=\frac{(\text{1}-ns)u}{a_{\text{X}}+b_{\text{X}}(\text{1}-ns)u}=\frac{u}{a_{\text{Z}}+b_{\text{X}}u}, \end{array}$$ (5)

where $a_{\text{Z}}=\frac{a_{\text{X}}}{\text{1}-ns}>a_{\text{X}}$ and it increases with increasing total contact area. Recall that, for free-living resident hosts, $\gamma_{\text{X}}(u)=\frac{u}{a_{\text{X}}+b_{\text{X}}u}$ (see Eq 2). Here, note that $a_{\text{Z}}$ is not a trait variation of $a_{\text{X}}$; it stands for the geometric effect of inhibition reduction for the mutant. Recall we do not vary the inhibition parameters across phenotypes of a species by assumption (also compare with [103,105]), and hence $\gamma_{\text{Z}}(u)$ still uses $b_{\text{X}}$ and not $b_{\text{Z}}$. Different inhibition parameters for phenotypes of the same species imply that their metabolite’s effect on them is also dissimilar, rendering their comparison impractical. We assume that the metabolic production, utilization, and effect do not vary across the phenotypes; they differ only in their survival and fecundity characteristics. The asymmetric assumption $b_{\text{Z}}\text{≠}b_{\text{X}}$ requires a new mutant definition (e.g., how the mutant internally processes the inhibitive metabolite differently from the resident, instead of limiting the effect at the membrane as currently assumed) and consequently entirely different ecological dynamics. Accordingly, we have excluded this case here and discussed it in detail only in Appendix A4.4 (and Fig A4) in S1 Appendix.

### Biophysical mechanism of inhibition reduction

When symbionts attach to the mutant host surface, they create a protective microenvironment through two coupled effects. First, and most importantly, they actively consume the host metabolite within the host’s diffusive boundary layer [108–111], creating a depletion zone. Second, their physical presence reduces the host membrane area directly exposed to bulk concentrations of inhibitive metabolites. Both effects are captured in the model by the surface coverage parameter (ns), such that 1−ns represents the fraction of the membrane that remains fully exposed to environmental conditions. For the consortium, the effective exposure is reduced by the covered fraction, leading to the inhibition function in Eq 5: $\gamma_{\text{Z}}(u)=\frac{(\text{1}-ns)u}{a_{\text{X}}+b_{\text{X}}(\text{1}-ns)u}=\frac{u}{a_{\text{Z}}+b_{\text{X}}u}$, where 0<1−ns<1 and $a_{\text{Z}}=\frac{a_{\text{X}}}{\text{1}-ns}>a_{\text{X}}$. The higher value of $a_{\text{Z}}$ means the consortium requires higher bulk concentrations of host metabolite to achieve the same level of growth inhibition as on free-living hosts. The net result is that the consortium experiences lower effective metabolite concentrations at its cell surface compared to a free-living host, even when both are in the same bulk medium. This makes the consortium more tolerant of toxic metabolite accumulation because its ectosymbionts shield it from and actively deplete the local concentration. The mechanism requires no special adaptations beyond already considered host-symbiont physical attachment, syntrophic relationship, and diffusive boundary layer that occurs naturally around all cells in aqueous environments. Our model recognizes that consumers (ectosymbionts) within the boundary layer can provide protective benefits to the mutant hosts beyond bulk medium effects. Though a spatial analysis with concentration gradient and diffusion might improve the model’s scope, we focus on the ecological dynamics of the hosts and symbionts without considering explicit spatial dynamics.

### Surface coverage reduces food consumption

The influx of the metabolites into the mutant host in the consortium is also constrained in proportion to the reduction in the free surface. The uptake of W by the mutant host is thus lowered by the presence of ectosymbionts. Let $p_{\text{Z}}\in (\text{0},\text{1})$ denote the measure of reduction in the consumption rate of W induced per ectosymbiont. The reduced consumption rate of the mutant host with n attached symbionts can be given by:


$$\begin{array}{c} \begin{array}{c} k_{\text{Z}}=\left(\text{1}-np_{\text{Z}}\right)k_{\text{X}}, \end{array} \end{array}$$ (6)

where $\text{1}-np_{\text{Z}}>\text{0}$ to maintain positive values. Larger contact surface aids survival of the mutant host via inhibition reduction but simultaneously corresponds to a lower fecundity via constrained food consumption. Thus, there is a trade-off between inhibition reduction and constrained consumption of the mutant hosts (see Fig 4A). The integration of the effects of occupied surface area on these two traits determines the mutant host’s net benefit, while also compensating for additional subsistence and replication costs compared to the resident host. Note that we distinguish between the impact of surface coverage on inhibition and consumption (using s and $p_{\text{Z}}$, respectively) to include the metabolic-specific effects on the hosts, i.e., s affects inhibition by U, while $p_{\text{Z}}$ affects consumption of W. The independently adjustable treatment of benefit (inhibition reduction) and cost (reduced food influx) is crucial in analyzing the mutant’s fate.

Similarly, ectosymbionts could consume the resource U either from the habitat through their free surface (as U dissolved in the environment of volume Ω, net concentration u/Ω) or directly from its mutant host without any dilution. The total per capita consumption of an ectosymbiont is $k_{\text{Y}}\left(\text{1}-p_{\text{YZ}}\right)u/\Omega +k_{\text{Y}}p_{\text{YZ}}u$, where $p_{\text{YZ}}\in (\text{0},\text{1})$ is the surface impact on the consumption of U by ectosymbionts (subscript YZ correspond to the ectosymbionts, i.e., species Y involved in consortium Z). For the free-living symbionts, entire surface is free, and the expression reduces to $k_{\text{Y}}u/\Omega$. For simplicity, we assume that an ectosymbiont’s consumption of the metabolite directly from its host is much larger than that of the diluted metabolite, i.e., $k_{\text{Y}}p_{\text{YZ}}u\gg k_{\text{Y}}\left(\text{1}-p_{\text{YZ}}\right)u/\Omega$, and we ignore their consumption from the habitat. Denoting $k_{\text{YZ}}$ as the consumption rate of the ectosymbionts, $k_{\text{YZ}}=k_{\text{Y}}p_{\text{YZ}}<k_{\text{Y}}$, i.e., the consumption rate of the ectosymbionts is lower than the consumption of the free-living symbionts. Moreover, the ectosymbiont consumption rate is proportional to the surface area of contact. The remaining metabolite after direct, collective consumption by n ectosymbionts is externalized by the consortium at the rate $k_{\text{Z}}w-nk_{\text{YZ}}k_{\text{Z}}w=\left(\text{1}-nk_{\text{YZ}}\right)k_{\text{Z}}w$ (recall the production of metabolite U by the consortium is proportional to its food uptake, i.e., $k_{\text{Z}}w$). Here, in order to make the release rate positive so that the consortium always releases some amount of U into its external habitat, we need $nk_{\text{YZ}}<\text{1}$. Substituting for $k_{\text{Z}}$ as in Eq 6, we simplify the above equation and define $\left(\text{1}-nk_{\text{YZ}}\right)\left(\text{1}-np_{\text{Z}}\right):=\kappa$, where $\text{1}-nk_{\text{YZ}},\text{1}-np_{\text{Z}}>\text{0}$. The consortium also releases the metabolite V (due to the ectosymbionts) into the habitat at a constant rate $\beta_{\text{Z}}$. The effect of the metabolite V on the ectosymbionts and the consortium is collectively incorporated in the consortium’s reduced growth rate. We assume that the symbiont metabolite does not have any direct self-inhibitory effect on the consortium, i.e., the metabolite-dependent growth inhibition of the consortium is based on the host metabolite only.

### Resident-mutant system: fixation of consortium

Based on the model descriptions, the consortium-forming mutant host has an advantage over the free-living resident in terms of reduced exposure to U, captured using the inhibition factor. However, the mutant phenotype suffers additional costs due to constrained food consumption and higher costs of living and reproduction, which are reflected in their reduced growth rate. Now using the dynamical approach for population dynamics as in the resident system, we analyze whether this consortium-forming host phenotype can invade the syntrophic resident system and replace the free-living phenotype [56]. The mutant consortial host (population density, z) is introduced as an additional dimension to the resident system, and the extended system is as follows:


$$\begin{array}{c} \begin{array}{c} \dot{x}=x\left(r_{\text{X}}-\frac{u}{a_{\text{X}}+b_{\text{X}}u}\right)\;,\\ \dot{y}=y\left(r_{\text{Y}}(u)-\frac{v}{a_{\text{Y}}+b_{\text{Y}}v}\right)\;,\\ \dot{z}=z\left(r_{\text{Z}}-\frac{u}{a_{\text{Z}}+b_{\text{X}}u}\right)\;,\\ \dot{u}=k_{\text{X}}wx+\kappa k_{\text{X}}wz-k_{\text{Y}}\frac{u}{\Omega }y-\delta_{\text{U}}u,\\ \dot{v}=\beta_{\text{Y}}y+\beta_{\text{Z}}z-\delta_{\text{V}}v, \end{array} \end{array}$$ (7)

where all parameters are strictly positive. More details in Appendix A3 in S1 Appendix. Note here that the resident and mutant phenotypes of the host do not coexist as, for positive values of x and z, a unique u that simultaneously equilibrates both x- and z-dynamics does not exist.

### Intraspecies competition replacing self-inhibition

To show that the consortium-forming phenotype cannot invade spontaneously without additional environmental stress in the form of metabolic inhibition, we analyze the resident-mutant dynamics in the absence of self-inhibition. Consider that population growth is now restricted exclusively by intraspecies, density-dependent competition instead of metabolite-dependent self-inhibition. The following alternative system dynamics reflects only the varied costs between the host phenotypes, removing the role of self-inhibition (more details in Appendix A5 in S1 Appendix):


$$\begin{array}{c} \begin{array}{c} \dot{x}=x\left(r_{\text{X}}-x-z\right)\;,\\ \dot{y}=y\left(r_{\text{Y}}(u)-y\right)\;,\\ \dot{z}=z\left(r_{\text{Z}}-x-z\right)\;,\\ \dot{u}=k_{\text{X}}wx+\kappa k_{\text{X}}wz-k_{\text{Y}}\frac{u}{\Omega }y-\delta_{\text{U}}u,\\ \dot{v}=\beta_{\text{Y}}y+\beta_{\text{Z}}z-\delta_{\text{V}}v. \end{array} \end{array}$$ (8)

### Mutant invasion and substitution: a dynamical approach

The model analysis is based on integrating the influence of density-dependent, multi-species ecological dynamics and environment on invasion by a mutant [57,58]. Most of the assumptions of the model are analogous to the adaptive dynamics approach [112–114]; for instance, the timescale separation between ecological dynamics and incidence of rare mutation (we also assume mutant population densities to be rare initially). However, metabolite-dependent population growth and inhibition are effectively modelled using a resource-consumer-based dynamics [38,115–117]. Also, our definition of the specific mutant host phenotype and its invasion, without analyzing evolutionary endpoints, motivated us to use a much flexible dynamical approach [55–58,118]. The resident-mutant dynamics is created by extending the resident ecological dynamics to include mutant population dynamics, where mutant invasion is based on the outcomes of ecological selection. Linear stability analysis [119–122] of the steady states (fixed points) of the resident-mutant dynamics suggests the potential outcomes to which the system converges. *Substitution* here corresponds to the stability of the fixed point where the invading mutant survives while the respective resident perishes; the costly mutant dominates and replaces the resident [56]. The substitution of the free-living resident by the consortium-forming mutant translates to the fixation of the consortium. While analyzing the dynamics of the systems, we are only interested in the stability of the fixed points. Even though the system could have complicated dynamics (for instance, limit cycles), this exceeds the scope of our work as it would require varying resident densities when the mutant is introduced. For simplicity, we prefer to first focus only on the case where the mutant is introduced to a stable resident system with unique fixed points. Assuming the mutant to be rare in density, analysis of the local behavior rather than the global behavior can also be justified.

## Supporting information

S1 Appendix Model analysis. (PDF)

S1 Code Mathematica code for numerical analysis and figure generation. (ZIP)
