# Supplementary material for: Host-initiated microbial association leads to stable ectosymbiosis in an ecological model
Source: PLoS Comput Biol. 2026 Sep 2;22(9):e1014699. doi: 10.1371/journal.pcbi.1014699 (PMC13537694; doi:10.1371/journal.pcbi.1014699)
Supplement: Supplementary file 1 [file pcbi.1014699.s001.pdf]

Supporting Information: Appendix

Host-initiated microbial association leads to stable ectosymbiosis in an ecological model

Nandakishor Krishnan*, István Zachar, Ádám Kun, Chaitanya S. Gokhale, József Garay

*Corresponding author (Email: nandakishor.kris@gmail.com)

Appendix A1. Stability analysis of one-species system

The one-species (host, species X) system dynamics is defined as follows:

$$\dot{x} = x\left(r_{\mathrm{X}} - \frac{u}{a_{\mathrm{X}} + b_{\mathrm{X}}u}\right),$$
$$\dot{u} = \phi x - \delta_{\mathrm{U}} u, \qquad (A1)$$

where $r_{\mathrm{X}} = \frac{k_{\mathrm{X}}w - l_{\mathrm{X}}}{c_{\mathrm{X}}}\ln 2 - d_{\mathrm{X}}$, $\phi = k_{\mathrm{X}}w$, and all parameters are strictly positive. System $A1$ can have two biologically feasible fixed points: trivial $(0, 0)$ and a unique interior fixed point $(x^*, u^*) = \left(\frac{r_{\mathrm{X}}a_{\mathrm{X}}\delta_{\mathrm{U}}}{\phi(1-r_{\mathrm{X}}b_{\mathrm{X}})}, \frac{r_{\mathrm{X}}a_{\mathrm{X}}}{1-r_{\mathrm{X}}b_{\mathrm{X}}}\right)$ that exists if and only if $r_{\mathrm{X}} < \frac{1}{b_{\mathrm{X}}}$. The Jacobian matrix corresponding to the system for arbitrary (x, u) is

$$J_{(x,u)} = \begin{pmatrix} r_{\mathrm{X}} - \frac{u}{a_{\mathrm{X}} + b_{\mathrm{X}}u} & \frac{-xa_{\mathrm{X}}}{(a_{\mathrm{X}} + b_{\mathrm{X}}u)^2} \\ \phi & -\delta_{\mathrm{U}} \end{pmatrix}.$$

$J_{(0,0)}$ has a positive real eigenvalue as $r_{\mathrm{X}} > 0$; hence, $(0, 0)$ is unstable. The real part of both eigenvalues of $J_{(x^*,u^*)}$ is always negative; hence, (x^*, u^*) is always stable, if it exists.

Appendix A2. Stability analysis of two-species system

The two-species (host-symbiont syntrophic pair, species X and Y) system dynamics is defined as follows:

$$\dot{x} = x\left(r_\mathrm{X} - \frac{u}{a_\mathrm{X} + b_\mathrm{X} u}\right),$$
$$\dot{y} = y\left(\eta u - \rho - \frac{v}{a_\mathrm{Y} + b_\mathrm{Y} v}\right), \qquad (A2)$$
$$\dot{u} = \phi x - \xi u y - \delta_\mathrm{U} u,$$
$$\dot{v} = \beta_\mathrm{Y} y - \delta_\mathrm{V} v,$$

where $\eta = \frac{k_\mathrm{Y}}{\Omega c_\mathrm{Y}} \ln 2$, $\rho = \frac{l_\mathrm{Y}}{c_\mathrm{Y}} \ln 2 + d_\mathrm{Y}$, $\xi = \frac{k_\mathrm{Y}}{\Omega}$ (rest of the parameters are as in Appendix A1), and all parameters are strictly positive. System $A2$ can have three biologically feasible fixed points: trivial $(0,0,0,0)$, one-species fixed point $(x^*, 0, u^*, 0)$, and an interior fixed point (x^+, y^+, u^+, v^+) where

$$x^+ = \frac{r_\mathrm{X} a_\mathrm{X}}{\phi(1 - r_\mathrm{X} b_\mathrm{X})}\left(\delta_\mathrm{U} + \frac{\delta_\mathrm{V} a_\mathrm{Y} \xi\left(-a_\mathrm{X} r_\mathrm{X} \eta + \rho(1 - b_\mathrm{X} r_\mathrm{X})\right)}{\beta_\mathrm{Y}\left(-1 + a_\mathrm{X} b_\mathrm{Y} r_\mathrm{X} \eta - b_\mathrm{Y}\rho + b_\mathrm{X}(r_\mathrm{X} + b_\mathrm{Y} r_\mathrm{X} \rho)\right)}\right),$$

$$y^+ = \frac{\delta_\mathrm{V} a_\mathrm{Y}\left(-a_\mathrm{X} r_\mathrm{X} \eta + \rho(1 - b_\mathrm{X} r_\mathrm{X})\right)}{\beta_\mathrm{Y}\left(-1 + a_\mathrm{X} b_\mathrm{Y} r_\mathrm{X} \eta - b_\mathrm{Y}\rho + b_\mathrm{X}(r_\mathrm{X} + b_\mathrm{Y} r_\mathrm{X} \rho)\right)},$$

$$u^+ = \frac{r_\mathrm{X} a_\mathrm{X}}{1 - r_\mathrm{X} b_\mathrm{X}},$$

$$v^+ = \frac{a_\mathrm{Y}\left(-a_\mathrm{X} r_\mathrm{X} \eta + \rho(1 - b_\mathrm{X} r_\mathrm{X})\right)}{\left(-1 + a_\mathrm{X} b_\mathrm{Y} r_\mathrm{X} \eta - b_\mathrm{Y}\rho + b_\mathrm{X}(r_\mathrm{X} + b_\mathrm{Y} r_\mathrm{X} \rho)\right)}.$$

The fixed point (x^+, y^+, u^+, v^+) exists if and only if $\frac{\rho}{\eta} < \frac{r_\mathrm{X} a_\mathrm{X}}{1 - r_\mathrm{X} b_\mathrm{X}} < \frac{\rho}{\eta} + \frac{1}{\eta b_\mathrm{Y}}$ and $r_\mathrm{X} < \frac{1}{b_\mathrm{X}}$. The Jacobian matrix corresponding to the system for arbitrary (x, y, u, v) is

$$J_{(x,y,u,v)} = \begin{pmatrix} r_\mathrm{X} - \frac{u}{a_\mathrm{X} + b_\mathrm{X} u} & 0 & \frac{-x a_\mathrm{X}}{(a_\mathrm{X} + b_\mathrm{X} u)^2} & 0 \\ 0 & \eta u - \rho - \frac{v}{a_\mathrm{Y} + b_\mathrm{Y} v} & \eta y & \frac{-y a_\mathrm{Y}}{(a_\mathrm{Y} + b_\mathrm{Y} v)^2} \\ \phi & -\xi u & -\xi y - \delta_\mathrm{U} & 0 \\ 0 & \beta_\mathrm{Y} & 0 & -\delta_\mathrm{V} \end{pmatrix}.$$

$J_{(0,0,0,0)}$ and $J_{(x^*,0,u^*,0)}$ have a positive real eigenvalue as $r_\mathrm{X} > 0$ and $\eta \frac{r_\mathrm{X} a_\mathrm{X}}{1 - r_\mathrm{X} b_\mathrm{X}} > \rho$, respectively; hence, corresponding fixed points are unstable. The Jacobian matrix corresponding to the system at (x^+, y^+, u^+, v^+) is

$$J_{(x^+,y^+,u^+,v^+)} = \begin{pmatrix} 0 & 0 & \dfrac{-x^+ a_X}{(a_X + b_X u^+)^2} & 0 \\ 0 & 0 & \eta y^+ & \dfrac{-y^+ a_Y}{(a_Y + b_Y v^+)^2} \\ \phi & -\xi u^+ & -\xi y^+ - \delta_U & 0 \\ 0 & \beta_Y & 0 & -\delta_V \end{pmatrix} := \begin{pmatrix} 0 & 0 & -j_{13} & 0 \\ 0 & 0 & j_{23} & -j_{24} \\ j_{31} & -j_{32} & -j_{33} & 0 \\ 0 & j_{42} & 0 & -j_{44} \end{pmatrix},$$

where, in the redefined matrix elements of $J_{(x^+,y^+,u^+,v^+)}$, $j_{13}, j_{23}, j_{24}, j_{31}, j_{32}, j_{33}, j_{42}$, and j_{44} are all strictly positive. Now, the characteristic equation of $J_{(x^+,y^+,u^+,v^+)}$, for λ denoting eigenvalues, can be expressed in the form

$$\lambda^4 + A_1\lambda^3 + A_2\lambda^2 + A_3\lambda + A_4 = 0,$$

where $A_1 = j_{33} + j_{44}$, $A_2 = j_{13}j_{31} + j_{23}j_{32} + j_{24}j_{42} + j_{33}j_{44}$, $A_3 = j_{24}j_{33}j_{42} + j_{13}j_{31}j_{44} + j_{23}j_{32}j_{44}$, and $A_4 = j_{13}j_{24}j_{31}j_{42}$. Here, as $A_1 > 0$, $A_2 > 0$, $A_3 > 0$, $A_4 > 0$, $A_1A_2 > A_3$, and $A_1A_2A_3 > A_1^2A_4 + A_3^2$, using the Routh-Hurwitz criterion, real part of all eigenvalues of $J_{(x^+,y^+,u^+,v^+)}$ is negative; hence, (x^+, y^+, u^+, v^+) is always stable, given it exists. For details on Routh-Hurwitz criterion and linear stability analysis concepts, see Edelstein-Keshet (2005), Gantmacher (1960), Khalil (2002), and Perko (2001) in the reference list of main text.

Appendix A3. Stability analysis of resident-mutant system

The resident-mutant system dynamics is defined as follows:

$$\begin{gathered} \dot{x} = x\left(r_X - \frac{u}{a_X + b_X u}\right), \\ \dot{y} = y\left(\eta u - \rho - \frac{v}{a_Y + b_Y v}\right), \\ \dot{z} = z\left(r_Z - \frac{u}{a_Z + b_X u}\right), \\ \dot{u} = \phi x + \kappa\phi z - \xi u y - \delta_U u, \\ \dot{v} = \beta_Y y + \beta_Z z - \delta_V v, \end{gathered} \qquad (A3)$$

where $r_Z = \frac{k_Z w - l_Z}{c_Z}\ln 2 - d_Z$, $k_Z = k_X(1 - np_Z)$, $a_Z = \frac{a_X}{1-ns}$, $\kappa = (1 - np_Z)(1 - nk_{YZ})$ (rest of the parameters are as in Appendix A2), and all parameters are strictly positive. System $A3$ can have five feasible fixed points: $E_0 = (0, 0, 0, 0, 0)$, $E_1 = (x^*, 0, 0, u^*, 0)$, $E_2 = (x^+, y^+, 0, u^+, v^+)$,

$E_\mathrm{P} = (0, \hat{y}, \hat{z}, \hat{u}, \hat{v})$, and $E_\mathrm{M} = (0, 0, \bar{z}, \bar{u}, \bar{v})$. The Jacobian matrix corresponding to the system for arbitrary (x, y, z, u, v) is

$$J_{(x,y,z,u,v)} = \begin{pmatrix} r_\mathrm{X} - \dfrac{u}{a_\mathrm{X} + b_\mathrm{X} u} & 0 & 0 & \dfrac{-x a_\mathrm{X}}{(a_\mathrm{X} + b_\mathrm{X} u)^2} & 0 \\ 0 & \eta u - \rho - \dfrac{v}{a_\mathrm{Y} + b_\mathrm{Y} v} & 0 & \eta y & \dfrac{-y a_\mathrm{Y}}{(a_\mathrm{Y} + b_\mathrm{Y} v)^2} \\ 0 & 0 & r_\mathrm{Z} - \dfrac{u}{a_\mathrm{Z} + b_\mathrm{X} u} & \dfrac{-z a_\mathrm{Z}}{(a_\mathrm{Z} + b_\mathrm{X} u)^2} & 0 \\ \phi & -\xi u & \kappa\phi & -\xi y - \delta_\mathrm{U} & 0 \\ 0 & \beta_\mathrm{Y} & \beta_\mathrm{Z} & 0 & -\delta_\mathrm{V} \end{pmatrix}.$$

E_0 and E_1 are always unstable. The resident fixed point $E_2 = (x^+, y^+, 0, u^+, v^+)$ is stable if and only if $r_\mathrm{Z} - \frac{u^+}{a_\mathrm{Z} + b_\mathrm{X} u^+} < 0$, i.e., $\frac{r_\mathrm{X} a_\mathrm{X}}{1 - r_\mathrm{X} b_\mathrm{X}} > \frac{r_\mathrm{Z} a_\mathrm{Z}}{1 - r_\mathrm{Z} b_\mathrm{X}}$.

The mutant-only fixed point $E_\mathrm{M} = (0, 0, \bar{z}, \bar{u}, \bar{v}) = \left(0, 0, \frac{r_\mathrm{Z} a_\mathrm{Z} \delta_\mathrm{U}}{\kappa\phi(1 - r_\mathrm{Z} b_\mathrm{X})}, \frac{r_\mathrm{Z} a_\mathrm{Z}}{1 - r_\mathrm{Z} b_\mathrm{X}}, \frac{r_\mathrm{Z} a_\mathrm{Z} \delta_\mathrm{U} \beta_\mathrm{Z}}{\delta_\mathrm{V} \kappa\phi(1 - r_\mathrm{Z} b_\mathrm{X})}\right)$ exists if and only if $r_\mathrm{Z} < \frac{1}{b_\mathrm{X}}$ and is stable if and only if:

1. $r_\mathrm{X} - \frac{\bar{u}}{a_\mathrm{X} + b_\mathrm{X} \bar{u}} < 0$, i.e., $\frac{r_\mathrm{X} a_\mathrm{X}}{1 - r_\mathrm{X} b_\mathrm{X}} < \frac{r_\mathrm{Z} a_\mathrm{Z}}{1 - r_\mathrm{Z} b_\mathrm{X}}$ and
2. $\eta\bar{u} - \rho - \frac{\bar{v}}{a_\mathrm{Y} + b_\mathrm{Y} \bar{v}} < 0$, i.e., $a_\mathrm{Z} r_\mathrm{Z} \left(\frac{\eta}{1 - r_\mathrm{Z} b_\mathrm{X}} - \frac{\beta_\mathrm{Z} \delta_\mathrm{U}}{a_\mathrm{Z} b_\mathrm{Y} r_\mathrm{Z} \beta_\mathrm{Z} \delta_\mathrm{U} + a_\mathrm{Y} \delta_\mathrm{V} \kappa\phi(1 - b_\mathrm{X} r_\mathrm{Z})}\right) < \rho$.

Assuming existence of E_2 and E_M, if condition 1 is not satisfied, the resident fixed point E_2 is stable; if only condition 2 is not satisfied, then $E_\mathrm{P} = (0, \hat{y}, \hat{z}, \hat{u}, \hat{v})$ can also exist and be stable. If E_M is stable, E_P does not exist; if E_P exists, $\frac{r_\mathrm{X} a_\mathrm{X}}{1 - r_\mathrm{X} b_\mathrm{X}} < \frac{r_\mathrm{Z} a_\mathrm{Z}}{1 - r_\mathrm{Z} b_\mathrm{X}}$ is necessary for its stability. Mutant invasion, i.e., stability of E_M or E_P happens only if $\frac{r_\mathrm{X} a_\mathrm{X}}{1 - r_\mathrm{X} b_\mathrm{X}} < \frac{r_\mathrm{Z} a_\mathrm{Z}}{1 - r_\mathrm{Z} b_\mathrm{X}}$, which can only be achieved by high values of a_Z relative to a_X. High values of ns satisfies the condition as $a_\mathrm{Z} = \frac{a_\mathrm{X}}{1 - ns}$. Additionally, restructuring condition 2 suggests that exclusive stability of E_M, i.e., extinction of free-living individuals, happens at high values of β_Z and low values of κ (high k_YZ and n lowers κ). For more details and numerical local stability investigations of all fixed points (example Jacobian matrices and eigenvalues), see Mathematica notebook provided as Supporting Information S1 Code.

Numerical examples

In Tables A1–A3 below, we provide numerical examples verifying the stability of each of the fixed points corresponding to arbitrary parameter values as in Table 1 and Fig 3 of main text (also see S1 Code). A fixed point is stable if all eigenvalues of the Jacobian matrix of system $A3$ evaluated at the point have negative real part.

Table A1. Numerical validation of stability of E_2. $s = 0.2$, $\beta_Z = 0.2$, $k_{YZ} = 0.05$, and rest of the parameter values are as in Table 1 of main text.

Fixed point	Numerical fixed point	Real part of eigenvalues of Jacobian matrix	Stability
$E_0(0,0,0,0,0)$	$(0,0,0,0,0)$	$56.51, 24.73, -2.87, -1, -1$	Unstable
$E_1(x^*,0,0,u^*,0)$	$(0.52,0,0,130,0)$	$-19.08, -0.5, -0.5, -1, 0.13$	Unstable
$E_M(0,0,\bar{z},\bar{u},\bar{v})$	$(0,0,0.406,55,0.081)$	$21.13, -0.5, -0.5, -1.66, -1$	Unstable
$E_2(x^+,y^+,0,u^+,v^+)$	$(0.52,0.097,0,130,0.195)$	$-19.08, -0.5, -0.5, -0.85, -0.15$	Stable

Table A2. Numerical validation of stability of E_P. $s = 0.4$, $\beta_Z = 0.2$, $k_{YZ} = 0.05$, and rest of the parameter values are as in Table 1 of main text.

Fixed point	Numerical fixed point	Real part of eigenvalues of Jacobian matrix	Stability
$E_0(0,0,0,0,0)$	$(0,0,0,0,0)$	$56.51, 24.73, -2.87, -1, -1$	Unstable
$E_1(x^*,0,0,u^*,0)$	$(0.52,0,0,130,0)$	$-0.5, -0.5, 4.1, -1, 0.13$	Unstable
$E_2(x^+,y^+,0,u^+,v^+)$	$(0.52,0.097,0,130,0.195)$	$-0.5, -0.5, 4.1, -0.85, -0.15$	Unstable
$E_M(0,0,\bar{z},\bar{u},\bar{v})$	$(0,0,1.217,164,0.243)$	$-5.65, -0.5, -0.5, -1, 0.76$	Unstable
$E_P(0,\hat{y},\hat{z},\hat{u},\hat{v})$	$(0,0.579,1.224,164,1.403)$	$-5.65, -0.5, -0.5, -0.5, -0.5$	Stable

Table A3. Numerical validation of stability of E_M. $s = 0.4$, $\beta_Z = 4$, $k_{YZ} = 0.06$, and rest of the parameter values are as in Table 1 of main text.

Fixed point	Numerical fixed point	Real part of eigenvalues of Jacobian matrix	Stability
$E_0(0,0,0,0,0)$	$(0,0,0,0,0)$	$56.51, 24.73, -2.87, -1, -1$	Unstable
$E_1(x^*,0,0,u^*,0)$	$(0.52,0,0,130,0)$	$-0.5, -0.5, 4.1, -1, 0.13$	Unstable
$E_2(x^+,y^+,0,u^+,v^+)$	$(0.52,0.097,0,130,0.195)$	$-0.5, -0.5, 4.1, -0.85, -0.15$	Unstable
$E_M(0,0,\bar{z},\bar{u},\bar{v})$	$(0,0,1.244,164,4.977)$	$-5.65, -0.5, -0.5, -2.24, -1$	Stable

Appendix A4. Numerical analyses

In Table 1 of main text, note that we used arbitrary numerical values (satisfying the model conditions and also in connection with Krishnan et al. (2024) in the reference list of main text). This is due to limitations in explicit information on metabolic costs and benefits of modern prokaryotic consortia and its arguable reliability in analyzing our hypothesis of early integration. However, we emphasize that our results hold regardless of the parameter values and whether the abstract, non-empty parameter regions are broad or not. In this section, we describe our results based on parameter sensitivity analyses of our model using the abstract numerical values. For more details of numerical investigations, see S1 Code.

A4.1. Metabolite dilution

Based on the condition for free-living population exclusion, high values of the habitat volume parameter (Ω) can make the consortium-only fixed point E_{M} exclusively stable. High values of host metabolite dilution (large habitat volume) guarantee the extinction of free-living symbionts (Fig A1), owing to their disadvantageous resource accessibility and competitive exclusion. Note that this host metabolite dilution factor does not affect the mutant invasion condition.

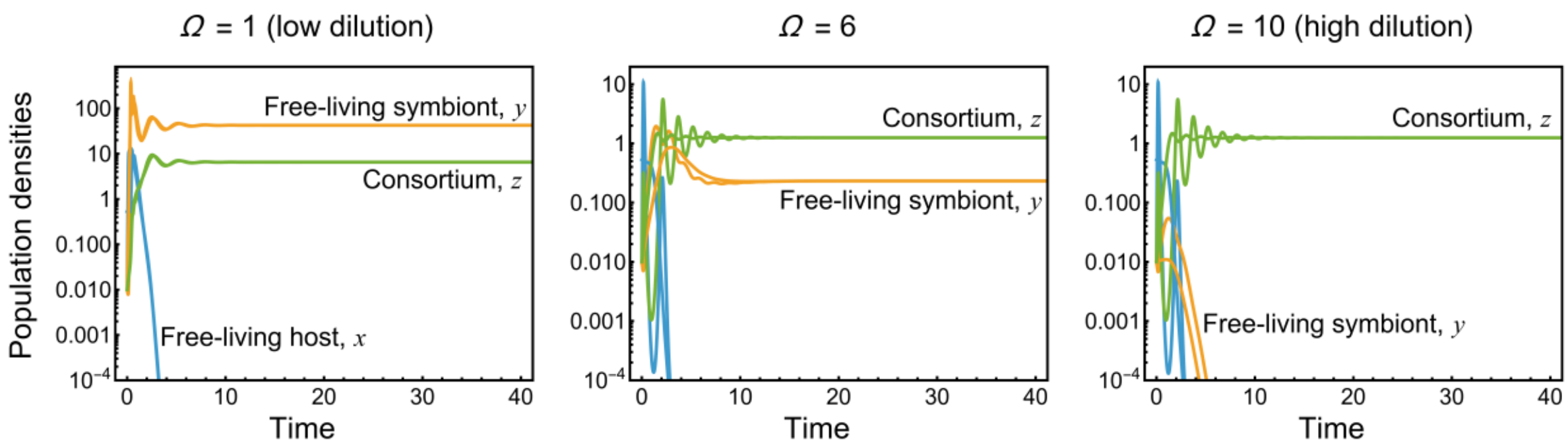

Fig A1. Impact of metabolite dilution. High dilution of host metabolite (large habitat volume) facilitates population-level exclusion of free-living symbionts. At low available concentrations of host metabolite due to dilution, the free-living symbionts are at a disadvantage in terms of resource utilization compared to the ectosymbionts having direct metabolite access. Parameter values are as in Table 1 in the main text, and initial conditions are arbitrary points close to E_0 and E_1.

A4.2. Ectosymbiont dynamics

To indirectly model the dynamics of ectosymbionts in the consortium, we consider the possibility of unsynchronized host-ectosymbiont replication. The ectosymbionts now replicate faster than their host, leading to a positive release or escape of free-living symbionts into the external habitat. Assuming the addition to the free-living symbiont pool is at a constant rate (denoted τ) and linearly dependent on the consortial density gives the following dynamics:

$$\begin{gathered}
\dot{x} = x\left(r_{\mathrm{X}} - \frac{u}{a_{\mathrm{X}} + b_{\mathrm{X}}u}\right), \\
\dot{y} = y\left(\eta u - \rho - \frac{v}{a_{\mathrm{Y}} + b_{\mathrm{Y}}v}\right) + \tau z, \\
\dot{z} = z\left(r_{\mathrm{Z}} - \frac{u}{a_{\mathrm{Z}} + b_{\mathrm{X}}u}\right), \\
\dot{u} = \phi x + \kappa\phi z - \xi u y - \delta_{\mathrm{U}}u, \\
\dot{v} = \beta_{\mathrm{Y}}y + \beta_{\mathrm{Z}}z - \delta_{\mathrm{V}}v,
\end{gathered} \tag{A4}$$

where $\tau > 0$ and rest of the parameters are as in the original model in system $A3$. Numerical results of system $A4$ suggest that higher values of τ promote the stability of E_{P} and hence survival of the free-living symbionts (Fig A2).

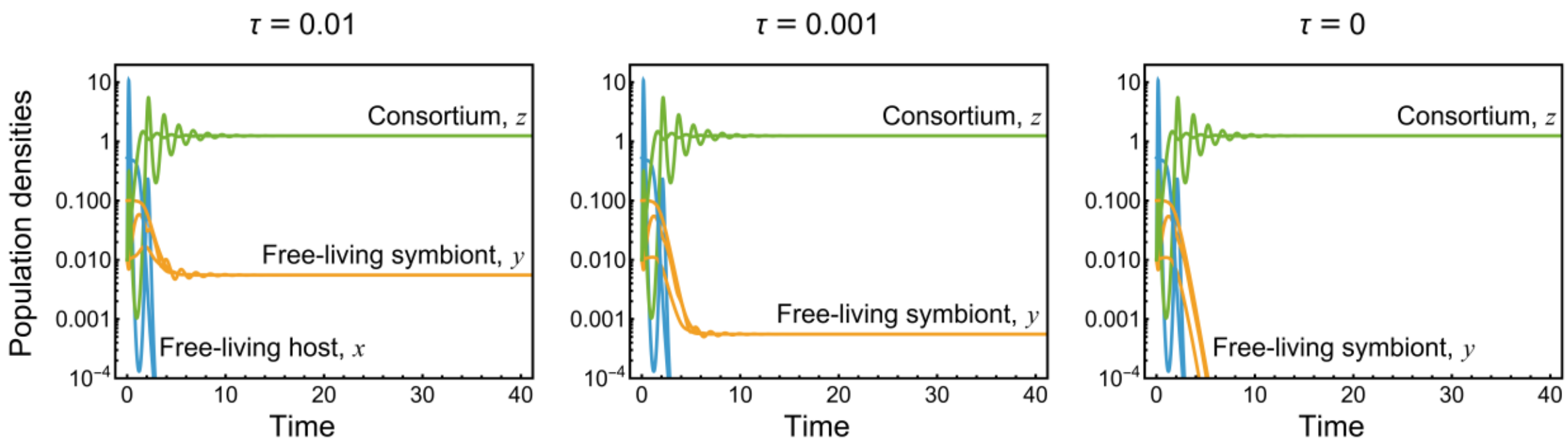

Fig A2. Impact of ectosymbiont dynamics. Upon introducing unsynchronized host-symbiont replication, as the escape or release factor (τ) approaches zero, free-living symbiont density declines and the system approaches population-level obligate ectosymbiosis. Parameter values are as defined in Table 1 in the main text, and initial conditions are arbitrary points close to E_0, E_1, and E_2.

A4.3. Vertical inheritance of ectosymbionts

Consider the possibility that the offspring of an ectosymbiotic, consortial host (Z) may not be necessarily ectosymbiotic, i.e., vertical inheritance of symbionts between hosts is not perfect. The offspring of the hosts fail to attach (or obtain) symbionts on their surface and hence exist

in free-living state, thus relaxing our original assumption that all mutant hosts exist only in the consortial form. If $\theta \in (0,1)$ denotes the effectiveness or reliability of vertical inheritance of ectosymbionts and $\theta = 1$ corresponds to perfect reliability, we can rewrite system $A3$ as:

$$\begin{gathered} \dot{x} = x\left(r_{\mathrm{X}} - \frac{u}{a_{\mathrm{X}} + b_{\mathrm{X}}u}\right) + (1-\theta) r_Z z, \\ \dot{y} = y\left(\eta u - \rho - \frac{v}{a_{\mathrm{Y}} + b_{\mathrm{Y}}v}\right), \\ \dot{z} = z\left(\theta r_Z - \frac{u}{a_{\mathrm{Z}} + b_{\mathrm{X}}u}\right), \\ \dot{u} = \phi x + \kappa \phi z - \xi u y - \delta_{\mathrm{U}} u, \\ \dot{v} = \beta_{\mathrm{Y}} y + \beta_{\mathrm{Z}} z - \delta_{\mathrm{V}} v. \end{gathered} \quad (A5)$$

Numerical analysis of the system $A5$ shows that if vertical inheritance is sufficiently accurate (if parameter value is above a critical value, $\theta > \theta_{\mathrm{critical}}$), consortial association can be stably maintained (Fig A3). For a similar result in a different model of ectosymbiosis, see Krishnan et al. (2024) in the reference list of main text.

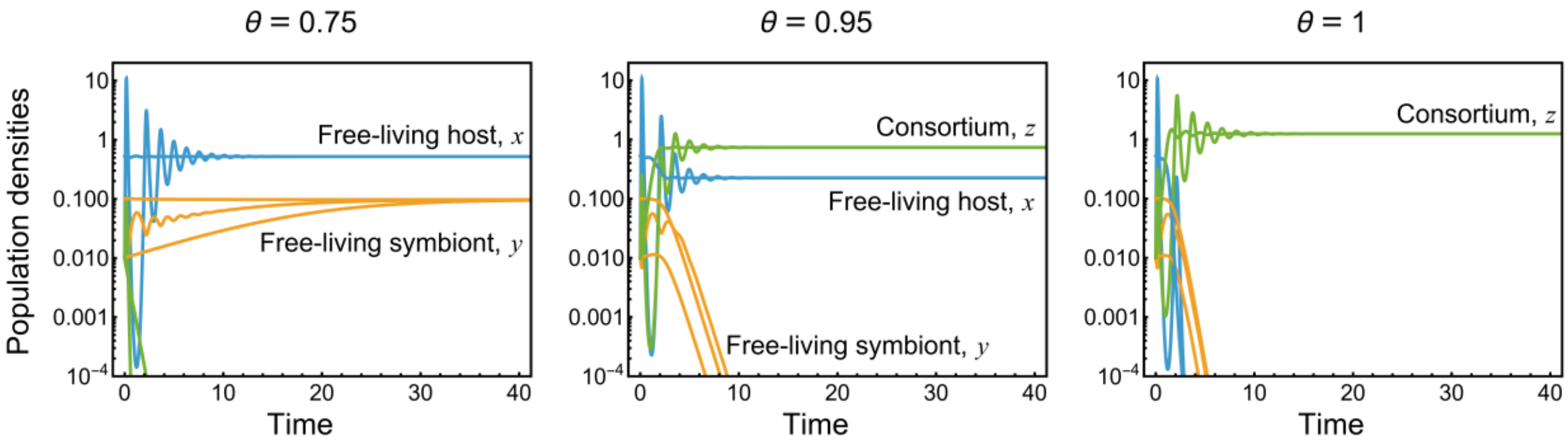

Fig A3. Impact of vertical inheritance. For $\theta < \theta_{\mathrm{critical}}$, the mutant cannot invade and the resident fixed point E_2 is stable. As θ increases above the critical value ($\theta_{\mathrm{critical}} \approx 0.8$), consortial density increases. Simultaneously, free-living host and symbiont densities decline with both extinct at $\theta = 1$, i.e., only perfect vertical inheritance gives exclusion of both free-living types. Parameter values are as defined in Table 1 in the main text; initial conditions are arbitrary points close to E_0, E_1, and E_2.

A4.4. Non-uniform metabolic effects

There could be mechanisms other than the geometric protective shielding from the ectosymbionts that could alter (and minimize) the effective self-inhibitory effect on the host; for instance, a mutant host with an enzyme that could internally neutralize self-inhibition. Any such mechanism would modify the inhibition parameters separately, i.e., b_{Z} would no longer

be identical to b_X (see Eq 5 of main text). A mutation resulting in such varied metabolic effect across the host phenotypes is presumably independent of that resulting in physical attachment (which we have modelled). Thus, varying both the inhibition parameters would biologically mean (or require) that we consider two mutations: attachment-inducing and metabolism-altering. This gives four different combinations or phenotypes and requires a completely new ecological model for the analysis.

Alternatively, we could define a single mutation that could alter both inhibition parameters together; for instance, the mutant having membrane structures that could simultaneously initiate ectosymbiosis and somehow reduce self-inhibition even without ectosymbionts. This assumption corresponds to b_X in z-dynamics of system $A3$ being replaced by b_Z, and the mutant invasion condition also changes accordingly. For such a hypothetical scenario, Fig A4 shows the sensitivity of the mutant invasion barrier to varying b_Z (i.e., $b_Z \neq b_X$) and the mathematical result is independent of the symmetry across the phenotypes in terms of metabolic effect.

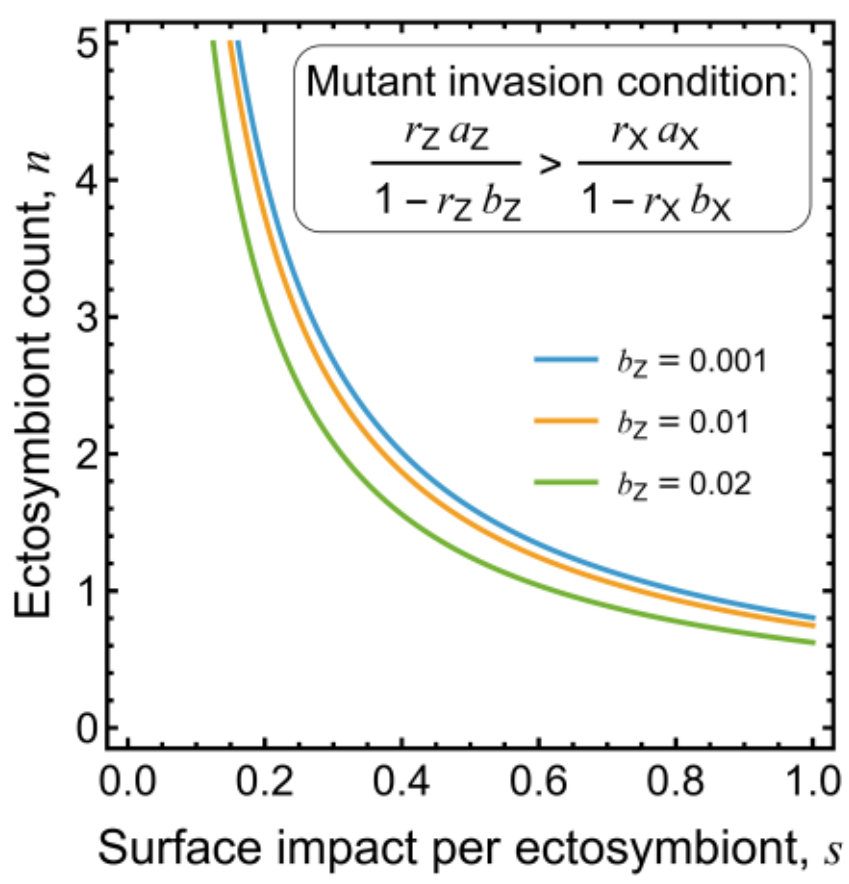

Fig A4. Impact of non-uniform metabolic effects. Shift in invasion boundary for non-uniform metabolic effects for the resident and mutant hosts ($b_Z \neq b_X$) is plotted. The invasion barrier shifts without qualitatively (mathematically) altering the existence of the invasion regime. Parameters are as in Table 1 of main text.

Note that this mechanistic variation, with a single mutation altering both parameters, is hard to justify realistically and is entirely outside the scope of our original model assumptions. We have consistently refrained ourselves from considering b_Z and b_X separately throughout our

model since the corresponding well-defined analysis demands an entirely new system with more dimensions to account for the multiple mutant phenotypes as noted before. In case the alternative mechanism is effective enough to completely eliminate any self-inhibition, then the benefit of ectosymbiosis disappears, making physical integration unlikely to emerge. Thus, altering the metabolic effects is not reasonable in understanding how ectosymbiosis could emerge and does not add much to our hypothesis.

Appendix A5. Stability in competitive resident-mutant system

An alternative resident-mutant system dynamics without self-inhibition is defined as follows:

$$\begin{gathered}\dot{x} = x(r_{\mathrm{X}} - x - z),\\ \dot{y} = y(\eta u - \rho - y),\\ \dot{z} = z(r_{\mathrm{Z}} - x - z),\\ \dot{u} = \phi x + \kappa\phi z - \xi u y - \delta_{\mathrm{U}} u,\\ \dot{v} = \beta_{\mathrm{Y}} y + \beta_{\mathrm{Z}} z - \delta_{\mathrm{V}} v.\end{gathered} \tag{A6}$$

System $A6$ can have five feasible fixed points (similar to the original self-inhibiting system $A3$). The Jacobian matrix corresponding to the system for arbitrary (x, y, z, u, v) is

$$J_{(x,y,z,u,v)} = \begin{pmatrix} r_{\mathrm{X}} - 2x - z & 0 & -x & 0 & 0 \\ 0 & \eta u - \rho - 2y & 0 & \eta y & 0 \\ -z & 0 & r_{\mathrm{Z}} - x - 2z & 0 & 0 \\ \phi & -\xi u & \kappa\phi & -\xi y - \delta_{\mathrm{U}} & 0 \\ 0 & \beta_{\mathrm{Y}} & \beta_{\mathrm{Z}} & 0 & -\delta_{\mathrm{V}} \end{pmatrix}.$$

Assuming the existence of all fixed points, the local stability analysis shows that the resident-only fixed point is stable always, and all other fixed points are unstable (note $r_{\mathrm{X}} > r_{\mathrm{Z}}$). The mutant host cannot invade this system. More details on the fixed points and the Jacobian matrices are in S1 Code.
