# Supplementary material for: Host-initiated microbial association leads to stable ectosymbiosis in an ecological model
Source: PLoS Comput Biol. 2026 Sep 2;22(9):e1014699. doi: 10.1371/journal.pcbi.1014699 (PMC13537694; doi:10.1371/journal.pcbi.1014699)
Supplement: Supplementary file 2 [file pcbi.1014699.s003.pdf]

Response to reviewers

Host-initiated microbial association leads to stable ectosymbiosis in an ecological model

Nandakishor Krishnan, István Zachar, Ádám Kun, Chaitanya S. Gokhale, József Garay

The authors are extremely grateful for the detailed and thoughtful reviews from the two reviewers. In response to the queries and comments, we have added new results and clarification statements in the revised manuscript, and the model presentation is also improved. The supplementary files are also revised accordingly. Below we provide pointwise responses to all the review comments, with indication of where we have made the necessary clarifications and/or added new results within the revised manuscript. Author responses are italicized and colored blue. We have also made other minor edits in the manuscript to improve clarity and readability. We believe the revision has improved the manuscript altogether, and we look forward to any further comments.

Reviewer #1

Major comments

1. Core mechanism and parameterization

The central claim is a geometric mechanism of reduced inhibition at the contact surface. This requires explicit biophysical grounding and separation from simpler consumption-only relief.

a) Provide a mechanism for reduced exposure at the membrane. If the effect is due to diffusion boundary layers or receptor saturation, state this and indicate how it maps to the equations.

Response: *A new subsection is added to the Materials and methods (Lines 637–658), describing the biophysical mechanism (based on diffusion boundary layers) of inhibition reduction.*

b) Do not reuse bX by default. Introduce bZ and report sensitivity to bZ != bX. Conclusions should not hinge on keeping the inhibition shape parameter identical across phenotypes.

Response: *The resident and mutant phenotypes differ only in their intrinsic death rate and costs of living and reproduction, but not in their metabolic utilization, production, or self-inhibition. Since the host phenotypes produce the same metabolite and their inhibitory effect on both are also identical, the inhibition parameters (a_X and b_X) do not*

vary innately. Note that a_{Z} is not a trait variation of a_{X}. Parameter a_{Z} varies because of the geometric benefit due to attached symbionts such that the inhibition term for the mutant is $\gamma_{\mathrm{Z}}(u) = \frac{(1-ns)u}{a_{\mathrm{X}}+b_{\mathrm{X}}(1-ns)u} = \frac{u}{a_{\mathrm{Z}}+b_{\mathrm{X}}u}$, where $a_{\mathrm{Z}} = \frac{a_{\mathrm{X}}}{1-ns} > a_{\mathrm{X}}$ and that of the resident is $\gamma_{\mathrm{X}}(u) = \frac{u}{a_{\mathrm{X}}+b_{\mathrm{X}}u}$. This is to maintain that the difference in the effective inhibition between the host phenotypes is purely in terms of the geometric mechanism and not by assumption. Conversely, assuming that b_{X} could vary ($b_{\mathrm{Z}} \neq b_{\mathrm{X}}$) would require that mutation altered the metabolite's impact on the two host phenotypes, which is inexplicable as we do not assume either the metabolite, its production pathway, or its effect on host changes. Moreover, it would also make the dependence of inhibition on s and n difficult to follow. We have clarified these in Lines 630–636.

c) Test alternative shielding functions with mechanistic rationale. The current aZ = aX/(1 - n*s) assumes linear scaling of inhibition relief with unoccupied surface. Inhibition is plausibly a saturating function of exposed surface, for example because of boundary-layer limits to diffusion or receptor-site saturation. Please test aZ = aX/(1 - (n*s)^alpha) with alpha != 1 and report how invasion regions shift. This is not merely an arbitrary sensitivity test; it evaluates whether the conclusion depends on the assumed linear geometry versus a more biophysically plausible saturation.

Response: *We have analyzed this by checking inhibition function $a_{\mathrm{Z}} = \frac{a_{\mathrm{X}}}{1-(ns)^{\alpha}}$ for $\alpha = 0.3$ and $\alpha = 2$. For value of $\alpha > 1$, the invasion region shrinks, while $\alpha < 1$ makes it broader (quantitatively). The results do not change with inhibition factor's varied dependence on the occupied surface (ns). Our results are independent of the assumed linear geometry for inhibition (see Fig 4C).*

d) Add a consumption-only control: hold aZ = aX (no geometric benefit) and increase local consumption terms (for example kYZ or βZ) to match the net benefit provided by surface shielding. If invasion persists, the mechanism is syntrophy-driven rather than geometry-driven; if invasion fails, the geometric hypothesis is necessary and should then be supported with empirical examples or plausible physics.

Response: *Mutant invasion (stability of either E_{M} or E_{P}) depends only on the growth rates (r_{X} and r_{Z}) and the inhibition parameters (b_{X}, a_{X}, and a_{Z}), i.e., invasion is purely geometry-driven. However, the conditions differentiating the stability between E_{M} and E_{P} are determined by the syntrophy parameters, implying the population-level obligacy in ectosymbiosis (stable E_{M}) is syntrophy-driven. If $a_{\mathrm{Z}} = a_{\mathrm{X}}$, invasion does not happen regardless of how other parameters are changed, i.e., without the benefit mutants cannot invade, as expected. Increasing k_{YZ} or β_{Z}, while the invasion condition is already met, stabilizes only E_{M} rather than E_{P}. These support our conclusion that geometric difference in benefit is key to invasion, while syntrophy drives population-level obligacy (Fig 3). Biophysical explanation for the geometric benefit of inhibition reduction is included in the revised manuscript (Lines 637–658).*

e) Clarify the sign logic by introducing a simple benefit factor if helpful (for example, bfac = 1 - n*s or 1 - (n*s)^alpha) and mapping it to the inhibition term.

Response: *We consider that ns represents the total surface area of the host occupied by the symbionts. Without loss of generality, we scaled the total finite surface area of the hosts to unity, and assuming unoccupied surface is always non-zero (no full*

coverage) leads to the inequality $1 - ns > 0$ (see Lines 622–625). The objective was to highlight more on how ns affect the dynamics via the inhibition parameter rather than presenting it in terms of an additional benefit parameter.

f) Justify allowing s (benefit-per-symbiont) and sZ (cost-per-symbiont) to differ. As a geometric baseline, test sZ = s; if they remain independent, provide a biological rationale (for example, distinct membrane domains).

Response: *Note that we have now relabeled s_{Z} to p_{Z} within the manuscript for clarity. We consider that the surface coverage by symbionts affects the host's consumption and inhibition reduction in different ways, controlled by two different parameters (p_{Z} and s). This is because the metabolite consumed by the host and that is responsible for its growth inhibition are different: W and U, respectively. By separating p_{Z} and s, we include the reasonable assumption that the host responds differently to the two metabolites. Contrarily, if these effects on the host are not assumed to be metabolite-specific, it would indeed be natural to consider $p_{\mathrm{Z}} = s$. Moreover, $p_{\mathrm{Z}} = s$ does not give invasion, i.e., using a single parameter to reflect impact on the two features is not feasible in our model. The two opposing effects of surface coverage on reducing consumption (cost) and increasing inhibition reduction (benefit) cancel out, never satisfying the mutant invasion condition. By keeping p_{Z} and s separate, we could distinctly quantify the positive and negative impacts that allow invasion. Using p_{Z}, we focused on keeping the mutant's growth rate effectively a constant, independent of s, and always significantly lower than the residents. We have now redefined the parameters to make the metabolite-specific effects clear (Table 1 and Lines 672–674).*

2. Ectosymbiont dynamics and inheritance: address the "fixed n" and passive vertical inheritance simplification

The model assumes a constant mean number of ectosymbionts per host (n) with "poor man's passive vertical inheritance," explicitly omitting symbiont birth-death-attachment-detachment dynamics and stochastic partitioning at division. Because reliable association and transmission are central to stabilizing ectosymbiosis, please assess robustness to variance in n (e.g., binomial partitioning) and discuss how explicit attachment/detachment or symbiont life cycles might affect invasion/stability. A minimal sensitivity analysis in SI would materially strengthen the argument.

Response: *We agree that the effect of explicit symbiont dynamics and variable symbiont numbers must be important factors in ectosymbiotic establishment that should be explored. In this paper, we deliberately ignored the explicit number of ectosymbionts-per-host focusing rather on the more general factor of the total occupied surface (i.e., ns). An average n was fixed so that variable s reflects impact of ns on the dynamics. A binomial treatment with focus on the exact number, spatial arrangement, and attachment-detachment dynamics of the ectosymbionts require extensive re-modelling and is beyond the scope of this manuscript. However, a numerical analysis featuring unsynchronized host-symbiont cell-cycle (an indirect approach to model ectosymbiont dynamics) is now added (Fig A2 of Appendix A4 in S1 Appendix). According to our results, unsynchronized replication enables the survival of free-living symbionts rather than their ecological exclusion (Lines 387–398 in main text). Please also see response to Reviewer #2 Q3.*

3. Mixed equilibrium (EP), coexistence, and bistability

Provide explicit existence and local stability criteria for EP, or numerical Jacobians with eigenvalues. Justify the statement that no equilibrium with coexisting resident and mutant hosts exists. If an analytic argument is infeasible, provide systematic numerical evidence. Clarify whether EP can be bistable with E2 or EM; if history dependence occurs, the interpretation of outcomes should be adjusted.

Response: *Explicit existence and stability conditions of fixed-point E_{P} are difficult to produce analytically. However, indeed numerical evidence of Jacobians and eigenvalues for stability of all feasible fixed points are included in S2 Source Code. Coexistence of resident and mutant phenotypes can be excluded algebraically as x- and z-dynamics cannot be simultaneously equilibrated for the same positive value of u (considering non-zero x and z). E_{P} cannot be bistable with E_2 or E_{M}; the two inequalities that differentiate the stability of each fixed point are mentioned at the end of Appendix A3 in S1 Appendix.*

4. Robustness and parameter sensitivity

Go beyond hand-picked parameter sets. Provide two-parameter maps quantifying the regions that yield E2, EP, and EM (for example, s vs n or m; k_YZ vs beta_Z; key cost and death parameters). Include sensitivity to the dilution factor Omega used for metabolite U. If invasion holds only when Omega is large but fails at Omega = 1, report this and interpret the biology. If viable regions are narrow, temper generality claims.

Response: *All the parameter regions relevant to the parameter choices and results are now included either in the manuscript or S2 Source Code (e.g., s vs n, a_{Z} vs r_{Z}, k_{YZ} vs β_{Z}, and κ vs β_{Z}). Sensitivity of the dynamics to the dilution factor is also newly added now (Lines 300–303 and Fig A1 of Appendix A4 in S1 Appendix). The dilution factor does not affect invasion; however, high dilution or large habitat volume aids the survival of ectosymbionts (due to their advantage of direct resource access) and exclusion of free-living ones (i.e., stable E_{M} against E_{P}).*

5. Adaptive Dynamics (AD), or temper evolutionary language

If the manuscript continues to make evolutionary claims, analyze selection on a relevant trait (for example, s, n, or m). In SI, include a one-trait PIP, classify singular points (convergence stability, ESS vs branching), and examine sensitivity to trade-off curvature (concave vs convex shielding and cost). If AD is not added, remove teleological phrasing and present results in ecological terms, for example, "the mutant can invade when ...", "the system converges to EM under conditions ...", without asserting evolutionary endpoints.

Response: *Thanks for the comment. Indeed, we focus on the substitution mechanism and do not aim at making evolutionary arguments. We have added a clarification statement (Lines 338–341) and toned down our evolutionary claims wherever applicable.*

6. Frequency dependence, finite symbiont pool, and public-good logic

In the current model, free-living symbionts function as a public good: any host benefits from their consumption of the inhibitory metabolite. When mutant hosts attach symbionts, they privatize a shared benefit, indirectly harming residents. This tragedy-of-the-commons

mechanism appears central to invasion yet is not articulated. Explicitly articulate this public-good privatization logic and at minimum discuss outcomes under a finite symbiont supply. Preferably add a simple extension with finite supply or attachment-rate dynamics (numerical simulations are acceptable if analytical treatment is intractable) to test necessity: does invasion rely on privatizing a limited partner pool, or does it occur even when symbiont availability is effectively unlimited?

Response: *We assume that the "free-living symbiont pool is much larger than the host population" and there is no restriction in terms of availability of the symbionts in the habitat. Also, note that the mutant host surface is finite and can only accommodate a limited number of symbionts. The mutant hosts attaching (or privatizing) the symbionts would only make negligible difference to the benefit the resident hosts receive from the free-living symbionts. Privatization does not harm other hosts or reduce their benefits significantly as the symbiont availability is still abundant in the habitat, hence the situation does not classify as tragedy-of-the-commons. However, we have numerically analyzed an alternative situation of a finite symbiont supply, and the outcomes are qualitatively similar (Lines 399–408).*

7. Scope, definitions, and claims

Define carefully:

* obligate metabolic dependence (cannot grow without the partner metabolite)
* obligate physical association (cannot reproduce without attachment)
* population-level obligate ectosymbiosis (free-living types extinct in the population)
* genotype-level obligate symbiosis (loss of autonomy at the genotype level, typically requiring genetic loss of autonomous functions over evolutionary time).

As formulated, EM reflects population-level ecological exclusion rather than genotype-level obligacy. Claims linking to eukaryogenesis should be tempered; without internalization, regulated inheritance, or genome reduction, the model is best positioned as a general theory for conditions favoring attachment in self-inhibiting systems. Include a null model with self-inhibition removed (aX and aY very large) to show that ectosymbiosis does not invade when only costs are present.

Response: *Thanks a lot for highlighting the terms requiring clarification. Usage of "obligate" is now clarified wherever applicable within the manuscript. We have added new text to the Discussion as well (Lines 421–438).*

The model indeed presents conditions favoring ectosymbiosis based on an already syntrophic interaction. Syntrophic theories on the emergence of eukaryogenetic endosymbiosis often assume that metabolic coupling and prolonged coexistence of the partners pre-date internalization, vertical inheritance, and genome reduction. We presented a model of ectosymbiosis, where the partners are already engaged in metabolic coupling and a primitive attachment mechanism appears to form an ecologically stable coexistence. Furthermore, genotype-level coevolutionary adaptations can be achieved by the group-selection of the host-symbiont consortium, which may eventually lead to internalization, vertical inheritance, and genome reduction.

An alternative model without self-inhibition is now included (Lines 713–721, 255–259 of main text, and Appendix A5 in S1 Appendix). Very large a_{X} *and* a_{Y} *would make the*

dynamics unbounded (X and Y grow exponentially); instead, we have used intraspecific competition to replace self-inhibition and test the hypothesis. The ectosymbiotic phenotype could not invade in the new system.

Minor points

1. Model exposition and notation

State explicitly that rX and rZ are not direct functions of the inhibitor; inhibition acts via u/(a + b*u) multiplying growth. Introduce kappa = (1 - n*k_YZ)*(1 - n*s_Z) early and provide a brief derivation or indicate whether it is an approximation and under what conditions it holds. If the consortium inhibition term reuses bX by assumption, say so plainly; otherwise define and analyze bZ.

Response: *Thanks for the suggestions. All recommended changes have been made (Lines 562, 687–693, and 630–636).*

2. Dilution and consistency

Explain the role of Omega and test sensitivity. Clarify the asymmetry in how Omega scales the symbiont's access to U but not the host's access to its resource, and test sensitivity to Omega across biologically plausible ranges.

Response: *Role of parameter Omega (Ω) is now explicitly defined (Lines 568–575), and sensitivity results are included (Lines 300–303 of main text and Fig A1 of Appendix A4 in S1 Appendix).*

The concentration of the host's resource is assumed to be constant and scaling it does not make any qualitative difference to the model results. Note that we assumed dilution does not affect host's food availability, owing to a continuously replenishing food source. We have explicitly indicated the dilution only for metabolite U as its concentration is a variable.

3. Figures and diagnostics

For time series, reduce overplotting or add nullclines/vector fields to clarify approach to equilibria. For region plots, add labels or contours indicating invasion and stability margins. Provide at least one EP example with numerical eigenvalues if EP remains a featured outcome.

Response: *Figures are now restructured (Fig 2 and 3), and solutions for different initial conditions are overlaid on the same time series subplots. Due to the high dimensionality of the extended system, phase portraits are not viable. Stability boundaries of parameter regions are indicated in region plots (Fig 4). Numerical example and analysis of the E_{P} fixed point are included in the S2 Source Code.*

4. Parameter ranges and empirical diagnostics

Where possible, bound key parameters by orders of magnitude from microbial systems (uptake rates, decay, inhibition parameters) or justify the explored ranges. Where possible,

bound key parameters using the empirical systems already referenced in the manuscript (for example, acetate inhibition in E. coli, alcohol toxicity in yeast, Chlorochromatium consortia). Even approximate values from these examples would help constrain the relevant parameter space.

Response: *Our original results are purely analytical and are valid regardless of the numerical values used. The arbitrary values for the parameters (Table 1) were only for the purpose of visualizing and verifying the results. Our results hold for an even wider range of parameter values without requiring any qualitative changes, given they comply with the assumptions of the model. All conditions for analytical results and their combinations limiting the parameter ranges are included in the S1 Appendix or S2 Source Code. The exact parameter values are irrelevant to the model as long as those are within the feasible bounds created by the conditions.*

5. Reproducibility

Extend SI code with scripts for parameter sweeps and, if added, AD PIPs. Document seeds and initial-condition ranges used to generate figures.

Response: *All data and codes used to produce the figures and verify the results are included in the manuscript (Table 1) and S2 Source Code.*

6. Editorial clarity

Use "consortium" (singular) and "consortia" (plural) consistently throughout. Replace ambiguous phrases such as "reducing the inhibition of accumulating products" with parameter-linked wording. State explicitly whether "consortium" and "mutant host" are treated interchangeably in equations and figures.

Response: *All recommended changes have been made.*

Reviewer #2

1. Model presentation and notation

The Results section frequently refers to variables, parameters, and equation-based statements without first presenting the model in a compact mathematical form. The full equations appear only in Methods and Appendices. In addition, the text names for the same entities are not always used consistently. These issues make the Results section hard to follow. I suggest introducing the final form of the model equations at the beginning of the Results and, at that point, giving each entity a standard full descriptive name together with its symbol. In the subsequent text, the authors can use text name + symbol, with the text name optionally shortened as long as it remains a clear subset of the original full name. Establishing this naming convention early and using it consistently would greatly improve readability. It would also be helpful to label the variables directly in Fig. 1 so that readers can easily connect the notation used in the text with the components shown in the diagram.

Response: *Thanks for the suggestions. We have made changes to the Results section to improve readability. All equation-based statements are now confined to Materials and methods or S1 Appendix. All notations are now labelled in Fig 1.*

2. Inconsistency between consumption and growth terms (Eq. 4)

In Eq. (4), the net growth rate r_Y includes a ln2 factor arising from the assumption that each cell consumes resource at constant rate throughout its cell cycle. However, the population-level consumption term, -k_Y u/Ω y, implies that per-capita consumption rate increases continuously as the population grows from 1 to 1.99 cells before division. Thus, the flux of resource consumption is measured in two incompatible ways in the growth equation and in the substrate equation. To maintain mass-balance consistency, the authors should either: include the same ln2 factor in the population-level consumption term (i.e., replace -k_Y u/Ω y with -ln2 k_Y u/Ω y), or remove the ln2 factor from r_Y (and possibly also from r_X and r_Z for consistency).

Response: *Our phrase "uniformly continuous resource consumption" might have been misleading here. The consumption is uniform, continuous, and at a constant rate k_{Y} throughout the symbiont's lifespan (clarified now at Lines 527, 532). The per-capita consumption of the symbiont is thus $k_{\mathrm{Y}}u/\Omega$, which indeed depends on the available metabolite concentration. The flux of resources in both growth and substrate equations depends linearly on the metabolite (growth rate $r_{\mathrm{Y}}(u) = \frac{k_{\mathrm{Y}}\frac{u}{\Omega} - l_{\mathrm{Y}}}{c_{\mathrm{Y}}} \ln 2 - d_{\mathrm{Y}}$). However, the $\ln 2$ factor in the growth rate is due to how the linearly consumed biomass is effectively translated to species growth based on a branching process. Rather than assuming that the population growth is solely based on the consumed biomass, we also account for parts of the consumed resource spent as costs of subsistence and replication. All the considered life history traits put together results in the varied growth rate with $\ln 2$ scaling. This is based on our previously published metabolite-dependent Malthusian growth formulation (Krishnan et al., 2024). Identical explanation for r_{X} and r_{Z}. Clarified now at Lines 534–536.*

3. Treatment of ectosymbiont dynamics in the mutant-only regime

The Discussion (lines 393–409) argues that the dynamics of attached ectosymbionts can be ignored because a large external symbiont pool effectively buffers them. This is reasonable when free-living symbionts are present, but it does not apply to the mutant-only equilibrium where no free-living symbionts remain. In the mutant-only case, the attached symbionts are the only symbiont population, so neglecting their dynamics can lead to behavior that appears biologically implausible. For example, the model allows free-living symbionts to be driven extinct by metabolite V produced by ectosymbionts, yet the same level of V does not appear to limit the ectosymbionts on the host. The authors may therefore need to either exclude the mutant-only equilibrium from the biological interpretation or refine the treatment of ectosymbiont dynamics near y→0.

Response: *The ectosymbionts receive a benefit in terms of inhibition reduction and are relatively more tolerant to metabolite V compared to the free-living ones. The free-living and ectosymbiotic symbionts differ in terms of their local environment, reduced inhibition, access to food, and effective growth rates. In our model where ectosymbiont dynamics is completely ignored, we assumed a mechanism such that the ectosymbionts' replication appears to be in synchronization with their host (though this assumption is far-fetched, this is a necessity for model tractability). However, we have now included (in Appendix A4 in S1 Appendix) a numerical analysis of a system with a faster replication rate for the symbionts, pertaining to their relatively smaller size. The symbiont offspring could either occupy additional receptors on the growing host or be released into the habitat at a constant rate (linear term $\tau \cdot z(t)$ added to y-dynamics). At positive values of τ (e.g., $\tau = 0.01$ and $\tau = 0.001$), free-living symbionts can survive. As τ increases, free-living symbiont density increases. Only $\tau = 0$ gives the existence and stability of the consortium-only equilibrium. With this new result, we indirectly model the ectosymbiont dynamics and show that perfect host-symbiont cell-cycle synchronization is a necessity for the consortium-only outcome in our model setting. We have discussed these results in the Discussion (Lines 387–398).*

4. Line 648–650: unclear inequality

The inequality nk_YZ<1 is stated without derivation. Based on the surrounding text, it is not clear how this condition arises, and it may be that a relationship involving u and ψ, such as nk_YZ u

Response: *Note that ψ is now relabelled as w.* We assume that n *ectosymbionts can collectively consume nk_{YZ} portion of the total metabolite produced by their host, where k_{YZ} is the consumption rate per ectosymbiont and metabolic produce of the consortial host is proportional to their own total consumption, i.e., $k_Z w$. Only the remaining portion $k_Z w - nk_{YZ}k_Z w$ is released into the habitat. The inequality $1 - n\mathrm{k}_{YZ} > 0$ assumes that the release rate is positive so that the consortial host always releases some amount of the metabolite into the external environment. Clarified in Lines 687–693.*

5. Ambiguity in the definitions of u and Ω

Table 1 defines u(t) as the "Concentration of host metabolic product U in the habitat" and Ω as the "Volume of the habitat or medium (reflects dilution)". However, this makes the meaning of u/Ω unclear. In different parts of the text, u and u/Ω appear to play different

roles (e.g., concentration near the host vs. far from the host, or intracellular vs. extracellular levels). The authors should clarify the physical interpretation of u, Ω, and u/Ω, and make the intended spatial or compartmental structure explicit.

Response: *Variable u is the total concentration of host metabolite U in the external environment, Ω is the total volume of the habitat, and u/Ω is the net concentration of host metabolite available to the free-living symbionts in the habitat for consumption. u/Ω represents dilution of the metabolite concentration due to its distribution over the whole habitat volume. The objective here was to introduce a parameter to indirectly distinguish the two modes of symbiont consumption: 1) direct consumption when symbionts are in host's contact (ectosymbiotic) and 2) consumption of the diluted metabolite by free-living symbionts. The dilution factor puts the free-living symbionts at a disadvantage in terms of resource accessibility. Clarifications are made wherever applicable in the manuscript (Lines 568–575).*
