# Supplementary material for: Host-initiated microbial association leads to stable ectosymbiosis in an ecological model
Source: PLoS Comput Biol. 2026 Sep 2;22(9):e1014699. doi: 10.1371/journal.pcbi.1014699 (PMC13537694; doi:10.1371/journal.pcbi.1014699)
Supplement: Supplementary file 3 [file pcbi.1014699.s004.pdf]

Response to reviewers (second revision)

Host-initiated microbial association leads to stable ectosymbiosis in an ecological model

Nandakishor Krishnan, István Zachar, Ádám Kun, Chaitanya S. Gokhale, József Garay

We are again grateful to the Reviewer for having put so much effort and thought into understanding our paper and into the review process to make it more understandable to the readers. We are also pleased that the Reviewer agrees that we have addressed previous concerns in detail and that the manuscript has improved greatly. With this latest round, we address the remaining concerns as best as we can, without digressing much from our original scope. Our responses below (italicized and blue) answer each point raised by the Reviewer; line numbers in the responses correspond to that in the latest manuscript version.

Reviewer #1

1. Core mechanism and parameterization

This point is improved, but not fully resolved. The added diffusion-based rationale for reduced inhibition at the host-symbiont contact surface is helpful, and the sensitivity test using a nonlinear shielding function is also a useful addition. The consumption-only control likewise helps clarify the structure of the model, because it makes clearer that invasion depends on the inhibition-related terms, whereas syntrophy affects whether the long-term outcome is facultative or consortium-only.

That said, one of my more specific requests was not actually carried out. I had asked for a sensitivity analysis in which b_Z is allowed to differ from b_X. Instead, the authors argue that b_Z should equal b_X by construction. That is a legitimate modeling decision, but it is not the same as demonstrating robustness to relaxing the assumption. In a phenomenological model, it would still have been informative to test whether the qualitative result persists when that symmetry is broken. I also think the revised explanation of why p_Z and s are separated, while clearer than before, makes it more evident that invasion depends on treating the cost and benefit sides of surface occupation as independently tunable. That dependence should be stated more directly in the manuscript.

Response: *In our model, the resident and mutant hosts vary only in terms of the presence or absence of an attachment-related feature and remain identical from any other point of view, metabolism included. Accordingly, we consider only a single mutation that results in attachment. The attachment consequently can affect only the metabolite flux, but not the internal metabolic effect of it. For* b_{Z} *to differ from* b_{X}*, a change in the internal processing of the metabolite must also be assumed (i.e., a change in metabolism), which is*

presumably unrelated to and thus independent of the effect of coupling with ectosymbiotic partners. Thus, $b_{\mathrm{Z}} \neq b_{\mathrm{X}}$ does not make any sense in our model unless we redefine our mutation or consider a new type of mutant (besides the one with attachment capability) with varied metabolic-specific effects. We have presented a hypothesis on how ectosymbiosis could emerge via physical interaction, hence we have deliberately focused on the conditions for stable ectosymbiotic attachment. An independent trait variation in terms of metabolism takes the focus away from our hypothesis and requires a different model description (and paper) altogether. (Also, if the host can by itself better neutralize its own inhibitive product, there is trivially no incentive for an ectosymbiotic partnership to emerge.) The Reviewer admits that ours is a legitimate modelling approach, and we strongly believe that the question should define the model and the model should define the analysis, not the other way around.

Nevertheless, in Appendix A4.4 in S1 Appendix, we have now discussed in detail the $b_{\mathrm{Z}} \neq b_{\mathrm{X}}$ scenario (explaining that this is a digression from our original question and mechanism) and carried out a mathematically driven sensitivity analysis (though not based on our original hypothesis) assuming varied b_{Z} corresponding to non-uniform metabolic effect across the two host types. Without being explicit on the biological reasoning behind breaking the symmetry of $b_{\mathrm{Z}} = b_{\mathrm{X}}$, we showed that the qualitative mathematical results persist regardless of whether or not the equality is assumed. We have clarified these in Lines 666–677 with reference to S1 Appendix.

Independent treatment of cost and benefit using p_{Z} and s is also now made explicit (Lines 713–717).

2. Ectosymbiont dynamics and inheritance

This concern remains only partly addressed, and in my view it is still important. The new extension with unsynchronized host-symbiont replication is valuable because it shows that the consortium-only outcome is much less robust once perfect synchronization is relaxed. The authors now acknowledge that consortium dominance in the present framework effectively depends on very low escape or release of ectosymbionts. That is an important clarification.

However, the broader issue I raised was not limited to synchronization alone. The manuscript still does not address variance in n, stochastic partitioning during host division, or explicit attachment-detachment dynamics. Those are not marginal details. They go directly to the question of whether the association can be stably transmitted and maintained. The revised manuscript is more open about this limitation, but the limitation itself remains substantial.

Response: *We agree with the Reviewer that these are important details of stable long-term transmission. Binomial treatment of ectosymbiont distribution with host division could, in theory, handle the exact n number of symbionts (or variance in n), their random partitioning during division, and attachment-detachment dynamics. However, such treatment is not a mere extension of our analytical model as it would constitute another, discrete, and (at least semi-) individual-based model built on stochastic and individual-based principles. Though these are important aspects to consider, we definitely think that such a model is beyond the scope of this manuscript, worth exploring in itself in another*

paper (which we are working on). In this paper, we have deliberately refrained ourselves from presenting conceptually distinct models that warrant distinct discussions.

Yet, to meet the rightful request to model a less perfect vertical inheritance, in addition to the relaxation of synchronization, we have now conducted a new numerical analysis (Fig A3 and Appendix A4.3 in S1 Appendix). Here, we consider the possibility of imperfect vertical inheritance (using parameter θ to denote its effectiveness) of symbionts from consortial hosts to their offspring. We observe that below a critical value $\theta < \theta_{\mathrm{critical}}$, the mutant cannot invade. For $\theta > \theta_{\mathrm{critical}}$, consortium is stable; and as θ increases, the free-living host population declines and if vertical inheritance is perfect ($\theta = 1$), it completely perishes. This new result proves that the consortial association can be stably maintained only if vertical inheritance is sufficiently accurate (explained now in Lines 412–422). As we have argued above, a more in-depth analysis of the transition from low to high accuracy of vertical inheritance would require a stochastic model built on entirely different principles.

3. Mixed equilibrium E_P, coexistence, and bistability

This point is much better than before. The authors now state more clearly that coexistence of resident and mutant host types can be excluded algebraically, and they indicate that E_P is not bistable with E_2 or E_M. They also provide numerical Jacobians and eigenvalue calculations in the supplementary computational material. That addresses much of the substance of my concern.

My remaining reservation is mainly one of presentation. The revised SI now makes the analytical stability results clearer for E_2 and E_M, but the local stability analysis of E_P is still deferred mainly to the code supplement rather than presented directly in the SI narrative.

Response: *Presentation of information on existence and stability of E_{P} is now improved. All fixed points are discussed explicitly in Appendix A3 in S1 Appendix, and their numerical analyses are included in S2 Code.*

4. Robustness and parameter sensitivity

This part is substantially improved. The added parameter-region plots are useful, and the clarification of the role of Omega is responsive to my earlier concern. In particular, it is now clearer that Omega does not affect invasion itself, but does affect whether the long-term ecological outcome is E_P or E_M. That is a helpful distinction.

At the technical level, I regard this issue as largely addressed. What remains weaker is the biological interpretation of those regions. The manuscript still does not say enough about whether the viable parts of parameter space are broad in any biologically constrained sense, as opposed to broad only within the internal logic of the abstract model.

Response: *To our knowledge, explicit data on metabolic costs and benefits of modern prokaryotic consortia (including measuring these in vivo) are limited. Moreover, it is also arguable whether any such data could be reliably used to assume anything about the early steps of integration. We emphasize that selection does not necessarily require a broad feasible parameter region to work with, any region that is biologically viable (even if narrow) may be enough for evolution to work. The fact that endosymbiosis between mitochondria and its host evolved only once suggests that either it was so successful that*

any further attempt was outcompeted or the required ecological and biochemical window for it is extremely narrow. If the parameter space turned out to be biologically narrow, that would explain the rarity of observed prokaryotic integrations. However, given the speculative nature of these considerations, we omitted such discussions in the manuscript and believe that the abstract, non-empty parameter region is sufficient for selection to start with. We have now added a statement at the beginning of Appendix A4 (numerical analyses) in S1 Appendix.

5. Evolutionary framing

This remains one of the main unresolved issues. In their response, the authors state that they do not aim to make evolutionary arguments and that they have toned down the corresponding language. However, the revised manuscript still repeatedly uses strongly evolutionary wording. It continues to state, in various places, that obligate ectosymbiosis can evolve, that consortial cooperation is selected for, that an evolutionary path exists, and that the model represents a plausible first step toward endosymbiotic integration. The manuscript also still frames the work in eco-evolutionary terms, even though the later clarification makes clear that evolutionary endpoints are not actually modeled.

This mismatch remains serious. An ecological mutant-invasion analysis can certainly motivate evolutionary hypotheses, but it does not, by itself, justify claims about evolutionary trajectories or long-term transitions such as endosymbiotic integration. If the authors do not wish to add an explicit evolutionary treatment, then the manuscript needs a much more consistent ecological framing throughout, especially in the abstract, author summary, and discussion.

Response: *Evolution happens when a heritable trait undergoes change in its frequency in a population over time. The fact that, in our model, a mutant with attachment abilities could invade and persist against a free-living resident population indisputably proves that the invasion can serve as the first step of a series of evolutionary changes. We focused on this particular invasion; and though we did not explicitly demonstrate evolutionarily stable endpoints based on resistance to all and any mutant invasions, our results still support evolutionary claims. Subsequent successful invasions of mutants, each better than the previous in terms of integration, are possible based on their selective advantage and incontrovertibly support our evolutionary hypothesis.*

Based on the incentive that stabilizes the mutant (from the mutant invasion condition), we predict that subsequent mutants with traits enabling improved integration are likely to be selected for. Ectosymbiosis acts as the first step, and self-inhibition as the ecological driver determines the direction of subsequent evolution (toward enhanced physical integration), where the destination or endpoint is based on evolutionary stability. In contrast to a single-trait optimization, we followed a different framework by Cressman et al. with trait invasion modelled step-by-step using multiple ecological dynamical systems, where step 1 was stabilization of syntrophy and step 2 was stabilization of ectosymbiosis. In this progressive setup, unlike in adaptive dynamics, outcomes of ecological selection (rather than globally optimal trait) aid the analysis of the underlying evolutionary process.

However, our model could predict subsequent mutant invasion (e.g., an invaginating host) only if the model circumstances remain consistent (e.g., resources, mechanisms, ecological conditions, etc.). If the circumstances vary (new environment, new mutation

type, etc.), predicting the outcome becomes impossible without separately considering these factors (which would require new models and papers). We do not demonstrate or make claims on the exact steps involved in the later stages of transition or the evolutionarily stable endpoints, but only discuss rational pathways and directions based on the suggestions from the multiple ecological analyses. We have now added more clarification (Lines 348–355) and carefully reworded some text in Abstract, Author summary, and Discussion.

6. Finite symbiont pool and public-good logic

This point is partly addressed. The authors now explain more clearly that the free-living symbiont pool is treated as effectively abundant, and they no longer lean as heavily on a tragedy-of-the-commons interpretation. They also state that a finite-supply extension gives qualitatively similar results. That clarification is useful.

Even so, I still think the manuscript could explain the benefit-allocation logic more clearly. Even under an abundant-pool assumption, the key conceptual issue is how attachment redistributes local benefit between resident and mutant host types, and how that redistribution contributes to invasion. That remains central to the interpretation of the model and deserves a sharper explanation in the paper itself.

Response: *Thank you for pointing out this aspect that needs better clarity. Attachment of symbionts on the surface of the mutants does not redistribute the benefit (from ectosymbiotic shielding or free-living symbiont availability in the pool) to any other host individual (either resident or mutant). This also does not affect mutant invasion as the growth rate and inhibition of a mutant individual are not modified by inhibition (or its reduction) to any other hosts. Self-inhibition to an individual is localized (i.e., a private benefit) and depends only on external metabolite concentration. Thus, except for the contribution of hosts to the metabolite bulk, they do not interfere with each other's benefits. We have now clarified these in Lines 431–436.*

7. Scope, definitions, and strength of claims

This is improved, but not fully corrected. The authors have made some constructive changes here. In particular, the distinction between different meanings of "obligate" is clearer than before, and the null model without self-inhibition is an important addition because it shows that consortium formation does not invade in the absence of the proposed ecological driver.

Still, the manuscript continues to move too quickly from ecological exclusion outcomes to broader claims about long-term symbiotic integration, vertical inheritance, higher-level individuality, and eukaryogenesis. Those ideas may well motivate the study, but they are not demonstrated by the present model. I therefore still think the claims need to be narrowed. The paper would be stronger if it stayed closer to what it actually shows: the ecological conditions under which a costly consortium-forming mutant can invade and persist when hosts suffer self-inhibition and attachment reduces that inhibition locally.

Response: *In our paper, we do not demonstrate the whole process of eukaryogenesis, i.e., syntrophy and attachment eventually followed by later transitions involving internalization, vertical inheritance, and genome reduction. We put our results into perspective by discussing them as potential pathways that could further multi-species*

microbial integration in an environment with metabolic inhibition (also explained in response to Q5). Eukaryogenesis indeed acts as our motivation for modelling, given it is known to be an early and stable example of such integration. Our model and conjectures are inspired by hypotheses by López-García, Moreira, Martin, Ettema, and others, primarily the ones where stable metabolic ectosymbiosis is considered to precede mitochondrial integration and eukaryogenesis (especially the syntrophy, hydrogen, and reverse-flow hypotheses). Please also see, e.g., Imachi et al., 2020 and Nobs et al., 2026 (in the main text references) for experimentally driven hypotheses on early archaea-bacteria integration. We mathematically showed that obligate ectosymbiosis could emerge from free-living, metabolically coupled partners, demonstrating one of the initial steps in the evolutionary transition. We believe that providing a broader context to our results helps mathematically less-interested readers to better understand our results. We have now clarified these considerations in Lines 464–473.

8. Minor issues

Several of the minor issues are now handled satisfactorily. The notation is clearer, the role of kappa is more explicit, the treatment of Omega is easier to follow, and the figures appear improved. Reproducibility is also better supported through the supplementary computational material.

My main remaining minor concern is that the local stability analysis of E_P is still largely deferred to the code supplement rather than presented directly in the SI narrative. The revised appendix now provides explicit analytical stability statements for E_2 and E_M, which improves the presentation substantially, but for E_P it still states that the stability conditions are difficult to obtain analytically and refers the reader to S2 for the numerical local stability analysis. A second remaining issue is empirical parameterization. The authors respond that the main conclusions are analytical and therefore do not depend on the exact numerical values used in the illustrations. That is reasonable as far as internal model logic is concerned, but it does not fully answer the interpretive point. A brief discussion of whether the explored parameter regions are at least broadly plausible for microbial systems would still strengthen the manuscript.

Response: *All minor concerns are answered in our above responses.*
