# Supplementary material for: Host-initiated microbial association leads to stable ectosymbiosis in an ecological model
Source: PLoS Comput Biol. 2026 Sep 2;22(9):e1014699. doi: 10.1371/journal.pcbi.1014699 (PMC13537694; doi:10.1371/journal.pcbi.1014699)
Supplement: Supplementary file 4 [file pcbi.1014699.s005.pdf]

Response to reviewers (third revision)

Host-initiated microbial association leads to stable ectosymbiosis in an ecological model

Nandakishor Krishnan, István Zachar, Ádám Kun, Chaitanya S. Gokhale, József Garay

We are pleased that the Reviewer is convinced by our previous responses and revisions. We have now made all the minor revisions suggested by the Reviewer. We are thankful to the Reviewers and the Editor for allowing us to improve our manuscript. We hope the Editor now consider our manuscript acceptable for publication.

Journal requirements

We noticed that you used the phrase 'data not shown' in the manuscript. We do not allow these references, as the PLOS data access policy requires that all data be either published with the manuscript or made available in a publicly accessible database. Please amend the supplementary material to include the referenced data or remove the references.

Authors' response: *We could not find any such phrase in the manuscript or the supplementary material. We do not refer to any non-cited data anywhere in the manuscript. Data Availability Statement is revised for clarification.*

Reviewer #1

1. Claim calibration.

The manuscript still uses "prove/proves" (Author Summary l. 56, Results l. 188, Discussion l. 349) and forward-looking statements such as "evolutionary path ... toward obligate endosymbiosis" and "could have eventually led to the endosymbiotic origin of mitochondria" (l. 61). A feasibility model shows possibility under assumptions, not proof, and does not analyze a mutation sequence, trait substitution, or evolutionary stability. Replace "prove" with "show", "demonstrate within the model", or "identify conditions under which", and phrase the trajectory and eukaryogenesis statements as hypotheses. The ecological-selection framing ("ecologically selected for", invasion as a first step) is acceptable; only the unhedged claims need softening, consistently in the Abstract, Author Summary, Results, and Discussion.

Authors' response: *Text rewritten at all places as suggested.*

2. Dependence on ectosymbiont retention should be visible in the main text.

Two separate sensitivity analyses bear on this. In the imperfect-inheritance model (Appendix A4.3), consortial persistence requires theta > theta_critical (approx 0.8 for the illustrative parameters), and both free-living types go extinct only at perfect inheritance (theta = 1); in the ectosymbiont-release model (Appendix A4.2), the consortium-only outcome E_M is likewise recovered only in the limit of zero escape (tau = 0), with small positive tau approaching it while retaining a low free-living symbiont density. This dependence is central, not a side caveat, and

sits uneasily with the Abstract's claim that population-level obligate ectosymbiosis emerges "under a wide range of conditions". Add a sentence in the Results near E_M stating it, and calibrate the Abstract to distinguish invasion and facultative ectosymbiosis, which hold over a range, from the obligate outcome, which requires these ideal-retention limits.

Authors' response: *Abstract revised and a statement added to Results.*

3. Scope of "obligate".

Keep E_M described as population-level ecological exclusion throughout. The statement that free-living extinction "effectively ensures" that symbionts cannot replicate without the host (l. 459-461) holds only within the ecological closure of the model; it is not genotype-level obligacy or a mechanistic loss of autonomy, and should be stated as such.

Authors' response: *Statement is now clarified.*

4. E_P stability presentation.

The E_P Jacobian and eigenvalues are computed in S2 Code, but the appendix narrative still only states the existence and necessary conditions and defers local stability to the code. Add one worked example in Appendix A3: the parameter set, the E_P fixed point, and its eigenvalues, so the result is verifiable without running the notebook.

Authors' response: *Numerical examples are now included (properly tabulated) in Appendix A3 of S1 Appendix.*

5. Parameter-region interpretation.

Appendix A4 already notes that the Table 1 values are arbitrary and that empirical data on the costs and benefits of prokaryotic consortia are limited. Bring one sentence to this effect into the main text, clarifying that the explored regions are internally consistent feasibility regions rather than empirically calibrated ranges. The issue is visibility in the manuscript, not absence from the supplement.

Authors' response: *A statement in this regard is now added to the caption of Table 1 in main text.*

Minor points

State the independence of s and p_Z (benefit vs cost of surface coverage) briefly in the Results, not only in Methods, as it is central to interpretation.

Ensure the s / p_Z renaming (formerly s_Z) and the definition kappa = (1 - n p_Z)(1 - n k_YZ) are consistent across the main text, appendix, and code, and check "consortium"/"consortia" usage.

Authors' response: *Done.*
