# Supplementary material for: Host-initiated microbial association leads to stable ectosymbiosis in an ecological model
Source: PLoS Comput Biol. 2026 Sep 2;22(9):e1014699. doi: 10.1371/journal.pcbi.1014699 (PMC13537694; doi:10.1371/journal.pcbi.1014699)
Supplement: Supplementary file 5 [file pcbi.1014699.s002.zip › S1_Code/S1_Code.pdf]

Supporting Information: Code

Host-initiated microbial association leads to stable ectosymbiosis in an ecological model

Nandakishor Krishnan*, István Zachar, Ádám Kun, Chaitanya S. Gokhale, József Garay

*Corresponding author (Email: nandakishor.kris@gmail.com)

This Wolfram Mathematica notebook (written in v14.3) contains verification of analytical results, numerical examples and analyses, and figure generation. Notebook requires dynamic updating enabled. Notebook contains two sections: Sections 1 and 2, with two and eight subsections, respectively. All input commands within a subsection should be run in the order of appearance. Output figures are prevented from being displayed. See appendices in Supporting Information S1 Appendix for details on the models.

Section 1. Resident system

1.1. Fixed points and linear stability analysis

In[•]:=
```
ClearAll["`*"];
```

Fixed points of the two-species system

In[•]:=
```
(*finding the fixed points of the two-species resident system in Appendix A2 in S1 Appendix*)

f1[x_, y_, u_, v_] := x * (rX - u/(aX + bX * u));
f2[x_, y_, u_, v_] := y * (η * u - ρ - v/(aY + bY * v));
f3[x_, y_, u_, v_] := ϕ * x - ξ * u * y - δU * u;
f4[x_, y_, u_, v_] := βY * y - δV * v;
Simplify[
 Solve[f1[x, y, u, v] == 0 && f2[x, y, u, v] == 0 && f3[x, y, u, v] == 0 && f4[x, y, u, v] == 0, {x, y, u, v}]]
(*Two of the below solutions are not feasible since atleast one density is negative always.*)
```

Out[•]=

$$\Bigg\{\Big\{x \to 0,\ y \to -\frac{aY\,\delta V\,\rho}{\beta Y + bY\,\beta Y\,\rho},\ u \to 0,\ v \to -\frac{aY\,\rho}{1 + bY\,\rho}\Big\},$$

$$\Big\{x \to \frac{aX\,rX\left(-\delta U + \frac{aY\,\delta V\,\xi\,(aX\,rX\,\eta + (-1 + bX\,rX)\,\rho)}{\beta Y\,(-1 + aX\,bY\,rX\,\eta - bY\,\rho + bX\,(rX + bY\,rX\,\rho))}\right)}{(-1 + bX\,rX)\,\phi},\ y \to \frac{aY\,\delta V\,(-aX\,rX\,\eta + \rho - bX\,rX\,\rho)}{\beta Y\,(-1 + aX\,bY\,rX\,\eta - bY\,\rho + bX\,(rX + bY\,rX\,\rho))},$$

$$u \to \frac{aX\,rX}{1 - bX\,rX},\ v \to \frac{aY\,(-aX\,rX\,\eta + \rho - bX\,rX\,\rho)}{-1 + aX\,bY\,rX\,\eta - bY\,\rho + bX\,(rX + bY\,rX\,\rho)}\Big\},\ \Big\{x \to \frac{aX\,rX\,\delta U}{\phi - bX\,rX\,\phi},\ y \to 0,\ u \to \frac{aX\,rX}{1 - bX\,rX},\ v \to 0\Big\},$$

$$\Big\{x \to 0,\ y \to -\frac{\delta U}{\xi},\ u \to \frac{\beta Y\,\delta U + bY\,\beta Y\,\delta U\,\rho - aY\,\delta V\,\xi\,\rho}{bY\,\beta Y\,\delta U\,\eta - aY\,\delta V\,\eta\,\xi},\ v \to -\frac{\beta Y\,\delta U}{\delta V\,\xi}\Big\},\ \{x \to 0,\ y \to 0,\ u \to 0,\ v \to 0\}\Bigg\}$$

Existence conditions of fixed points

In[∘]:=
```
E0 = {0, 0, 0, 0}(*trivial eq.*);
```

$$E1 = \left\{\frac{aX\,rX\,\delta U}{\phi - bX\,rX\,\phi}, 0, \frac{aX\,rX}{1 - bX\,rX}, 0\right\}$$ (*one-sp eq. E1(x*,0,u*,0) exists if 1-bXrX > 0*);

$$E2 = \left\{\frac{aX\,rX\left(-\delta U + \frac{aY\,\delta V\,\xi\,(aX\,rX\,\eta + (-1+bX\,rX)\,\rho)}{\beta Y\,(-1+aX\,bY\,rX\,\eta - bY\,\rho + bX\,(rX + bY\,rX\,\rho))}\right)}{(-1 + bX\,rX)\,\phi}, \frac{aY\,\delta V\,(-aX\,rX\,\eta + \rho - bX\,rX\,\rho)}{\beta Y\,(-1 + aX\,bY\,rX\,\eta - bY\,\rho + bX\,(rX + bY\,rX\,\rho))}, \frac{aX\,rX}{1 - bX\,rX}, \frac{aY\,(-aX\,rX\,\eta + \rho - bX\,rX\,\rho)}{-1 + aX\,bY\,rX\,\eta - bY\,\rho + bX\,(rX + bY\,rX\,\rho)}\right\};$$

```
(*existence conditions of the interior E2(x+,y+,u+,v+) eq.*)

Reduce[Extract[E2, {1}] > 0 && Extract[E2, {2}] > 0 && Extract[E2, {3}] > 0 && Extract[E2, {4}] > 0 &&
  rX > 0 && aX > 0 && bX > 0 && aY > 0 && bY > 0 && η > 0 && ρ > 0 && ϕ > 0 && ξ > 0 && δU > 0 && δV > 0 && βY > 0]
```

Out[∘]=

$$\rho > 0 \,\&\&\, rX > 0 \,\&\&\, 0 < bX < \frac{1}{rX} \,\&\&\, \eta > 0 \,\&\&\, aX > \frac{\rho - bX\,rX\,\rho}{rX\,\eta} \,\&\&$$
$$0 < bY < \frac{1 - bX\,rX}{aX\,rX\,\eta - \rho + bX\,rX\,\rho} \,\&\&\, \xi > 0 \,\&\&\, \delta V > 0 \,\&\&\, \delta U > 0 \,\&\&\, \beta Y > 0 \,\&\&\, aY > 0 \,\&\&\, \phi > 0$$

Jacobian matrices and eigenvalues (stability analysis)

In[∘]:=
```
(*linear stability analysis of the fixed points using Jacobian matrices*)

jacobian[{x_, y_, u_, v_}] = Simplify[
   D[{f1[x, y, u, v], f2[x, y, u, v], f3[x, y, u, v], f4[x, y, u, v]}, {{x, y, u, v}}]] // MatrixForm
(*Jacobian matrix corresponding to the system at arbitrary (x,y,u,v)*)
```

Out[∘]//MatrixForm=

$$\begin{pmatrix} rX - \frac{u}{aX+bX\,u} & 0 & -\frac{aX\,x}{(aX+bX\,u)^2} & 0 \\ 0 & -\frac{v}{aY+bY\,v} + u\,\eta - \rho & y\,\eta & -\frac{aY\,y}{(aY+bY\,v)^2} \\ \phi & -u\,\xi & -\delta U - y\,\xi & 0 \\ 0 & \beta Y & 0 & -\delta V \end{pmatrix}$$

In[∘]:=
```
jacobian[{x, y, u, v} /. {{x, y, u, v} → E0}]
Eigenvalues[%]
(*One eigenvalue is positive real, hence E0(0,0,0,0) is unstable.*)
```

Out[∘]//MatrixForm=

$$\begin{pmatrix} rX & 0 & 0 & 0 \\ 0 & -\rho & 0 & 0 \\ \phi & 0 & -\delta U & 0 \\ 0 & \beta Y & 0 & -\delta V \end{pmatrix}$$

Out[∘]=

$$\{rX, -\delta U, -\delta V, -\rho\}$$

```
In[•]:= FullSimplify[jacobian[{x, y, u, v} /. {{x, y, u, v} → E1}]]
        FullSimplify[Eigenvalues[%]]
        (*One eigenvalue is positive real as aX rX η/(1-bX rX)-ρ > 0 from existence conditions,
        hence E1(x*,0,u*,0) is unstable.*)
```

Out[•]//MatrixForm=

$$\begin{pmatrix} 0 & 0 & \frac{rX\,(-1+bX\,rX)\,\delta U}{\phi} & 0 \\ 0 & \frac{aX\,rX\,\eta}{1-bX\,rX}-\rho & 0 & 0 \\ \phi & \frac{aX\,rX\,\xi}{-1+bX\,rX} & -\delta U & 0 \\ 0 & \beta Y & 0 & -\delta V \end{pmatrix}$$

Out[•]=

$$\left\{-\delta V,\ \frac{aX\,rX\,\eta}{1-bX\,rX}-\rho,\ -\frac{1}{2}\sqrt{\delta U}\left(\sqrt{\delta U}+\sqrt{4\,rX\,(-1+bX\,rX)+\delta U}\right),\ \frac{1}{2}\left(-\delta U+\sqrt{\delta U}\sqrt{4\,rX\,(-1+bX\,rX)+\delta U}\right)\right\}$$

```
In[•]:= FullSimplify[jacobian[{x, y, u, v} /. {{x, y, u, v} → E2}]];
        FullSimplify[Eigenvalues[%]];
        (*The eigenvalues of the Jacobian matrix at the interior
         fixed point E2(x+,y+,u+,v+) are complicated to analyze directly.*)
```

```
In[•]:= (*To simplify the above matrix,
        we define it as M such that all j__ are positive. For the signs of matrix elements,
        substitute x+, y+, u+, and v+ into the general Jacobian without further simplification.*)
```

$$M = \begin{pmatrix} 0 & 0 & -j13 & 0 \\ 0 & 0 & j23 & -j24 \\ j31 & -j32 & -j33 & 0 \\ 0 & j42 & 0 & -j44 \end{pmatrix};$$

```
        Eigenvalues[M];
```

```
In[•]:= (*using Routh-Hurwitz criterion to analyze the stability of the interior fixed point E2*)

        chareq = CharacteristicPolynomial[M, λ]
```

Out[•]=

$$j13\,j24\,j31\,j42 + j24\,j33\,j42\,\lambda + j13\,j31\,j44\,\lambda + j23\,j32\,j44\,\lambda + j13\,j31\,\lambda^2 + j23\,j32\,\lambda^2 + j24\,j42\,\lambda^2 + j33\,j44\,\lambda^2 + j33\,\lambda^3 + j44\,\lambda^3 + \lambda^4$$

```
In[•]:= (*defining coefficients of the charactersitic polynomial of M*)

        A1 = Coefficient[chareq, λ, 3];
        A2 = Coefficient[chareq, λ, 2];
        A3 = Coefficient[chareq, λ, 1];
        A4 = Coefficient[chareq, λ, 0];
```

```
In[•]:= Expand[A1 * A2 - A3]
        Expand[A1 * A2 * A3 - A1 * A1 * A4 - A3 * A3]
        Reduce[A1 * A2 - A3 ≤ 0 && j44 > 0 && j42 > 0 && j33 > 0 && j32 > 0 && j31 > 0 && j24  > 0 && j23 > 0 && j13 > 0,
          {j44, j42, j33, j32, j31, j24, j23, j13}, Reals]
        Reduce[A1 * A2 * A3 - A1 * A1 * A4 - A3 * A3 ≤ 0 && j44 > 0 && j42 > 0 && j33 > 0 && j32 > 0 &&
           j31 > 0 && j24  > 0 && j23 > 0 && j13 > 0, {j44, j42, j33, j32, j31, j24, j23, j13}, Reals]
        (*A1, A2, A3, A4, A1*A2-A3, and A1*A2*A3-A1*A1*A4-A3*A3 are positive.*)
```

Out[•]=

$$j13\,j31\,j33 + j23\,j32\,j33 + j33^2\,j44 + j24\,j42\,j44 + j33\,j44^2$$

Out[•]=

$$j23\,j24\,j32\,j33^2\,j42 + j13^2\,j31^2\,j33\,j44 + 2\,j13\,j23\,j31\,j32\,j33\,j44 + j23^2\,j32^2\,j33\,j44 - 2\,j13\,j24\,j31\,j33\,j42\,j44 + j24\,j33^3\,j42\,j44 + j24^2\,j33\,j42^2\,j44 + j13\,j31\,j33^2\,j44^2 + j23\,j32\,j33^2\,j44^2 + j23\,j24\,j32\,j42\,j44^2 + j24\,j33^2\,j42\,j44^2 + j13\,j31\,j33\,j44^3 + j23\,j32\,j33\,j44^3$$

Out[•]=

False

Out[•]=

False

```
In[•]:= Reduce[A1 * A2 - A3 > 0 && j44 > 0 && j42 > 0 && j33 > 0 && j32 > 0 && j31 > 0 && j24 > 0 && j23 > 0 && j13 > 0,
          {j44, j42, j33, j32, j31, j24, j23, j13}, Reals]
        Reduce[A1 * A2 * A3 - A1 * A1 * A4 - A3 * A3 > 0 && j44 > 0 && j42 > 0 && j33 > 0 && j32 > 0 &&
           j31 > 0 && j24 > 0 && j23 > 0 && j13 > 0, {j44, j42, j33, j32, j31, j24, j23, j13}, Reals]
        (*A1, A2, A3, A4, A1*A2-A3, and A1*A2*A3-A1*A1*A4-A3*A3 are positive.*)
Out[•]=
        j44 > 0 && j42 > 0 && j33 > 0 && j32 > 0 && j31 > 0 && j24 > 0 && j23 > 0 && j13 > 0
Out[•]=
        j44 > 0 && j42 > 0 && j33 > 0 && j32 > 0 && j31 > 0 && j24 > 0 && j23 > 0 && j13 > 0

        (*NOTE: By Routh-Hurwitz criterion, the real part of all eigenvalues of M are negative;
        hence, E2(x+,y+,u+,v+) is always stable if it exists.*)
```

Linear stability analysis in the one-species system

```
In[•]:= (*finding fixed points of the one-species resident system in Appendix A1 in S1 Appendix*)
        f1[x_, u_] := x * (rX - u/(aX + bX * u));
        f2[x_, u_] := ϕ * x - δU * u;
        Simplify[Solve[f1[x, u] == 0 && f2[x, u] == 0, {x, u}]]
```

Out[•]=

$$\left\{\left\{x \to \frac{aX\ rX\ \delta U}{\phi - bX\ rX\ \phi},\ u \to \frac{aX\ rX}{1 - bX\ rX}\right\},\ \{x \to 0,\ u \to 0\}\right\}$$

```
In[•]:= (*defining fixed points*)
        oneE0 = {0, 0} (*trivial eq.*);
```

$$\text{oneE1} = \left\{\frac{aX\ rX\ \delta U}{\phi - bX\ rX\ \phi},\ \frac{aX\ rX}{1 - bX\ rX}\right\} \text{(*interior eq.*);}$$

```
In[•]:= (*Jacobian matrix corresponding to the system at arbitrary (x,u)*)

        jacobian[{x_, u_}] = Simplify[D[{f1[x, u], f2[x, u]}, {{x, u}}]] // MatrixForm
```

Out[•]//MatrixForm=

$$\begin{pmatrix} rX - \frac{u}{aX+bX\ u} & -\frac{aX\ x}{(aX+bX\ u)^2} \\ \phi & -\delta U \end{pmatrix}$$

```
In[•]:= jacobian[{x, u} /. {{x, u} → oneE0}]
        Eigenvalues[%]
        (*One eigenvalue is positive real, hence unstable.*)
```

Out[•]//MatrixForm=

$$\begin{pmatrix} rX & 0 \\ \phi & -\delta U \end{pmatrix}$$

Out[•]=

$$\{rX,\ -\delta U\}$$

```
In[•]:= FullSimplify[jacobian[{x, u} /. {{x, u} → oneE1}]]
        FullSimplify[Eigenvalues[%]]
        (*Real part of both eigenvalues are negative, hence stable.*)
```

Out[•]//MatrixForm=

$$\begin{pmatrix} 0 & \frac{rX\ (-1+bX\ rX)\ \delta U}{\phi} \\ \phi & -\delta U \end{pmatrix}$$

Out[•]=

$$\left\{-\frac{1}{2}\sqrt{\delta U}\left(\sqrt{\delta U} + \sqrt{4\ rX\ (-1 + bX\ rX) + \delta U}\right),\ \frac{1}{2}\left(-\delta U + \sqrt{\delta U}\ \sqrt{4\ rX\ (-1 + bX\ rX) + \delta U}\right)\right\}$$

1.2. Numerical analysis and figure generation

```
In[•]:= ClearAll["`*"];
```

Parameters, definitions, and stability analysis of the two-species system

In[•]:=
```
(*arbitrary parameter values based on model conditions*)
para2 = {w → 500, kX → 0.5, cX → 3, lX → 4, dX → 0.33, kY → 0.1, cY → 0.3, lY → 1.2,
   dY → 0.1, Ω → 10, aX → 1, bX → 0.01, aY → 1.5, bY → 0.015, δU → 1, δV → 1, βY → 2};

(*substitutions to simplify the system as in Appendix A2 in S1 Appendix*)
rX = (kX * w - lX)/cX * (N[Log[2]]) - dX;
η = kY/(Ω * cY) * (N[Log[2]]);
ρ = lY/cY * (N[Log[2]]) + dY;
ϕ = kX * w;
ξ = kY/Ω;

(*two-species dynamical system*)
dynamics2 = {
   x'[t] == x[t] * (rX - u[t]/(aX + bX * u[t])),
   y'[t] == y[t] * (η * u[t] - ρ - v[t]/(aY + bY * v[t])),
   u'[t] == ϕ * x[t] - ξ * u[t] * y[t] - δU * u[t],
   v'[t] == βY * y[t] - δV * v[t]
  };
```

In[•]:=
```
(*existence conditions of fixed points*)
1 - bX * rX > 0 /. para2
ρ/η < (aX rX)/(1 - bX rX) < ρ/η + 1/(η * bY) /. para2
```

Out[•]=
```
True
```

Out[•]=
```
True
```

In[•]:=
```
(*finding the numerical fixed points of the two-species resident system*)
f1[x_, y_, u_, v_] := x * (rX - u/(aX + bX * u)) /. para2;
f2[x_, y_, u_, v_] := y * (η * u - ρ - v/(aY + bY * v)) /. para2;
f3[x_, y_, u_, v_] := ϕ * x - ξ * u * y - δU * u /. para2;
f4[x_, y_, u_, v_] := βY * y - δV * v /. para2;
Simplify[NSolve[{f1[x, y, u, v] == 0, f2[x, y, u, v] == 0,
   f3[x, y, u, v] == 0, f4[x, y, u, v] == 0, x ≥ 0, y ≥ 0, u ≥ 0, v ≥ 0}, {x, y, u, v}]]
```

Out[•]=
```
{{x → 0, v → 0, y → 0, u → 0}, {x → 0.519711, v → 0, y → 0, u → 129.928},
 {x → 0.520216, v → 0.194445, y → 0.0972227, u → 129.928}}
```

In[ ]:=
```
E0 = {0, 0, 0, 0}(*trivial eq.*)
```

$$E1 = \left\{\frac{aX\,rX\,\delta U}{\phi - bX\,rX\,\phi},\ 0,\ \frac{aX\,rX}{1 - bX\,rX},\ 0\right\} \ /.\ para2 \text{(*one-sp eq. E1(x*,0,u*,0)*)}$$

$$E2 = \left\{\frac{aX\,rX\left(-\delta U + \frac{aY\,\delta V\,\xi\,(aX\,rX\,\eta + (-1 + bX\,rX)\,\rho)}{\beta Y\,(-1 + aX\,bY\,rX\,\eta - bY\,\rho + bX\,(rX + bY\,rX\,\rho))}\right)}{(-1 + bX\,rX)\,\phi},\ \frac{aY\,\delta V\,(-aX\,rX\,\eta + \rho - bX\,rX\,\rho)}{\beta Y\,(-1 + aX\,bY\,rX\,\eta - bY\,\rho + bX\,(rX + bY\,rX\,\rho))},\ \frac{aX\,rX}{1 - bX\,rX},\ \frac{aY\,(-aX\,rX\,\eta + \rho - bX\,rX\,\rho)}{-1 + aX\,bY\,rX\,\eta - bY\,\rho + bX\,(rX + bY\,rX\,\rho)}\right\} \ /.\ para2 \text{(*interior eq. E2(x+,y+,u+,v+)*)}$$

Out[ ]=
```
{0, 0, 0, 0}
```

Out[ ]=
```
{0.519711, 0, 129.928, 0}
```

Out[ ]=
```
{0.520216, 0.0972227, 129.928, 0.194445}
```

In[ ]:=
```
(*linear stability analysis of the fixed points in the two-species system*)

jacobian[{x_, y_, u_, v_}] =
  FullSimplify[D[{f1[x, y, u, v], f2[x, y, u, v], f3[x, y, u, v], f4[x, y, u, v]}, {{x, y, u, v}}]];
```

In[ ]:=
```
Eigenvalues[jacobian[{x, y, u, v} /. {{x, y, u, v} → E0}]]
(*unstable*)
```

Out[ ]=
```
{56.5081, -2.87259, -1., -1.}
```

In[ ]:=
```
Eigenvalues[jacobian[{x, y, u, v} /. {{x, y, u, v} → E1}]]
(*unstable*)
```

Out[ ]=
```
{-0.5 + 4.93219 i, -0.5 - 4.93219 i, -1. + 0. i, 0.129379 + 0. i}
```

In[ ]:=
```
Eigenvalues[jacobian[{x, y, u, v} /. {{x, y, u, v} → E2}]]
(*stable*)
```

Out[ ]=
```
{-0.500486 + 4.93485 i, -0.500486 - 4.93485 i, -0.847689 + 0. i, -0.152311 + 0. i}
```

Parameters, definitions, and stability analysis of the one-species system

In[ ]:=
```
(*arbitrary parameter values based on model conditions*)
para1 = {kX → 0.5, w → 500, cX → 3, lX → 4, dX → 0.33, aX → 1, bX → 0.01, δU → 1};

(*substitutions to simplify the system as in Appendix A1 in S1 Appendix*)
rX = (kX * w - lX) / cX * (N[Log[2]]) - dX;
ϕ = kX * w;

(*one-species dynamical system*)
dynamics1 = {
   x'[t] == x[t] * (rX - u[t] / (aX + bX * u[t])),
   u'[t] == ϕ * x[t] - δU * u[t]
  };
```

In[ ]:=
```
(*existence of interior fixed point*)
1 - bX * rX > 0 /. para1
```

Out[ ]=
```
True
```

In[ ]:=
```
(*finding fixed points of the one-species system numerically*)
f1[x_, u_] := x * (rX - u/(aX + bX * u)) /. para1;
f2[x_, u_] := ϕ * x - δU * u /. para1;
Simplify[NSolve[{f1[x, u] == 0, f2[x, u] == 0}, {x, u}]]
```
Out[ ]=
```
{{x → 0.519711, u → 129.928}, {x → 0., u → 0.}}
```
In[ ]:=
```
oneE0 = {0, 0} (*trivial eq.*)
oneE1 = {(aX rX δU)/(ϕ - bX rX ϕ), (aX rX)/(1 - bX rX)} /. para1(*interior eq. (x*,u* ) *)
```
Out[ ]=
```
{0, 0}
```
Out[ ]=
```
{0.519711, 129.928}
```
In[ ]:=
```
(*linear stability analysis of the two fixed points in the one-species system*)

jacobian[{x_, u_}] = Simplify[D[{f1[x, u], f2[x, u]}, {{x, u}}]];
```
In[ ]:=
```
Eigenvalues[jacobian[{x, u} /. {{x, u} → oneE0}]]
(*unstable*)
```
Out[ ]=
```
{56.5081, -1.}
```
In[ ]:=
```
Eigenvalues[jacobian[{x, u} /. {{x, u} → oneE1}]]
(*stable*)
```
Out[ ]=
```
{-0.5 + 4.93219 i, -0.5 - 4.93219 i}
```
Figure generation

In[ ]:=
```
(*plotting the solutions of the one-species system w.r.t time;
initial condition: arbitrary point in the nbhd of trivial eq.*)

Plot[Evaluate[NDSolveValue[{dynamics1 /. para1,
     x[0] == 0.01, u[0] == 0.01}, {x[t], u[t]}, {t, 0, 100}]], {t, 0, 100},
  ScalingFunctions → "Log", PlotRange → {{-0.6, 20.6}, All}, PlotPoints → 600,

  PlotStyle → {Directive[RGBColor[0.24, 0.6, 0.8], Thickness[0.008]],
    Directive[RGBColor[0.5, 0.2, 0.9], Thickness[0.008]]},

  Frame → True, FrameTicks → {{All, None}, {All, None}},
  FrameLabel → {Style["Time", FontFamily → "Arial", Black, 18],
    Style["Densities", FontFamily → "Arial", Black, 18]},
  FrameStyle → Directive[Black, Thickness[0.005], 14],

  Epilog → {Inset[Style["Free-living host, x", FontFamily → "Arial", Black, 18], {15.5, -1.5}],
    Inset[Style["Host metabolite conc., u", FontFamily → "Arial", Black, 18], {14, 4}]},

  ImageSize → 400, ImagePadding → {{65, 10}, {50, 10}}, AspectRatio → 0.75];
```

```
In[]:= (*phase portrait of the one-species system with a trajectory
         from an arbitrary point in the nbhd of the trivial eq. displayed*)

       phase = VectorPlot[Evaluate[{f1[x, u] /. para1, f2[x, u] /. para1}], {x, 0, 12}, {u, 0, 300},
          PlotRange → {{-0.5, 12.5}, {-10, 310}}, VectorColorFunction → None, VectorStyle → Gray,

          FrameLabel → {Style["Free-living host density, x", FontFamily → "Arial", 20, Black],
            Style["Host metabolite conc., u", FontFamily → "Arial", 20, Black]},
          FrameStyle → Directive[Black, Thickness[0.006], 14]];

       icsol = NDSolve[{dynamics1 /. para1, x[0] == 0.01, u[0] == 0.01}, {x, u}, {t, 0, 100}];
       trajsol = ParametricPlot[Evaluate[{x[t], u[t]} /. icsol], {t, 0, 100},
          PlotRange → {{-0.5, 12.5}, {-10, 310}}, PlotPoints → 600, PlotStyle → Directive[Black]];

       Show[phase, trajsol, Epilog → {Red, PointSize[Medium], Point[oneE1]},
         ImageSize → 330, AspectRatio → 1, ImagePadding → {{55, 10}, {50, 10}}];

In[]:= fn = 0.01 * # &(*to scale the u-axis*);
       phase = StreamPlot[Evaluate[{f1[x, u] /. para1, f2[x, u] /. para1}], {x, 0, 12}, {u, 0, 300},
          PlotRange → {{-0.5, 12.5}, All}, ScalingFunctions → {None, {fn, InverseFunction[fn]}},
          StreamColorFunction → None, StreamStyle → Gray, StreamScale → Large, StreamPoints → 20,

          PlotLabel → Style["(A) Self-inhibiting hosts", FontFamily → "Arial", 22, Black],
          FrameLabel → {Style["Free-living host density, x", FontFamily → "Arial", 20, Black],
            Style["Host metabolite conc., u", FontFamily → "Arial", 20, Black]},
          FrameStyle → Directive[Black, Thickness[0.006], 14],
          FrameTicks → {{{0, 50, 100, 150, 200, 250, 300}, Automatic}, {All, Automatic}}];

       icsol = NDSolve[{dynamics1 /. para1, x[0] == 0.01, u[0] == 0.01}, {x, u}, {t, 0, 100}];
       trajsol = ParametricPlot[Evaluate[{x[t], u[t]} /. icsol],
          {t, 0, 100}, ScalingFunctions → {None, {fn, InverseFunction[fn]}},
          PlotRange → {{-0.5, 12.5}, All}, PlotPoints → 600, PlotStyle → Directive[Black]];

       figres1 = Show[phase, trajsol, Epilog → {Red, PointSize[Medium], Point[{0.52, 1.30}]},
          ImageSize → 330, AspectRatio → 1, ImagePadding → {{55, 10}, {50, 10}}];
```

In[ ]:=
```
(*plotting the solutions of the two-species resident system w.r.t time;
initial conditions: arbitrary points in the nbhd of trivial eq. and one-species eq.*)

DsolRes = ParametricNDSolveValue[{dynamics2 /. para2, x[0] == x0, y[0] == y0, u[0] == u0, v[0] == v0},
   {x[t], y[t], u[t], v[t]}, {t, 0, 100}, {x0, y0, u0, v0}];

icres0 = DsolRes[0.01, 0.01, 0.01, 0.01];
(*arbitrary point in the nbhd of trivial eq. E0(0,0,0,0) *)
icres1 = DsolRes[0.53, 0.01, 131, 0.01];
(*arbitrary point in the nbhd of one-species eq. E1(x*,0,u*,0) *)

figres2 = Plot[Evaluate[Join[icres0, icres1]], {t, 0, 100},
   ScalingFunctions → "Log", PlotRange → {{-1, 41}, All}, PlotPoints → 300,

   PlotLabel → Style["(B) Stable syntrophic pair", FontFamily → "Arial", 22, Black],
   PlotStyle → {Directive[RGBColor[0.24, 0.6, 0.8], Thickness[0.008]],
     Directive[RGBColor[0.95, 0.627, 0.1425], Thickness[0.008]],
     Directive[RGBColor[0.5, 0.2, 0.9], Thickness[0.008]],
     Directive[RGBColor[0.6, 0.2, 0.3], Thickness[0.008]]},

   Frame → True, FrameStyle → Directive[Black, Thickness[0.006], 15],
   FrameTicks → {{All, None}, {All, None}}, FrameLabel → {Style["Time", FontFamily → "Arial",
      Black, 20], Style["Densities", FontFamily → "Arial", Black, 20]},

   PlotLegends → Placed[
     LineLegend[{Style["Free-living host, x", FontFamily → "Arial", 14, Black],
       Style["Free-living symbiont, y", FontFamily → "Arial", 14, Black],
       Style["Host metabolite conc., u", FontFamily → "Arial", 14, Black],
       Style["Symbiont metabolite conc., v", FontFamily → "Arial",
        14, Black]}, LegendLayout → "Column"],
     {0.66, 0.22}],

   ImagePadding → {{70, 10}, {50, 10}}, ImageSize → 430, AspectRatio → 0.75];
```

In[ ]:=
```
(*Fig 2 in the main text*)
figres = GraphicsRow[{figres1, figres2}, Spacings → 20, ImageSize → {750, 350}];
```

In[ ]:=
```
SetDirectory[NotebookDirectory[]];
Export["Fig2.tiff", figres, ImageResolution → 600];
```

Section 2. Resident-mutant system

2.1. Fixed-points and linear stability analysis

In[∘]:=
```
ClearAll["`*"];
```

Fixed points of resident-mutant system

In[∘]:=
```
(*finding fixed points of the resident-mutant system in Appendix A3 in S1 Appendix*)

f1[x_, y_, z_, u_, v_] := x * (rX - u/(aX + bX * u));
f2[x_, y_, z_, u_, v_] := y * (η * u - ρ - v/(aY + bY * v));
f3[x_, y_, z_, u_, v_] := z * (rZ - u/(aZ + bX * u));
f4[x_, y_, z_, u_, v_] := ϕ * x + κ * ϕ * z - ξ * u * y - δU * u;
f5[x_, y_, z_, u_, v_] := βY * y + βZ * z - δV * v;
Simplify[Solve[{f1[x, y, z, u, v] == 0 && f2[x, y, z, u, v] == 0 &&
    f3[x, y, z, u, v] == 0 && f4[x, y, z, u, v] == 0 && f5[x, y, z, u, v] == 0}, {x, y, z, u, v}]]
(*Two of the below solutions are not feasible since atleast one density is negative.*)
```

Out[∘]=

$$\left\{\left\{x\to 0,\ y\to -\frac{aY\,\delta V\,\rho}{\beta Y+bY\,\beta Y\,\rho},\ z\to 0,\ u\to 0,\ v\to -\frac{aY\,\rho}{1+bY\,\rho}\right\},\right.$$

$$\left\{x\to \frac{aX\,rX\left(-\delta U+\frac{aY\,\delta V\,\xi\,(aX\,rX\,\eta+(-1+bX\,rX)\,\rho)}{\beta Y\,(-1+aX\,bY\,rX\,\eta-bY\,\rho+bX\,(rX+bY\,rX\,\rho))}\right)}{(-1+bX\,rX)\,\phi},\ y\to \frac{aY\,\delta V\,(-aX\,rX\,\eta+\rho-bX\,rX\,\rho)}{\beta Y\,(-1+aX\,bY\,rX\,\eta-bY\,\rho+bX\,(rX+bY\,rX\,\rho))},\right.$$

$$\left.z\to 0,\ u\to \frac{aX\,rX}{1-bX\,rX},\ v\to \frac{aY\,(-aX\,rX\,\eta+\rho-bX\,rX\,\rho)}{-1+aX\,bY\,rX\,\eta-bY\,\rho+bX\,(rX+bY\,rX\,\rho)}\right\},$$

$$\left\{x\to \frac{aX\,rX\,\delta U}{\phi-bX\,rX\,\phi},\ y\to 0,\ z\to 0,\ u\to \frac{aX\,rX}{1-bX\,rX},\ v\to 0\right\},$$

$$\left\{x\to 0,\ y\to \frac{aZ^2\,bY\,rZ^2\,\beta Z\,\delta U\,\eta-aY\,(-1+bX\,rZ)^2\,\delta V\,\kappa\,\rho\,\phi+aZ\,rZ\,(-1+bX\,rZ)\,(\beta Z\,(\delta U+bY\,\delta U\,\rho)-aY\,\delta V\,\eta\,\kappa\,\phi)}{(-1+aZ\,bY\,rZ\,\eta-bY\,\rho+bX\,(rZ+bY\,rZ\,\rho))\,(-aZ\,rZ\,\beta Z\,\xi+(-1+bX\,rZ)\,\beta Y\,\kappa\,\phi)},\right.$$

$$z\to -\frac{aZ\,rZ\,(aY\,\delta V\,\xi\,(-aZ\,rZ\,\eta+\rho-bX\,rZ\,\rho)+\beta Y\,\delta U\,(-1+aZ\,bY\,rZ\,\eta-bY\,\rho+bX\,(rZ+bY\,rZ\,\rho)))}{(-1+aZ\,bY\,rZ\,\eta-bY\,\rho+bX\,(rZ+bY\,rZ\,\rho))\,(-aZ\,rZ\,\beta Z\,\xi+(-1+bX\,rZ)\,\beta Y\,\kappa\,\phi)},$$

$$\left.u\to \frac{aZ\,rZ}{1-bX\,rZ},\ v\to \frac{aY\,(-aZ\,rZ\,\eta+\rho-bX\,rZ\,\rho)}{-1+aZ\,bY\,rZ\,\eta-bY\,\rho+bX\,(rZ+bY\,rZ\,\rho)}\right\},$$

$$\left\{x\to 0,\ y\to 0,\ z\to \frac{aZ\,rZ\,\delta U}{\kappa\,\phi-bX\,rZ\,\kappa\,\phi},\ u\to \frac{aZ\,rZ}{1-bX\,rZ},\ v\to \frac{aZ\,rZ\,\beta Z\,\delta U}{\delta V\,\kappa\,\phi-bX\,rZ\,\delta V\,\kappa\,\phi}\right\},$$

$$\left.\left\{x\to 0,\ y\to -\frac{\delta U}{\xi},\ z\to 0,\ u\to \frac{\beta Y\,\delta U+bY\,\beta Y\,\delta U\,\rho-aY\,\delta V\,\xi\,\rho}{bY\,\beta Y\,\delta U\,\eta-aY\,\delta V\,\eta\,\xi},\ v\to -\frac{\beta Y\,\delta U}{\delta V\,\xi}\right\},\ \{x\to 0,\ y\to 0,\ z\to 0,\ u\to 0,\ v\to 0\}\right\}$$

Existence of fixed points

In[ ]:=

```
E0 = {0, 0, 0, 0, 0}(*trivial eq. E0*);
```

$$E1 = \left\{\frac{aX\,rX\,\delta U}{\phi - bX\,rX\,\phi}, 0, 0, \frac{aX\,rX}{1 - bX\,rX}, 0\right\}\text{(*one-sp eq. E1(x*,0,0,u*,0) exists if 1-bXrX > 0*);}$$

$$E2 = \left\{\frac{aX\,rX\left(-\delta U + \frac{aY\,\delta V\,\xi\,(aX\,rX\,\eta + (-1+bX\,rX)\,\rho)}{\beta Y\,(-1+aX\,bY\,rX\,\eta - bY\,\rho + bX\,(rX+bY\,rX\,\rho))}\right)}{(-1 + bX\,rX)\,\phi}, \frac{aY\,\delta V\,(-aX\,rX\,\eta + \rho - bX\,rX\,\rho)}{\beta Y\,(-1 + aX\,bY\,rX\,\eta - bY\,\rho + bX\,(rX + bY\,rX\,\rho))},\right.$$
$$\left. 0, \frac{aX\,rX}{1 - bX\,rX}, \frac{aY\,(-aX\,rX\,\eta + \rho - bX\,rX\,\rho)}{-1 + aX\,bY\,rX\,\eta - bY\,\rho + bX\,(rX + bY\,rX\,\rho)}\right\}\text{(*eq. E2(x+,y+,0,u+,v+)*);}$$

$$EP = \left\{0, \frac{aZ^2\,bY\,rZ^2\,\beta Z\,\delta U\,\eta - aY\,(-1 + bX\,rZ)^2\,\delta V\,\kappa\,\rho\,\phi + aZ\,rZ\,(-1 + bX\,rZ)\,(\beta Z\,(\delta U + bY\,\delta U\,\rho) - aY\,\delta V\,\eta\,\kappa\,\phi)}{(-1 + aZ\,bY\,rZ\,\eta - bY\,\rho + bX\,(rZ + bY\,rZ\,\rho))\,(-aZ\,rZ\,\beta Z\,\xi + (-1 + bX\,rZ)\,\beta Y\,\kappa\,\phi)},\right.$$
$$-\frac{aZ\,rZ\,(aY\,\delta V\,\xi\,(-aZ\,rZ\,\eta + \rho - bX\,rZ\,\rho) + \beta Y\,\delta U\,(-1 + aZ\,bY\,rZ\,\eta - bY\,\rho + bX\,(rZ + bY\,rZ\,\rho)))}{(-1 + aZ\,bY\,rZ\,\eta - bY\,\rho + bX\,(rZ + bY\,rZ\,\rho))\,(-aZ\,rZ\,\beta Z\,\xi + (-1 + bX\,rZ)\,\beta Y\,\kappa\,\phi)},$$
$$\left.\frac{aZ\,rZ}{1 - bX\,rZ}, \frac{aY\,(-aZ\,rZ\,\eta + \rho - bX\,rZ\,\rho)}{-1 + aZ\,bY\,rZ\,\eta - bY\,\rho + bX\,(rZ + bY\,rZ\,\rho)}\right\}\text{(*eq. EP(0, y^hat, z^hat, u^hat, v^hat)*);}$$

$$EM = \left\{0, 0, \frac{aZ\,rZ\,\delta U}{\kappa\,\phi - bX\,rZ\,\kappa\,\phi}, \frac{aZ\,rZ}{1 - bX\,rZ}, \frac{aZ\,rZ\,\beta Z\,\delta U}{\delta V\,\kappa\,\phi - bX\,rZ\,\delta V\,\kappa\,\phi}\right\}$$
$$\text{(*eq. EM(0,0, z^bar, u^bar, v^bar) exists if 1-bXrZ > 0*);}$$

```
(*existence conditions of E2*)
Simplify[Reduce[Extract[E2, {1}] > 0 && Extract[E2, {2}] > 0 && Extract[E2, {4}] > 0 &&
    Extract[E2, {5}] > 0 && rX > rZ > 0 && bX > 0 && aY > 0 && bY > 0 && aZ > aX > 0 &&
    δU > 0 && δV > 0 && ϕ > 0 && ξ > 0 && η > 0 && ρ > 0 && βY > 0 && κ > 0 && βZ > 0]]
```

```
(*existence conditions of EP*)
Simplify[Reduce[Extract[EP, {2}] > 0 && Extract[EP, {3}] > 0 && Extract[EP, {4}] > 0 &&
    Extract[EP, {5}] > 0 && rX > rZ > 0 && bX > 0 && aY > 0 && bY > 0 && aZ > aX > 0 &&
    δU > 0 && δV > 0 && ϕ > 0 && ξ > 0 && η > 0 && ρ > 0 && βY > 0 && κ > 0 && βZ > 0]]
```

Out[ ]=

$$rZ > 0\ \&\&\ rX > 0\ \&\&\ bX > 0\ \&\&\ \rho > 0\ \&\&\ \eta > 0\ \&\&\ bY > 0\ \&\&\ \xi > 0\ \&\&\ \delta V > 0\ \&\&\ \delta U > 0\ \&\&\ \beta Y > 0\ \&\&\ aY > 0\ \&\&\ \phi > 0\ \&\&$$
$$\kappa > 0\ \&\&\ \beta Z > 0\ \&\&\ rZ < rX\ \&\&\ bX\,rX < 1\ \&\&\ \rho < aX\,rX\,\eta + bX\,rX\,\rho\ \&\&\ aX < aZ\ \&\&\ rX\,(bX + aX\,bY\,\eta + bX\,bY\,\rho) < 1 + bY\,\rho$$

Out[ ]=

$$\rho > 0\ \&\&\ rZ > 0\ \&\&\ 0 < bX < \frac{1}{rZ}\ \&\&\ \eta > 0\ \&\&\ rZ\,\eta\,(aZ\,rZ\,\eta + (-1 + bX\,rZ)\,\rho) > 0\ \&\&$$
$$0 < bY < \frac{1 - bX\,rZ}{aZ\,rZ\,\eta - \rho + bX\,rZ\,\rho}\ \&\&\ \xi > 0\ \&\&\ \delta V > 0\ \&\&\ \delta U > 0\ \&\&\ \beta Y > 0\ \&\&\ aY > 0\ \&\&\ \phi > 0\ \&\&$$
$$\beta Z > 0\ \&\&\ \kappa > \frac{aZ\,rZ\,\beta Z\,\delta U\,(-1 + aZ\,bY\,rZ\,\eta - bY\,\rho + bX\,(rZ + bY\,rZ\,\rho))}{aY\,(-1 + bX\,rZ)\,\delta V\,(aZ\,rZ\,\eta + (-1 + bX\,rZ)\,\rho)\,\phi}\ \&\&\ rX > rZ\ \&\&\ 0 < aX < aZ$$

Stability analysis

In[]:=
```
(*linear stability analysis of the fixed points using Jacobian matrices*)

jacobian[{x_, y_, z_, u_, v_}] =
 Simplify[D[{f1[x, y, z, u, v], f2[x, y, z, u, v], f3[x, y, z, u, v], f4[x, y, z, u, v],
     f5[x, y, z, u, v]}, {{x, y, z, u, v}}]] // MatrixForm
(*Jacobian matrix corresponding to the resident-mutant system at arbitrary (x,y,z,u,v)*)
```

Out[]//MatrixForm=

$$\begin{pmatrix} rX - \frac{u}{aX+bX\,u} & 0 & 0 & -\frac{aX\,x}{(aX+bX\,u)^2} & 0 \\ 0 & -\frac{v}{aY+bY\,v} + u\,\eta - \rho & 0 & y\,\eta & -\frac{aY\,y}{(aY+bY\,v)^2} \\ 0 & 0 & rZ - \frac{u}{aZ+bX\,u} & -\frac{aZ\,z}{(aZ+bX\,u)^2} & 0 \\ \phi & -u\,\xi & \kappa\,\phi & -\delta U - y\,\xi & 0 \\ 0 & \beta Y & \beta Z & 0 & -\delta V \end{pmatrix}$$

In[]:=
```
jacobian[{x, y, z, u, v} /. {{x, y, z, u, v} → E0}]
Eigenvalues[%]
(*One eigenvalue is positive real, hence E0 is unstable*)
```

Out[]//MatrixForm=

$$\begin{pmatrix} rX & 0 & 0 & 0 & 0 \\ 0 & -\rho & 0 & 0 & 0 \\ 0 & 0 & rZ & 0 & 0 \\ \phi & 0 & \kappa\,\phi & -\delta U & 0 \\ 0 & \beta Y & \beta Z & 0 & -\delta V \end{pmatrix}$$

Out[]=

$$\{rX,\ rZ,\ -\delta U,\ -\delta V,\ -\rho\}$$

In[]:=
```
FullSimplify[jacobian[{x, y, z, u, v} /. {{x, y, z, u, v} → E1}]]
FullSimplify[Eigenvalues[%]]
(*One eigenvalue is positive real as (aX rX η)/(1-bX rX)-ρ > from existence condition of E2,
hence E1 is unstable*)
```

Out[]//MatrixForm=

$$\begin{pmatrix} 0 & 0 & 0 & \frac{rX\,(-1+bX\,rX)\,\delta U}{\phi} & 0 \\ 0 & \frac{aX\,rX\,\eta}{1-bX\,rX} - \rho & 0 & 0 & 0 \\ 0 & 0 & -\frac{aX\,rX}{aZ+aX\,bX\,rX-aZ\,bX\,rX} + rZ & 0 & 0 \\ \phi & \frac{aX\,rX\,\xi}{-1+bX\,rX} & \kappa\,\phi & -\delta U & 0 \\ 0 & \beta Y & \beta Z & 0 & -\delta V \end{pmatrix}$$

Out[]=

$$\left\{-\frac{aX\,rX}{aZ + aX\,bX\,rX - aZ\,bX\,rX} + rZ,\ -\delta V,\ \frac{aX\,rX\,\eta}{1 - bX\,rX} - \rho,\right.$$
$$\left.\frac{1}{2}\left(-\delta U + \sqrt{\delta U}\ \sqrt{4\,rX\,(-1 + bX\,rX) + \delta U}\right),\ -\frac{1}{2}\sqrt{\delta U}\ \left(\sqrt{\delta U} + \sqrt{4\,rX\,(-1 + bX\,rX) + \delta U}\right)\right\}$$

In[]:=
```
FullSimplify[jacobian[{x, y, z, u, v} /. {{x, y, z, u, v} → E2}]];
FullSimplify[Eigenvalues[%]];

(* NOTE: Stability condition of E2 is determined based on the two-species resident system;
 all eigenvalues have negative real part if rZ - (aX rX)/(aZ+aX bX rX-aZ bX rX) < 0, i.e., (aZ rZ)/(1-rZ bX) < (aX rX)/(1-bX rX).)
```

In[]:=
```
FullSimplify[jacobian[{x, y, z, u, v} /. {{x, y, z, u, v} → EP}]];

(* NOTE: Stability condition of fixed point EP is
  difficult to obtain analytically due to the complexity of the Jacobian;
however, rX-(aZ rZ)/(aX-aX bX rZ+aZ bX rZ) < 0 is necessary, i.e., (aZ rZ)/(1-rZ bX) > (aX rX)/(1-bX rX).*)
```

In[∘]:=
```
FullSimplify[jacobian[{x, y, z, u, v} /. {{x, y, z, u, v} → EM}]]
FullSimplify[Eigenvalues[%]]
```

Out[∘]//MatrixForm=

$$\begin{pmatrix} rX - \frac{aZ\ rZ}{aX - aX\ bX\ rZ + aZ\ bX\ rZ} & 0 & 0 & 0 & 0 \\ 0 & -\rho + aZ\ rZ \left(\frac{\eta}{1 - bX\ rZ} + \frac{\beta Z\ \delta U}{-aZ\ bY\ rZ\ \beta Z\ \delta U + aY\ (-1 + bX\ rZ)\ \delta V\ \kappa\ \phi}\right) & 0 & 0 & 0 \\ 0 & 0 & 0 & \frac{rZ\ (-1 + bX\ rZ)\ \delta U}{\kappa\ \phi} & 0 \\ \phi & \frac{aZ\ rZ\ \xi}{-1 + bX\ rZ} & \kappa\ \phi & -\delta U & 0 \\ 0 & \beta Y & \beta Z & 0 & -\delta V \end{pmatrix}$$

Out[∘]=

$$\left\{\frac{aZ\ (-1 + bX\ rX)\ rZ + aX\ (rX - bX\ rX\ rZ)}{aX - aX\ bX\ rZ + aZ\ bX\ rZ},\ -\delta V,\right.$$
$$-\rho + aZ\ rZ \left(\frac{\eta}{1 - bX\ rZ} + \frac{\beta Z\ \delta U}{-aZ\ bY\ rZ\ \beta Z\ \delta U + aY\ (-1 + bX\ rZ)\ \delta V\ \kappa\ \phi}\right),$$
$$\left.\frac{1}{2}\left(-\delta U + \sqrt{\delta U}\ \sqrt{4\ rZ\ (-1 + bX\ rZ) + \delta U}\right),\ -\frac{1}{2}\sqrt{\delta U}\left(\sqrt{\delta U} + \sqrt{4\ rZ\ (-1 + bX\ rZ) + \delta U}\right)\right\}$$

(*EM is stable if: 1) $\frac{aZ\ (-1+bX\ rX)\ rZ + aX\ (rX - bX\ rX\ rZ)}{aX - aX\ bX\ rZ + aZ\ bX\ rZ} < 0$ (i.e., $\frac{aZ\ rZ}{1 - rZ\ bX} > \frac{aX\ rX}{1 - bX\ rX}$) and 2) $-\rho + aZ\ rZ \left(\frac{\eta}{1 - bX\ rZ} + \frac{\beta Z\ \delta U}{-aZ\ bY\ rZ\ \beta Z\ \delta U + aY\ (-1+bX\ rZ)\ \delta V\ \kappa\ \phi}\right) < 0$ *)

In[∘]:= (* NOTE: y^hat (i.e., y@EP) < 0 if EM is stable; EP does not exist if EM is stable*)

Reduce[Extract[EP, {2}] ≥ 0 && $\frac{aZ\ (-1 + bX\ rX)\ rZ + aX\ (rX - bX\ rX\ rZ)}{aX - aX\ bX\ rZ + aZ\ bX\ rZ} < 0$ &&
$-\rho + aZ\ rZ \left(\frac{\eta}{1 - bX\ rZ} + \frac{\beta Z\ \delta U}{-aZ\ bY\ rZ\ \beta Z\ \delta U + aY\ (-1 + bX\ rZ)\ \delta V\ \kappa\ \phi}\right) < 0$ &&
(1 - bX rZ) > 0 && (1 - bX rX) > 0 && aZ > aX > 0 && βY > 0 && βZ > 0 && δU > 0 && δV > 0 &&
rX > rZ > 0 && bX > 0 && bY > 0 && aY > 0 && κ > 0 && η > 0 && ρ > 0 && ϕ > 0 && ξ > 0,
{rX, aX, aY, βY, βZ, δU, δV, rZ, bX, bY, aZ, κ, η, ρ, ϕ, ξ}, Reals]

Reduce[Extract[EP, {2}] ≥ 0 && $-\rho + aZ\ rZ \left(\frac{\eta}{1 - bX\ rZ} + \frac{\beta Z\ \delta U}{-aZ\ bY\ rZ\ \beta Z\ \delta U + aY\ (-1 + bX\ rZ)\ \delta V\ \kappa\ \phi}\right) < 0$ &&
(1 - bX rZ) > 0 && (1 - bX rX) > 0 && aZ > aX > 0 && βY > 0 && βZ > 0 && δU > 0 && δV > 0 &&
rX > rZ > 0 && bX > 0 && bY > 0 && aY > 0 && κ > 0 && η > 0 && ρ > 0 && ϕ > 0 && ξ > 0,
{rX, aX, aY, βY, βZ, δU, δV, rZ, bX, bY, aZ, κ, η, ρ, ϕ, ξ}, Reals]

Out[∘]=
False

Out[∘]=
False

In[∘]:= (*Rearranging $-\rho + aZ\ rZ \left(\frac{\eta}{1 - bX\ rZ} + \frac{\beta Z\ \delta U}{-aZ\ bY\ rZ\ \beta Z\ \delta U + aY\ (-1+bX\ rZ)\ \delta V\ \kappa\ \phi}\right) <$ 0 gives $\frac{\beta Z\ \delta U}{\delta V\ \kappa\ \phi} > \frac{aY\ (-1+bX\ rZ)\ (aZ\ rZ\ \eta - \rho + bX\ rZ\ \rho)}{aZ\ rZ\ (-1+bX\ rZ + aZ\ bY\ rZ\ \eta - bY\ \rho + bX\ bY\ rZ\ \rho)}$ *)

Solve[$-\rho + aZ\ rZ \left(\frac{\eta}{1 - bX\ rZ} + \frac{\beta Z\ \delta U}{-aZ\ bY\ rZ\ \beta Z\ \delta U + aY\ (-1 + bX\ rZ)\ \delta V\ \kappa\ \phi}\right)$ == 0, βZ]

Out[∘]=

$$\left\{\left\{\beta Z \to \frac{aY\ (-1 + bX\ rZ)\ \delta V\ \kappa\ (aZ\ rZ\ \eta - \rho + bX\ rZ\ \rho)\ \phi}{aZ\ rZ\ \delta U\ (-1 + bX\ rZ + aZ\ bY\ rZ\ \eta - bY\ \rho + bX\ bY\ rZ\ \rho)}\right\}\right\}$$

2.2. Parameter regions

In[∘]:=
```
ClearAll["`*"];
```

Mutant invasion condition

```
In[•]:= (*calculating the resident growth rate*)
w = 500; Ω = 10; kX = 0.5; lX = 4; cX = 3; dX = 0.33; bX = 0.01; aX = 1;

rX = (kX * w - lX)/cX * (N[Log[2]]) - dX

Out[•]=
56.5081

In[•]:= (*mutant invasion condition*)
(rZ * aZ)/(1 - rZ * bX) > (rX * aX)/(1 - rX * bX);


(*growth rate vs growth inhibition parameter region for mutant invasion condition*)

p1 = RegionPlot[{(rZ * aZ)/(1 - rZ * bX) > (rX * aX)/(1 - rX * bX), (rZ * aZ)/(1 - rZ * bX) < (rX * aX)/(1 - rX * bX)}, {aZ, aX, 20}, {rZ, 0, rX},
  PlotRange → {{aX - 0.5, 20.5}, {-1.5, rX + 1.5}}, PlotStyle → Directive[Opacity[0.4]],
  PlotLabel → Style["(A) Benefit-cost trade-off", FontFamily → "Arial", Black, 20],
  BoundaryStyle → Directive[Thickness[Small], Dashing[Small]],

  Frame → True,
  FrameLabel → {Style[" Growth inhibition factor, a_Z", FontFamily → "Arial", 18, Black],
    Style["Growth rate, r_Z", FontFamily → "Arial", 18, Black]},
  FrameStyle → Directive[FontFamily → "Arial", Black, 16, Thickness[0.006]],
  FrameTicks → {{All, Automatic}, {All, Automatic}},

  Epilog → {
    {Inset[Framed[
       Style["Mutant invasion condition:\n(r_Z a_Z)/(1 - r_Z b_X) > (r_X a_X)/(1 - r_X b_X)", FontFamily → "Arial", Black, 16],
       RoundingRadius → 10, FrameStyle → Directive[Black, Thickness[0.5]]], {12, 45}]}, {Inset[
      Style["Mutant host invades\n(ectosymbiosis)", FontFamily → "Arial", Black, 16], {13, 24}]},
    {Inset[Style["Mutant host cannot invade", FontFamily → "Arial", Black, 16], {9.5, 4}]},
    {Inset[Style["a_X", FontFamily → "Arial", Black, 18], {1, -3.5}]},
    {Inset[Style["r_X", FontFamily → "Arial", Black, 18], {-0.4, 56.5}]}
   }, PlotRangeClipping → False,

  ImagePadding → {{All, All}, {All, 20}}, ImageSize → {360, 360}, AspectRatio → 1];
```

In[•]:=
```
(*fixing rZ (cost to mutant) and varying aZ (benefit to mutant)*)
w = 500; Ω = 10; kZ = 0.3; lZ = 5; cZ = 4; dZ = 0.4;

rZ = (kZ * w - lZ)/cZ * (N[Log[2]]) - dZ;(*rZ = 24.7*)

(*mutant invasion condition, where s represents surface impact per-
 ectosymbiont and n represents number of ectosymbionts; aZ = aX/(1-n*s) *)
(rZ * aX)/((1 - rZ * bX) * (1 - n * s)) > (rX * aX)/(1 - rX * bX);

p2 = Show[RegionPlot[{(rZ * aX)/((1 - rZ * bX) * (1 - n * s)) > (rX * aX)/(1 - rX * bX) && 0 < 1 - n * s < 1,
    (rZ * aX)/((1 - rZ * bX) * (1 - n * s)) < (rX * aX)/(1 - rX * bX) && 0 < 1 - n * s < 1},
   {s, 0, 1}, {n, 0, 5}, PlotRange → {{-0.03, 1.03}, {-0.15, 5.15}},

    PlotStyle → Directive[Opacity[0.4]],
    PlotLabel → Style["(B) Total surface coverage", FontFamily → "Arial", Black, 20],
    BoundaryStyle → Directive[Thickness[Small], Dashing[Small]], PlotLegends →
     Placed[SwatchLegend[{Style["Consortium\nstabilizes", FontFamily → "Arial", Black, 16]},
       LegendMarkerSize → 15], {0.78, 0.45}],

    Frame → True,
    FrameLabel → {Style["Surface impact per ectosymbiont, s", FontFamily → "Arial", 18, Black],
      Style["Ectosymbiont count, n", FontFamily → "Arial", 18, Black]},
    FrameStyle → Directive[FontFamily → "Arial", Black, 16, Thickness[0.006]]],

   ContourPlot[n * s == 1, {s, 0, 1}, {n, 0, 5}, ContourStyle → Directive[Black, Dashed, Thick],
    PlotLegends → Placed[LineLegend[{Style["n · s = 1", FontFamily → "Arial", 16, Black]},
       LegendLayout → "Column", LegendMarkerSize → {{25, 10}}], {{0.75, 0.58}}]],

   Epilog → {{Inset[Style["Invasion barrier", FontFamily → "Arial", Black, 16], {0.57, 4}]},
     {Inset[Style["Ectosymbiotic consortium\ndoes not stabilize", FontFamily → "Arial",
        Black, 16], {0.4, 0.5}]}, Arrow[{{0.37, 3.8}, {0.25, 3}}]},

   ImagePadding → {{All, All}, {All, 20}}, ImageSize → {360, 360}, AspectRatio → 1];
```

In[•]:=
```
(*checking feasible parameters for mutant invasion*)

(rZ * aX)/((1 - rZ * bX) * (1 - n * s)) > (rX * aX)/(1 - rX * bX) /. {s → 0.4, n → 2}
(rZ * aX)/((1 - rZ * bX) * (1 - n * s)) > (rX * aX)/(1 - rX * bX) /. {s → 0.2, n → 2}
```

Out[•]=
```
True
```

Out[•]=
```
False
```

In[ ]:=
```
(*analyzing shifts in invasion barrier; α introduces non-linear geometry*)
rZ * aX / ((1 - rZ * bX) * (1 - (n * s)^α)) > rX * aX / (1 - rX * bX)
```

Out[ ]=

$$\frac{32.849}{1-(n\,s)^{\alpha}} > 129.928$$

In[ ]:=
```
p3 = ContourPlot[{32.849 == 129.928 * (1 - (n * s)^0.3),
    32.849 == 129.928 * (1 - (n * s)^1), 32.849 == 129.928 * (1 - (n * s)^2), n * s == 1},
   {s, 0, 1}, {n, 0, 5}, PlotRange → {{-0.03, 1.03}, {-0.15, 5.15}}, PlotPoints → 300,

   ContourStyle → {Thick, Thick, Thick, Directive[Black, Dashed, Thick]},
   PlotLabel → Style["(C) Invasion barrier shift", FontFamily → "Arial", Black, 20],

   FrameLabel → {Style["Surface impact per ectosymbiont, s", FontFamily → "Arial", 18, Black],
     Style["Ectosymbiont count, n", FontFamily → "Arial", 18, Black]},
   FrameStyle → Directive[FontFamily → "Arial", Black, 16, Thickness[0.006]],

   PlotLegends → Placed[LineLegend[{Style["α = 0.3", FontFamily → "Arial", 12, Black],
        Style["α = 1.0", FontFamily → "Arial", 12, Black], Style["α = 2.0",
         FontFamily → "Arial", 12, Black], Style["n · s = 1", FontFamily → "Arial", 12, Black]},
      LegendLayout → "Column", LegendMarkerSize → {{25, 10}}], {{0.82, 0.51}}],

   Epilog →
    {Inset[Framed[Style["Inhibition parameter:\n aZ = aX / (1 - (n s)^α)", FontFamily → "Arial", Black, 16],
       RoundingRadius → 10, FrameStyle → Directive[Black, Thickness[0.5]]], {0.7, 4.3}]},

   ImagePadding → {{All, All}, {All, 20}}, ImageSize → {360, 360}, AspectRatio → 1];
```

In[ ]:=
```
(*Fig 4 in main text*)
regplot = GraphicsRow[{p1, p2, p3}, ImageSize → {1100, 400}, Spacings → -10];
```

In[ ]:=
```
SetDirectory[NotebookDirectory[]];
Export["Fig4.tiff", regplot, ImageResolution → 600];


(*analyzing shifts in invasion barrier for bZ ≠ bX*)

w = 500;
Ω = 10;
kX = 0.5;
lX = 4;
cX = 3;
dX = 0.33;
bX = 0.01;
aX = 1;
kZ = 0.3;
lZ = 5;
cZ = 4;
dZ = 0.4;
```

```
rX = ((kX * w - lX) / cX) * (N[Log[2]]) - dX;
rZ = ((kZ * w - lZ) / cZ) * (N[Log[2]]) - dZ;

(rZ * aX) / ((1 - rZ * bZ) * (1 - (n * s))) > (rX * aX) / (1 - rX * bX)
```

Out[∘]=

$$\frac{24.7266}{(1 - 24.7266\,bZ)\,(1 - n\,s)} > 129.928$$

```
In[∘]:= (*Fig A4 in S1 Appendix*)
p4 = ContourPlot[{24.7266 == 129.928 * (1 - 24.7266 * 0.001) * (1 - n * s), 24.7266 ==
      129.928 * (1 - 24.7266 * 0.01) * (1 - n * s), 24.7266 == 129.928 * (1 - 24.7266 * 0.02) * (1 - n * s)},
    {s, 0, 1}, {n, 0, 5}, PlotRange -> {{-0.03, 1.03}, {-0.15, 5.15}}, PlotPoints -> 300,

   ContourStyle -> {Thick, Thick, Thick},

   FrameLabel -> {Style["Surface impact per ectosymbiont, s", FontFamily -> "Arial", 18, Black],
     Style["Ectosymbiont count, n", FontFamily -> "Arial", 18, Black]},
   FrameStyle -> Directive[FontFamily -> "Arial", Black, 16, Thickness[0.006]],

   PlotLegends ->
    Placed[LineLegend[{Style["bZ = 0.001", FontFamily -> "Arial", 12, Black], Style["bZ = 0.01",
         FontFamily -> "Arial", 12, Black], Style["bZ = 0.02", FontFamily -> "Arial", 12, Black]},
       LegendLayout -> "Column", LegendMarkerSize -> {{25, 10}}], {{0.75, 0.45}}],

   Epilog -> {
     {Inset[Framed[
        Style["Mutant invasion condition:\n(rZ aZ)/(1 - rZ bZ) > (rX aX)/(1 - rX bX)", FontFamily -> "Arial", Black, 16],
        RoundingRadius -> 10, FrameStyle -> Directive[Black, Thickness[0.5]]], {0.62, 4.3}]}},

   ImagePadding -> {{All, All}, {All, 20}}, ImageSize -> {340, 340}, AspectRatio -> 1];

In[∘]:= SetDirectory[NotebookDirectory[]];
Export["FigA4.tiff", p4, ImageResolution -> 600];

In[∘]:= ClearAll["`*"];
```

Condition for extinction of free-living phenotypes

In[∘]:=

$$\text{condition2} = -\rho + aZ\,rZ\left(\frac{\eta}{1 - bX\,rZ} + \frac{\beta Z\,\delta U}{-aZ\,bY\,rZ\,\beta Z\,\delta U + aY\,(-1 + bX\,rZ)\,\delta V\,\kappa\,\phi}\right);$$

(*condition for exclusive stability of EM: condition2 < 0,
rearrangement gives $\frac{\beta Z\ \delta U}{\delta V\ \kappa\ \phi} > \frac{aY\ (-1+bX\ rZ)\ (aZ\ rZ\ \eta-\rho+bX\ rZ\ \rho)}{aZ\ rZ\ (-1+bX\ rZ+aZ\ bY\ rZ\ \eta-bY\ \rho+bX\ bY\ rZ\ \rho)}$ *)

```
In[•]:= (*kYZ vs βZ parameter region for extinction of free-living symbionts*)

w = 500; kX = 0.5; cX = 3; lX = 4; dX = 0.3; aX = 1; bX = 0.01;

kY = 0.1;
cY = 0.3;
lY = 1.2;
dY = 0.1;
Ω = 10;
aY = 1.5;
bY = 0.015; δU = 1;
δV = 1;
βY = 2;
lZ = 5;
cZ = 4;
dZ = 0.4;
pZ = 0.2;

n = 2; s = 0.4;

aZ = aX/(1 - n*s);
kZ = kX*(1 - n*pZ);
rX = ((kX*w - lX)/cX)*(N[Log[2]]) - dX;
rZ = ((kZ*w - lZ)/cZ)*(N[Log[2]]) - dZ;
η = (kY/(Ω*cY))*(N[Log[2]]);
ρ = (lY/cY)*(N[Log[2]]) + dY;
ϕ = kX*w;
ϰ = (1 - kYZ*n)*(1 - n*pZ);
ξ = kY/Ω;

RegionPlot[{condition2 < 0, condition2 > 0}, {kYZ, 0, 0.1}, {βZ, 0, 4},

  PlotRange → {{-0.003, 0.103}, {-0.12, 4.12}},
  PlotStyle → Directive[Opacity[0.4]], BoundaryStyle → Directive[Thickness[Small], Dashed],
  PlotLabel → Style["Extinction of free-living type", FontFamily → "Arial", Black, 20],

  Frame → True, FrameLabel →
   {Style["kYZ", FontFamily → "Arial", 18, Black], Style["βZ", FontFamily → "Arial", 18, Black]},
  FrameStyle → Directive[FontFamily → "Arial", Black, 16, Thickness[0.006]],

  Epilog → {{Inset[Style["Free-living phenotype perishes\n(obligate ectosymbiosis)",
        FontFamily → "Arial", Black, 16], {0.052, 3}]}, {Inset[
      Style["Free-living phenotype survives", FontFamily → "Arial", Black, 16], {0.05, 0.35}]}},

  ImagePadding → {{All, All}, {All, 20}}, ImageSize → {360, 360}, AspectRatio → 1];

In[•]:= N[Solve[Rationalize[condition2, 0] == 0]]
Out[•]=
{{βZ → 1.28116 - 2.56232 kYZ}}
```

In[•]:=
```
(*checking feasible parameters for extinction of free-living symbionts*)

condition2 < 0 /. {βZ → 4, kYZ → 0.06}
condition2 < 0 /. {βZ → 0.2, kYZ → 0.05}
```

Out[•]=
```
True
```

Out[•]=
```
False
```

In[•]:=
```
(*ϰ vs βZ parameter region for extinction of free-living symbionts*)

RegionPlot[{condition2 < 0, condition2 > 0}, {ϰ, 0.48, 0.6}, {βZ, 0, 4},

  PlotRange → {{0.477, 0.603}, {-0.12, 4.12}},
  PlotStyle → Directive[Opacity[0.4]], BoundaryStyle → Directive[Thickness[Small], Dashed],
  PlotLabel → Style["Extinction of free-living type", FontFamily → "Arial", Black, 20],

  Frame → True, FrameLabel →
   {Style["ϰ", FontFamily → "Arial", 18, Black], Style["βZ", FontFamily → "Arial", 18, Black]},
  FrameStyle → Directive[FontFamily → "Arial", Black, 16, Thickness[0.006]],

  Epilog → {{Inset[Style["Free-living phenotype perishes\n(obligate ectosymbiosis)",
       FontFamily → "Arial", Black, 16], {0.54, 3}]}, {Inset[
      Style["Free-living phenotype survives", FontFamily → "Arial", Black, 16], {0.54, 0.5}]}},

  ImagePadding → {{All, All}, {All, 20}}, ImageSize → {360, 360}, AspectRatio → 1];
```

2.3. Numerical analysis and figure generation

In[•]:=
```
ClearAll["`*"];
```

Parameters and definitions

```
In[•]:= (*arbitrary parameter values based on the model conditions and parameter explorations*)
paraR = {w → 500, kX → 0.5, cX → 3, lX → 4, dX → 0.33, kY → 0.1, cY → 0.3,
   lY → 1.2, dY → 0.1, Ω → 10, aX → 1, bX → 0.01, aY → 1.5, bY → 0.015, δU → 1, δV → 1,
   βY → 2, lZ → 5, cZ → 4, dZ → 0.4, n → 2, pZ → 0.2, kYZ → 0.05, βZ → 0.2, s → 0.2};

paraP = {w → 500, kX → 0.5, cX → 3, lX → 4, dX → 0.33, kY → 0.1, cY → 0.3,
   lY → 1.2, dY → 0.1, Ω → 10, aX → 1, bX → 0.01, aY → 1.5, bY → 0.015, δU → 1, δV → 1,
   βY → 2, lZ → 5, cZ → 4, dZ → 0.4, n → 2, pZ → 0.2, kYZ → 0.05, βZ → 0.2, s → 0.4};

paraM = {w → 500, kX → 0.5, cX → 3, lX → 4, dX → 0.33, kY → 0.1, cY → 0.3,
   lY → 1.2, dY → 0.1, Ω → 10, aX → 1, bX → 0.01, aY → 1.5, bY → 0.015, δU → 1, δV → 1,
   βY → 2, lZ → 5, cZ → 4, dZ → 0.4, n → 2, pZ → 0.2, kYZ → 0.06, βZ → 4, s → 0.4};

(*substitutions to simplify the system*)
aZ = aX / (1 - n * s);
κ = (1 - kYZ * n) * (1 - n * pZ);
kZ = kX * (1 - n * pZ);
rX = (kX * w - lX) / cX * (N[Log[2]]) - dX;
rZ = (kZ * w - lZ) / cZ * (N[Log[2]]) - dZ;
η = kY / (Ω * cY) * (N[Log[2]]);
ρ = lY / cY * (N[Log[2]]) + dY;
ϕ = kX * w;
ξ = kY / Ω;

(*dynamical system as in Appendix A3 in S1 Appendix*)
dynamics = {
   x'[t] == x[t] * (rX - u[t] / (aX + bX * u[t])),
   y'[t] == y[t] * (η * u[t] - ρ - v[t] / (aY + bY * v[t])),
   z'[t] == z[t] * (rZ - u[t] / (aZ + bX * u[t])),
   u'[t] == ϕ * x[t] + κ * ϕ * z[t] - ξ * u[t] * y[t] - δU * u[t],
   v'[t] == βY * y[t] + βZ * z[t] - δV * v[t]
  };
```

Stability of E_2

In[•]:=
```
(*finding fixed points numerically*)
f1[x_, y_, z_, u_, v_] := x * (rX - u/(aX + bX * u)) /. paraR;
f2[x_, y_, z_, u_, v_] := y * (η * u - ρ - v/(aY + bY * v)) /. paraR;
f3[x_, y_, z_, u_, v_] := z * (rZ - u/(aZ + bX * u)) /. paraR;
f4[x_, y_, z_, u_, v_] := ϕ * x + κ * ϕ * z - ξ * u * y - δU * u /. paraR;
f5[x_, y_, z_, u_, v_] := βY * y + βZ * z - δV * v /. paraR;
Simplify[NSolve[{f1[x, y, z, u, v] == 0, f2[x, y, z, u, v] == 0, f3[x, y, z, u, v] == 0,
   f4[x, y, z, u, v] == 0, f5[x, y, z, u, v] == 0, x ≥ 0, y ≥ 0, z ≥ 0, u ≥ 0, v ≥ 0}, {x, y, z, u, v}]]
```

Out[•]=
```
{{x → 0, z → 0, v → 0, y → 0, u → 0}, {x → 0, z → 0.405544, v → 0.0811087, y → 0, u → 54.7484},
 {x → 0.520216, z → 0, v → 0.194445, y → 0.0972227, u → 129.928},
 {x → 0.519711, z → 0, v → 0, y → 0, u → 129.928}}
```

In[•]:=
```
E0 = {0, 0, 0, 0, 0} (*trivial eq.*)

E1 = {(aX rX δU)/(ϕ - bX rX ϕ), 0, 0, (aX rX)/(1 - bX rX), 0} /. paraR (*one-sp eq.*)

EM = {0, 0, (aZ rZ δU)/(κ ϕ - bX rZ κ ϕ), (aZ rZ)/(1 - bX rZ), (aZ rZ βZ δU)/(δV κ ϕ - bX rZ δV κ ϕ)} /. paraR (*consortium-only eq.*)

E2 = {(aX rX (-δU + (aY δV ξ (aX rX η + (-1 + bX rX) ρ))/(βY (-1 + aX bY rX η - bY ρ + bX (rX + bY rX ρ)))))/((-1 + bX rX) ϕ),
  (aY δV (-aX rX η + ρ - bX rX ρ))/(βY (-1 + aX bY rX η - bY ρ + bX (rX + bY rX ρ))), 0,
  (aX rX)/(1 - bX rX), (aY (-aX rX η + ρ - bX rX ρ))/(-1 + aX bY rX η - bY ρ + bX (rX + bY rX ρ))} /. paraR (*two-sp resident-only eq.*)
```

Out[•]=
```
{0, 0, 0, 0, 0}
```

Out[•]=
```
{0.519711, 0, 0, 129.928, 0}
```

Out[•]=
```
{0, 0, 0.405544, 54.7484, 0.0811087}
```

Out[•]=
```
{0.520216, 0.0972227, 0, 129.928, 0.194445}
```

In[•]:=
```
(*Jacobian matrix at arbitrary (x,y,z,u,v) *)
jacobian[{x_, y_, z_, u_, v_}] =
  FullSimplify[D[{f1[x, y, z, u, v], f2[x, y, z, u, v], f3[x, y, z, u, v],
     f4[x, y, z, u, v], f5[x, y, z, u, v]}, {{x, y, z, u, v}}]];
```

In[•]:=
```
Eigenvalues[jacobian[{x, y, z, u, v} /. {{x, y, z, u, v} → E0}]]
(*unstable*)
```

Out[•]=
```
{56.5081, 24.7266, -2.87259, -1., -1.}
```

In[•]:=
```
Eigenvalues[jacobian[{x, y, z, u, v} /. {{x, y, z, u, v} → E1}]]
(*unstable*)
```

Out[•]=
```
{-19.0799 + 0. i, -0.5 + 4.93219 i, -0.5 - 4.93219 i, -1. + 0. i, 0.129379 + 0. i}
```

In[*]:=
```
Eigenvalues[jacobian[{x, y, z, u, v} /. {{x, y, z, u, v} → EM}]]
(*unstable*)
```
Out[*]=
```
{21.1291 + 0. i, -0.5 + 4.28515 i, -0.5 - 4.28515 i, -1.66166 + 0. i, -1. + 0. i}
```

In[*]:=
```
Eigenvalues[jacobian[{x, y, z, u, v} /. {{x, y, z, u, v} → E2}]]
(*stable*)
```
Out[*]=
```
{-19.0799 + 0. i, -0.500486 + 4.93485 i, -0.500486 - 4.93485 i, -0.847689 + 0. i, -0.152311 + 0. i}
```

Stability of E_P

In[*]:=
```
(*finding fixed points numerically*)
f1[x_, y_, z_, u_, v_] := x * (rX - u/(aX + bX * u)) /. paraP;
f2[x_, y_, z_, u_, v_] := y * (η * u - ρ - v/(aY + bY * v)) /. paraP;
f3[x_, y_, z_, u_, v_] := z * (rZ - u/(aZ + bX * u)) /. paraP;
f4[x_, y_, z_, u_, v_] := ϕ * x + κ * ϕ * z - ξ * u * y - δU * u /. paraP;
f5[x_, y_, z_, u_, v_] := βY * y + βZ * z - δV * v /. paraP;
Simplify[NSolve[{f1[x, y, z, u, v] == 0, f2[x, y, z, u, v] == 0, f3[x, y, z, u, v] == 0,
   f4[x, y, z, u, v] == 0, f5[x, y, z, u, v] == 0, x ≥ 0, y ≥ 0, z ≥ 0, u ≥ 0, v ≥ 0}, {x, y, z, u, v}]]
```
Out[*]=
```
{{x → 0, z → 0, v → 0, y → 0, u → 0}, {x → 0.519711, z → 0, v → 0, y → 0, u → 129.928},
 {x → 0.520216, z → 0, v → 0.194445, y → 0.0972227, u → 129.928},
 {x → 0, z → 1.22368, v → 1.40283, y → 0.579046, u → 164.245},
 {x → 0, z → 1.21663, v → 0.243326, y → 0, u → 164.245}}
```

In[•]:=
```
E0 = {0, 0, 0, 0, 0}
```

$$\text{E1} = \left\{\frac{\text{aX rX }\delta\text{U}}{\phi - \text{bX rX }\phi}, 0, 0, \frac{\text{aX rX}}{1 - \text{bX rX}}, 0\right\} \text{ /. paraP(*one-sp eq.*)}$$

$$\text{EM} = \left\{0, 0, \frac{\text{aZ rZ }\delta\text{U}}{\kappa\,\phi - \text{bX rZ }\kappa\,\phi}, \frac{\text{aZ rZ}}{1 - \text{bX rZ}}, \frac{\text{aZ rZ }\beta\text{Z }\delta\text{U}}{\delta\text{V }\kappa\,\phi - \text{bX rZ }\delta\text{V }\kappa\,\phi}\right\} \text{ /. paraP(*consortium-only eq.*)}$$

$$\text{E2} = \left\{\frac{\text{aX rX}\left(-\delta\text{U} + \frac{\text{aY }\delta\text{V }\xi\ (\text{aX rX }\eta + (-1+\text{bX rX})\ \rho)}{\beta\text{Y }(-1+\text{aX bY rX }\eta - \text{bY }\rho + \text{bX }(\text{rX}+\text{bY rX }\rho))}\right)}{(-1 + \text{bX rX})\ \phi}, \frac{\text{aY }\delta\text{V }(-\text{aX rX }\eta + \rho - \text{bX rX }\rho)}{\beta\text{Y }(-1 + \text{aX bY rX }\eta - \text{bY }\rho + \text{bX }(\text{rX} + \text{bY rX }\rho))}, 0, \frac{\text{aX rX}}{1 - \text{bX rX}}, \frac{\text{aY }(-\text{aX rX }\eta + \rho - \text{bX rX }\rho)}{-1 + \text{aX bY rX }\eta - \text{bY }\rho + \text{bX }(\text{rX} + \text{bY rX }\rho)}\right\} \text{ /. paraP(*two-species resident-only eq.*)}$$

$$\text{EP} = \left\{0, \frac{\text{aZ}^2\text{ bY rZ}^2\ \beta\text{Z }\delta\text{U }\eta - \text{aY }(-1 + \text{bX rZ})^2\ \delta\text{V }\kappa\,\rho\,\phi + \text{aZ rZ }(-1 + \text{bX rZ})\ (\beta\text{Z }(\delta\text{U} + \text{bY }\delta\text{U }\rho) - \text{aY }\delta\text{V }\eta\,\kappa\,\phi)}{(-1 + \text{aZ bY rZ }\eta - \text{bY }\rho + \text{bX }(\text{rZ} + \text{bY rZ }\rho))\ (-\text{aZ rZ }\beta\text{Z }\xi + (-1 + \text{bX rZ})\ \beta\text{Y }\kappa\,\phi)}, -\frac{\text{aZ rZ }(\text{aY }\delta\text{V }\xi\ (-\text{aZ rZ }\eta + \rho - \text{bX rZ }\rho) + \beta\text{Y }\delta\text{U }(-1 + \text{aZ bY rZ }\eta - \text{bY }\rho + \text{bX }(\text{rZ} + \text{bY rZ }\rho)))}{(-1 + \text{aZ bY rZ }\eta - \text{bY }\rho + \text{bX }(\text{rZ} + \text{bY rZ }\rho))\ (-\text{aZ rZ }\beta\text{Z }\xi + (-1 + \text{bX rZ})\ \beta\text{Y }\kappa\,\phi)}, \frac{\text{aZ rZ}}{1 - \text{bX rZ}}, \frac{\text{aY }(-\text{aZ rZ }\eta + \rho - \text{bX rZ }\rho)}{-1 + \text{aZ bY rZ }\eta - \text{bY }\rho + \text{bX }(\text{rZ} + \text{bY rZ }\rho)}\right\} \text{ /. paraP (*mutant eq. with free-living symbionts*)}$$

Out[•]=
```
{0, 0, 0, 0, 0}
```

Out[•]=
```
{0.519711, 0, 0, 129.928, 0}
```

Out[•]=
```
{0, 0, 1.21663, 164.245, 0.243326}
```

Out[•]=
```
{0.520216, 0.0972227, 0, 129.928, 0.194445}
```

Out[•]=
```
{0, 0.579046, 1.22368, 164.245, 1.40283}
```

In[•]:=
```
(*Jacobian matrix at arbitrary (x,y,z,u,v) *)
jacobian[{x_, y_, z_, u_, v_}] =
  FullSimplify[D[{f1[x, y, z, u, v], f2[x, y, z, u, v], f3[x, y, z, u, v],
     f4[x, y, z, u, v], f5[x, y, z, u, v]}, {{x, y, z, u, v}}]];
```

In[•]:=
```
Eigenvalues[jacobian[{x, y, z, u, v} /. {{x, y, z, u, v} → E0}]]
(*unstable*)
```

Out[•]=
```
{56.5081, 24.7266, -2.87259, -1., -1.}
```

In[•]:=
```
Eigenvalues[jacobian[{x, y, z, u, v} /. {{x, y, z, u, v} → E1}]]
(*unstable*)
```

Out[•]=
```
{-0.5 + 4.93219 i, -0.5 - 4.93219 i, 4.10077 + 0. i, -1. + 0. i, 0.129379 + 0. i}
```

In[•]:=
```
Eigenvalues[jacobian[{x, y, z, u, v} /. {{x, y, z, u, v} → E2}]]
(*unstable*)
```

Out[•]=
```
{-0.500486 + 4.93485 i, -0.500486 - 4.93485 i, 4.10077 + 0. i, -0.847689 + 0. i, -0.152311 + 0. i}
```

In[•]:=
```
Eigenvalues[jacobian[{x, y, z, u, v} /. {{x, y, z, u, v} → EM}]]
(*unstable*)
```

Out[•]=
```
{-5.64828 + 0. i, -0.5 + 4.28515 i, -0.5 - 4.28515 i, -1. + 0. i, 0.760456 + 0. i}
```

In[•]:=
```
Eigenvalues[jacobian[{x, y, z, u, v} /. {{x, y, z, u, v} → EP}]]
(*stable*)
```

Out[•]=
```
{-5.64828 + 0. ⅈ, -0.502895 + 4.29993 ⅈ, -0.502895 - 4.29993 ⅈ, -0.5 + 0.707729 ⅈ, -0.5 - 0.707729 ⅈ}
```

Stability of E_M

In[•]:=
```
(*finding fixed points numerically*)
```

$$\text{f1[x\_, y\_, z\_, u\_, v\_] := x} * \left(\text{rX} - \frac{\text{u}}{\text{aX} + \text{bX} * \text{u}}\right) \text{ /. paraM;}$$

$$\text{f2[x\_, y\_, z\_, u\_, v\_] := y} * \left(\eta * \text{u} - \rho - \frac{\text{v}}{\text{aY} + \text{bY} * \text{v}}\right) \text{ /. paraM;}$$

$$\text{f3[x\_, y\_, z\_, u\_, v\_] := z} * \left(\text{rZ} - \frac{\text{u}}{\text{aZ} + \text{bX} * \text{u}}\right) \text{ /. paraM;}$$

```
f4[x_, y_, z_, u_, v_] := ϕ * x + κ * ϕ * z - ξ * u * y - δU * u /. paraM;
f5[x_, y_, z_, u_, v_] := βY * y + βZ * z - δV * v /. paraM;
Simplify[NSolve[{f1[x, y, z, u, v] == 0, f2[x, y, z, u, v] == 0, f3[x, y, z, u, v] == 0,
   f4[x, y, z, u, v] == 0, f5[x, y, z, u, v] == 0, x ≥ 0, y ≥ 0, z ≥ 0, u ≥ 0, v ≥ 0}, {x, y, z, u, v}]]
```

Out[•]=
```
{{x → 0, z → 0, v → 0, y → 0, u → 0}, {x → 0.519711, z → 0, v → 0, y → 0, u → 129.928},
 {x → 0.520216, z → 0, v → 0.194445, y → 0.0972227, u → 129.928},
 {x → 0, z → 1.24428, v → 4.97713, y → 0, u → 164.245}}
```

In[•]:=
```
E0 = {0, 0, 0, 0, 0} (*trivial eq.*)
```

$$\text{E1} = \left\{\frac{\text{aX rX } \delta\text{U}}{\phi - \text{bX rX } \phi}, 0, 0, \frac{\text{aX rX}}{1 - \text{bX rX}}, 0\right\} \text{ /. paraM(*one-sp eq.*)}$$

$$\text{EM} = \left\{0, 0, \frac{\text{aZ rZ } \delta\text{U}}{\kappa\,\phi - \text{bX rZ } \kappa\,\phi}, \frac{\text{aZ rZ}}{1 - \text{bX rZ}}, \frac{\text{aZ rZ } \beta\text{Z } \delta\text{U}}{\delta\text{V } \kappa\,\phi - \text{bX rZ } \delta\text{V } \kappa\,\phi}\right\} \text{ /. paraM(*consortium-only eq.*)}$$

$$\text{E2} = \left\{\frac{\text{aX rX}\left(-\delta\text{U} + \frac{\text{aY } \delta\text{V } \xi\ (\text{aX rX } \eta + (-1 + \text{bX rX})\ \rho)}{\beta\text{Y } (-1 + \text{aX bY rX } \eta - \text{bY } \rho + \text{bX } (\text{rX} + \text{bY rX } \rho))}\right)}{(-1 + \text{bX rX})\ \phi}, \frac{\text{aY } \delta\text{V } (-\text{aX rX } \eta + \rho - \text{bX rX } \rho)}{\beta\text{Y } (-1 + \text{aX bY rX } \eta - \text{bY } \rho + \text{bX } (\text{rX} + \text{bY rX } \rho))}, 0, \frac{\text{aX rX}}{1 - \text{bX rX}}, \frac{\text{aY } (-\text{aX rX } \eta + \rho - \text{bX rX } \rho)}{-1 + \text{aX bY rX } \eta - \text{bY } \rho + \text{bX } (\text{rX} + \text{bY rX } \rho)}\right\} \text{ /. paraM(*two-sp resident-only eq.*)}$$

Out[•]=
```
{0, 0, 0, 0, 0}
```

Out[•]=
```
{0.519711, 0, 0, 129.928, 0}
```

Out[•]=
```
{0, 0, 1.24428, 164.245, 4.97713}
```

Out[•]=
```
{0.520216, 0.0972227, 0, 129.928, 0.194445}
```

In[•]:=
```
(*Jacobian matrix at arbitrary (x,y,z,u,v) *)
jacobian[{x_, y_, z_, u_, v_}] = FullSimplify[D[{f1[x, y, z, u, v], f2[x, y, z, u, v],
     f3[x, y, z, u, v], f4[x, y, z, u, v], f5[x, y, z, u, v]}, {{x, y, z, u, v}}]];
```

In[•]:=
```
Eigenvalues[jacobian[{x, y, z, u, v} /. {{x, y, z, u, v} → E0}]]
(*unstable*)
```

Out[•]=
```
{56.5081, 24.7266, -2.87259, -1., -1.}
```

```
In[•]:= Eigenvalues[jacobian[{x, y, z, u, v} /. {{x, y, z, u, v} → E1}]]
        (*unstable*)
Out[•]=
        {-0.5 + 4.93219 i, -0.5 - 4.93219 i, 4.10077 + 0. i, -1. + 0. i, 0.129379 + 0. i}

In[•]:= Eigenvalues[jacobian[{x, y, z, u, v} /. {{x, y, z, u, v} → E2}]]
        (*unstable*)
Out[•]=
        {-0.500486 + 4.93485 i, -0.500486 - 4.93485 i, 4.10077 + 0. i, -0.847689 + 0. i, -0.152311 + 0. i}

In[•]:= Eigenvalues[jacobian[{x, y, z, u, v} /. {{x, y, z, u, v} → EM}]]
        (*stable*)
Out[•]=
        {-5.64828 + 0. i, -0.5 + 4.28515 i, -0.5 - 4.28515 i, -2.23849 + 0. i, -1. + 0. i}
```

Figure generation

```
In[•]:= (*plotting solutions for the free-living host X, free-living symbiont Y,
        and consortium Z densities as time series plots*)

        DsolR =
          ParametricNDSolveValue[{dynamics /. paraR, x[0] == x0, y[0] == y0, z[0] == z0, u[0] == u0, v[0] == v0},
           {x[t], y[t], z[t]}, {t, 0, 100}, {x0, y0, z0, u0, v0}];(*stable resident-only E2 fixed point*)

        ic0 = DsolR[0.01, 0.01, 0.01, 0.01, 0.01];
        (*arbitrary point in the nbhd of E0 as the initial condition*)
        ic1 = DsolR[0.53, 0.01, 0.01, 131, 0.01];
        (*arbitrary point in the nbhd of E1 as the initial condition*)
        ic2 = DsolR[0.53, 0.1, 0.01, 131, 0.2];
        (*arbitrary point in the nbhd of E2 as the initial condition*)

        spA =
          Plot[Evaluate[Join[ic0, ic1, ic2]], {t, 0, 100}, PlotPoints → 300, ScalingFunctions → "Log",
           PlotStyle → {Directive[RGBColor[0.24, 0.6, 0.8], Thickness[0.008]],
             Directive[RGBColor[0.95, 0.627, 0.1425], Thickness[0.008]],
             Directive[RGBColor[0.455, 0.7, 0.21], Thickness[0.008]]}(*default RGB colors*),

           PlotRange → {{-1, 41}, {0.0001, Automatic}}, Frame → True,
           FrameTicks → {{All, None}, {All, None}}, FrameStyle → Directive[Black, Thickness[0.006], 18],

           Epilog → {Inset[Style["Free-living host, x", FontFamily → "Arial", Black, 21], {29.5, 0.1}],
             Inset[Style["Free-living symbiont, y", FontFamily → "Arial", Black, 21], {26.7, -3.8}]},

           AspectRatio → 0.75, ImagePadding → {{50, 15}, {25, 10}}, ImageSize → {400, 300}];
```

In[ ]:=

```
DsolP =
  ParametricNDSolveValue[{dynamics /. paraP, x[0] == x0, y[0] == y0, z[0] == z0, u[0] == u0, v[0] == v0},
   {x[t], y[t], z[t]}, {t, 0, 100}, {x0, y0, z0, u0, v0}];(*stable EP fixed point*)

ic0 = DsolP[0.01, 0.01, 0.01, 0.01, 0.01];
ic1 = DsolP[0.53, 0.01, 0.01, 131, 0.01];
ic2 = DsolP[0.53, 0.1, 0.01, 131, 0.2];

spB =
  Plot[Evaluate[Join[ic0, ic1, ic2]], {t, 0, 100}, PlotPoints → 300, ScalingFunctions → "Log",
   PlotStyle → {Directive[RGBColor[0.24, 0.6, 0.8], Thickness[0.008]],
     Directive[RGBColor[0.95, 0.627, 0.1425], Thickness[0.008]],
     Directive[RGBColor[0.455, 0.7, 0.21], Thickness[0.008]]},

   PlotRange → {{-1, 41}, {0.0001, Automatic}}, Frame → True,
   FrameTicks → {{All, None}, {All, None}}, FrameStyle → Directive[Black, Thickness[0.006], 18],

   Epilog → {Inset[Style["Consortium, z", FontFamily → "Arial", Black, 21], {32.1, 1}],
     Inset[Style["Free-living symbiont, y", FontFamily → "Arial", Black, 21], {26.6, -1.5}]},

   AspectRatio → 0.75, ImagePadding → {{50, 15}, {25, 10}}, ImageSize → {400, 300}];
```

In[ ]:=

```
DsolM =
  ParametricNDSolveValue[{dynamics /. paraM, x[0] == x0, y[0] == y0, z[0] == z0, u[0] == u0, v[0] == v0},
   {x[t], y[t], z[t]}, {t, 0, 100}, {x0, y0, z0, u0, v0}];(*stable mutant-only EM fixed point*)

ic0 = DsolM[0.01, 0.01, 0.01, 0.01, 0.01];
ic1 = DsolM[0.53, 0.01, 0.01, 131, 0.01];
ic2 = DsolM[0.53, 0.1, 0.01, 131, 0.2];

spC =
  Plot[Evaluate[Join[ic0, ic1, ic2]], {t, 0, 100}, PlotPoints → 300, ScalingFunctions → "Log",
   PlotStyle → {Directive[RGBColor[0.24, 0.6, 0.8], Thickness[0.008]],
     Directive[RGBColor[0.95, 0.627, 0.1425], Thickness[0.008]],
     Directive[RGBColor[0.455, 0.7, 0.21], Thickness[0.008]]},

   PlotRange → {{-1, 41}, {0.0001, Automatic}}, Frame → True,
   FrameTicks → {{All, None}, {All, None}}, FrameStyle → Directive[Black, Thickness[0.006], 18],

   Epilog → {Inset[Style["Consortium, z", FontFamily → "Arial", Black, 21], {32, 1}]},

   AspectRatio → 0.75, ImagePadding → {{50, 15}, {25, 10}}, ImageSize → {400, 300}];
```

```
In[•]:= (* plotting solutions for the host and symbiont
         metabolite (U and V) concentrations as time series plots*)
        DsolR =
          ParametricNDSolveValue[{dynamics /. paraR, x[0] == x0, y[0] == y0, z[0] == z0, u[0] == u0, v[0] == v0},
           {u[t], v[t]}, {t, 0, 100}, {x0, y0, z0, u0, v0}];(*stable E2*)

        ic0 = DsolR[0.01, 0.01, 0.01, 0.01, 0.01];
        ic1 = DsolR[0.53, 0.01, 0.01, 131, 0.01];
        ic2 = DsolR[0.53, 0.1, 0.01, 131, 0.2];

        mcA =
          Plot[Evaluate[Join[ic0, ic1, ic2]], {t, 0, 100}, ScalingFunctions → "Log",
           PlotStyle → {Directive[RGBColor[0.5, 0.2, 0.9], Thickness[0.008]],
             Directive[RGBColor[0.6, 0.2, 0.3], Thickness[0.008]]}, PlotPoints → 300,

           PlotRange → {{-1, 41}, {0.001, Automatic}}, Frame → True,
           FrameTicks → {{All, None}, {All, None}}, FrameStyle → Directive[Black, Thickness[0.006], 18],

           Epilog → {Inset[Style["Host metabolite, u", FontFamily → "Arial", Black, 21], {29.5, 4}],
             Inset[Style["Symbiont metabolite, v", FontFamily → "Arial", Black, 21], {27, -0.7}]},

           ImagePadding → {{50, 15}, {25, 10}}, ImageSize → {400, 300}, AspectRatio → 0.75];

In[•]:=
        DsolP =
          ParametricNDSolveValue[{dynamics /. paraP, x[0] == x0, y[0] == y0, z[0] == z0, u[0] == u0, v[0] == v0},
           {u[t], v[t]}, {t, 0, 100}, {x0, y0, z0, u0, v0}];(*stable EP*)

        ic0 = DsolP[0.01, 0.01, 0.01, 0.01, 0.01];
        ic1 = DsolP[0.53, 0.01, 0.01, 131, 0.01];
        ic2 = DsolP[0.53, 0.1, 0.01, 131, 0.2];

        mcB =
          Plot[Evaluate[Join[ic0, ic1, ic2]], {t, 0, 100}, ScalingFunctions → "Log",
           PlotStyle → {Directive[RGBColor[0.5, 0.2, 0.9], Thickness[0.008]],
             Directive[RGBColor[0.6, 0.2, 0.3], Thickness[0.008]]}, PlotPoints → 300,

           PlotRange → {{-1, 41}, {0.001, Automatic}}, Frame → True,
           FrameTicks → {{All, None}, {All, None}}, FrameStyle → Directive[Black, Thickness[0.006], 18],

           ImagePadding → {{50, 15}, {25, 10}}, ImageSize → {400, 300}, AspectRatio → 0.75];
```

```
In[•]:= DsolM =
          ParametricNDSolveValue[{dynamics /. paraM, x[0] == x0, y[0] == y0, z[0] == z0, u[0] == u0, v[0] == v0},
           {u[t], v[t]}, {t, 0, 100}, {x0, y0, z0, u0, v0}];(*stable EM*)

        ic0 = DsolM[0.01, 0.01, 0.01, 0.01, 0.01];
        ic1 = DsolM[0.53, 0.01, 0.01, 131, 0.01];
        ic2 = DsolM[0.53, 0.1, 0.01, 131, 0.2];

        mcC =
          Plot[Evaluate[Join[ic0, ic1, ic2]], {t, 0, 100}, ScalingFunctions → "Log",
           PlotStyle → {Directive[RGBColor[0.5, 0.2, 0.9], Thickness[0.008]],
             Directive[RGBColor[0.6, 0.2, 0.3], Thickness[0.008]]}, PlotPoints → 300,
           PlotRange → {{-1, 41}, {0.001, Automatic}}, Frame → True,
           FrameTicks → {{All, None}, {All, None}}, FrameStyle → Directive[Black, Thickness[0.006], 18],

           ImagePadding → {{50, 15}, {25, 10}}, ImageSize → {400, 300}, AspectRatio → 0.75];

In[•]:= (*Fig 3 in the main text*)
        grid = Pane[Grid[
            MapThread[Prepend,
             {
              Append[
               Prepend[{{spA, spB, spC}, {mcA, mcB, mcC}},
                {Style["(A) Resident system resists invasion", FontFamily → "Arial", 24, Black],
                 Style["(B) Invasion, facultative symbionts", FontFamily → "Arial", 24, Black],
                 Style["(C) Invasion, obligate ectosymbiosis", FontFamily → "Arial", 24, Black]}],

               {Style["Time", FontFamily → "Arial", 24, Black], Style["Time",
                 FontFamily → "Arial", 24, Black], Style["Time", FontFamily → "Arial", 24, Black]}],

              {"", Rotate[Style["Population densities", FontFamily → "Arial", 24, Black], 90 Degree],
               Rotate[Style["Metabolite concentrations", FontFamily → "Arial", 24, Black], 90 Degree], ""}

             }], Spacings → {1, 0}], ImageMargins → {{15, 5}, {10, 10}}];

In[•]:= SetDirectory[NotebookDirectory[]];
        Export["Fig3.tiff", grid, ImageResolution → 600];
```

2.4. Finite symbiont pool

```
In[•]:= ClearAll["`*"];

In[•]:= (*assume independent existence of symbionts and a constant symbiont density, y = 1, y'[t] = 0*)
```

$$f1[x\_, z\_, u\_, v\_] := x * \left(rX - \frac{u}{aX + bX * u}\right)$$

$$f2[x\_, z\_, u\_, v\_] := z * \left(rZ - \frac{u}{aZ + bX * u}\right);$$

$$f3[x\_, z\_, u\_, v\_] := \phi * x + \kappa * \phi * z - \xi * u - \delta U * u;$$

$$f4[x\_, z\_, u\_, v\_] := \beta Y + \beta Z * z - \delta V * v;$$

```
Simplify[Solve[
   {f1[x, z, u, v] == 0 && f2[x, z, u, v] == 0 && f3[x, z, u, v] == 0 && f4[x, z, u, v] == 0}, {x, z, u, v}]]
```

Out[•]=

$$\left\{\left\{x \to \frac{aX\, rX\, (\delta U + \xi)}{\phi - bX\, rX\, \phi},\ z \to 0,\ u \to \frac{aX\, rX}{1 - bX\, rX},\ v \to \frac{\beta Y}{\delta V}\right\},\right.$$

$$\left\{x \to 0,\ z \to \frac{aZ\, rZ\, \delta U + aZ\, rZ\, \xi}{\kappa\, \phi - bX\, rZ\, \kappa\, \phi},\ u \to \frac{aZ\, rZ}{1 - bX\, rZ},\ v \to \frac{\beta Y + \frac{aZ\, rZ\, \beta Z\, (\delta U + \xi)}{(1 - bX\, rZ)\, \kappa\, \phi}}{\delta V}\right\},\ \left.\left\{x \to 0,\ z \to 0,\ u \to 0,\ v \to \frac{\beta Y}{\delta V}\right\}\right\}$$

In[•]:=
```
(*fixed points*)
```
$$E0 = \left\{0, 0, 0, \frac{\beta Y}{\delta V}\right\};$$

$$E2 = \left\{\frac{aX\, rX\, (\delta U + \xi)}{\phi - bX\, rX\, \phi}, 0, \frac{aX\, rX}{1 - bX\, rX}, \frac{\beta Y}{\delta V}\right\};$$

$$EM = \left\{0, \frac{aZ\, rZ\, \delta U + aZ\, rZ\, \xi}{\kappa\phi - bX\, rZ\, \kappa\phi}, \frac{aZ\, rZ}{1 - bX\, rZ}, \frac{\beta Y + \frac{aZ\, rZ\, \beta Z\, (\delta U+\xi)}{(1-bX\, rZ)\, \kappa\phi}}{\delta V}\right\};$$

In[•]:=
```
(*Jacobian matrix at arbitrary (x,z,u,v) *)
jacobian[{x_, z_, u_, v_}] =
  FullSimplify[D[{f1[x, z, u, v], f2[x, z, u, v], f3[x, z, u, v], f4[x, z, u, v]}, {{x, z, u, v}}]];
```

In[•]:=
```
Eigenvalues[jacobian[{x, z, u, v} /. {{x, z, u, v} → E0}]]
(*unstable*)
```

Out[•]=
$$\{rX, rZ, -\delta V, -\delta U - \xi\}$$

In[•]:=
```
FullSimplify[Eigenvalues[jacobian[{x, z, u, v} /. {{x, z, u, v} → E2}]]]
```
(*Stability condition of E2 is $rZ - \frac{aX\ rX}{aZ+aX\ bX\ rX-aZ\ bX\ rX} < 0$, i.e., $\frac{aZ\ rZ}{1-rZ\ bX} < \frac{aX\ rX}{1-bX\ rX}$ *)

Out[•]=
$$\left\{-\frac{aX\, rX}{aZ + aX\, bX\, rX - aZ\, bX\, rX} + rZ, -\delta V, \frac{1}{2}\left(-\delta U - \xi + \sqrt{\delta U + \xi}\ \sqrt{4\, rX\, (-1 + bX\, rX) + \delta U + \xi}\right), \frac{1}{2}\left(-\delta U - \xi - \sqrt{\delta U + \xi}\ \sqrt{4\, rX\, (-1 + bX\, rX) + \delta U + \xi}\right)\right\}$$

In[•]:=
```
FullSimplify[Eigenvalues[jacobian[{x, z, u, v} /. {{x, z, u, v} → EM}]]]
```
(*Stability condition of EM is $\frac{aZ\ (-1+bX\ rX)\ rZ+aX\ (rX-bX\ rX\ rZ)}{aX-aX\ bX\ rZ+aZ\ bX\ rZ} < 0$, i.e., $\frac{aZ\ rZ}{1-rZ\ bX} > \frac{aX\ rX}{1-bX\ rX}$ *)

Out[•]=
$$\left\{\frac{aZ\, (-1 + bX\, rX)\, rZ + aX\, (rX - bX\, rX\, rZ)}{aX - aX\, bX\, rZ + aZ\, bX\, rZ}, -\delta V, \frac{1}{2}\left(-\delta U - \xi + \sqrt{\delta U + \xi}\ \sqrt{4\, rZ\, (-1 + bX\, rZ) + \delta U + \xi}\right), \frac{1}{2}\left(-\delta U - \xi - \sqrt{\delta U + \xi}\ \sqrt{4\, rZ\, (-1 + bX\, rZ) + \delta U + \xi}\right)\right\}$$

```
In[]:= (*arbitrary parameter values based on model conditions*)

paraR = {w → 500, kX → 0.5, cX → 3, lX → 4, dX → 0.33, kY → 0.1, cY → 0.3,
   lY → 1.2, dY → 0.1, Ω → 10, aX → 1, bX → 0.01, aY → 1.5, bY → 0.015, δU → 1, δV → 1,
   βY → 2, lZ → 5, cZ → 4, dZ → 0.4, n → 2, pZ → 0.2, kYZ → 0.05, βZ → 0.2, s → 0.2};

paraP = {w → 500, kX → 0.5, cX → 3, lX → 4, dX → 0.33, kY → 0.1, cY → 0.3,
   lY → 1.2, dY → 0.1, Ω → 10, aX → 1, bX → 0.01, aY → 1.5, bY → 0.015, δU → 1, δV → 1,
   βY → 2, lZ → 5, cZ → 4, dZ → 0.4, n → 2, pZ → 0.2, kYZ → 0.05, βZ → 0.2, s → 0.4};

paraM = {w → 500, kX → 0.5, cX → 3, lX → 4, dX → 0.33, kY → 0.1, cY → 0.3,
   lY → 1.2, dY → 0.1, Ω → 10, aX → 1, bX → 0.01, aY → 1.5, bY → 0.015, δU → 1, δV → 1,
   βY → 2, lZ → 5, cZ → 4, dZ → 0.4, n → 2, pZ → 0.2, kYZ → 0.06, βZ → 4, s → 0.4};


(*substitutions to simplify the system*)
aZ = aX / (1 - n * s);
κ = (1 - kYZ * n) * (1 - n * pZ);
kZ = kX * (1 - n * pZ);
rX = (kX * w - lX) / cX * (N[Log[2]]) - dX;
rZ = (kZ * w - lZ) / cZ * (N[Log[2]]) - dZ;
η = kY / (Ω * cY) * (N[Log[2]]);
ρ = lY / cY * (N[Log[2]]) + dY;
ϕ = kX * w;
ξ = kY / Ω;

(*new dynamical system with y = 1 and y'[t] = 0*)
dynamics = {
   x'[t] == x[t] * (rX - u[t] / (aX + bX * u[t])),
   z'[t] == z[t] * (rZ - u[t] / (aZ + bX * u[t])),
   u'[t] == ϕ * x[t] + κ * ϕ * z[t] - ξ * u[t] - δU * u[t],
   v'[t] == βY + βZ * z[t] - δV * v[t]
  };

In[]:= (*finding fixed points numerically for paraR*)
f1[x_, z_, u_, v_] := x * (rX - u / (aX + bX * u)) /. paraR;
f2[x_, z_, u_, v_] := z * (rZ - u / (aZ + bX * u)) /. paraR;
f3[x_, z_, u_, v_] := ϕ * x + κ * ϕ * z - ξ * u - δU * u /. paraR;
f4[x_, z_, u_, v_] := βY + βZ * z - δV * v /. paraR;
Simplify[NSolve[{f1[x, z, u, v] == 0, f2[x, z, u, v] == 0,
   f3[x, z, u, v] == 0, f4[x, z, u, v] == 0, x ≥ 0, z ≥ 0, u ≥ 0, v ≥ 0}, {x, z, u, v}]]

Out[]=
{{x → 0, z → 0, u → 0, v → 2.}, {x → 0.524908, z → 0, u → 129.928, v → 2.},
 {x → 0, z → 0.409599, u → 54.7484, v → 2.08192}}
```

```
In[]:= E0 = {0, 0, 0, 2};

E2 = {0.524908, 0, 129.928, 2};

EM = {0, 0.409599, 54.7484, 2.08192};

In[]:= (*Jacobian matrix at arbitrary (x,z,u,v) *)
jacobian[{x_, z_, u_, v_}] =
  FullSimplify[D[{f1[x, z, u, v], f2[x, z, u, v], f3[x, z, u, v], f4[x, z, u, v]}, {{x, z, u, v}}]];

In[]:= Eigenvalues[jacobian[{x, z, u, v} /. {{x, z, u, v} → E0}]]
(*unstable*)
Out[]=
{56.5081, 24.7266, -1.01, -1.}

In[]:= Eigenvalues[jacobian[{x, z, u, v} /. {{x, z, u, v} → EM}]]
(*unstable*)
Out[]=
{21.1291 + 0. i, -0.505003 + 4.30623 i, -0.505003 - 4.30623 i, -1. + 0. i}

In[]:= Eigenvalues[jacobian[{x, z, u, v} /. {{x, z, u, v} → E2}]]
(*stable*)
Out[]=
{-19.08 + 0. i, -0.505028 + 4.95653 i, -0.505028 - 4.95653 i, -1. + 0. i}

In[]:= (*plotting the corresponding solutions of x and z*)

DsolR = ParametricNDSolveValue[{dynamics /. paraR, x[0] == x0, z[0] == z0, u[0] == u0, v[0] == v0},
   {x[t], z[t]}, {t, 0, 100}, {x0, z0, u0, v0}];

ic0 = DsolR[0.01, 0.01, 0.01, 2.01];
ic1 = DsolR[0.53, 0.01, 131, 2.01];

fig1 =
  Plot[Evaluate[Join[ic0, ic1]], {t, 0, 100}, ScalingFunctions → "Log",
   PlotStyle → {Directive[RGBColor[0.24, 0.6, 0.8], Thickness[0.008]],
     Directive[RGBColor[0.455, 0.7, 0.21], Thickness[0.008]]}, PlotPoints → 300,

   PlotRange → {{-1.2, 41.2}, {0.0001, All}}, Frame → True, FrameStyle →
    Directive[Black, Thickness[0.006], 18], FrameTicks → {{Automatic, None}, {All, None}},

   Epilog →
    {Inset[Text[Style["Free-living host, x", FontFamily → "Arial", Black, 21]], {30, -1.4}]},

   ImageSize → {400, 300}, ImagePadding → {{50, 15}, {20, 10}}, AspectRatio → 0.75];

In[]:= (*finding fixed points numerically for paraP*)
f1[x_, z_, u_, v_] := x * (rX - u/(aX + bX * u)) /. paraP;
f2[x_, z_, u_, v_] := z * (rZ - u/(aZ + bX * u)) /. paraP;
f3[x_, z_, u_, v_] := ϕ * x + κ * ϕ * z - ξ * u - δU * u /. paraP;
f4[x_, z_, u_, v_] := βY + βZ * z - δV * v /. paraP;
Simplify[NSolve[{f1[x, z, u, v] == 0, f2[x, z, u, v] == 0,
   f3[x, z, u, v] == 0, f4[x, z, u, v] == 0, x ≥ 0, z ≥ 0, u ≥ 0, v ≥ 0}, {x, z, u, v}]]
Out[]=
{{x → 0, z → 0, u → 0, v → 2.}, {x → 0, z → 1.2288, u → 164.245, v → 2.24576},
 {x → 0.524908, z → 0, u → 129.928, v → 2.}}
```

```
In[•]:= E0 = {0, 0, 0, 2};

        E2 = {0.524908, 0, 129.928, 2};

        EM = {0, 1.2288, 164.245, 2.24576};

In[•]:= (*Jacobian matrix at arbitrary (x,z,u,v) *)
        jacobian[{x_, z_, u_, v_}] =
          FullSimplify[D[{f1[x, z, u, v], f2[x, z, u, v], f3[x, z, u, v], f4[x, z, u, v]}, {{x, z, u, v}}]];

In[•]:= Eigenvalues[jacobian[{x, z, u, v} /. {{x, z, u, v} → E0}]]
        (*unstable*)
Out[•]=
        {56.5081, 24.7266, -1.01, -1.}

In[•]:= Eigenvalues[jacobian[{x, z, u, v} /. {{x, z, u, v} → E2}]]
        (*unstable*)
Out[•]=
        {-0.505028 + 4.95653 i, -0.505028 - 4.95653 i, 4.10074 + 0. i, -1. + 0. i}

In[•]:= Eigenvalues[jacobian[{x, z, u, v} /. {{x, z, u, v} → EM}]]
        (*stable*)
Out[•]=
        {-5.64826 + 0. i, -0.504992 + 4.30624 i, -0.504992 - 4.30624 i, -1. + 0. i}

In[•]:= (*plotting the corresponding solutions of x and z*)

        DsolP = ParametricNDSolveValue[{dynamics /. paraP, x[0] == x0, z[0] == z0, u[0] == u0, v[0] == v0},
           {x[t], z[t]}, {t, 0, 100}, {x0, z0, u0, v0}];

        ic0 = DsolP[0.01, 0.01, 0.01, 2.01];
        ic1 = DsolP[0.53, 0.01, 131, 2.01];

        fig2 =
          Plot[Evaluate[Join[ic0, ic1]], {t, 0, 100}, ScalingFunctions → "Log",
           PlotStyle → {Directive[RGBColor[0.24, 0.6, 0.8], Thickness[0.008]],
             Directive[RGBColor[0.455, 0.7, 0.21], Thickness[0.008]]}, PlotPoints → 300,

           PlotRange → {{-1.2, 41.2}, {0.0001, All}}, Frame → True, FrameStyle →
            Directive[Black, Thickness[0.006], 18], FrameTicks → {{Automatic, None}, {All, None}},

           Epilog → {Inset[Text[Style["Consortium, z", FontFamily → "Arial", Black, 21]], {32, -0.4}]},

           ImageSize → {400, 300}, ImagePadding → {{50, 15}, {20, 10}}, AspectRatio → 0.75];

In[•]:= (*finding fixed points numerically for paraM*)
        f1[x_, z_, u_, v_] := x * (rX - u/(aX + bX * u)) /. paraM;
        f2[x_, z_, u_, v_] := z * (rZ - u/(aZ + bX * u)) /. paraM;
        f3[x_, z_, u_, v_] := ϕ * x + κ * ϕ * z - ξ * u - δU * u /. paraM;
        f4[x_, z_, u_, v_] := βY + βZ * z - δV * v /. paraM;
        Simplify[NSolve[{f1[x, z, u, v] == 0, f2[x, z, u, v] == 0,
           f3[x, z, u, v] == 0, f4[x, z, u, v] == 0, x ≥ 0, z ≥ 0, u ≥ 0, v ≥ 0}, {x, z, u, v}]]
Out[•]=
        {{x → 0, z → 0, u → 0, v → 2.}, {x → 0, z → 1.25672, u → 164.245, v → 7.0269},
         {x → 0.524908, z → 0, u → 129.928, v → 2.}}
```

```
In[*]:= E0 = {0, 0, 0, 2};

        E2 = {0.524908, 0, 129.928, 2};

        EM = {0, 1.25672, 164.245, 7.0269};

In[*]:= (*Jacobian matrix at arbitrary (x,z,u,v) *)
        jacobian[{x_, z_, u_, v_}] =
          FullSimplify[D[{f1[x, z, u, v], f2[x, z, u, v], f3[x, z, u, v], f4[x, z, u, v]}, {{x, z, u, v}}]];

In[*]:= Eigenvalues[jacobian[{x, z, u, v} /. {{x, z, u, v} → E0}]]
        (*unstable*)

Out[*]=
        {56.5081, 24.7266, -1.01, -1.}

In[*]:= Eigenvalues[jacobian[{x, z, u, v} /. {{x, z, u, v} → E2}]]
        (*unstable*)

Out[*]=
        {-0.505028 + 4.95653 i, -0.505028 - 4.95653 i, 4.10074 + 0. i, -1. + 0. i}

In[*]:= Eigenvalues[jacobian[{x, z, u, v} /. {{x, z, u, v} → EM}]]
        (*stable*)

Out[*]=
        {-5.64826 + 0. i, -0.504992 + 4.30623 i, -0.504992 - 4.30623 i, -1. + 0. i}

In[*]:= (*plotting the corresponding solutions of x and z*)

        DsolM = ParametricNDSolveValue[{dynamics /. paraM, x[0] == x0, z[0] == z0, u[0] == u0, v[0] == v0},
           {x[t], z[t]}, {t, 0, 100}, {x0, z0, u0, v0}];

        ic0 = DsolM[0.01, 0.01, 0.01, 2.01];
        ic1 = DsolM[0.53, 0.01, 131, 2.01];

        fig3 =
          Plot[Evaluate[Join[ic0, ic1]], {t, 0, 100}, ScalingFunctions → "Log",
            PlotStyle → {Directive[RGBColor[0.24, 0.6, 0.8], Thickness[0.008]],
              Directive[RGBColor[0.455, 0.7, 0.21], Thickness[0.008]]}, PlotPoints → 300,

            PlotRange → {{-1.2, 41.2}, {0.0001, All}}, Frame → True, FrameStyle →
             Directive[Black, Thickness[0.006], 18], FrameTicks → {{Automatic, None}, {All, None}},

            Epilog → {Inset[Text[Style["Consortium, z", FontFamily → "Arial", Black, 21]], {32, -0.4}]},

            ImageSize → {400, 300}, ImagePadding → {{50, 15}, {20, 10}}, AspectRatio → 0.75];
```

```
In[•]:= Pane[Grid[
          MapThread[Prepend,
           {
            Append[
             Prepend[{{fig1, fig2, fig3}}, {Style["Stable resident (for y = 1)", FontFamily → "Arial", 24,
                 Black], Style["Stable mutant (for y = 1)", FontFamily → "Arial", 24, Black],
               Style["Stable mutant (for y = 1)", FontFamily → "Arial", 24, Black]}],

             {Style["Time", FontFamily → "Arial", 24, Black], Style["Time",
               FontFamily → "Arial", 24, Black], Style["Time", FontFamily → "Arial", 24, Black]}],

            {"", Rotate[Style["Population densities", FontFamily → "Arial", 24, Black], 90 Degree], ""}

           }], Spacings → {1, 0}], ImageMargins → {{15, 5}, {10, 10}}];
        (*NOTE: The results are qualitatively similar
          even if we assume a finite symbiont supply in our model.*)
```

2.5. Sensitivity to dilution factor

```
In[•]:= ClearAll["`*"];
```

In[•]:=
```
(*arbitrary parameter values except Omega*)
para = {w → 500, kX → 0.5, cX → 3, lX → 4, dX → 0.33, kY → 0.1, cY → 0.3,
   lY → 1.2, dY → 0.1, aX → 1, bX → 0.01, aY → 1.5, bY → 0.015, δU → 1, δV → 1,
   βY → 2, lZ → 5, cZ → 4, dZ → 0.4, n → 2, pZ → 0.2, kYZ → 0.06, βZ → 4, s → 0.4};


(*substitutions to simplify the system*)
aZ = aX / (1 - n * s);
κ = (1 - kYZ * n) * (1 - n * pZ);
kZ = kX * (1 - n * pZ);
rX = (kX * w - lX) / cX * (N[Log[2]]) - dX;
rZ = (kZ * w - lZ) / cZ * (N[Log[2]]) - dZ;
η = kY / (Ω * cY) * (N[Log[2]]);
ρ = lY / cY * (N[Log[2]]) + dY;
ϕ = kX * w;
ξ = kY / Ω;


(*dynamical system*)
dynamics = {
   x'[t] == x[t] * (rX - u[t] / (aX + bX * u[t])),
   y'[t] == y[t] * (η * u[t] - ρ - v[t] / (aY + bY * v[t])),
   z'[t] == z[t] * (rZ - u[t] / (aZ + bX * u[t])),
   u'[t] == ϕ * x[t] + κ * ϕ * z[t] - ξ * u[t] * y[t] - δU * u[t],
   v'[t] == βY * y[t] + βZ * z[t] - δV * v[t]
  };

(*finding critical value of Omega *)
N[Reduce[Rationalize[
   {1 - bX * rX > 0 && 1 - bX * rZ > 0 && -ρ + aZ rZ (η / (1 - bX rZ) + βZ δU / (-aZ bY rZ βZ δU + aY (-1 + bX rZ) δV κ ϕ)) < 0 &&
     rZ * aZ / (1 - rZ * bX) > rX * aX / (1 - rX * bX)} /. para, 0], {Ω}, PositiveReals]]

N[Reduce[Rationalize[
   {1 - bX * rX > 0 && 1 - bX * rZ > 0 && -ρ + aZ rZ (η / (1 - bX rZ) + βZ δU / (-aZ bY rZ βZ δU + aY (-1 + bX rZ) δV κ ϕ)) < 0 &&
     (ρ / η + 1 / (η * bY)) > rZ * aZ / (1 - rZ * bX) > rX * aX / (1 - rX * bX) > ρ / η} /. para, 0], {Ω}, PositiveReals]]
```

Out[•]=
```
Ω > 6.28981
```

Out[•]=
```
6.28981 < Ω < 10.4504
```

```
In[•]:= (*arbitrary parameter values with varied Omega*)
para1 = {w → 500, kX → 0.5, cX → 3, lX → 4, dX → 0.33, kY → 0.1, cY → 0.3,
   lY → 1.2, dY → 0.1, Ω → 1, aX → 1, bX → 0.01, aY → 1.5, bY → 0.015, δU → 1, δV → 1,
   βY → 2, lZ → 5, cZ → 4, dZ → 0.4, n → 2, pZ → 0.2, kYZ → 0.06, βZ → 4, s → 0.4};

para2 = {w → 500, kX → 0.5, cX → 3, lX → 4, dX → 0.33, kY → 0.1, cY → 0.3,
   lY → 1.2, dY → 0.1, Ω → 6, aX → 1, bX → 0.01, aY → 1.5, bY → 0.015, δU → 1, δV → 1,
   βY → 2, lZ → 5, cZ → 4, dZ → 0.4, n → 2, pZ → 0.2, kYZ → 0.06, βZ → 4, s → 0.4};

para3 = {w → 500, kX → 0.5, cX → 3, lX → 4, dX → 0.33, kY → 0.1, cY → 0.3,
   lY → 1.2, dY → 0.1, Ω → 10, aX → 1, bX → 0.01, aY → 1.5, bY → 0.015, δU → 1, δV → 1,
   βY → 2, lZ → 5, cZ → 4, dZ → 0.4, n → 2, pZ → 0.2, kYZ → 0.06, βZ → 4, s → 0.4};



(*substitutions to simplify the system*)
aZ = aX / (1 - n * s);
κ = (1 - kYZ * n) * (1 - n * pZ);
kZ = kX * (1 - n * pZ);
rX = (kX * w - lX) / cX * (N[Log[2]]) - dX;
rZ = (kZ * w - lZ) / cZ * (N[Log[2]]) - dZ;
η = kY / (Ω * cY) * (N[Log[2]]);
ρ = lY / cY * (N[Log[2]]) + dY;
ϕ = kX * w;
ξ = kY / Ω;

(*dynamical system*)
dynamics = {
   x'[t] == x[t] * (rX - u[t] / (aX + bX * u[t])),
   y'[t] == y[t] * (η * u[t] - ρ - v[t] / (aY + bY * v[t])),
   z'[t] == z[t] * (rZ - u[t] / (aZ + bX * u[t])),
   u'[t] == ϕ * x[t] + κ * ϕ * z[t] - ξ * u[t] * y[t] - δU * u[t],
   v'[t] == βY * y[t] + βZ * z[t] - δV * v[t]
  };
```

In[]:=

```
(*finding fixed points numerically for Omega = 1*)
```

$$f1[x\_, y\_, z\_, u\_, v\_] := x * \left(rX - \frac{u}{aX + bX * u}\right) /. \text{para1};$$

$$f2[x\_, y\_, z\_, u\_, v\_] := y * \left(\eta * u - \rho - \frac{v}{aY + bY * v}\right) /. \text{para1};$$

$$f3[x\_, y\_, z\_, u\_, v\_] := z * \left(rZ - \frac{u}{aZ + bX * u}\right) /. \text{para1};$$

$$f4[x\_, y\_, z\_, u\_, v\_] := \phi * x + \kappa * \phi * z - \xi * u * y - \delta U * u /. \text{para1};$$

$$f5[x\_, y\_, z\_, u\_, v\_] := \beta Y * y + \beta Z * z - \delta V * v /. \text{para1};$$

```
Simplify[NSolve[{f1[x, y, z, u, v] == 0, f2[x, y, z, u, v] == 0, f3[x, y, z, u, v] == 0,
   f4[x, y, z, u, v] == 0, f5[x, y, z, u, v] == 0, x ≥ 0, y ≥ 0, z ≥ 0, u ≥ 0, v ≥ 0}, {x, y, z, u, v}]]
```

Out[]=

```
{{x → 0, z → 6.52767, v → 111.033, y → 42.4614, u → 164.245},
 {x → 2.30473, z → 0, v → 68.6927, y → 34.3464, u → 129.928}, {x → 0, z → 0, v → 0, y → 0, u → 0},
 {x → 0, z → 1.24428, v → 4.97713, y → 0, u → 164.245}, {x → 0.519711, z → 0, v → 0, y → 0, u → 129.928}}
```

In[]:=

```
E0 = {0, 0, 0, 0, 0}
```

$$E1 = \left\{\frac{aX\,rX\,\delta U}{\phi - bX\,rX\,\phi}, 0, 0, \frac{aX\,rX}{1 - bX\,rX}, 0\right\} /. \text{para1}$$

$$E2 = \left\{\frac{aX\,rX\left(-\delta U + \frac{aY\,\delta V\,\xi\,(aX\,rX\,\eta + (-1 + bX\,rX)\,\rho)}{\beta Y\,(-1 + aX\,bY\,rX\,\eta - bY\,\rho + bX\,(rX + bY\,rX\,\rho))}\right)}{(-1 + bX\,rX)\,\phi}, \frac{aY\,\delta V\,(-aX\,rX\,\eta + \rho - bX\,rX\,\rho)}{\beta Y\,(-1 + aX\,bY\,rX\,\eta - bY\,\rho + bX\,(rX + bY\,rX\,\rho))},\right.$$
$$\left. 0, \frac{aX\,rX}{1 - bX\,rX}, \frac{aY\,(-aX\,rX\,\eta + \rho - bX\,rX\,\rho)}{-1 + aX\,bY\,rX\,\eta - bY\,\rho + bX\,(rX + bY\,rX\,\rho)}\right\} /. \text{para1}$$

$$EM = \left\{0, 0, \frac{aZ\,rZ\,\delta U}{\kappa\,\phi - bX\,rZ\,\kappa\,\phi}, \frac{aZ\,rZ}{1 - bX\,rZ}, \frac{aZ\,rZ\,\beta Z\,\delta U}{\delta V\,\kappa\,\phi - bX\,rZ\,\delta V\,\kappa\,\phi}\right\} /. \text{para1}$$

$$EP = \left\{0, \frac{aZ^2\,bY\,rZ^2\,\beta Z\,\delta U\,\eta - aY\,(-1 + bX\,rZ)^2\,\delta V\,\kappa\,\rho\,\phi + aZ\,rZ\,(-1 + bX\,rZ)\,(\beta Z\,(\delta U + bY\,\delta U\,\rho) - aY\,\delta V\,\eta\,\kappa\,\phi)}{(-1 + aZ\,bY\,rZ\,\eta - bY\,\rho + bX\,(rZ + bY\,rZ\,\rho))\,(-aZ\,rZ\,\beta Z\,\xi + (-1 + bX\,rZ)\,\beta Y\,\kappa\,\phi)},\right.$$
$$-\frac{aZ\,rZ\,(aY\,\delta V\,\xi\,(-aZ\,rZ\,\eta + \rho - bX\,rZ\,\rho) + \beta Y\,\delta U\,(-1 + aZ\,bY\,rZ\,\eta - bY\,\rho + bX\,(rZ + bY\,rZ\,\rho)))}{(-1 + aZ\,bY\,rZ\,\eta - bY\,\rho + bX\,(rZ + bY\,rZ\,\rho))\,(-aZ\,rZ\,\beta Z\,\xi + (-1 + bX\,rZ)\,\beta Y\,\kappa\,\phi)},$$
$$\left.\frac{aZ\,rZ}{1 - bX\,rZ}, \frac{aY\,(-aZ\,rZ\,\eta + \rho - bX\,rZ\,\rho)}{-1 + aZ\,bY\,rZ\,\eta - bY\,\rho + bX\,(rZ + bY\,rZ\,\rho)}\right\} /. \text{para1}$$

Out[]=

```
{0, 0, 0, 0, 0}
```

Out[]=

```
{0.519711, 0, 0, 129.928, 0}
```

Out[]=

```
{2.30473, 34.3464, 0, 129.928, 68.6927}
```

Out[]=

```
{0, 0, 1.24428, 164.245, 4.97713}
```

Out[]=

```
{0, 42.4614, 6.52767, 164.245, 111.033}
```

In[]:=

```
(*Jacobian matrix at arbitrary (x,y,z,u,v) *)
jacobian[{x_, y_, z_, u_, v_}] =
  FullSimplify[D[{f1[x, y, z, u, v], f2[x, y, z, u, v], f3[x, y, z, u, v],
     f4[x, y, z, u, v], f5[x, y, z, u, v]}, {{x, y, z, u, v}}]];
```

In[]:=

```
Eigenvalues[jacobian[{x, y, z, u, v} /. {{x, y, z, u, v} → E0}]]
(*unstable*)
```

Out[]=

```
{56.5081, 24.7266, -2.87259, -1., -1.}
```

```
In[]:= Eigenvalues[jacobian[{x, y, z, u, v} /. {{x, y, z, u, v} → E1}]]
       (*unstable*)
Out[]=
       {27.1471 + 0. i, -0.5 + 4.93219 i, -0.5 - 4.93219 i, 4.10077 + 0. i, -1. + 0. i}

In[]:= Eigenvalues[jacobian[{x, y, z, u, v} /. {{x, y, z, u, v} → E2}]]
       (*unstable*)
Out[]=
       {-2.1505 + 14.6677 i, -2.1505 - 14.6677 i, 4.10077 + 0. i, -0.566819 + 2.76758 i, -0.566819 - 2.76758 i}

In[]:= Eigenvalues[jacobian[{x, y, z, u, v} /. {{x, y, z, u, v} → EM}]]
       (*unstable*)
Out[]=
       {31.9153 + 0. i, -5.64828 + 0. i, -0.5 + 4.28515 i, -0.5 - 4.28515 i, -1. + 0. i}

In[]:= Eigenvalues[jacobian[{x, y, z, u, v} /. {{x, y, z, u, v} → EP}]]
       (*stable*)
Out[]=
       {-2.56594 + 16.0807 i, -2.56594 - 16.0807 i,
        -5.64828 + 0. i, -0.557125 + 2.35278 i, -0.557125 - 2.35278 i}

In[]:=
       (*plotting corresponding solutions for x, y, and z*)

       Dsol1 =
         ParametricNDSolveValue[{dynamics /. para1, x[0] == x0, y[0] == y0, z[0] == z0, u[0] == u0, v[0] == v0},
          {x[t], y[t], z[t]}, {t, 0, 100}, {x0, y0, z0, u0, v0}];(*Omega = 1*)

       ic0 = Dsol1[0.01, 0.01, 0.01, 0.01, 0.01];
       ic1 = Dsol1[0.53, 0.01, 0.01, 131, 0.01];

       omega1 =
         Plot[Evaluate[Join[ic0, ic1]], {t, 0, 100}, ScalingFunctions → "Log",
          PlotPoints → 300, PlotStyle → {Directive[RGBColor[0.24, 0.6, 0.8], Thickness[0.008]],
            Directive[RGBColor[0.95, 0.627, 0.1425], Thickness[0.008]],
            Directive[RGBColor[0.455, 0.7, 0.21], Thickness[0.008]]},

          PlotRange → {{-1.2, 41.2}, {0.0001, Automatic}}, Frame → True, FrameStyle →
           Directive[Black, Thickness[0.006], 18], FrameTicks → {{Automatic, None}, {All, None}},

          Epilog → {Inset[Text[Style["Consortium, z", FontFamily → "Arial", Black, 21]], {32, 1}],
            Inset[Text[Style["Free-living symbiont, y", FontFamily → "Arial", Black, 21]], {27.2, 4.7}],
            Inset[Text[Style["Free-living host, x", FontFamily → "Arial", Black, 21]], {13.8, -7}]},

          ImageSize → {400, 300}, ImagePadding → {{50, 15}, {20, 10}}, AspectRatio → 0.75];

In[]:= (*finding fixed points numerically for Omega = 6*)
       f1[x_, y_, z_, u_, v_] := x * (rX - u/(aX + bX * u)) /. para2;
       f2[x_, y_, z_, u_, v_] := y * (η * u - ρ - v/(aY + bY * v)) /. para2;
       f3[x_, y_, z_, u_, v_] := z * (rZ - u/(aZ + bX * u)) /. para2;
       f4[x_, y_, z_, u_, v_] := ϕ * x + κ * ϕ * z - ξ * u * y - δU * u /. para2;
       f5[x_, y_, z_, u_, v_] := βY * y + βZ * z - δV * v /. para2;
       Simplify[NSolve[{f1[x, y, z, u, v] == 0, f2[x, y, z, u, v] == 0, f3[x, y, z, u, v] == 0,
           f4[x, y, z, u, v] == 0, f5[x, y, z, u, v] == 0, x ≥ 0, y ≥ 0, z ≥ 0, u ≥ 0, v ≥ 0}, {x, y, z, u, v}]];
```

In[•]:=

```
E0 = {0, 0, 0, 0, 0}
```

$$E1 = \left\{\frac{aX\,rX\,\delta U}{\phi - bX\,rX\,\phi}, 0, 0, \frac{aX\,rX}{1 - bX\,rX}, 0\right\} /.\ para2$$

$$E2 = \left\{\frac{aX\,rX\left(-\delta U + \frac{aY\,\delta V\,\xi\,(aX\,rX\,\eta + (-1+bX\,rX)\,\rho)}{\beta Y\,(-1+aX\,bY\,rX\,\eta - bY\,\rho + bX\,(rX+bY\,rX\,\rho))}\right)}{(-1 + bX\,rX)\,\phi}, \frac{aY\,\delta V\,(-aX\,rX\,\eta + \rho - bX\,rX\,\rho)}{\beta Y\,(-1 + aX\,bY\,rX\,\eta - bY\,\rho + bX\,(rX + bY\,rX\,\rho))},\right.$$
$$\left. 0, \frac{aX\,rX}{1 - bX\,rX}, \frac{aY\,(-aX\,rX\,\eta + \rho - bX\,rX\,\rho)}{-1 + aX\,bY\,rX\,\eta - bY\,\rho + bX\,(rX + bY\,rX\,\rho)}\right\} /.\ para2$$

$$EM = \left\{0, 0, \frac{aZ\,rZ\,\delta U}{\kappa\,\phi - bX\,rZ\,\kappa\,\phi}, \frac{aZ\,rZ}{1 - bX\,rZ}, \frac{aZ\,rZ\,\beta Z\,\delta U}{\delta V\,\kappa\,\phi - bX\,rZ\,\delta V\,\kappa\,\phi}\right\} /.\ para2$$

$$EP = \left\{0, \frac{aZ^2\,bY\,rZ^2\,\beta Z\,\delta U\,\eta - aY\,(-1 + bX\,rZ)^2\,\delta V\,\kappa\,\rho\,\phi + aZ\,rZ\,(-1 + bX\,rZ)\,(\beta Z\,(\delta U + bY\,\delta U\,\rho) - aY\,\delta V\,\eta\,\kappa\,\phi)}{(-1 + aZ\,bY\,rZ\,\eta - bY\,\rho + bX\,(rZ + bY\,rZ\,\rho))\,(-aZ\,rZ\,\beta Z\,\xi + (-1 + bX\,rZ)\,\beta Y\,\kappa\,\phi)},\right.$$
$$-\frac{aZ\,rZ\,(aY\,\delta V\,\xi\,(-aZ\,rZ\,\eta + \rho - bX\,rZ\,\rho) + \beta Y\,\delta U\,(-1 + aZ\,bY\,rZ\,\eta - bY\,\rho + bX\,(rZ + bY\,rZ\,\rho)))}{(-1 + aZ\,bY\,rZ\,\eta - bY\,\rho + bX\,(rZ + bY\,rZ\,\rho))\,(-aZ\,rZ\,\beta Z\,\xi + (-1 + bX\,rZ)\,\beta Y\,\kappa\,\phi)},$$
$$\left.\frac{aZ\,rZ}{1 - bX\,rZ}, \frac{aY\,(-aZ\,rZ\,\eta + \rho - bX\,rZ\,\rho)}{-1 + aZ\,bY\,rZ\,\eta - bY\,\rho + bX\,(rZ + bY\,rZ\,\rho)}\right\} /.\ para2$$

Out[•]=

```
{0, 0, 0, 0, 0}
```

Out[•]=

```
{0.519711, 0, 0, 129.928, 0}
```

Out[•]=

```
{0.53401, 1.65078, 0, 129.928, 3.30155}
```

Out[•]=

```
{0, 0, 1.24428, 164.245, 4.97713}
```

Out[•]=

```
{0, 0.23234, 1.2491, 164.245, 5.46108}
```

In[•]:=

```
(*Jacobian matrix at arbitrary (x,y,z,u,v) *)
jacobian[{x_, y_, z_, u_, v_}] =
  FullSimplify[D[{f1[x, y, z, u, v], f2[x, y, z, u, v], f3[x, y, z, u, v],
     f4[x, y, z, u, v], f5[x, y, z, u, v]}, {{x, y, z, u, v}}]];
```

In[•]:=

```
Eigenvalues[jacobian[{x, y, z, u, v} /. {{x, y, z, u, v} → E0}]]
(*unstable*)
```

Out[•]=

```
{56.5081, 24.7266, -2.87259, -1., -1.}
```

In[•]:=

```
Eigenvalues[jacobian[{x, y, z, u, v} /. {{x, y, z, u, v} → E1}]]
(*unstable*)
```

Out[•]=

```
{-0.5 + 4.93219 i, -0.5 - 4.93219 i, 4.10077 + 0. i, 2.13069 + 0. i, -1. + 0. i}
```

In[•]:=

```
Eigenvalues[jacobian[{x, y, z, u, v} /. {{x, y, z, u, v} → E2}]]
(*unstable*)
```

Out[•]=

```
{-0.513749 + 5.01383 i, -0.513749 - 5.01383 i,
 4.10077 + 0. i, -0.500007 + 1.3418 i, -0.500007 - 1.3418 i}
```

In[•]:=

```
Eigenvalues[jacobian[{x, y, z, u, v} /. {{x, y, z, u, v} → EM}]]
(*unstable*)
```

Out[•]=

```
{-5.64828 + 0. i, -0.5 + 4.28515 i, -0.5 - 4.28515 i, -1. + 0. i, 0.291424 + 0. i}
```

In[•]:=
```
Eigenvalues[jacobian[{x, y, z, u, v} /. {{x, y, z, u, v} → EP}]]
(*stable*)
```
Out[•]=
```
{-5.64828 + 0. i, -0.501937 + 4.29486 i,
 -0.501937 - 4.29486 i, -0.499999 + 0.19971 i, -0.499999 - 0.19971 i}
```
In[•]:=
```
(*plotting corresponding solutions for x, y, and z*)

Dsol2 =
  ParametricNDSolveValue[{dynamics /. para2, x[0] == x0, y[0] == y0, z[0] == z0, u[0] == u0, v[0] == v0},
   {x[t], y[t], z[t]}, {t, 0, 100}, {x0, y0, z0, u0, v0}];(*Omega = 6*)

ic0 = Dsol2[0.01, 0.01, 0.01, 0.01, 0.01];
ic1 = Dsol2[0.53, 0.01, 0.01, 131, 0.01];

omega6 =
  Plot[Evaluate[Join[ic0, ic1]], {t, 0, 100}, ScalingFunctions → "Log",
   PlotPoints → 300, PlotStyle → {Directive[RGBColor[0.24, 0.6, 0.8], Thickness[0.008]],
     Directive[RGBColor[0.95, 0.627, 0.1425], Thickness[0.008]],
     Directive[RGBColor[0.455, 0.7, 0.21], Thickness[0.008]]},

   PlotRange → {{-1.2, 41.2}, {0.0001, Automatic}}, Frame → True, FrameStyle →
    Directive[Black, Thickness[0.006], 18], FrameTicks → {{Automatic, None}, {All, None}},

   Epilog →
    {Inset[Text[Style["Consortium, z", FontFamily → "Arial", Black, 21]], {32.5, 1}], Inset[
      Text[Style["Free-living symbiont, y", FontFamily → "Arial", Black, 21]], {27.3, -2.2}]},

   ImageSize → {400, 300}, ImagePadding → {{50, 15}, {20, 10}}, AspectRatio → 0.75];
```
In[•]:=
```
(*finding fixed points numerically for Omega = 10*)
f1[x_, y_, z_, u_, v_] := x * (rX - u/(aX + bX * u)) /. para3;
f2[x_, y_, z_, u_, v_] := y * (η * u - ρ - v/(aY + bY * v)) /. para3;
f3[x_, y_, z_, u_, v_] := z * (rZ - u/(aZ + bX * u)) /. para3;
f4[x_, y_, z_, u_, v_] := ϕ * x + κ * ϕ * z - ξ * u * y - δU * u /. para3;
f5[x_, y_, z_, u_, v_] := βY * y + βZ * z - δV * v /. para3;
Simplify[NSolve[{f1[x, y, z, u, v] == 0, f2[x, y, z, u, v] == 0, f3[x, y, z, u, v] == 0,
   f4[x, y, z, u, v] == 0, f5[x, y, z, u, v] == 0, x ≥ 0, y ≥ 0, z ≥ 0, u ≥ 0, v ≥ 0}, {x, y, z, u, v}]]
```
Out[•]=
```
{{x → 0, z → 0, v → 0, y → 0, u → 0}, {x → 0.519711, z → 0, v → 0, y → 0, u → 129.928},
 {x → 0.520216, z → 0, v → 0.194445, y → 0.0972227, u → 129.928},
 {x → 0, z → 1.24428, v → 4.97713, y → 0, u → 164.245}}
```

In[•]:= E0 = {0, 0, 0, 0, 0}

$$E1 = \left\{\frac{aX\ rX\ \delta U}{\phi - bX\ rX\ \phi}, 0, 0, \frac{aX\ rX}{1 - bX\ rX}, 0\right\} \text{ /. para3}$$

$$E2 = \left\{\frac{aX\ rX\left(-\delta U + \frac{aY\ \delta V\ \xi\ (aX\ rX\ \eta + (-1+bX\ rX)\ \rho)}{\beta Y\ (-1+aX\ bY\ rX\ \eta - bY\ \rho + bX\ (rX+bY\ rX\ \rho))}\right)}{(-1 + bX\ rX)\ \phi}, \frac{aY\ \delta V\ (-aX\ rX\ \eta + \rho - bX\ rX\ \rho)}{\beta Y\ (-1 + aX\ bY\ rX\ \eta - bY\ \rho + bX\ (rX + bY\ rX\ \rho))}, 0, \frac{aX\ rX}{1 - bX\ rX}, \frac{aY\ (-aX\ rX\ \eta + \rho - bX\ rX\ \rho)}{-1 + aX\ bY\ rX\ \eta - bY\ \rho + bX\ (rX + bY\ rX\ \rho)}\right\} \text{ /. para3}$$

$$EM = \left\{0, 0, \frac{aZ\ rZ\ \delta U}{\kappa\ \phi - bX\ rZ\ \kappa\ \phi}, \frac{aZ\ rZ}{1 - bX\ rZ}, \frac{aZ\ rZ\ \beta Z\ \delta U}{\delta V\ \kappa\ \phi - bX\ rZ\ \delta V\ \kappa\ \phi}\right\} \text{ /. para3}$$

Out[•]=
{0, 0, 0, 0, 0}

Out[•]=
{0.519711, 0, 0, 129.928, 0}

Out[•]=
{0.520216, 0.0972227, 0, 129.928, 0.194445}

Out[•]=
{0, 0, 1.24428, 164.245, 4.97713}

In[•]:=
```
(*Jacobian matrix at arbitrary (x,y,z,u,v) *)
jacobian[{x_, y_, z_, u_, v_}] =
  FullSimplify[D[{f1[x, y, z, u, v], f2[x, y, z, u, v], f3[x, y, z, u, v],
     f4[x, y, z, u, v], f5[x, y, z, u, v]}, {{x, y, z, u, v}}]];
```

In[•]:=
```
Eigenvalues[jacobian[{x, y, z, u, v} /. {{x, y, z, u, v} → E0}]]
(*unstable*)
```

Out[•]=
{56.5081, 24.7266, -2.87259, -1., -1.}

In[•]:=
```
Eigenvalues[jacobian[{x, y, z, u, v} /. {{x, y, z, u, v} → E1}]]
(*unstable*)
```

Out[•]=
{-0.5 + 4.93219 i, -0.5 - 4.93219 i, 4.10077 + 0. i, -1. + 0. i, 0.129379 + 0. i}

In[•]:=
```
Eigenvalues[jacobian[{x, y, z, u, v} /. {{x, y, z, u, v} → E2}]]
(*unstable*)
```

Out[•]=
{-0.500486 + 4.93485 i, -0.500486 - 4.93485 i, 4.10077 + 0. i, -0.847689 + 0. i, -0.152311 + 0. i}

In[•]:=
```
Eigenvalues[jacobian[{x, y, z, u, v} /. {{x, y, z, u, v} → EM}]]
(*stable*)
```

Out[•]=
{-5.64828 + 0. i, -0.5 + 4.28515 i, -0.5 - 4.28515 i, -2.23849 + 0. i, -1. + 0. i}

```
In[•]:= (*plotting corresponding solutions for x, y, and z*)
Dsol3 =
  ParametricNDSolveValue[{dynamics /. para3, x[0] == x0, y[0] == y0, z[0] == z0, u[0] == u0, v[0] == v0},
   {x[t], y[t], z[t]}, {t, 0, 100}, {x0, y0, z0, u0, v0}];(*Omega = 10*)

ic0 = Dsol3[0.01, 0.01, 0.01, 0.01, 0.01];
ic1 = Dsol3[0.53, 0.01, 0.01, 131, 0.01];

omega10 =
  Plot[Evaluate[Join[ic0, ic1]], {t, 0, 100}, ScalingFunctions → "Log", PlotPoints → 300,

   PlotStyle → {Directive[RGBColor[0.24, 0.6, 0.8], Thickness[0.008]],
     Directive[RGBColor[0.95, 0.627, 0.1425], Thickness[0.008]],
     Directive[RGBColor[0.455, 0.7, 0.21], Thickness[0.008]]},
   PlotRange → {{-1.2, 41.2}, {0.0001, Automatic}}, Frame → True, FrameStyle →
    Directive[Black, Thickness[0.006], 18], FrameTicks → {{Automatic, None}, {All, None}},

   Epilog → {Inset[Text[Style["Consortium, z", FontFamily → "Arial", Black, 21]], {32, 1}],
     Inset[Text[Style["Free-living symbiont, y", FontFamily → "Arial", Black, 21]], {18, -7}]},

   ImageSize → {400, 300}, ImagePadding → {{50, 15}, {20, 10}}, AspectRatio → 0.75];

In[•]:= (*Fig A1 in S1 Appendix*)
omega =
  Pane[Grid[
    MapThread[Prepend,
     {
      Append[
       Prepend[{{omega1, omega6, omega10}}, {Style[" Ω = 1 (low dilution)",
          FontFamily → "Arial", 24, Black], Style["Ω = 6", FontFamily → "Arial", 24, Black],
         Style["Ω = 10 (high dilution)", FontFamily → "Arial", 24, Black]}],

       {Style["Time", FontFamily → "Arial", 24, Black], Style["Time",
         FontFamily → "Arial", 24, Black], Style["Time", FontFamily → "Arial", 24, Black]}],

      {"", Rotate[Style["Population densities", FontFamily → "Arial", 24, Black], 90 Degree], ""}

     }], Spacings → {1, 0}], ImageMargins → {{15, 5}, {10, 10}}];

(*NOTE: Low dilution of host metabolite (via a small habitat volume) stabilizes the free-
  living symbionts (stable EP). On the contrary,
higher values of dilution factor leads to their extinction
 (stability of EM rather than EP). See Appendix A4.1 in S1 Appendix.*)

In[•]:= SetDirectory[NotebookDirectory[]];
Export["FigA1.tiff", omega, ImageResolution → 600];
```

2.6. Sensitivity to escape factor

```
In[•]:= ClearAll["`*"];
```

In[∘]:=
```
(*arbitrary parameter values with varied τ*)

para1 = {w → 500, kX → 0.5, cX → 3, lX → 4, dX → 0.33, kY → 0.1, Ω → 10, cY → 0.3,
   lY → 1.2, dY → 0.1, aX → 1, bX → 0.01, aY → 1.5, bY → 0.015, δU → 1, δV → 1, βY → 2,
   lZ → 5, cZ → 4, dZ → 0.4, n → 2, pZ → 0.2, kYZ → 0.06, βZ → 4, s → 0.4, τ → 0.01};

para2 = {w → 500, kX → 0.5, cX → 3, lX → 4, dX → 0.33, kY → 0.1, Ω → 10, cY → 0.3,
   lY → 1.2, dY → 0.1, aX → 1, bX → 0.01, aY → 1.5, bY → 0.015, δU → 1, δV → 1, βY → 2,
   lZ → 5, cZ → 4, dZ → 0.4, n → 2, pZ → 0.2, kYZ → 0.06, βZ → 4, s → 0.4, τ → 0.001};

para3 = {w → 500, kX → 0.5, cX → 3, lX → 4, dX → 0.33, kY → 0.1, Ω → 10,
   cY → 0.3, lY → 1.2, dY → 0.1, aX → 1, bX → 0.01, aY → 1.5, bY → 0.015, δU → 1, δV → 1,
   βY → 2, lZ → 5, cZ → 4, dZ → 0.4, n → 2, pZ → 0.2, kYZ → 0.06, βZ → 4, s → 0.4, τ → 0};


(*substitutions to simplify the system*)
aZ = aX/(1 - n*s);
κ = (1 - kYZ*n)*(1 - n*pZ);
kZ = kX*(1 - n*pZ);
rX = (kX*w - lX)/cX * N[Log[2]] - dX;
rZ = (kZ*w - lZ)/cZ * N[Log[2]] - dZ;
η = kY/(Ω*cY) * (N[Log[2]]);
ρ = lY/cY * (N[Log[2]]) + dY;
ϕ = kX*w;
ξ = kY/Ω;

(*dynamical system with the symbiont escape factor τ as in Appendix A4.2 in S1 Appendix*)
dynamics = {
   x'[t] == x[t]*(rX - u[t]/(aX + bX*u[t])),
   y'[t] == y[t]*(η*u[t] - ρ - v[t]/(aY + bY*v[t])) + τ*z[t],
   z'[t] == z[t]*(rZ - u[t]/(aZ + bX*u[t])),
   u'[t] == ϕ*x[t] + κ*ϕ*z[t] - ξ*u[t]*y[t] - δU*u[t],
   v'[t] == βY*y[t] + βZ*z[t] - δV*v[t]
  };
```

In[ ]:=
```
(*numerical solutions for τ = 0.01*)
f1[x_, y_, z_, u_, v_] := x * (rX - u/(aX + bX * u)) /. para1;
f2[x_, y_, z_, u_, v_] := y * (η * u - ρ - v/(aY + bY * v)) + τ * z /. para1;
f3[x_, y_, z_, u_, v_] := z * (rZ - u/(aZ + bX * u)) /. para1;
f4[x_, y_, z_, u_, v_] := ϕ * x + κ * ϕ * z - ξ * u * y - δU * u /. para1;
f5[x_, y_, z_, u_, v_] := βY * y + βZ * z - δV * v /. para1;
Simplify[NSolve[{f1[x, y, z, u, v] == 0, f2[x, y, z, u, v] == 0, f3[x, y, z, u, v] == 0,
   f4[x, y, z, u, v] == 0, f5[x, y, z, u, v] == 0, x ≥ 0, y ≥ 0, z ≥ 0, u ≥ 0, v ≥ 0}, {x, y, z, u, v}]]
```

Out[ ]=
```
{{x → 0, v → 0, z → 0, y → 0, u → 0}, {x → 0.519711, v → 0, z → 0, y → 0, u → 129.928},
 {x → 0.520216, v → 0.194445, z → 0, y → 0.0972227, u → 129.928},
 {x → 0, v → 4.98849, z → 1.24435, y → 0.00554188, u → 164.245}}
```

In[ ]:=
```
E0 = {0, 0, 0, 0, 0}

E1 = {(aX rX δU)/(ϕ - bX rX ϕ), 0, 0, (aX rX)/(1 - bX rX), 0} /. para1

E2 = {(aX rX (-δU + (aY δV ξ (aX rX η + (-1+bX rX) ρ))/(βY (-1+aX bY rX η-bY ρ+bX (rX+bY rX ρ)))))/((-1 + bX rX) ϕ),
   (aY δV (-aX rX η + ρ - bX rX ρ))/(βY (-1 + aX bY rX η - bY ρ + bX (rX + bY rX ρ))),
   0, (aX rX)/(1 - bX rX), (aY (-aX rX η + ρ - bX rX ρ))/(-1 + aX bY rX η - bY ρ + bX (rX + bY rX ρ))} /. para1
EP = {0, 0.00554188, 1.24435, 164.245, 4.98849}
```

Out[ ]=
```
{0, 0, 0, 0, 0}
```

Out[ ]=
```
{0.519711, 0, 0, 129.928, 0}
```

Out[ ]=
```
{0.520216, 0.0972227, 0, 129.928, 0.194445}
```

Out[ ]=
```
{0, 0.00554188, 1.24435, 164.245, 4.98849}
```

In[ ]:=
```
(*Jacobian matrix at arbitrary (x,y,z,u,v) *)
jacobian[{x_, y_, z_, u_, v_}] =
  FullSimplify[D[{f1[x, y, z, u, v], f2[x, y, z, u, v], f3[x, y, z, u, v],
     f4[x, y, z, u, v], f5[x, y, z, u, v]}, {{x, y, z, u, v}}]];
```

In[ ]:=
```
Eigenvalues[jacobian[{x, y, z, u, v} /. {{x, y, z, u, v} → E0}]]
(*unstable*)
```

Out[ ]=
```
{56.5081, 24.7266, -2.87259, -1., -1.}
```

In[ ]:=
```
Eigenvalues[jacobian[{x, y, z, u, v} /. {{x, y, z, u, v} → E1}]]
(*unstable*)
```

Out[ ]=
```
{-0.5 + 4.93219 i, -0.5 - 4.93219 i, 4.10077 + 0. i, -1. + 0. i, 0.129379 + 0. i}
```

In[ ]:=
```
Eigenvalues[jacobian[{x, y, z, u, v} /. {{x, y, z, u, v} → E2}]]
(*unstable*)
```

Out[ ]=
```
{-0.500486 + 4.93485 i, -0.500486 - 4.93485 i, 4.10077 + 0. i, -0.847689 + 0. i, -0.152311 + 0. i}
```

```
In[]:= Eigenvalues[jacobian[{x, y, z, u, v} /. {{x, y, z, u, v} → EP}]]
       (*stable*)
Out[]=
       {-5.64826 + 0. i, -0.500076 + 4.28525 i, -0.500076 - 4.28525 i, -2.23971 + 0. i, -1.00554 + 0. i}

In[]:= (*plotting corresponding solutions for x, y, and z*)

       Dsol1 =
         ParametricNDSolveValue[{dynamics /. para1, x[0] == x0, y[0] == y0, z[0] == z0, u[0] == u0, v[0] == v0},
          {x[t], y[t], z[t]}, {t, 0, 100}, {x0, y0, z0, u0, v0}];(*τ = 0.01*)

       ic0 = Dsol1[0.01, 0.01, 0.01, 0.01, 0.01];
       ic1 = Dsol1[0.53, 0.01, 0.01, 131, 0.01];
       ic2 = Dsol1[0.53, 0.1, 0.01, 131, 0.2];

       τ1 =
         Plot[Evaluate[Join[ic0, ic1, ic2]], {t, 0, 100}, ScalingFunctions → "Log", PlotPoints → 300,

          PlotStyle → {Directive[RGBColor[0.24, 0.6, 0.8], Thickness[0.008]],
            Directive[RGBColor[0.95, 0.627, 0.1425], Thickness[0.008]],
            Directive[RGBColor[0.455, 0.7, 0.21], Thickness[0.008]]},

          PlotRange → {{-1.2, 41.2}, {0.0001, Automatic}}, Frame → True, FrameStyle →
           Directive[Black, Thickness[0.006], 18], FrameTicks → {{Automatic, None}, {All, None}},

          Epilog → {Inset[Text[Style["Consortium, z", FontFamily → "Arial", Black, 21]], {32.5, 1}],
            Inset[Text[Style["Free-living symbiont, y", FontFamily → "Arial", Black, 21]], {27, -4.4}],
            Inset[Text[Style["Free-living host, x", FontFamily → "Arial", Black, 21]], {13.7, -8}]},

          ImageSize → {400, 300}, ImagePadding → {{50, 15}, {20, 10}}, AspectRatio → 0.75] ;

In[]:= (*numerical solutions for τ = 0.001*)
       f1[x_, y_, z_, u_, v_] := x * (rX - u/(aX + bX * u)) /. para2;
       f2[x_, y_, z_, u_, v_] := y * (η * u - ρ - v/(aY + bY * v)) + τ * z /. para2;
       f3[x_, y_, z_, u_, v_] := z * (rZ - u/(aZ + bX * u)) /. para2;
       f4[x_, y_, z_, u_, v_] := ϕ * x + κ * ϕ * z - ξ * u * y - δU * u /. para2;
       f5[x_, y_, z_, u_, v_] := βY * y + βZ * z - δV * v /. para2;
       Simplify[NSolve[{f1[x, y, z, u, v] == 0, f2[x, y, z, u, v] == 0, f3[x, y, z, u, v] == 0,
          f4[x, y, z, u, v] == 0, f5[x, y, z, u, v] == 0, x ≥ 0, y ≥ 0, z ≥ 0, u ≥ 0, v ≥ 0}, {x, y, z, u, v}]]
Out[]=
       {{x → 0, v → 0, z → 0, y → 0, u → 0}, {x → 0.519711, v → 0, z → 0, y → 0, u → 129.928},
        {x → 0.520216, v → 0.194445, z → 0, y → 0.0972227, u → 129.928},
        {x → 0, v → 4.97826, z → 1.24429, y → 0.00055569, u → 164.245}}
```

In[*]:=
```
E0 = {0, 0, 0, 0, 0}
```

$$E1 = \left\{\frac{aX\,rX\,\delta U}{\phi - bX\,rX\,\phi}, 0, 0, \frac{aX\,rX}{1 - bX\,rX}, 0\right\} /.\ para3$$

$$E2 = \left\{\frac{aX\,rX\left(-\delta U + \frac{aY\,\delta V\,\xi\,(aX\,rX\,\eta + (-1+bX\,rX)\,\rho)}{\beta Y\,(-1+aX\,bY\,rX\,\eta - bY\,\rho + bX\,(rX+bY\,rX\,\rho))}\right)}{(-1 + bX\,rX)\,\phi}, \frac{aY\,\delta V\,(-aX\,rX\,\eta + \rho - bX\,rX\,\rho)}{\beta Y\,(-1 + aX\,bY\,rX\,\eta - bY\,\rho + bX\,(rX + bY\,rX\,\rho))},\right.$$
$$\left. 0, \frac{aX\,rX}{1 - bX\,rX}, \frac{aY\,(-aX\,rX\,\eta + \rho - bX\,rX\,\rho)}{-1 + aX\,bY\,rX\,\eta - bY\,\rho + bX\,(rX + bY\,rX\,\rho)}\right\} /.\ para3$$

```
EP = {0, 0.00055569, 1.24429, 164.245, 4.97826}
```

Out[*]=
```
{0, 0, 0, 0, 0}
```

Out[*]=
```
{0.519711, 0, 0, 129.928, 0}
```

Out[*]=
```
{0.520216, 0.0972227, 0, 129.928, 0.194445}
```

Out[*]=
```
{0, 0.00055569, 1.24429, 164.245, 4.97826}
```

In[*]:=
```
(*Jacobian matrix at arbitrary (x,y,z,u,v) *)
jacobian[{x_, y_, z_, u_, v_}] =
  FullSimplify[D[{f1[x, y, z, u, v], f2[x, y, z, u, v], f3[x, y, z, u, v],
     f4[x, y, z, u, v], f5[x, y, z, u, v]}, {{x, y, z, u, v}}]];
```

In[*]:=
```
Eigenvalues[jacobian[{x, y, z, u, v} /. {{x, y, z, u, v} → E0}]]
(*unstable*)
```

Out[*]=
```
{56.5081, 24.7266, -2.87259, -1., -1.}
```

In[*]:=
```
Eigenvalues[jacobian[{x, y, z, u, v} /. {{x, y, z, u, v} → E1}]]
(*unstable*)
```

Out[*]=
```
{-0.5 + 4.93219 i, -0.5 - 4.93219 i, 4.10077 + 0. i, -1. + 0. i, 0.129379 + 0. i}
```

In[*]:=
```
Eigenvalues[jacobian[{x, y, z, u, v} /. {{x, y, z, u, v} → E2}]]
(*unstable*)
```

Out[*]=
```
{-0.500486 + 4.93485 i, -0.500486 - 4.93485 i, 4.10077 + 0. i, -0.847689 + 0. i, -0.152311 + 0. i}
```

In[*]:=
```
Eigenvalues[jacobian[{x, y, z, u, v} /. {{x, y, z, u, v} → EP}]]
(*stable*)
```

Out[*]=
```
{-5.64826 + 0. i, -0.5 + 4.28517 i, -0.5 - 4.28517 i, -2.23861 + 0. i, -1.00056 + 0. i}
```

In[•]:=
```
(*plotting corresponding solutions for x, y, and z*)

Dsol2 =
  ParametricNDSolveValue[{dynamics /. para2, x[0] == x0, y[0] == y0, z[0] == z0, u[0] == u0, v[0] == v0},
   {x[t], y[t], z[t]}, {t, 0, 100}, {x0, y0, z0, u0, v0}];(*τ = 0.001*)

ic0 = Dsol2[0.01, 0.01, 0.01, 0.01, 0.01];
ic1 = Dsol2[0.53, 0.01, 0.01, 131, 0.01];
ic2 = Dsol2[0.53, 0.1, 0.01, 131, 0.2];

τ2 =
  Plot[Evaluate[Join[ic0, ic1, ic2]], {t, 0, 100}, ScalingFunctions → "Log",
   PlotPoints → 300, PlotStyle → {Directive[RGBColor[0.24, 0.6, 0.8], Thickness[0.008]],
     Directive[RGBColor[0.95, 0.627, 0.1425], Thickness[0.008]],
     Directive[RGBColor[0.455, 0.7, 0.21], Thickness[0.008]]},

   PlotRange → {{-1.2, 41.2}, {0.0001, Automatic}}, Frame → True, FrameStyle →
    Directive[Black, Thickness[0.006], 18], FrameTicks → {{Automatic, None}, {All, None}},

   Epilog →
    {Inset[Text[Style["Consortium, z", FontFamily → "Arial", Black, 21]], {32.5, 1}], Inset[
      Text[Style["Free-living symbiont, y", FontFamily → "Arial", Black, 21]], {27, -6.7}]},

   ImageSize → {400, 300}, ImagePadding → {{50, 15}, {20, 10}}, AspectRatio → 0.75];
```

In[•]:=
```
(*numerical solutions for τ = 0*)
f1[x_, y_, z_, u_, v_] := x * (rX - u/(aX + bX * u)) /. para3;
f2[x_, y_, z_, u_, v_] := y * (η * u - ρ - v/(aY + bY * v)) + τ * z /. para3;
f3[x_, y_, z_, u_, v_] := z * (rZ - u/(aZ + bX * u)) /. para3;
f4[x_, y_, z_, u_, v_] := ϕ * x + κ * ϕ * z - ξ * u * y - δU * u /. para3;
f5[x_, y_, z_, u_, v_] := βY * y + βZ * z - δV * v /. para3;
Simplify[NSolve[{f1[x, y, z, u, v] == 0, f2[x, y, z, u, v] == 0, f3[x, y, z, u, v] == 0,
   f4[x, y, z, u, v] == 0, f5[x, y, z, u, v] == 0, x ≥ 0, y ≥ 0, z ≥ 0, u ≥ 0, v ≥ 0}, {x, y, z, u, v}]]
```

Out[•]=
```
{{x → 0, z → 0, v → 0, y → 0, u → 0}, {x → 0.519711, z → 0, v → 0, y → 0, u → 129.928},
 {x → 0.520216, z → 0, v → 0.194445, y → 0.0972227, u → 129.928},
 {x → 0, z → 1.24428, v → 4.97713, y → 0, u → 164.245}}
```

In[•]:=

```
E0 = {0, 0, 0, 0, 0}
```

$$E1 = \left\{\frac{aX\ rX\ \delta U}{\phi - bX\ rX\ \phi}, 0, 0, \frac{aX\ rX}{1 - bX\ rX}, 0\right\} \text{/. para3}$$

$$E2 = \left\{\frac{aX\ rX\left(-\delta U + \frac{aY\ \delta V\ \xi\ (aX\ rX\ \eta + (-1 + bX\ rX)\ \rho)}{\beta Y\ (-1 + aX\ bY\ rX\ \eta - bY\ \rho + bX\ (rX + bY\ rX\ \rho))}\right)}{(-1 + bX\ rX)\ \phi}, \frac{aY\ \delta V\ (-aX\ rX\ \eta + \rho - bX\ rX\ \rho)}{\beta Y\ (-1 + aX\ bY\ rX\ \eta - bY\ \rho + bX\ (rX + bY\ rX\ \rho))}, 0, \frac{aX\ rX}{1 - bX\ rX}, \frac{aY\ (-aX\ rX\ \eta + \rho - bX\ rX\ \rho)}{-1 + aX\ bY\ rX\ \eta - bY\ \rho + bX\ (rX + bY\ rX\ \rho)}\right\} \text{/. para3}$$

```
EM = {0, 0, 1.24428, 164.245, 4.97713}
```

Out[•]=

{0, 0, 0, 0, 0}

Out[•]=

{0.519711, 0, 0, 129.928, 0}

Out[•]=

{0.520216, 0.0972227, 0, 129.928, 0.194445}

Out[•]=

{0, 0, 1.24428, 164.245, 4.97713}

In[•]:=

```
(*Jacobian matrix at arbitrary (x,y,z,u,v) *)
jacobian[{x_, y_, z_, u_, v_}] =
  FullSimplify[D[{f1[x, y, z, u, v], f2[x, y, z, u, v], f3[x, y, z, u, v],
     f4[x, y, z, u, v], f5[x, y, z, u, v]}, {{x, y, z, u, v}}]];
```

In[•]:=

```
Eigenvalues[jacobian[{x, y, z, u, v} /. {{x, y, z, u, v} → E0}]]
(*unstable*)
```

Out[•]=

{56.5081, 24.7266, -2.87259, -1., -1.}

In[•]:=

```
Eigenvalues[jacobian[{x, y, z, u, v} /. {{x, y, z, u, v} → E1}]]
(*unstable*)
```

Out[•]=

{-0.5 + 4.93219 i, -0.5 - 4.93219 i, 4.10077 + 0. i, -1. + 0. i, 0.129379 + 0. i}

In[•]:=

```
Eigenvalues[jacobian[{x, y, z, u, v} /. {{x, y, z, u, v} → E2}]]
(*unstable*)
```

Out[•]=

{-0.500486 + 4.93485 i, -0.500486 - 4.93485 i, 4.10077 + 0. i, -0.847689 + 0. i, -0.152311 + 0. i}

In[•]:=

```
Eigenvalues[jacobian[{x, y, z, u, v} /. {{x, y, z, u, v} → EM}]]
(*stable*)
```

Out[•]=

{-5.64826 + 0. i, -0.499992 + 4.28515 i, -0.499992 - 4.28515 i, -2.23849 + 0. i, -1. + 0. i}

```
In[•]:= (*plotting corresponding solutions for x, y, and z*)

        Dsol3 =
          ParametricNDSolveValue[{dynamics /. para3, x[0] == x0, y[0] == y0, z[0] == z0, u[0] == u0, v[0] == v0},
           {x[t], y[t], z[t]}, {t, 0, 100}, {x0, y0, z0, u0, v0}];(*τ = 0*)

        ic0 = Dsol3[0.01, 0.01, 0.01, 0.01, 0.01];
        ic1 = Dsol3[0.53, 0.01, 0.01, 131, 0.01];
        ic2 = Dsol3[0.53, 0.1, 0.01, 131, 0.2];

        τ3 =
          Plot[Evaluate[Join[ic0, ic1, ic2]], {t, 0, 100}, ScalingFunctions → "Log", PlotPoints → 300,

           PlotStyle → {Directive[RGBColor[0.24, 0.6, 0.8], Thickness[0.008]],
             Directive[RGBColor[0.95, 0.627, 0.1425], Thickness[0.008]],
             Directive[RGBColor[0.455, 0.7, 0.21], Thickness[0.008]]},

           PlotRange → {{-1.2, 41.2}, {0.0001, Automatic}}, Frame → True, FrameStyle →
            Directive[Black, Thickness[0.006], 18], FrameTicks → {{Automatic, None}, {All, None}},

           Epilog → {Inset[Text[Style["Consortium, z", FontFamily → "Arial", Black, 21]], {32.5, 1}],
             Inset[Text[Style["Free-living symbiont, y", FontFamily → "Arial", Black, 21]], {19, -8}]},

           ImageSize → {400, 300}, ImagePadding → {{50, 15}, {20, 10}}, AspectRatio → 0.75];

In[•]:= (*Fig A2 in S1 Appendix*)
        escape = Pane[Grid[
            MapThread[Prepend,
             {
              Append[
               Prepend[{{τ1, τ2, τ3}},
                {Style["τ = 0.01", FontFamily → "Arial", 24, Black], Style["τ = 0.001",
                  FontFamily → "Arial", 24, Black], Style["τ = 0", FontFamily → "Arial", 24, Black]}],

               {Style["Time", FontFamily → "Arial", 24, Black], Style["Time",
                 FontFamily → "Arial", 24, Black], Style["Time", FontFamily → "Arial", 24, Black]}],

              {"", Rotate[Style["Population densities", FontFamily → "Arial", 24, Black], 90 Degree], ""}

             }], Spacings → {1, 0}], ImageMargins → {{15, 5}, {10, 10}}];

        (*NOTE: Only synchronized host-symbiont replication
           (release or escape factor τ = 0) gives extinction of free-living symbionts in our model. y-
          density increases as τ increases. See Appendix A4.2 in S1 Appendix.*)

In[•]:= SetDirectory[NotebookDirectory[]];
        Export["FigA2.tiff", escape, ImageResolution → 600];
```

2.7. Imperfect vertical inheritance

```
In[•]:= ClearAll["`*"];
```

```
In[•]:= (*assume the vertical inheritance of consortial hosts (Z) is not completely accurate;
effectiveness of vertical inheritance is θ*)
f1[x_, y_, z_, u_, v_] := x * (rX - u/(aX + bX * u)) + (1 - θ) * rZ * z;
f2[x_, y_, z_, u_, v_] := y * (η * u - ρ - v/(aY + bY * v));
f3[x_, y_, z_, u_, v_] := z * (θ * rZ - u/(aZ + bX * u));
f4[x_, y_, z_, u_, v_] := ϕ * x + κ * ϕ * z - ξ * u * y - δU * u;
f5[x_, y_, z_, u_, v_] := βY * y + βZ * z - δV * v;
Simplify[Solve[{f1[x, y, z, u, v] == 0 && f2[x, y, z, u, v] == 0 &&
    f3[x, y, z, u, v] == 0 && f4[x, y, z, u, v] == 0 && f5[x, y, z, u, v] == 0}, {x, y, z, u, v}]]
```

Out[*]=

$$\left\{\left\{x\to 0,\ y\to -\frac{aY\,\delta V\,\rho}{\beta Y+bY\,\beta Y\,\rho},\ z\to 0,\ u\to 0,\ v\to -\frac{aY\,\rho}{1+bY\,\rho}\right\},\right.$$

$$\left\{x\to \frac{aX\,rX\left(-\delta U+\frac{aY\,\delta V\,\xi\,(aX\,rX\,\eta+(-1+bX\,rX)\,\rho)}{\beta Y\,(-1+aX\,bY\,rX\,\eta-bY\,\rho+bX\,(rX+bY\,rX\,\rho))}\right)}{(-1+bX\,rX)\,\phi},\ y\to \frac{aY\,\delta V\,(-aX\,rX\,\eta+\rho-bX\,rX\,\rho)}{\beta Y\,(-1+aX\,bY\,rX\,\eta-bY\,\rho+bX\,(rX+bY\,rX\,\rho))},\right.$$

$$\left.z\to 0,\ u\to \frac{aX\,rX}{1-bX\,rX},\ v\to \frac{aY\,(-aX\,rX\,\eta+\rho-bX\,rX\,\rho)}{-1+aX\,bY\,rX\,\eta-bY\,\rho+bX\,(rX+bY\,rX\,\rho)}\right\},$$

$$\left\{x\to \frac{aZ\,rZ^2\,\delta U\,(-1+\theta)\,\theta\,(aX-aX\,bX\,rZ\,\theta+aZ\,bX\,rZ\,\theta)}{(-1+bX\,rZ\,\theta)\,(aX\,(-1+bX\,rZ\,\theta)\,(rZ\,(-1+\theta)+rX\,\kappa)+aZ\,rZ\,\theta\,(\kappa+bX\,(rZ-rZ\,\theta-rX\,\kappa)))\,\phi},\right.$$

$$y\to 0,\ z\to \frac{aZ\,rZ\,\delta U\,\theta\,(aZ\,(-1+bX\,rX)\,rZ\,\theta+aX\,(rX-bX\,rX\,rZ\,\theta))}{(-1+bX\,rZ\,\theta)\,(aX\,(-1+bX\,rZ\,\theta)\,(rZ\,(-1+\theta)+rX\,\kappa)+aZ\,rZ\,\theta\,(\kappa+bX\,(rZ-rZ\,\theta-rX\,\kappa)))\,\phi},$$

$$u\to \frac{aZ\,rZ\,\theta}{1-bX\,rZ\,\theta},$$

$$\left.v\to \frac{aZ\,rZ\,\beta Z\,\delta U\,\theta\,(aZ\,(-1+bX\,rX)\,rZ\,\theta+aX\,(rX-bX\,rX\,rZ\,\theta))}{\delta V\,(-1+bX\,rZ\,\theta)\,(aX\,(-1+bX\,rZ\,\theta)\,(rZ\,(-1+\theta)+rX\,\kappa)+aZ\,rZ\,\theta\,(\kappa+bX\,(rZ-rZ\,\theta-rX\,\kappa)))\,\phi}\right\},$$

$$\Big\{x\to -\Big(\Big(aZ\,rZ^2\,(-1+\theta)\,\theta\,(aX-aX\,bX\,rZ\,\theta+aZ\,bX\,rZ\,\theta)\,(aY\,\delta V\,\xi\,(-aZ\,rZ\,\eta\,\theta+\rho-bX\,rZ\,\theta\,\rho)+\beta Y\,\delta U$$
$$(-1+aZ\,bY\,rZ\,\eta\,\theta-bY\,\rho+bX\,rZ\,\theta\,(1+bY\,\rho)))\Big)\Big/\Big((-1+aZ\,bY\,rZ\,\eta\,\theta-bY\,\rho+bX\,rZ\,\theta\,(1+bY\,\rho))$$
$$(aX\,(-1+bX\,rZ\,\theta)\,(aZ\,rX\,rZ\,\beta Z\,\theta\,\xi-\beta Y\,(-1+bX\,rZ\,\theta)\,(rZ\,(-1+\theta)+rX\,\kappa)\,\phi)+$$
$$aZ\,rZ\,\theta\,(aZ\,(1-bX\,rX)\,rZ\,\beta Z\,\theta\,\xi+\beta Y\,(-1+bX\,rZ\,\theta)\,(bX\,rZ\,(-1+\theta)-\kappa+bX\,rX\,\kappa)\,\phi))\Big)\Big),$$

$$y\to \Big(-aX\,(-1+bX\,rZ\,\theta)\,\Big(aZ^2\,bY\,rX\,rZ^2\,\beta Z\,\delta U\,\eta\,\theta^2-aY\,\delta V\,(-1+bX\,rZ\,\theta)^2\,(rZ\,(-1+\theta)+rX\,\kappa)\,\rho\,\phi+$$
$$aZ\,rZ\,\theta\,(-1+bX\,rZ\,\theta)\,(rX\,\beta Z\,(\delta U+bY\,\delta U\,\rho)-aY\,rZ\,\delta V\,\eta\,(-1+\theta)\,\phi-aY\,rX\,\delta V\,\eta\,\kappa\,\phi)\Big)+$$
$$aZ\,rZ\,\theta\,\Big(aZ^2\,bY\,(-1+bX\,rX)\,rZ^2\,\beta Z\,\delta U\,\eta\,\theta^2-aY\,\delta V\,(-1+bX\,rZ\,\theta)^2\,(bX\,rZ\,(-1+\theta)-\kappa+bX\,rX\,\kappa)\,\rho\,\phi+$$
$$aZ\,rZ\,\theta\,(-1+bX\,rZ\,\theta)\,((-1+bX\,rX)\,\beta Z\,\delta U\,(1+bY\,\rho)+aY\,\delta V\,\eta\,(\kappa+bX\,(rZ-rZ\,\theta-rX\,\kappa))\,\phi)\Big)\Big)\Big/$$
$$((-1+aZ\,bY\,rZ\,\eta\,\theta-bY\,\rho+bX\,rZ\,\theta\,(1+bY\,\rho))$$
$$(aX\,(-1+bX\,rZ\,\theta)\,(aZ\,rX\,rZ\,\beta Z\,\theta\,\xi-\beta Y\,(-1+bX\,rZ\,\theta)\,(rZ\,(-1+\theta)+rX\,\kappa)\,\phi)+$$
$$aZ\,rZ\,\theta\,(aZ\,(1-bX\,rX)\,rZ\,\beta Z\,\theta\,\xi+\beta Y\,(-1+bX\,rZ\,\theta)\,(bX\,rZ\,(-1+\theta)-\kappa+bX\,rX\,\kappa)\,\phi))),$$

$$z\to -((aZ\,rZ\,\theta\,(aZ\,(-1+bX\,rX)\,rZ\,\theta+aX\,(rX-bX\,rX\,rZ\,\theta))\,(aY\,\delta V\,\xi\,(-aZ\,rZ\,\eta\,\theta+\rho-bX\,rZ\,\theta\,\rho)+\beta Y$$
$$\delta U\,(-1+aZ\,bY\,rZ\,\eta\,\theta-bY\,\rho+bX\,rZ\,\theta\,(1+bY\,\rho))))\,/\,((-1+aZ\,bY\,rZ\,\eta\,\theta-bY\,\rho+bX\,rZ\,\theta\,(1+bY\,\rho))$$
$$(aX\,(-1+bX\,rZ\,\theta)\,(aZ\,rX\,rZ\,\beta Z\,\theta\,\xi-\beta Y\,(-1+bX\,rZ\,\theta)\,(rZ\,(-1+\theta)+rX\,\kappa)\,\phi)+$$
$$aZ\,rZ\,\theta\,(aZ\,(1-bX\,rX)\,rZ\,\beta Z\,\theta\,\xi+\beta Y\,(-1+bX\,rZ\,\theta)\,(bX\,rZ\,(-1+\theta)-\kappa+bX\,rX\,\kappa)\,\phi)))),$$

$$\left.u\to \frac{aZ\,rZ\,\theta}{1-bX\,rZ\,\theta},\ v\to \frac{aY\,(-aZ\,rZ\,\eta\,\theta+\rho-bX\,rZ\,\theta\,\rho)}{-1+aZ\,bY\,rZ\,\eta\,\theta-bY\,\rho+bX\,rZ\,\theta\,(1+bY\,\rho)}\right\},\ \{x\to 0,$$

$$y\to -\frac{\delta U}{\xi},$$

$$z\to 0,$$

$$u\to \frac{\beta Y\,\delta U+bY\,\beta Y\,\delta U\,\rho-aY\,\delta V\,\xi\,\rho}{bY\,\beta Y\,\delta U\,\eta-aY\,\delta V\,\eta\,\xi},$$

$$\left.v\to -\frac{\beta Y\,\delta U}{\delta V\,\xi}\right\},\ \{x\to 0,$$

$$y\to 0,$$
$$z\to 0,$$
$$u\to 0,$$
$$v\to 0\},$$

$$\left\{x\to \frac{aX\,rX\,\delta U}{\phi-bX\,rX\,\phi},\right.$$

$$y\to 0,$$
$$z\to 0,$$

$$u\to \frac{aX\,rX}{1-bX\,rX},$$

$$\left.\left.v\to 0\right\}\right\}$$

In[•]:=
```
(*defining fixed points*)
E0 = {0, 0, 0, 0, 0};

E1 = {(aX rX δU)/(ϕ - bX rX ϕ), 0, 0, (aX rX)/(1 - bX rX), 0};

E2 = {(aX rX (-δU + (aY δV ξ (aX rX η + (-1+bX rX) ρ))/(βY (-1+aX bY rX η-bY ρ+bX (rX+bY rX ρ)))))/((-1 + bX rX) ϕ),
   (aY δV (-aX rX η + ρ - bX rX ρ))/(βY (-1 + aX bY rX η - bY ρ + bX (rX + bY rX ρ))), 0, (aX rX)/(1 - bX rX),
   (aY (-aX rX η + ρ - bX rX ρ))/(-1 + aX bY rX η - bY ρ + bX (rX + bY rX ρ))};

EM = {(aZ rZ^2 δU (-1 + θ) θ (aX - aX bX rZ θ + aZ bX rZ θ))/((-1 + bX rZ θ) (aX (-1 + bX rZ θ) (rZ (-1 + θ) + rX κ) + aZ rZ θ (κ + bX (rZ - rZ θ - rX κ))) ϕ), 0,
   (aZ rZ δU θ (aZ (-1 + bX rX) rZ θ + aX (rX - bX rX rZ θ)))/((-1 + bX rZ θ) (aX (-1 + bX rZ θ) (rZ (-1 + θ) + rX κ) + aZ rZ θ (κ + bX (rZ - rZ θ - rX κ))) ϕ), (aZ rZ θ)/(1 - bX rZ θ),
   (aZ rZ βZ δU θ (aZ (-1 + bX rX) rZ θ + aX (rX - bX rX rZ θ)))/(δV (-1 + bX rZ θ) (aX (-1 + bX rZ θ) (rZ (-1 + θ) + rX κ) + aZ rZ θ (κ + bX (rZ - rZ θ - rX κ))) ϕ)};

EP = {-((aZ rZ^2 (-1 + θ) θ (aX - aX bX rZ θ + aZ bX rZ θ)
        (aY δV ξ (-aZ rZ η θ + ρ - bX rZ θ ρ) + βY δU (-1 + aZ bY rZ η θ - bY ρ + bX rZ θ (1 + bY ρ)))) /
      ((-1 + aZ bY rZ η θ - bY ρ + bX rZ θ (1 + bY ρ))
        (aX (-1 + bX rZ θ) (aZ rX rZ βZ θ ξ - βY (-1 + bX rZ θ) (rZ (-1 + θ) + rX κ) ϕ) +
          aZ rZ θ (aZ (1 - bX rX) rZ βZ θ ξ + βY (-1 + bX rZ θ) (bX rZ (-1 + θ) - κ + bX rX κ) ϕ)))),
   (-aX (-1 + bX rZ θ) (aZ^2 bY rX rZ^2 βZ δU η θ^2 - aY δV (-1 + bX rZ θ)^2 (rZ (-1 + θ) + rX κ) ρ ϕ +
          aZ rZ θ (-1 + bX rZ θ) (rX βZ (δU + bY δU ρ) - aY rZ δV η (-1 + θ) ϕ - aY rX δV η κ ϕ)) +
      aZ rZ θ (aZ^2 bY (-1 + bX rX) rZ^2 βZ δU η θ^2 - aY δV (-1 + bX rZ θ)^2 (bX rZ (-1 + θ) - κ + bX rX κ) ρ ϕ +
          aZ rZ θ (-1 + bX rZ θ) ((-1 + bX rX) βZ δU (1 + bY ρ) + aY δV η (κ + bX (rZ - rZ θ - rX κ)) ϕ))) /
    ((-1 + aZ bY rZ η θ - bY ρ + bX rZ θ (1 + bY ρ)) (aX (-1 + bX rZ θ)
         (aZ rX rZ βZ θ ξ - βY (-1 + bX rZ θ) (rZ (-1 + θ) + rX κ) ϕ) +
        aZ rZ θ (aZ (1 - bX rX) rZ βZ θ ξ + βY (-1 + bX rZ θ) (bX rZ (-1 + θ) - κ + bX rX κ) ϕ))),
  -((aZ rZ θ (aZ (-1 + bX rX) rZ θ + aX (rX - bX rX rZ θ)) (aY δV ξ (-aZ rZ η θ + ρ - bX rZ θ ρ) + βY
          δU (-1 + aZ bY rZ η θ - bY ρ + bX rZ θ (1 + bY ρ)))) / ((-1 + aZ bY rZ η θ - bY ρ + bX rZ θ (1 + bY ρ))
      (aX (-1 + bX rZ θ) (aZ rX rZ βZ θ ξ - βY (-1 + bX rZ θ) (rZ (-1 + θ) + rX κ) ϕ) +
        aZ rZ θ (aZ (1 - bX rX) rZ βZ θ ξ + βY (-1 + bX rZ θ) (bX rZ (-1 + θ) - κ + bX rX κ) ϕ)))),
  (aZ rZ θ)/(1 - bX rZ θ), (aY (-aZ rZ η θ + ρ - bX rZ θ ρ))/(-1 + aZ bY rZ η θ - bY ρ + bX rZ θ (1 + bY ρ))};
```

In[•]:=
```
(*x@EM = 0 if and only if θ = 1*)
Reduce[Extract[EM, {1}] == 0 && (1 - bX * rZ) > 0 && (1 - bX * rX) > 0 && aZ > aX > 0 && βY > 0 && βZ > 0 &&
  δU > 0 && δV > 0 && rX > rZ > 0 && bX > 0 && bY > 0 && aY > 0 && κ > 0 && η > 0 && ρ > 0 && ϕ > 0 && ξ > 0 && θ > 0,
 {rX, aX, aY, βY, βZ, δU, δV, rZ, bX, bY, aZ, κ, η , ρ, ϕ, ξ, θ}, Reals]
```

Out[•]=

$$\left(rX > 0 \,\&\&\, aX > 0 \,\&\&\, aY > 0 \,\&\&\, \beta Y > 0 \,\&\&\, \beta Z > 0 \,\&\&\, \delta U > 0 \,\&\&\, \delta V > 0 \,\&\&\, 0 < rZ < rX \,\&\&\, 0 < bX < \frac{1}{rX} \,\&\&\, bY > 0 \,\&\&\, aX < aZ < \frac{-aX\,rX + aX\,bX\,rX\,rZ}{-rZ + bX\,rX\,rZ} \,\&\&\, \kappa > 0 \,\&\&\, \eta > 0 \,\&\&\, \rho > 0 \,\&\&\, \phi > 0 \,\&\&\, \xi > 0 \,\&\&\, \theta == 1\right) \;||$$

$$\left(rX > 0 \,\&\&\, aX > 0 \,\&\&\, aY > 0 \,\&\&\, \beta Y > 0 \,\&\&\, \beta Z > 0 \,\&\&\, \delta U > 0 \,\&\&\, \delta V > 0 \,\&\&\, 0 < rZ < rX \,\&\&\, 0 < bX < \frac{1}{rX} \,\&\&\, bY > 0 \,\&\&\, aZ > \frac{-aX\,rX + aX\,bX\,rX\,rZ}{-rZ + bX\,rX\,rZ} \,\&\&\, \kappa > 0 \,\&\&\, \eta > 0 \,\&\&\, \rho > 0 \,\&\&\, \phi > 0 \,\&\&\, \xi > 0 \,\&\&\, \theta == 1\right)$$

```
In[•]:= (*arbitrary parameter values with varied θ*)
para1 = {w → 500, kX → 0.5, cX → 3, lX → 4, dX → 0.33, kY → 0.1, Ω → 10, cY → 0.3,
   lY → 1.2, dY → 0.1, aX → 1, bX → 0.01, aY → 1.5, bY → 0.015, δU → 1, δV → 1, βY → 2,
   lZ → 5, cZ → 4, dZ → 0.4, n → 2, pZ → 0.2, kYZ → 0.06, βZ → 4, s → 0.4, θ → 0.75};

para2 = {w → 500, kX → 0.5, cX → 3, lX → 4, dX → 0.33, kY → 0.1, Ω → 10, cY → 0.3,
   lY → 1.2, dY → 0.1, aX → 1, bX → 0.01, aY → 1.5, bY → 0.015, δU → 1, δV → 1, βY → 2,
   lZ → 5, cZ → 4, dZ → 0.4, n → 2, pZ → 0.2, kYZ → 0.06, βZ → 4, s → 0.4, θ → 0.95};

para3 = {w → 500, kX → 0.5, cX → 3, lX → 4, dX → 0.33, kY → 0.1, Ω → 10, cY → 0.3,
   lY → 1.2, dY → 0.1, aX → 1, bX → 0.01, aY → 1.5, bY → 0.015, δU → 1, δV → 1, βY → 2,
   lZ → 5, cZ → 4, dZ → 0.4, n → 2, pZ → 0.2, kYZ → 0.06, βZ → 4, s → 0.4, θ → 1.0};

(*substitutions to simplify the system*)
aZ = aX / (1 - n * s);
κ = (1 - kYZ * n) * (1 - n * pZ);
kZ = kX * (1 - n * pZ);
rX = (kX * w - lX) / cX * (N[Log[2]]) - dX;
rZ = (kZ * w - lZ) / cZ * (N[Log[2]]) - dZ;
η = kY / (Ω * cY) * (N[Log[2]]);
ρ = lY / cY * (N[Log[2]]) + dY;
ϕ = kX * w;
ξ = kY / Ω;

(*dynamical system with the vertical inheritance
 parameter θ as in Appendix A4.3 in S1 Appendix*)
dynamics = {
   x'[t] == x[t] * (rX - u[t] / (aX + bX * u[t])) + (1 - θ) * rZ * z[t],
   y'[t] == y[t] * (η * u[t] - ρ - v[t] / (aY + bY * v[t])),
   z'[t] == z[t] * (θ * rZ - u[t] / (aZ + bX * u[t])),
   u'[t] == ϕ * x[t] + κ * ϕ * z[t] - ξ * u[t] * y[t] - δU * u[t],
   v'[t] == βY * y[t] + βZ * z[t] - δV * v[t]
  };
```

In[∘]:=
```
(*numerical fixed points for θ = 0.75*)
f1[x_, y_, z_, u_, v_] := x * (rX - u/(aX + bX * u)) + (1 - θ) * rZ * z /. para1;
f2[x_, y_, z_, u_, v_] := y * (η * u - ρ - v/(aY + bY * v)) /. para1;
f3[x_, y_, z_, u_, v_] := z * (θ * rZ - u/(aZ + bX * u)) /. para1;
f4[x_, y_, z_, u_, v_] := ϕ * x + κ * ϕ * z - ξ * u * y - δU * u /. para1;
f5[x_, y_, z_, u_, v_] := βY * y + βZ * z - δV * v /. para1;
Simplify[NSolve[{f1[x, y, z, u, v] == 0, f2[x, y, z, u, v] == 0, f3[x, y, z, u, v] == 0,
   f4[x, y, z, u, v] == 0, f5[x, y, z, u, v] == 0, x ≥ 0, y ≥ 0, z ≥ 0, u ≥ 0, v ≥ 0}, {x, y, z, u, v}]]
```

Out[∘]=
```
{{x → 0, v → 0, z → 0, y → 0, u → 0}, {x → 0.519711, v → 0, z → 0, y → 0, u → 129.928},
 {x → 0.520216, v → 0.194445, z → 0, y → 0.0972227, u → 129.928}}
```

In[∘]:=
```
E0 = {0, 0, 0, 0, 0}
```

$$E1 = \left\{\frac{aX\, rX\, \delta U}{\phi - bX\, rX\, \phi}, 0, 0, \frac{aX\, rX}{1 - bX\, rX}, 0\right\} /.\ para1$$

$$E2 = \left\{\frac{aX\, rX\left(-\delta U + \frac{aY\, \delta V\, \xi\, (aX\, rX\, \eta + (-1 + bX\, rX)\, \rho)}{\beta Y\, (-1 + aX\, bY\, rX\, \eta - bY\, \rho + bX\, (rX + bY\, rX\, \rho))}\right)}{(-1 + bX\, rX)\, \phi}, \frac{aY\, \delta V\, (-aX\, rX\, \eta + \rho - bX\, rX\, \rho)}{\beta Y\, (-1 + aX\, bY\, rX\, \eta - bY\, \rho + bX\, (rX + bY\, rX\, \rho))},\right.$$
$$\left. 0, \frac{aX\, rX}{1 - bX\, rX}, \frac{aY\, (-aX\, rX\, \eta + \rho - bX\, rX\, \rho)}{-1 + aX\, bY\, rX\, \eta - bY\, \rho + bX\, (rX + bY\, rX\, \rho)}\right\} /.\ para1$$

Out[∘]=
```
{0, 0, 0, 0, 0}
```

Out[∘]=
```
{0.519711, 0, 0, 129.928, 0}
```

Out[∘]=
```
{0.520216, 0.0972227, 0, 129.928, 0.194445}
```

In[∘]:=
```
jacobian[{x_, y_, z_, u_, v_}] =
  FullSimplify[D[{f1[x, y, z, u, v], f2[x, y, z, u, v], f3[x, y, z, u, v],
     f4[x, y, z, u, v], f5[x, y, z, u, v]}, {{x, y, z, u, v}}]];
```

In[∘]:=
```
Eigenvalues[jacobian[{x, y, z, u, v} /. {{x, y, z, u, v} → E0}]]
(*unstable*)
```

Out[∘]=
```
{56.5081, 18.5449, -2.87259, -1., -1.}
```

In[∘]:=
```
Eigenvalues[jacobian[{x, y, z, u, v} /. {{x, y, z, u, v} → E1}]]
(*unstable*)
```

Out[∘]=
```
{-0.5 + 4.93219 i, -0.5 - 4.93219 i, -2.08087 + 0. i, -1. + 0. i, 0.129379 + 0. i}
```

In[∘]:=
```
Eigenvalues[jacobian[{x, y, z, u, v} /. {{x, y, z, u, v} → E2}]]
(*stable*)
```

Out[∘]=
```
{-0.500486 + 4.93485 i, -0.500486 - 4.93485 i, -2.08087 + 0. i, -0.847689 + 0. i, -0.152311 + 0. i}
```

In[•]:=
```
Dsol1 =
  ParametricNDSolveValue[{dynamics /. para1, x[0] == x0, y[0] == y0, z[0] == z0, u[0] == u0, v[0] == v0},
   {x[t], y[t], z[t]}, {t, 0, 100}, {x0, y0, z0, u0, v0}];(*θ = 0.75*)

ic0 = Dsol1[0.01, 0.01, 0.01, 0.01, 0.01];
ic1 = Dsol1[0.53, 0.01, 0.01, 131, 0.01];
ic2 = Dsol1[0.53, 0.1, 0.01, 131, 0.2];

θ1 =
  Plot[Evaluate[Join[ic0, ic1, ic2]], {t, 0, 100}, ScalingFunctions → "Log", PlotPoints → 300,

   PlotStyle → {Directive[RGBColor[0.24, 0.6, 0.8], Thickness[0.008]],
     Directive[RGBColor[0.95, 0.627, 0.1425], Thickness[0.008]],
     Directive[RGBColor[0.455, 0.7, 0.21], Thickness[0.008]]},

   PlotRange → {{-1.2, 41.2}, {0.0001, Automatic}}, Frame → True, FrameStyle →
    Directive[Black, Thickness[0.006], 18], FrameTicks → {{Automatic, None}, {All, None}},

   Epilog → {Inset[Text[Style["Free-living host, x", FontFamily → "Arial", Black, 21]], {30, 0.2}],
     Inset[Text[Style["Free-living symbiont, y", FontFamily → "Arial", Black, 21]], {27, -4}]},

   ImageSize → {400, 300}, ImagePadding → {{50, 15}, {20, 10}}, AspectRatio → 0.75];
```

In[•]:=
```
(*numerical fixed points for θ = 0.95*)
f1[x_, y_, z_, u_, v_] := x * (rX - u/(aX + bX * u)) + (1 - θ) * rZ * z /. para2;
f2[x_, y_, z_, u_, v_] := y * (η * u - ρ - v/(aY + bY * v)) /. para2;
f3[x_, y_, z_, u_, v_] := z * (θ * rZ - u/(aZ + bX * u)) /. para2;
f4[x_, y_, z_, u_, v_] := ϕ * x + κ * ϕ * z - ξ * u * y - δU * u /. para2;
f5[x_, y_, z_, u_, v_] := βY * y + βZ * z - δV * v /. para2;
Simplify[NSolve[{f1[x, y, z, u, v] == 0, f2[x, y, z, u, v] == 0, f3[x, y, z, u, v] == 0,
   f4[x, y, z, u, v] == 0, f5[x, y, z, u, v] == 0, x ≥ 0, y ≥ 0, z ≥ 0, u ≥ 0, v ≥ 0}, {x, y, z, u, v}]]
```

Out[•]=
```
{{x → 0, v → 0, z → 0, y → 0, u → 0}, {x → 0.519711, v → 0, z → 0, y → 0, u → 129.928},
 {x → 0.520216, v → 0.194445, z → 0, y → 0.0972227, u → 129.928},
 {x → 0.225096, v → 2.94659, z → 0.736647, y → 0, u → 153.512}}
```

In[•]:=
```
E0 = {0, 0, 0, 0, 0}
```

$$E1 = \left\{\frac{aX\,rX\,\delta U}{\phi - bX\,rX\,\phi}, 0, 0, \frac{aX\,rX}{1 - bX\,rX}, 0\right\} /.\ para2$$

$$E2 = \left\{\frac{aX\,rX\left(-\delta U + \frac{aY\,\delta V\,\xi\,(aX\,rX\,\eta + (-1 + bX\,rX)\,\rho)}{\beta Y\,(-1 + aX\,bY\,rX\,\eta - bY\,\rho + bX\,(rX + bY\,rX\,\rho))}\right)}{(-1 + bX\,rX)\,\phi}, \frac{aY\,\delta V\,(-aX\,rX\,\eta + \rho - bX\,rX\,\rho)}{\beta Y\,(-1 + aX\,bY\,rX\,\eta - bY\,\rho + bX\,(rX + bY\,rX\,\rho))}, 0, \frac{aX\,rX}{1 - bX\,rX}, \frac{aY\,(-aX\,rX\,\eta + \rho - bX\,rX\,\rho)}{-1 + aX\,bY\,rX\,\eta - bY\,\rho + bX\,(rX + bY\,rX\,\rho)}\right\} /.\ para2$$

$$EM = \left\{\frac{aZ\,rZ^2\,\delta U\,(-1 + \theta)\,\theta\,(aX - aX\,bX\,rZ\,\theta + aZ\,bX\,rZ\,\theta)}{(-1 + bX\,rZ\,\theta)\,(aX\,(-1 + bX\,rZ\,\theta)\,(rZ\,(-1 + \theta) + rX\,\kappa) + aZ\,rZ\,\theta\,(\kappa + bX\,(rZ - rZ\,\theta - rX\,\kappa)))\,\phi}, 0, \frac{aZ\,rZ\,\delta U\,\theta\,(aZ\,(-1 + bX\,rX)\,rZ\,\theta + aX\,(rX - bX\,rX\,rZ\,\theta))}{(-1 + bX\,rZ\,\theta)\,(aX\,(-1 + bX\,rZ\,\theta)\,(rZ\,(-1 + \theta) + rX\,\kappa) + aZ\,rZ\,\theta\,(\kappa + bX\,(rZ - rZ\,\theta - rX\,\kappa)))\,\phi}, \frac{aZ\,rZ\,\theta}{1 - bX\,rZ\,\theta}, \frac{aZ\,rZ\,\beta Z\,\delta U\,\theta\,(aZ\,(-1 + bX\,rX)\,rZ\,\theta + aX\,(rX - bX\,rX\,rZ\,\theta))}{\delta V\,(-1 + bX\,rZ\,\theta)\,(aX\,(-1 + bX\,rZ\,\theta)\,(rZ\,(-1 + \theta) + rX\,\kappa) + aZ\,rZ\,\theta\,(\kappa + bX\,(rZ - rZ\,\theta - rX\,\kappa)))\,\phi}\right\} /.\ para2$$

Out[•]=
```
{0, 0, 0, 0, 0}
```

Out[•]=
```
{0.519711, 0, 0, 129.928, 0}
```

Out[•]=
```
{0.520216, 0.0972227, 0, 129.928, 0.194445}
```

Out[•]=
```
{0.225096, 0, 0.736647, 153.512, 2.94659}
```

In[•]:=
```
jacobian[{x_, y_, z_, u_, v_}] =
  FullSimplify[D[{f1[x, y, z, u, v], f2[x, y, z, u, v], f3[x, y, z, u, v],
     f4[x, y, z, u, v], f5[x, y, z, u, v]}, {{x, y, z, u, v}}]];
```

In[•]:=
```
Eigenvalues[jacobian[{x, y, z, u, v} /. {{x, y, z, u, v} → E0}]]
(*unstable*)
```

Out[•]=
```
{56.5081, 23.4903, -2.87259, -1., -1.}
```

In[•]:=
```
Eigenvalues[jacobian[{x, y, z, u, v} /. {{x, y, z, u, v} → E1}]]
(*unstable*)
```

Out[•]=
```
{-0.5 + 4.93219 i, -0.5 - 4.93219 i, 2.86444 + 0. i, -1. + 0. i, 0.129379 + 0. i}
```

In[•]:=
```
Eigenvalues[jacobian[{x, y, z, u, v} /. {{x, y, z, u, v} → E2}]]
(*unstable*)
```

Out[•]=
```
{-0.500486 + 4.93485 i, -0.500486 - 4.93485 i, 2.86444 + 0. i, -0.847689 + 0. i, -0.152311 + 0. i}
```

In[•]:=
```
Eigenvalues[jacobian[{x, y, z, u, v} /. {{x, y, z, u, v} → EM}]]
(*stable*)
```

Out[•]=
```
{-0.643752 + 4.35117 i, -0.643752 - 4.35117 i, -3.75849 + 0. i, -1.23389 + 0. i, -1. + 0. i}
```

In[•]:=
```
(*plotting corresponding solutions for x, y, and z*)

Dsol2 =
  ParametricNDSolveValue[{dynamics /. para2, x[0] == x0, y[0] == y0, z[0] == z0, u[0] == u0, v[0] == v0},
   {x[t], y[t], z[t]}, {t, 0, 100}, {x0, y0, z0, u0, v0}];(*θ = 0.95*)

ic0 = Dsol2[0.01, 0.01, 0.01, 0.01, 0.01];
ic1 = Dsol2[0.53, 0.01, 0.01, 131, 0.01];
ic2 = Dsol2[0.53, 0.1, 0.01, 131, 0.2];

θ2 =
  Plot[Evaluate[Join[ic0, ic1, ic2]], {t, 0, 100}, ScalingFunctions → "Log",
   PlotPoints → 300, PlotStyle → {Directive[RGBColor[0.24, 0.6, 0.8], Thickness[0.008]],
     Directive[RGBColor[0.95, 0.627, 0.1425], Thickness[0.008]],
     Directive[RGBColor[0.455, 0.7, 0.21], Thickness[0.008]]},

   PlotRange → {{-1.2, 41.2}, {0.0001, Automatic}}, Frame → True, FrameStyle →
    Directive[Black, Thickness[0.006], 18], FrameTicks → {{Automatic, None}, {All, None}},

   Epilog → {Inset[Text[Style["Consortium, z", FontFamily → "Arial", Black, 21]], {32, 0.5}],
     Inset[Text[Style["Free-living host, x", FontFamily → "Arial", Black, 21]], {29.5, -2.4}],
     Inset[Text[Style["Free-living symbiont, y", FontFamily → "Arial", Black, 21]], {22, -8}]},

   ImageSize → {400, 300}, ImagePadding → {{50, 15}, {20, 10}}, AspectRatio → 0.75];
```

In[•]:=
```
(*numerical fixed points for θ = 1*)
f1[x_, y_, z_, u_, v_] := x * (rX - u/(aX + bX * u)) + (1 - θ) * rZ * z /. para3;
f2[x_, y_, z_, u_, v_] := y * (η * u - ρ - v/(aY + bY * v)) /. para3;
f3[x_, y_, z_, u_, v_] := z * (θ * rZ - u/(aZ + bX * u)) /. para3;
f4[x_, y_, z_, u_, v_] := ϕ * x + κ * ϕ * z - ξ * u * y - δU * u /. para3;
f5[x_, y_, z_, u_, v_] := βY * y + βZ * z - δV * v /. para3;
Simplify[NSolve[{f1[x, y, z, u, v] == 0, f2[x, y, z, u, v] == 0, f3[x, y, z, u, v] == 0,
   f4[x, y, z, u, v] == 0, f5[x, y, z, u, v] == 0, x ≥ 0, y ≥ 0, z ≥ 0, u ≥ 0, v ≥ 0}, {x, y, z, u, v}]]
```

Out[•]=
```
{{x → 0, z → 0, v → 0, y → 0, u → 0}, {x → 0.519711, z → 0, v → 0, y → 0, u → 129.928},
 {x → 0.520216, z → 0, v → 0.194445, y → 0.0972227, u → 129.928},
 {x → 0, z → 1.24428, v → 4.97713, y → 0, u → 164.245}}
```

In[•]:=

```
E0 = {0, 0, 0, 0, 0}
```

$$\text{E1} = \left\{\frac{\text{aX rX }\delta\text{U}}{\phi - \text{bX rX }\phi}, 0, 0, \frac{\text{aX rX}}{1 - \text{bX rX}}, 0\right\} \text{ /. para3}$$

$$\text{E2} = \left\{\frac{\text{aX rX}\left(-\delta\text{U} + \frac{\text{aY }\delta\text{V }\xi\text{ (aX rX }\eta + (-1+\text{bX rX})\ \rho)}{\beta\text{Y }(-1+\text{aX bY rX }\eta - \text{bY }\rho + \text{bX (rX+bY rX }\rho))}\right)}{(-1 + \text{bX rX})\ \phi}, \frac{\text{aY }\delta\text{V }(-\text{aX rX }\eta + \rho - \text{bX rX }\rho)}{\beta\text{Y }(-1 + \text{aX bY rX }\eta - \text{bY }\rho + \text{bX (rX + bY rX }\rho))},\right.$$
$$\left. 0, \frac{\text{aX rX}}{1 - \text{bX rX}}, \frac{\text{aY }(-\text{aX rX }\eta + \rho - \text{bX rX }\rho)}{-1 + \text{aX bY rX }\eta - \text{bY }\rho + \text{bX (rX + bY rX }\rho)}\right\} \text{ /. para3}$$

$$\text{EM} = \left\{\frac{\text{aZ rZ}^2\ \delta\text{U }(-1 + \theta)\ \theta\ (\text{aX} - \text{aX bX rZ }\theta + \text{aZ bX rZ }\theta)}{(-1 + \text{bX rZ }\theta)\ (\text{aX }(-1 + \text{bX rZ }\theta)\ (\text{rZ }(-1 + \theta) + \text{rX }\kappa) + \text{aZ rZ }\theta\ (\kappa + \text{bX (rZ} - \text{rZ }\theta - \text{rX }\kappa)))\ \phi}, 0,\right.$$
$$\frac{\text{aZ rZ }\delta\text{U }\theta\ (\text{aZ }(-1 + \text{bX rX})\text{ rZ }\theta + \text{aX (rX} - \text{bX rX rZ }\theta))}{(-1 + \text{bX rZ }\theta)\ (\text{aX }(-1 + \text{bX rZ }\theta)\ (\text{rZ }(-1 + \theta) + \text{rX }\kappa) + \text{aZ rZ }\theta\ (\kappa + \text{bX (rZ} - \text{rZ }\theta - \text{rX }\kappa)))\ \phi}, \frac{\text{aZ rZ }\theta}{1 - \text{bX rZ }\theta},$$
$$\left.\frac{\text{aZ rZ }\beta\text{Z }\delta\text{U }\theta\ (\text{aZ }(-1 + \text{bX rX})\text{ rZ }\theta + \text{aX (rX} - \text{bX rX rZ }\theta))}{\delta\text{V }(-1 + \text{bX rZ }\theta)\ (\text{aX }(-1 + \text{bX rZ }\theta)\ (\text{rZ }(-1 + \theta) + \text{rX }\kappa) + \text{aZ rZ }\theta\ (\kappa + \text{bX (rZ} - \text{rZ }\theta - \text{rX }\kappa)))\ \phi}\right\} \text{ /. para3}$$

Out[•]=

```
{0, 0, 0, 0, 0}
```

Out[•]=

```
{0.519711, 0, 0, 129.928, 0}
```

Out[•]=

```
{0.520216, 0.0972227, 0, 129.928, 0.194445}
```

Out[•]=

```
{0., 0, 1.24428, 164.245, 4.97713}
```

In[•]:=

```
jacobian[{x_, y_, z_, u_, v_}] =
  FullSimplify[D[{f1[x, y, z, u, v], f2[x, y, z, u, v], f3[x, y, z, u, v],
     f4[x, y, z, u, v], f5[x, y, z, u, v]}, {{x, y, z, u, v}}]];
```

In[•]:=

```
Eigenvalues[jacobian[{x, y, z, u, v} /. {{x, y, z, u, v} → E0}]]
(*unstable*)
```

Out[•]=

```
{56.5081, 24.7266, -2.87259, -1., -1.}
```

In[•]:=

```
Eigenvalues[jacobian[{x, y, z, u, v} /. {{x, y, z, u, v} → E1}]]
(*unstable*)
```

Out[•]=

```
{-0.5 + 4.93219 ⅈ, -0.5 - 4.93219 ⅈ, 4.10077 + 0. ⅈ, -1. + 0. ⅈ, 0.129379 + 0. ⅈ}
```

In[•]:=

```
Eigenvalues[jacobian[{x, y, z, u, v} /. {{x, y, z, u, v} → E2}]]
(*unstable*)
```

Out[•]=

```
{-0.500486 + 4.93485 ⅈ, -0.500486 - 4.93485 ⅈ, 4.10077 + 0. ⅈ, -0.847689 + 0. ⅈ, -0.152311 + 0. ⅈ}
```

In[•]:=

```
Eigenvalues[jacobian[{x, y, z, u, v} /. {{x, y, z, u, v} → EM}]]
(*stable*)
```

Out[•]=

```
{-5.64828 + 0. ⅈ, -0.5 + 4.28515 ⅈ, -0.5 - 4.28515 ⅈ, -2.23849 + 0. ⅈ, -1. + 0. ⅈ}
```

```
In[•]:= (*plotting corresponding solutions for x, y, and z*)

Dsol3 =
  ParametricNDSolveValue[{dynamics /. para3, x[0] == x0, y[0] == y0, z[0] == z0, u[0] == u0, v[0] == v0},
   {x[t], y[t], z[t]}, {t, 0, 100}, {x0, y0, z0, u0, v0}];(*θ = 1*)

ic0 = Dsol3[0.01, 0.01, 0.01, 0.01, 0.01];
ic1 = Dsol3[0.53, 0.01, 0.01, 131, 0.01];
ic2 = Dsol3[0.53, 0.1, 0.01, 131, 0.2];

θ3 =
  Plot[Evaluate[Join[ic0, ic1, ic2]], {t, 0, 100}, ScalingFunctions → "Log", PlotPoints → 300,

   PlotStyle → {Directive[RGBColor[0.24, 0.6, 0.8], Thickness[0.008]],
     Directive[RGBColor[0.95, 0.627, 0.1425], Thickness[0.008]],
     Directive[RGBColor[0.455, 0.7, 0.21], Thickness[0.008]]},

   PlotRange → {{-1.2, 41.2}, {0.0001, Automatic}}, Frame → True, FrameStyle →
    Directive[Black, Thickness[0.006], 18], FrameTicks → {{Automatic, None}, {All, None}},

   Epilog → {Inset[Text[Style["Consortium, z", FontFamily → "Arial", Black, 21]], {32.5, 1}]},

   ImageSize → {400, 300}, ImagePadding → {{50, 15}, {20, 10}}, AspectRatio → 0.75];

In[•]:= (*Fig A3 in S1 Appendix*)
theta = Pane[Grid[
    MapThread[Prepend,
     {
      Append[
       Prepend[{{θ1, θ2, θ3}},
        {Style["θ = 0.75", FontFamily → "Arial", 24, Black], Style["θ = 0.95",
          FontFamily → "Arial", 24, Black], Style["θ = 1", FontFamily → "Arial", 24, Black]}],

       {Style["Time", FontFamily → "Arial", 24, Black], Style["Time",
         FontFamily → "Arial", 24, Black], Style["Time", FontFamily → "Arial", 24, Black]}],

      {"", Rotate[Style["Population densities", FontFamily → "Arial", 24, Black], 90 Degree], ""}

     }], Spacings → {1, 0}], ImageMargins → {{15, 5}, {10, 10}}];

(*NOTE: Below the critical value (θ = 0.83),
resident-only fixed point is stable. Above the critical value,
consortium is stable. Only perfect vertical inheritance (θ = 1) gives extinction of both free-
 living populations in our model. See Appendix A4.3 in S1 Appendix.*)

In[•]:= SetDirectory[NotebookDirectory[]];
Export["FigA3.tiff", theta, ImageResolution → 600];
```

2.8. Analysis of inhibition-free system

```
In[•]:= ClearAll["`*"];
```

Fixed points and existence conditions

In[∘]:=
```
(*replacing self-inhibition with intraspecies competition as in Appendix A5 in S1 Appendix*)

f1[x_, y_, z_, u_, v_] := x * (rX - x - z);
f2[x_, y_, z_, u_, v_] := y * (η * u - ρ - y);
f3[x_, y_, z_, u_, v_] := z * (rZ - x - z);
f4[x_, y_, z_, u_, v_] := ϕ * x + κ * ϕ * z - ξ * u * y - δU * u;
f5[x_, y_, z_, u_, v_] := βY * y + βZ * z - δV * v;
Simplify[Solve[{f1[x, y, z, u, v] == 0 && f2[x, y, z, u, v] == 0 &&
    f3[x, y, z, u, v] == 0 && f4[x, y, z, u, v] == 0 && f5[x, y, z, u, v] == 0}, {x, y, z, u, v}]]
(*only five biologically feasible fixed points exist.*)
```

Out[∘]=

$$\Big\{\Big\{x\to 0,\ y\to -\rho,\ z\to 0,\ u\to 0,\ v\to -\frac{\beta Y\,\rho}{\delta V}\Big\},\ \Big\{x\to 0,\ y\to -\frac{\delta U}{\xi},\ z\to 0,\ u\to \frac{-\delta U+\xi\rho}{\eta\,\xi},\ v\to -\frac{\beta Y\,\delta U}{\delta V\,\xi}\Big\},$$

$$\Big\{x\to rX,\ y\to -\frac{\delta U+\xi\rho+\sqrt{(\delta U-\xi\rho)^2+4\,rX\,\eta\,\xi\,\phi}}{2\,\xi},\ z\to 0,\ u\to -\frac{\delta U-\xi\rho+\sqrt{(\delta U-\xi\rho)^2+4\,rX\,\eta\,\xi\,\phi}}{2\,\eta\,\xi},$$

$$v\to -\frac{\beta Y\left(\delta U+\xi\rho+\sqrt{(\delta U-\xi\rho)^2+4\,rX\,\eta\,\xi\,\phi}\right)}{2\,\delta V\,\xi}\Big\},\ \Big\{x\to rX,\ y\to -\frac{\delta U+\xi\rho-\sqrt{(\delta U-\xi\rho)^2+4\,rX\,\eta\,\xi\,\phi}}{2\,\xi},$$

$$z\to 0,\ u\to \frac{-\delta U+\xi\rho+\sqrt{(\delta U-\xi\rho)^2+4\,rX\,\eta\,\xi\,\phi}}{2\,\eta\,\xi},\ v\to -\frac{\beta Y\left(\delta U+\xi\rho-\sqrt{(\delta U-\xi\rho)^2+4\,rX\,\eta\,\xi\,\phi}\right)}{2\,\delta V\,\xi}\Big\},$$

$$\Big\{x\to 0,\ y\to -\frac{\delta U+\xi\rho+\sqrt{(\delta U-\xi\rho)^2+4\,rZ\,\eta\,\kappa\,\xi\,\phi}}{2\,\xi},\ z\to rZ,\ u\to -\frac{\delta U-\xi\rho+\sqrt{(\delta U-\xi\rho)^2+4\,rZ\,\eta\,\kappa\,\xi\,\phi}}{2\,\eta\,\xi},$$

$$v\to -\frac{\beta Y\,\delta U-2\,rZ\,\beta Z\,\xi+\beta Y\,\xi\,\rho+\beta Y\sqrt{(\delta U-\xi\rho)^2+4\,rZ\,\eta\,\kappa\,\xi\,\phi}}{2\,\delta V\,\xi}\Big\},$$

$$\Big\{x\to 0,\ y\to -\frac{\delta U+\xi\rho-\sqrt{(\delta U-\xi\rho)^2+4\,rZ\,\eta\,\kappa\,\xi\,\phi}}{2\,\xi},\ z\to rZ,\ u\to \frac{-\delta U+\xi\rho+\sqrt{(\delta U-\xi\rho)^2+4\,rZ\,\eta\,\kappa\,\xi\,\phi}}{2\,\eta\,\xi},$$

$$v\to \frac{-\beta Y\,\delta U+2\,rZ\,\beta Z\,\xi-\beta Y\,\xi\,\rho+\beta Y\sqrt{(\delta U-\xi\rho)^2+4\,rZ\,\eta\,\kappa\,\xi\,\phi}}{2\,\delta V\,\xi}\Big\},\ \{x\to 0,\ y\to 0,\ z\to 0,\ u\to 0,\ v\to 0\},$$

$$\Big\{x\to 0,\ y\to 0,\ z\to rZ,\ u\to \frac{rZ\,\kappa\,\phi}{\delta U},\ v\to \frac{rZ\,\beta Z}{\delta V}\Big\},\ \Big\{x\to rX,\ y\to 0,\ z\to 0,\ u\to \frac{rX\,\phi}{\delta U},\ v\to 0\Big\}\Big\}$$

In[∘]:=

Reduce[

$$-\frac{\delta U+\xi\rho+\sqrt{(\delta U-\xi\rho)^2+4\,rX\,\eta\,\xi\,\phi}}{2\,\xi}\ge 0\ \&\&\ -\frac{\delta U-\xi\rho+\sqrt{(\delta U-\xi\rho)^2+4\,rX\,\eta\,\xi\,\phi}}{2\,\eta\,\xi}\ge 0\ \&\&$$

$$-\frac{\beta Y\left(\delta U+\xi\rho+\sqrt{(\delta U-\xi\rho)^2+4\,rX\,\eta\,\xi\,\phi}\right)}{2\,\delta V\,\xi}\ge 0\ \&\&\ \beta Y>0\ \&\&\ \beta Z>0\ \&\&\ \delta U>0\ \&\&\ \delta V>0\ \&\&\ rX>rZ>0\ \&\&$$

κ > 0 && η > 0 && ρ > 0 && ϕ > 0 && ξ > 0, {rX, βY, βZ, δU, δV, rZ, κ, η , ρ, ϕ, ξ}, Reals]

Reduce[

$$-\frac{\delta U+\xi\rho+\sqrt{(\delta U-\xi\rho)^2+4\,rZ\,\eta\,\kappa\,\xi\,\phi}}{2\,\xi}\ge 0\ \&\&\ -\frac{\delta U-\xi\rho+\sqrt{(\delta U-\xi\rho)^2+4\,rZ\,\eta\,\kappa\,\xi\,\phi}}{2\,\eta\,\xi}\ge 0\ \&\&$$

$$-\frac{\beta Y\,\delta U-2\,rZ\,\beta Z\,\xi+\beta Y\,\xi\,\rho+\beta Y\sqrt{(\delta U-\xi\rho)^2+4\,rZ\,\eta\,\kappa\,\xi\,\phi}}{2\,\delta V\,\xi}\ge 0\ \&\&\ \beta Y>0\ \&\&\ \beta Z>0\ \&\&\ \delta U>0\ \&\&\ \delta V>0\ \&\&$$

rX > rZ > 0 && κ > 0 && η > 0 && ρ > 0 && ϕ > 0 && ξ > 0, {rX, βY, βZ, δU, δV, rZ, κ, η , ρ, ϕ, ξ}, Reals]

Out[∘]=
```
False
```

Out[∘]=
```
False
```

In[ ]:=
```
E0 = {0, 0, 0, 0, 0}(*trivial eq.*);
E1 = {rX, 0, 0, rX ϕ/δU, 0}(*one-species eq.*);
E2 = {rX, -(δU + ξ ρ - Sqrt[(δU - ξ ρ)^2 + 4 rX η ξ ϕ])/(2 ξ), 0, (-δU + ξ ρ + Sqrt[(δU - ξ ρ)^2 + 4 rX η ξ ϕ])/(2 η ξ),
  -(βY (δU + ξ ρ - Sqrt[(δU - ξ ρ)^2 + 4 rX η ξ ϕ]))/(2 δV ξ)}(*two-species resident eq.*);
EP = {0, -(δU + ξ ρ - Sqrt[(δU - ξ ρ)^2 + 4 rZ η κ ξ ϕ])/(2 ξ), rZ,
  (-δU + ξ ρ + Sqrt[(δU - ξ ρ)^2 + 4 rZ η κ ξ ϕ])/(2 η ξ), (-βY δU + 2 rZ βZ ξ - βY ξ ρ + βY Sqrt[(δU - ξ ρ)^2 + 4 rZ η κ ξ ϕ])/(2 δV ξ)}
  (* mutant eq. with free-living symbiont*);
EM = {0, 0, rZ, rZ κ ϕ/δU, rZ βZ/δV}(*consortium-only eq.*);
```

In[ ]:=
```
(*existence of E2*)
Reduce[
 Extract[E2, {2}] > 0 && Extract[E2, {4}] > 0 && Extract[E2, {5}] > 0 && βY > 0 && βZ > 0 && δU > 0 &&
  δV > 0 && rX > rZ > 0 && κ > 0 && η > 0 && ρ > 0 && ϕ > 0 && ξ > 0, {rX, βY, βZ, δU, δV, rZ, κ, η, ρ, ϕ, ξ}]
```

Out[ ]=

$$rX > 0 \,\&\&\, \beta Y > 0 \,\&\&\, \beta Z > 0 \,\&\&\, \delta U > 0 \,\&\&\, \delta V > 0 \,\&\&\, 0 < rZ < rX \,\&\&\, \kappa > 0 \,\&\&\, \eta > 0 \,\&\&\, \rho > 0 \,\&\&\, \phi > \frac{\delta U\, \rho}{rX\, \eta} \,\&\&\, \xi > 0$$

In[ ]:=
```
(*existence of EP*)
Reduce[
 Extract[EP, {2}] > 0 && Extract[EP, {4}] > 0 && Extract[EP, {5}] > 0 && βY > 0 && βZ > 0 && δU > 0 &&
  δV > 0 && rX > rZ > 0 && κ > 0 && η > 0 && ρ > 0 && ϕ > 0 && ξ > 0, {rX, βY, βZ, δU, δV, rZ, κ, η, ρ, ϕ, ξ}]
```

Out[ ]=

$$rX > 0 \,\&\&\, \beta Y > 0 \,\&\&\, \beta Z > 0 \,\&\&\, \delta U > 0 \,\&\&\, \delta V > 0 \,\&\&\, 0 < rZ < rX \,\&\&\, \kappa > 0 \,\&\&\, \eta > 0 \,\&\&\, \rho > 0 \,\&\&\, \phi > \frac{\delta U\, \rho}{rZ\, \eta\, \kappa} \,\&\&\, \xi > 0$$

Linear stability in the competitive system

In[ ]:=
```
(*Jacobian matrix at arbitrary (x,y,z,u,v) *)
jacobian[{x_, y_, z_, u_, v_}] =
 Simplify[D[{f1[x, y, z, u, v], f2[x, y, z, u, v], f3[x, y, z, u, v], f4[x, y, z, u, v],
    f5[x, y, z, u, v]}, {{x, y, z, u, v}}]] // MatrixForm
```

Out[ ]//MatrixForm=

$$\begin{pmatrix} rX - 2x - z & 0 & -x & 0 & 0 \\ 0 & -2y + u\eta - \rho & 0 & y\eta & 0 \\ -z & 0 & rZ - x - 2z & 0 & 0 \\ \phi & -u\xi & \kappa\phi & -\delta U - y\xi & 0 \\ 0 & \beta Y & \beta Z & 0 & -\delta V \end{pmatrix}$$

In[ ]:=
```
jacobian[{x, y, z, u, v} /. {{x, y, z, u, v} → E0}]
Eigenvalues[%]
(*One eigenvalue is positive real; E0 is unstable.*)
```

Out[ ]//MatrixForm=

$$\begin{pmatrix} rX & 0 & 0 & 0 & 0 \\ 0 & -\rho & 0 & 0 & 0 \\ 0 & 0 & rZ & 0 & 0 \\ \phi & 0 & \kappa\phi & -\delta U & 0 \\ 0 & \beta Y & \beta Z & 0 & -\delta V \end{pmatrix}$$

Out[ ]=

$$\{rX, rZ, -\delta U, -\delta V, -\rho\}$$

In[•]:=
```
jacobian[{x, y, z, u, v} /. {{x, y, z, u, v} → E1}]
FullSimplify[Eigenvalues[%]]
(*One eigenvalue is positive real as -ρ+rX η ϕ/δU > 0 from existence of E2; E1 is unstable.*)
```

Out[•]//MatrixForm=

$$\begin{pmatrix} -rX & 0 & -rX & 0 & 0 \\ 0 & -\rho + \frac{rX\,\eta\,\phi}{\delta U} & 0 & 0 & 0 \\ 0 & 0 & -rX + rZ & 0 & 0 \\ \phi & -\frac{rX\,\xi\,\phi}{\delta U} & \kappa\,\phi & -\delta U & 0 \\ 0 & \beta Y & \beta Z & 0 & -\delta V \end{pmatrix}$$

Out[•]=

$$\left\{-rX,\ -rX + rZ,\ -\delta U,\ -\delta V,\ -\rho + \frac{rX\,\eta\,\phi}{\delta U}\right\}$$

In[•]:=
```
FullSimplify[jacobian[{x, y, z, u, v} /. {{x, y, z, u, v} → EP}]]
FullSimplify[Eigenvalues[%]]
(*One eigenvalue is positive real as rX > rZ; EP is unstable.*)
```

Out[•]//MatrixForm=

$$\begin{pmatrix} rX - rZ & 0 & 0 & 0 & 0 \\ 0 & \frac{\delta U + \xi\rho - \sqrt{(\delta U - \xi\rho)^2 + 4\,rZ\,\eta\kappa\xi\phi}}{2\xi} & 0 & -\frac{\eta\left(\delta U + \xi\rho - \sqrt{(\delta U - \xi\rho)^2 + 4\,rZ\,\eta\kappa\xi\phi}\right)}{2\xi} & 0 \\ -rZ & 0 & -rZ & 0 & 0 \\ \phi & -\frac{-\delta U + \xi\rho + \sqrt{(\delta U - \xi\rho)^2 + 4\,rZ\,\eta\kappa\xi\phi}}{2\eta} & \kappa\,\phi & \frac{1}{2}\left(-\delta U + \xi\rho - \sqrt{(\delta U - \xi\rho)^2 + 4\,rZ\,\eta\kappa\xi\phi}\right) & 0 \\ 0 & \beta Y & \beta Z & 0 & -\delta V \end{pmatrix}$$

Out[•]=

$$\Big\{rX - rZ,\ -rZ,\ -\delta V,$$
$$-\frac{1}{4\eta\xi}\Bigg(\delta U\,\eta\,(-1+\xi) - \eta\,\xi\,(1+\xi)\,\rho + \eta\sqrt{(\delta U - \xi\rho)^2 + 4\,rZ\,\eta\kappa\xi\phi} + \eta\,\xi\sqrt{(\delta U - \xi\rho)^2 + 4\,rZ\,\eta\kappa\xi\phi} +$$
$$\sqrt{\Bigg(\eta^2\bigg(\Big(\delta U\,(-1+\xi) + (1+\xi)\Big(-\xi\rho + \sqrt{(\delta U - \xi\rho)^2 + 4\,rZ\,\eta\kappa\xi\phi}\Big)\Big)^2 + 8\,\xi\Big(-\delta U^2 - 4\,rZ\,\eta\kappa\xi\phi +}$$
$$\xi\rho\Big(-\xi\rho + \sqrt{(\delta U - \xi\rho)^2 + 4\,rZ\,\eta\kappa\xi\phi}\Big) + \delta U\Big(2\xi\rho + \sqrt{(\delta U - \xi\rho)^2 + 4\,rZ\,\eta\kappa\xi\phi}\Big)\Big)\bigg)\Bigg)}\ \Bigg),$$
$$\frac{1}{4\eta\xi}\Bigg(\delta U\,\eta - \delta U\,\eta\,\xi + \eta\,\xi\,\rho + \eta\,\xi^2\rho - \eta\sqrt{(\delta U - \xi\rho)^2 + 4\,rZ\,\eta\kappa\xi\phi} - \eta\,\xi\sqrt{(\delta U - \xi\rho)^2 + 4\,rZ\,\eta\kappa\xi\phi} +$$
$$\sqrt{\Bigg(\eta^2\bigg(\Big(\delta U\,(-1+\xi) + (1+\xi)\Big(-\xi\rho + \sqrt{(\delta U - \xi\rho)^2 + 4\,rZ\,\eta\kappa\xi\phi}\Big)\Big)^2 + 8\,\xi\Big(-\delta U^2 - 4\,rZ\,\eta\kappa\xi\phi +}$$
$$\xi\rho\Big(-\xi\rho + \sqrt{(\delta U - \xi\rho)^2 + 4\,rZ\,\eta\kappa\xi\phi}\Big) + \delta U\Big(2\xi\rho + \sqrt{(\delta U - \xi\rho)^2 + 4\,rZ\,\eta\kappa\xi\phi}\Big)\Big)\bigg)\Bigg)}\ \Bigg)\Big\}$$

In[•]:=
```
FullSimplify[jacobian[{x, y, z, u, v} /. {{x, y, z, u, v} → EM}]]
FullSimplify[Eigenvalues[%]]
(*One eigenvalue is positive real as rX > rZ; EM is unstable.*)
```

Out[•]//MatrixForm=

$$\begin{pmatrix} rX - rZ & 0 & 0 & 0 & 0 \\ 0 & -\rho + \frac{rZ\,\eta\,\kappa\,\phi}{\delta U} & 0 & 0 & 0 \\ -rZ & 0 & -rZ & 0 & 0 \\ \phi & -\frac{rZ\,\kappa\,\xi\,\phi}{\delta U} & \kappa\,\phi & -\delta U & 0 \\ 0 & \beta Y & \beta Z & 0 & -\delta V \end{pmatrix}$$

Out[•]=

$$\left\{rX - rZ,\ -rZ,\ -\delta U,\ -\delta V,\ -\rho + \frac{rZ\,\eta\,\kappa\,\phi}{\delta U}\right\}$$

In[]:=

```
FullSimplify[jacobian[{x, y, z, u, v} /. {{x, y, z, u, v} → E2}]]
FullSimplify[Eigenvalues[%]]
```

Out[]//MatrixForm=

$$\begin{pmatrix} -rX & 0 & -rX & 0 & 0 \\ 0 & \frac{\delta U+\xi\rho-\sqrt{(\delta U-\xi\rho)^2+4\,rX\,\eta\,\xi\,\phi}}{2\xi} & 0 & -\frac{\eta\left(\delta U+\xi\rho-\sqrt{(\delta U-\xi\rho)^2+4\,rX\,\eta\,\xi\,\phi}\right)}{2\xi} & 0 \\ 0 & 0 & -rX+rZ & 0 & 0 \\ \phi & -\frac{-\delta U+\xi\rho+\sqrt{(\delta U-\xi\rho)^2+4\,rX\,\eta\,\xi\,\phi}}{2\eta} & \kappa\,\phi & \frac{1}{2}\left(-\delta U+\xi\rho-\sqrt{(\delta U-\xi\rho)^2+4\,rX\,\eta\,\xi\,\phi}\right) & 0 \\ 0 & \beta Y & \beta Z & 0 & -\delta V \end{pmatrix}$$

Out[]=

$$\Big\{-rX,\ -rX+rZ,\ -\delta V,$$

$$-\frac{1}{4\eta\xi}\Bigg(\delta U\,\eta\,(-1+\xi)-\eta\,\xi\,(1+\xi)\,\rho+\eta\sqrt{(\delta U-\xi\rho)^2+4\,rX\,\eta\,\xi\,\phi}+\eta\,\xi\sqrt{(\delta U-\xi\rho)^2+4\,rX\,\eta\,\xi\,\phi}+$$
$$\sqrt{\Bigg(\eta^2\Bigg(\Big(\delta U\,(-1+\xi)+(1+\xi)\Big(-\xi\rho+\sqrt{(\delta U-\xi\rho)^2+4\,rX\,\eta\,\xi\,\phi}\Big)\Big)^2+8\,\xi\Big(-\delta U^2-4\,rX\,\eta\,\xi\,\phi+}$$
$$\xi\rho\Big(-\xi\rho+\sqrt{(\delta U-\xi\rho)^2+4\,rX\,\eta\,\xi\,\phi}\Big)+\delta U\Big(2\,\xi\rho+\sqrt{(\delta U-\xi\rho)^2+4\,rX\,\eta\,\xi\,\phi}\Big)\Big)\Bigg)\Bigg)}\Bigg),$$

$$\frac{1}{4\eta\xi}\Bigg(\delta U\,\eta-\delta U\,\eta\,\xi+\eta\,\xi\,\rho+\eta\,\xi^2\rho-\eta\sqrt{(\delta U-\xi\rho)^2+4\,rX\,\eta\,\xi\,\phi}-\eta\,\xi\sqrt{(\delta U-\xi\rho)^2+4\,rX\,\eta\,\xi\,\phi}+$$
$$\sqrt{\Bigg(\eta^2\Bigg(\Big(\delta U\,(-1+\xi)+(1+\xi)\Big(-\xi\rho+\sqrt{(\delta U-\xi\rho)^2+4\,rX\,\eta\,\xi\,\phi}\Big)\Big)^2+8\,\xi\Big(-\delta U^2-4\,rX\,\eta\,\xi\,\phi+}$$
$$\xi\rho\Big(-\xi\rho+\sqrt{(\delta U-\xi\rho)^2+4\,rX\,\eta\,\xi\,\phi}\Big)+\delta U\Big(2\,\xi\rho+\sqrt{(\delta U-\xi\rho)^2+4\,rX\,\eta\,\xi\,\phi}\Big)\Big)\Bigg)\Bigg)}\Bigg)\Big\}$$

```
(*simplifying the elements of Jacobian matrix at E2 (m__) for easier calculations*)
```

In[]:=

```
(*m22 < 0*)
Reduce[
 (δU + ξ ρ - Sqrt[(δU - ξ ρ)^2 + 4 rX η ξ ϕ])/(2 ξ) ≥ 0 && ϕ > (δU * ρ)/(rX * η) && ϕ > (δU * ρ)/(rZ * η * κ) && βY > 0 && βZ > 0 && δU > 0 && δV > 0 &&
  rX > rZ > 0 && κ > 0 && η > 0 && ρ > 0 && ϕ > 0 && ξ > 0, {rX, βY, βZ, δU, δV, rZ, κ, η , ρ, ϕ, ξ}]
```

Out[]=

```
False
```

In[]:=

```
(*m24 > 0*)
Reduce[
 -(η (δU + ξ ρ - Sqrt[(δU - ξ ρ)^2 + 4 rX η ξ ϕ]))/(2 ξ) ≤ 0 && ϕ > (δU * ρ)/(rX * η) && ϕ > (δU * ρ)/(rZ * η * κ) && βY > 0 && βZ > 0 && δU > 0 &&
  δV > 0 && rX > rZ > 0 && κ > 0 && η > 0 && ρ > 0 && ϕ > 0 && ξ > 0, {rX, βY, βZ, δU, δV, rZ, κ, η , ρ, ϕ, ξ}]
```

Out[]=

```
False
```

In[]:=

```
(*m42 < 0*)
Reduce[
 -(-δU + ξ ρ + Sqrt[(δU - ξ ρ)^2 + 4 rX η ξ ϕ])/(2 η) ≥ 0 && ϕ > (δU * ρ)/(rX * η) && ϕ > (δU * ρ)/(rZ * η * κ) && βY > 0 && βZ > 0 && δU > 0 &&
  δV > 0 && rX > rZ > 0 && κ > 0 && η > 0 && ρ > 0 && ϕ > 0 && ξ > 0, {rX, βY, βZ, δU, δV, rZ, κ, η , ρ, ϕ, ξ}]
```

Out[]=

```
False
```

In[•]:=
```
(*m44 < 0*)
Reduce[
 1/2 (-δU + ξ ρ - Sqrt[(δU - ξ ρ)^2 + 4 rX η ξ ϕ]) ≥ 0 && ϕ > (δU * ρ)/(rX * η) && ϕ > (δU * ρ)/(rZ * η * κ) && βY > 0 && βZ > 0 && δU > 0 &&
  δV > 0 && rX > rZ > 0 && κ > 0 && η > 0 && ρ > 0 && ϕ > 0 && ξ > 0, {rX, βY, βZ, δU, δV, rZ, κ, η, ρ, ϕ, ξ}]
```

Out[•]=
```
False
```

In[•]:=
```
(*redefining the matrix with sign of elements such that all a__ > 0*)
FullSimplify[Eigenvalues[{{-a11, 0, -a13, 0, 0}, {0, -a22, 0, a24, 0},
   {0, 0, -a33, 0, 0}, {a41, -a42, a43, -a44, 0}, {0, a52, a53, 0 , -a55}}]]
```

Out[•]=

$$\left\{-a11,\ -a33,\ \frac{1}{2}\left(-a22-\sqrt{-4\,a24\,a42+(a22-a44)^2}-a44\right),\ \frac{1}{2}\left(-a22+\sqrt{-4\,a24\,a42+(a22-a44)^2}-a44\right),\ -a55\right\}$$

```
(*NOTE: All eigenvalues of Jacobian matrix at E2 have negative real part,
hence E2 is stable. The resident-only fixed point is stable always in the competitive system;
the mutant cannot invade the system.*)
```

Numerical analysis

In[•]:=
```
(*numerical analysis of the competitive system*)

para = {w → 500, kX → 0.5, cX → 3, lX → 4, dX → 0.33, kY → 0.1, cY → 0.3, lY → 1.2, dY → 0.1, Ω → 10,
   δU → 1, δV → 1, βY → 2, lZ → 5, cZ → 4, dZ → 0.4, n → 2, pZ → 0.2, kYZ → 0.06, βZ → 4};

κ = (1 - kYZ * n) * (1 - n * pZ);
kZ = kX * (1 - n * pZ);
rX = (kX * w - lX)/cX * (N[Log[2]]) - dX;
rZ = (kZ * w - lZ)/cZ * (N[Log[2]]) - dZ;
η = kY/(Ω * cY) * (N[Log[2]]);
ρ = lY/cY * (N[Log[2]]) + dY;
ϕ = kX * w;
ξ = kY/Ω;
```

In[•]:=
```
(*numerically solving the competitive system*)
f1[x_, y_, z_, u_, v_] := x * (rX - x - z) /. para;
f2[x_, y_, z_, u_, v_] := y * (η * u - ρ - y) /. para;
f3[x_, y_, z_, u_, v_] := z * (rZ - x - z) /. para;
f4[x_, y_, z_, u_, v_] := ϕ * x + κ * ϕ * z - ξ * u * y - δU * u /. para;
f5[x_, y_, z_, u_, v_] := βY * y + βZ * z - δV * v /. para;
Simplify[NSolve[{f1[x, y, z, u, v] == 0, f2[x, y, z, u, v] == 0, f3[x, y, z, u, v] == 0,
   f4[x, y, z, u, v] == 0, f5[x, y, z, u, v] == 0, x ≥ 0, y ≥ 0, z ≥ 0, u ≥ 0, v ≥ 0}, {x, y, z, u, v}]]
```

Out[•]=
```
{{x → 56.5081, y → 0., z → 0., u → 14127., v → 0.},
 {x → 56.5081, y → 135.643, z → 0., u → 5995.09, v → 271.287},
 {x → 0., y → 0., z → 24.7266, u → 3263.91, v → 98.9063},
 {x → 0., y → 48.0608, z → 24.7266, u → 2204.44, v → 195.028}, {x → 0., y → 0., z → 0., u → 0., v → 0.}}
```

In[ ]:=
```
(*fixed points*)
E0 = {0, 0, 0, 0, 0}
E1 = {rX, 0, 0, rX ϕ/δU, 0} /. para
E2 = {rX, -(δU + ξ ρ - Sqrt[(δU - ξ ρ)^2 + 4 rX η ξ ϕ])/(2 ξ), 0,
    (-δU + ξ ρ + Sqrt[(δU - ξ ρ)^2 + 4 rX η ξ ϕ])/(2 η ξ), -(βY (δU + ξ ρ - Sqrt[(δU - ξ ρ)^2 + 4 rX η ξ ϕ]))/(2 δV ξ)} /. para
EP = {0, -(δU + ξ ρ - Sqrt[(δU - ξ ρ)^2 + 4 rZ η κ ξ ϕ])/(2 ξ), rZ, (-δU + ξ ρ + Sqrt[(δU - ξ ρ)^2 + 4 rZ η κ ξ ϕ])/(2 η ξ),
    (-βY δU + 2 rZ βZ ξ - βY ξ ρ + βY Sqrt[(δU - ξ ρ)^2 + 4 rZ η κ ξ ϕ])/(2 δV ξ)} /. para
EM = {0, 0, rZ, rZ κ ϕ/δU, rZ βZ/δV} /. para
```

Out[ ]=
{0, 0, 0, 0, 0}

Out[ ]=
{56.5081, 0, 0, 14127., 0}

Out[ ]=
{56.5081, 135.643, 0, 5995.09, 271.287}

Out[ ]=
{0, 48.0608, 24.7266, 2204.44, 195.028}

Out[ ]=
{0, 0, 24.7266, 3263.91, 98.9063}

In[ ]:=
```
(*Jacobian matrix at arbitrary (x,y,z,u,v) *)
jacobian[{x_, y_, z_, u_, v_}] = Simplify[D[{f1[x, y, z, u, v], f2[x, y, z, u, v],
      f3[x, y, z, u, v], f4[x, y, z, u, v], f5[x, y, z, u, v]}, {{x, y, z, u, v}}]];
```

In[ ]:=
```
Eigenvalues[jacobian[{x, y, z, u, v} /. {{x, y, z, u, v} → E0}]]
(*unstable*)
```

Out[ ]=
{56.5081, 24.7266, -2.87259, -1., -1.}

In[ ]:=
```
Eigenvalues[jacobian[{x, y, z, u, v} /. {{x, y, z, u, v} → E1}]]
(*unstable*)
```

Out[ ]=
{323.531, -56.5081, -31.7815, -1., -1.}

In[ ]:=
```
Eigenvalues[jacobian[{x, y, z, u, v} /. {{x, y, z, u, v} → EM}]]
(*unstable*)
```

Out[ ]=
{72.5397, 31.7815, -24.7266, -1., -1.}

In[ ]:=
```
Eigenvalues[jacobian[{x, y, z, u, v} /. {{x, y, z, u, v} → EP}]]
(*unstable*)
```

Out[ ]=
{-47.5292, 31.7815, -24.7266, -2.0122, -1.}

In[ ]:=
```
Eigenvalues[jacobian[{x, y, z, u, v} /. {{x, y, z, u, v} → E2}]]
(*stable*)
```

Out[ ]=
{-134.218, -56.5081, -31.7815, -3.78131, -1.}
