# Supplementary material for: Host-initiated microbial association leads to stable ectosymbiosis in an ecological model
Source: PLoS Comput Biol. 2026 Sep 2;22(9):e1014699. doi: 10.1371/journal.pcbi.1014699 (PMC13537694; doi:10.1371/journal.pcbi.1014699)
Supplement: Peer review history

# Peer review: Decision Letter 0

**DOI:** 10.1371/journal.pcbi.1014699.r001

**Authors:** Virginia E. Pitzer, Christopher Rao

1 Dec 2025

Host-initiated microbial association leads to stable ectosymbiosis in an ecological model

PLOS Computational Biology

Dear Dr. Krishnan,

Thank you for submitting your manuscript to PLOS Computational Biology. After careful consideration, we feel that it has merit but does not fully meet PLOS Computational Biology's publication criteria as it currently stands. Therefore, we invite you to submit a revised version of the manuscript that addresses the points raised during the review process.

Please submit your revised manuscript by Jan 31 2026 11:59PM. If you will need more time than this to complete your revisions, please reply to this message or contact the journal office at ploscompbiol@plos.org. When you're ready to submit your revision, log on to https://www.editorialmanager.com/pcompbiol/ and select the 'Submissions Needing Revision' folder to locate your manuscript file.

Please include the following items when submitting your revised manuscript:

* A rebuttal letter that responds to each point raised by the editor and reviewer(s). You should upload this letter as a separate file labeled 'Response to Reviewers'. This file does not need to include responses to formatting updates and technical items listed in the 'Journal Requirements' section below.

* A marked-up copy of your manuscript that highlights changes made to the original version. You should upload this as a separate file labeled 'Revised Manuscript with Track Changes'.

* An unmarked version of your revised paper without tracked changes. You should upload this as a separate file labeled 'Manuscript'.

If you would like to make changes to your financial disclosure, competing interests statement, or data availability statement, please make these updates within the submission form at the time of resubmission. Guidelines for resubmitting your figure files are available below the reviewer comments at the end of this letter

We look forward to receiving your revised manuscript.

Kind regards,

Christopher Rao

Academic Editor

PLOS Computational Biology

Virginia Pitzer

Editor-in-Chief

PLOS Computational Biology

**Additional Editor Comments (if provided):**

**Journal Requirements:**

If the reviewer comments include a recommendation to cite specific previously published works, please review and evaluate these publications to determine whether they are relevant and should be cited. There is no requirement to cite these works unless the editor has indicated otherwise.

[Note: HTML markup is below. Please do not edit.]

**Reviewers' comments:**

Reviewer's Responses to Questions

**Comments to the Authors:**

**Please note here if the review is uploaded as an attachment.**

Reviewer #1: Manuscript: "Host-initiated microbial association leads to stable ectosymbiosis in an ecological model" (PCOMPBIOL-D-25-01620)

Authors: Nandakishor Krishnan, Istvan Zachar, Adam Kun, Chaitanya S. Gokhale, Jozsef Garay

Summary

The study analyzes when a free-living syntrophic pair can transition to a physically attached ectosymbiotic consortium. The modeling proceeds in three stages: (i) a self-inhibiting host; (ii) a two-species free-living syntrophy; (iii) a resident-mutant system where the mutant host forms consortia with altered growth and inhibition parameters. The central claim of the paper is that attachment lowers effective self-inhibition for the consortium phenotype (implemented via aZ) while imposing a cost through reduced resource uptake (for example, kZ < kX). Invasion conditions are written as inequalities comparing effective growth-inhibition terms (for example rZ aZ/(1 - rZ bX) vs rX aX/(1 - rX bX)). Numerical examples illustrate three outcomes: E2 (resident coexistence with free-living symbionts), EP (mixed free-living and attached), and EM (mutant-only). Parameters are presented as qualitative or illustrative; mathematics and code are provided in the SI. The narrative sometimes links these outcomes to obligate ectosymbiosis and steps toward endosymbiosis.

Recommendation: Major Revision

Major comments

1. Core mechanism and parameterization

The central claim is a geometric mechanism of reduced inhibition at the contact surface. This requires explicit biophysical grounding and separation from simpler consumption-only relief.

a) Provide a mechanism for reduced exposure at the membrane. If the effect is due to diffusion boundary layers or receptor saturation, state this and indicate how it maps to the equations.

b) Do not reuse bX by default. Introduce bZ and report sensitivity to bZ != bX. Conclusions should not hinge on keeping the inhibition shape parameter identical across phenotypes.

c) Test alternative shielding functions with mechanistic rationale. The current aZ = aX/(1 - n*s) assumes linear scaling of inhibition relief with unoccupied surface. Inhibition is plausibly a saturating function of exposed surface, for example because of boundary-layer limits to diffusion or receptor-site saturation. Please test aZ = aX/(1 - (n*s)^alpha) with alpha != 1 and report how invasion regions shift. This is not merely an arbitrary sensitivity test; it evaluates whether the conclusion depends on the assumed linear geometry versus a more biophysically plausible saturation.

d) Add a consumption-only control: hold aZ = aX (no geometric benefit) and increase local consumption terms (for example kYZ or βZ) to match the net benefit provided by surface shielding. If invasion persists, the mechanism is syntrophy-driven rather than geometry-driven; if invasion fails, the geometric hypothesis is necessary and should then be supported with empirical examples or plausible physics.

e) Clarify the sign logic by introducing a simple benefit factor if helpful (for example, bfac = 1 - n*s or 1 - (n*s)^alpha) and mapping it to the inhibition term.

f) Justify allowing s (benefit-per-symbiont) and sZ (cost-per-symbiont) to differ. As a geometric baseline, test sZ = s; if they remain independent, provide a biological rationale (for example, distinct membrane domains).

2. Ectosymbiont dynamics and inheritance: address the "fixed n" and passive vertical inheritance simplification

The model assumes a constant mean number of ectosymbionts per host (n) with "poor man's passive vertical inheritance," explicitly omitting symbiont birth-death-attachment-detachment dynamics and stochastic partitioning at division. Because reliable association and transmission are central to stabilizing ectosymbiosis, please assess robustness to variance in n (e.g., binomial partitioning) and discuss how explicit attachment/detachment or symbiont life cycles might affect invasion/stability. A minimal sensitivity analysis in SI would materially strengthen the argument.

3. Mixed equilibrium (EP), coexistence, and bistability

Provide explicit existence and local stability criteria for EP, or numerical Jacobians with eigenvalues. Justify the statement that no equilibrium with coexisting resident and mutant hosts exists. If an analytic argument is infeasible, provide systematic numerical evidence. Clarify whether EP can be bistable with E2 or EM; if history dependence occurs, the interpretation of outcomes should be adjusted.

4. Robustness and parameter sensitivity

Go beyond hand-picked parameter sets. Provide two-parameter maps quantifying the regions that yield E2, EP, and EM (for example, s vs n or m; k_YZ vs beta_Z; key cost and death parameters). Include sensitivity to the dilution factor Omega used for metabolite U. If invasion holds only when Omega is large but fails at Omega = 1, report this and interpret the biology. If viable regions are narrow, temper generality claims.

5. Adaptive Dynamics (AD), or temper evolutionary language

If the manuscript continues to make evolutionary claims, analyze selection on a relevant trait (for example, s, n, or m). In SI, include a one-trait PIP, classify singular points (convergence stability, ESS vs branching), and examine sensitivity to trade-off curvature (concave vs convex shielding and cost). If AD is not added, remove teleological phrasing and present results in ecological terms, for example, "the mutant can invade when ...", "the system converges to EM under conditions ...", without asserting evolutionary endpoints.

6. Frequency dependence, finite symbiont pool, and public-good logic

In the current model, free-living symbionts function as a public good: any host benefits from their consumption of the inhibitory metabolite. When mutant hosts attach symbionts, they privatize a shared benefit, indirectly harming residents. This tragedy-of-the-commons mechanism appears central to invasion yet is not articulated. Explicitly articulate this public-good privatization logic and at minimum discuss outcomes under a finite symbiont supply. Preferably add a simple extension with finite supply or attachment-rate dynamics (numerical simulations are acceptable if analytical treatment is intractable) to test necessity: does invasion rely on privatizing a limited partner pool, or does it occur even when symbiont availability is effectively unlimited?

7. Scope, definitions, and claims

Define carefully:

* obligate metabolic dependence (cannot grow without the partner metabolite)

* obligate physical association (cannot reproduce without attachment)

* population-level obligate ectosymbiosis (free-living types extinct in the population)

* genotype-level obligate symbiosis (loss of autonomy at the genotype level, typically requiring genetic loss of autonomous functions over evolutionary time)

As formulated, EM reflects population-level ecological exclusion rather than genotype-level obligacy. Claims linking to eukaryogenesis should be tempered; without internalization, regulated inheritance, or genome reduction, the model is best positioned as a general theory for conditions favoring attachment in self-inhibiting systems. Include a null model with self-inhibition removed (aX and aY very large) to show that ectosymbiosis does not invade when only costs are present.

Minor points

1. Model exposition and notation

State explicitly that rX and rZ are not direct functions of the inhibitor; inhibition acts via u/(a + b*u) multiplying growth. Introduce kappa = (1 - n*k_YZ)*(1 - n*s_Z) early and provide a brief derivation or indicate whether it is an approximation and under what conditions it holds. If the consortium inhibition term reuses bX by assumption, say so plainly; otherwise define and analyze bZ.

2. Dilution and consistency

Explain the role of Omega and test sensitivity. Clarify the asymmetry in how Omega scales the symbiont's access to U but not the host's access to its resource, and test sensitivity to Omega across biologically plausible ranges.

3. Figures and diagnostics

For time series, reduce overplotting or add nullclines/vector fields to clarify approach to equilibria. For region plots, add labels or contours indicating invasion and stability margins. Provide at least one EP example with numerical eigenvalues if EP remains a featured outcome.

4. Parameter ranges and empirical diagnostics

Where possible, bound key parameters by orders of magnitude from microbial systems (uptake rates, decay, inhibition parameters) or justify the explored ranges. Where possible, bound key parameters using the empirical systems already referenced in the manuscript (for example, acetate inhibition in E. coli, alcohol toxicity in yeast, Chlorochromatium consortia). Even approximate values from these examples would help constrain the relevant parameter space.

5. Reproducibility

Extend SI code with scripts for parameter sweeps and, if added, AD PIPs. Document seeds and initial-condition ranges used to generate figures.

6. Editorial clarity

Use "consortium" (singular) and "consortia" (plural) consistently throughout. Replace ambiguous phrases such as "reducing the inhibition of accumulating products" with parameter-linked wording. State explicitly whether "consortium" and "mutant host" are treated interchangeably in equations and figures.

With these changes, the manuscript would move from a possibility result under stylized assumptions to a testable theory with clear conditions under which attachment can invade and persist.

Reviewer #2: In this manuscript, the authors develop a mathematical model to investigate whether a mutant host capable of physically binding symbionts can invade a resident population of free-living hosts and symbionts. Under metabolite-induced self-inhibition, an ectosymbiotic consortium is less exposed to the toxic metabolite due to its reduced effective surface area, but incurs lower resource uptake and higher metabolic costs. The authors analyze this tradeoff and derive conditions under which the mutant host can or cannot invade.

Overall, the work presents an interesting hypothesis for the ecological origins of ectosymbiotic consortia, potentially representing a first step toward endosymbiotic integration. The tradeoff between reduced inhibition and slowed growth is conceptually appealing, and the invasion conditions are potentially insightful. However, several issues in presentation and model formulation should be addressed:

Model presentation and notation

The Results section frequently refers to variables, parameters, and equation-based statements without first presenting the model in a compact mathematical form. The full equations appear only in Methods and Appendices. In addition, the text names for the same entities are not always used consistently. These issues make the Results section hard to follow.

I suggest introducing the final form of the model equations at the beginning of the Results and, at that point, giving each entity a standard full descriptive name together with its symbol. In the subsequent text, the authors can use text name + symbol, with the text name optionally shortened as long as it remains a clear subset of the original full name. Establishing this naming convention early and using it consistently would greatly improve readability. It would also be helpful to label the variables directly in Fig. 1 so that readers can easily connect the notation used in the text with the components shown in the diagram.

Inconsistency between consumption and growth terms (Eq. 4)

In Eq. (4), the net growth rate r_Y includes a ln⁡2 factor arising from the assumption that each cell consumes resource at constant rate throughout its cell cycle. However, the population-level consumption term, -k_Y u/Ω y, implies that per-capita consumption rate increases continuously as the population grows from 1 to 1.99 cells before division. Thus, the flux of resource consumption is measured in two incompatible ways in the growth equation and in the substrate equation. To maintain mass-balance consistency, the authors should either:

∙ include the same ln⁡2 factor in the population-level consumption term (i.e., replace -k_Y u/Ω y with -ln⁡2 k_Y u/Ω y), or

∙ remove the ln⁡2 factor from r_Y (and possibly also from r_X and r_Z for consistency).

Treatment of ectosymbiont dynamics in the mutant-only regime

The Discussion (lines 393–409) argues that the dynamics of attached ectosymbionts can be ignored because a large external symbiont pool effectively buffers them. This is reasonable when free-living symbionts are present, but it does not apply to the mutant-only equilibrium where no free-living symbionts remain.

In the mutant-only case, the attached symbionts are the only symbiont population, so neglecting their dynamics can lead to behavior that appears biologically implausible. For example, the model allows free-living symbionts to be driven extinct by metabolite V produced by ectosymbionts, yet the same level of V does not appear to limit the ectosymbionts on the host. The authors may therefore need to either exclude the mutant-only equilibrium from the biological interpretation or refine the treatment of ectosymbiont dynamics near y→0.

Line 648–650: unclear inequality

The inequality nk_YZ<1 is stated without derivation. Based on the surrounding text, it is not clear how this condition arises, and it may be that a relationship involving u and ψ, such as nk_YZ u<k_z>

Ambiguity in the definitions of u and Ω

Table 1 defines u(t) as the “Concentration of host metabolic product U in the habitat” and Ω as the “Volume of the habitat or medium (reflects dilution)”. However, this makes the meaning of u/Ω unclear. In different parts of the text, u and u/Ω appear to play different roles (e.g., concentration near the host vs. far from the host, or intracellular vs. extracellular levels). The authors should clarify the physical interpretation of u, Ω, and u/Ω, and make the intended spatial or compartmental structure explicit.</k_z>

**********

**Have the authors made all data and (if applicable) computational code underlying the findings in their manuscript fully available?**

The PLOS Data policy requires authors to make all data and code underlying the findings described in their manuscript fully available without restriction, with rare exception (please refer to the Data Availability Statement in the manuscript PDF file). The data and code should be provided as part of the manuscript or its supporting information, or deposited to a public repository. For example, in addition to summary statistics, the data points behind means, medians and variance measures should be available. If there are restrictions on publicly sharing data or code —e.g. participant privacy or use of data from a third party—those must be specified.

Reviewer #1: Yes

Reviewer #2: None

**********

PLOS authors have the option to publish the peer review history of their article (what does this mean?). If published, this will include your full peer review and any attached files.

If you choose “no”, your identity will remain anonymous but your review may still be made public.

**Do you want your identity to be public for this peer review?**  For information about this choice, including consent withdrawal, please see our Privacy Policy.

Reviewer #1: No

Reviewer #2: No

[NOTE: If reviewer comments were submitted as an attachment file, they will be attached to this email and accessible via the submission site. Please log into your account, locate the manuscript record, and check for the action link "View Attachments". If this link does not appear, there are no attachment files.]

**Figure resubmission:**

**Reproducibility:**

?>

# Author comment: Author response to Decision Letter 1

**DOI:** 10.1371/journal.pcbi.1014699.r002

17 Feb 2026

Attachment Submitted filename: Response_to_Reviewers.pdf

# Peer review: Decision Letter 1

**DOI:** 10.1371/journal.pcbi.1014699.r003

**Authors:** Virginia E. Pitzer, Christopher Rao

5 Apr 2026

PCOMPBIOL-D-25-01620R1

Host-initiated microbial association leads to stable ectosymbiosis in an ecological model

PLOS Computational Biology

Dear Dr. Krishnan,

Thank you for submitting your revised manuscript to PLOS Computational Biology. After careful consideration, we feel that it has merit but does not fully meet PLOS Computational Biology's publication criteria as it currently stands. Therefore, we invite you to submit a revised version of the manuscript that addresses the points raised during the review process.

Please submit your revised manuscript by Jun 05 2026 11:59PM. If you will need more time than this to complete your revisions, please reply to this message or contact the journal office at ploscompbiol@plos.org. When you're ready to submit your revision, log on to https://www.editorialmanager.com/pcompbiol/ and select the 'Submissions Needing Revision' folder to locate your manuscript file.

Please include the following items when submitting your revised manuscript:

* A letter that responds to each point raised by the editor and reviewer(s). You should upload this letter as a separate file labeled 'Response to Reviewers'. This file does not need to include responses to formatting updates and technical items listed in the 'Journal Requirements' section below.

* A marked-up copy of your manuscript that highlights changes made to the original version. You should upload this as a separate file labeled 'Revised Manuscript with Track Changes'.

* An unmarked version of your revised paper without tracked changes. You should upload this as a separate file labeled 'Manuscript'.

If you would like to make changes to your financial disclosure, competing interests statement, or data availability statement, please make these updates within the submission form at the time of resubmission. Guidelines for resubmitting your figure files are available below the reviewer comments at the end of this letter.

We look forward to receiving your revised manuscript.

Kind regards,

Christopher Rao

Academic Editor

PLOS Computational Biology

Virginia Pitzer

Editor-in-Chief

PLOS Computational Biology

**********

Note: If the reviewer comments include a recommendation to cite specific previously published works, please review and evaluate these publications to determine whether they are relevant and should be cited. There is no requirement to cite these works unless the editor has indicated otherwise.

**Reviewers' comments:**

Reviewer's Responses to Questions

Reviewer #1: Manuscript: "Host-initiated microbial association leads to stable ectosymbiosis in an ecological model" (PCOMPBIOL-D-25-01620R1)

Authors: Nandakishor Krishnan, Istvan Zachar, Adam Kun, Chaitanya S. Gokhale, Jozsef Garay

Recommendation: Major Revision

The revised manuscript is clearly improved, and the authors have made a serious effort to respond to my previous review. Several additions are useful and move the paper in a better direction. In particular, they now provide a more explicit biophysical rationale for the proposed surface-shielding mechanism, examine a nonlinear shielding function, include a consumption-only control, expand the parameter-region analysis, add a null model without self-inhibition, and discuss an extension with unsynchronized host-symbiont replication. These are meaningful revisions.

However, I do not think the manuscript is yet ready for acceptance. Some of my concerns have been addressed satisfactorily, but others remain only partly resolved, and a few still affect the central interpretation of the study. Most importantly, the manuscript continues to move beyond what is actually shown by the ecological invasion-and-stability analysis. For that reason, my recommendation remains Major Revision.

1. Core mechanism and parameterization

This point is improved, but not fully resolved. The added diffusion-based rationale for reduced inhibition at the host-symbiont contact surface is helpful, and the sensitivity test using a nonlinear shielding function is also a useful addition. The consumption-only control likewise helps clarify the structure of the model, because it makes clearer that invasion depends on the inhibition-related terms, whereas syntrophy affects whether the long-term outcome is facultative or consortium-only.

That said, one of my more specific requests was not actually carried out. I had asked for a sensitivity analysis in which b_Z is allowed to differ from b_X. Instead, the authors argue that b_Z should equal b_X by construction. That is a legitimate modeling decision, but it is not the same as demonstrating robustness to relaxing the assumption. In a phenomenological model, it would still have been informative to test whether the qualitative result persists when that symmetry is broken. I also think the revised explanation of why p_Z and s are separated, while clearer than before, makes it more evident that invasion depends on treating the cost and benefit sides of surface occupation as independently tunable. That dependence should be stated more directly in the manuscript.

2. Ectosymbiont dynamics and inheritance

This concern remains only partly addressed, and in my view it is still important. The new extension with unsynchronized host-symbiont replication is valuable because it shows that the consortium-only outcome is much less robust once perfect synchronization is relaxed. The authors now acknowledge that consortium dominance in the present framework effectively depends on very low escape or release of ectosymbionts. That is an important clarification.

However, the broader issue I raised was not limited to synchronization alone. The manuscript still does not address variance in n, stochastic partitioning during host division, or explicit attachment-detachment dynamics. Those are not marginal details. They go directly to the question of whether the association can be stably transmitted and maintained. The revised manuscript is more open about this limitation, but the limitation itself remains substantial.

3. Mixed equilibrium E_P, coexistence, and bistability

This point is much better than before. The authors now state more clearly that coexistence of resident and mutant host types can be excluded algebraically, and they indicate that E_P is not bistable with E_2 or E_M. They also provide numerical Jacobians and eigenvalue calculations in the supplementary computational material. That addresses much of the substance of my concern.

My remaining reservation is mainly one of presentation. The revised SI now makes the analytical stability results clearer for E_2 and E_M, but the local stability analysis of E_P is still deferred mainly to the code supplement rather than presented directly in the SI narrative.

4. Robustness and parameter sensitivity

This part is substantially improved. The added parameter-region plots are useful, and the clarification of the role of Omega is responsive to my earlier concern. In particular, it is now clearer that Omega does not affect invasion itself, but does affect whether the long-term ecological outcome is E_P or E_M. That is a helpful distinction.

At the technical level, I regard this issue as largely addressed. What remains weaker is the biological interpretation of those regions. The manuscript still does not say enough about whether the viable parts of parameter space are broad in any biologically constrained sense, as opposed to broad only within the internal logic of the abstract model.

5. Evolutionary framing

This remains one of the main unresolved issues. In their response, the authors state that they do not aim to make evolutionary arguments and that they have toned down the corresponding language. However, the revised manuscript still repeatedly uses strongly evolutionary wording. It continues to state, in various places, that obligate ectosymbiosis can evolve, that consortial cooperation is selected for, that an evolutionary path exists, and that the model represents a plausible first step toward endosymbiotic integration. The manuscript also still frames the work in eco-evolutionary terms, even though the later clarification makes clear that evolutionary endpoints are not actually modeled.

This mismatch remains serious. An ecological mutant-invasion analysis can certainly motivate evolutionary hypotheses, but it does not, by itself, justify claims about evolutionary trajectories or long-term transitions such as endosymbiotic integration. If the authors do not wish to add an explicit evolutionary treatment, then the manuscript needs a much more consistent ecological framing throughout, especially in the abstract, author summary, and discussion.

6. Finite symbiont pool and public-good logic

This point is partly addressed. The authors now explain more clearly that the free-living symbiont pool is treated as effectively abundant, and they no longer lean as heavily on a tragedy-of-the-commons interpretation. They also state that a finite-supply extension gives qualitatively similar results. That clarification is useful.

Even so, I still think the manuscript could explain the benefit-allocation logic more clearly. Even under an abundant-pool assumption, the key conceptual issue is how attachment redistributes local benefit between resident and mutant host types, and how that redistribution contributes to invasion. That remains central to the interpretation of the model and deserves a sharper explanation in the paper itself.

7. Scope, definitions, and strength of claims

This is improved, but not fully corrected. The authors have made some constructive changes here. In particular, the distinction between different meanings of "obligate" is clearer than before, and the null model without self-inhibition is an important addition because it shows that consortium formation does not invade in the absence of the proposed ecological driver.

Still, the manuscript continues to move too quickly from ecological exclusion outcomes to broader claims about long-term symbiotic integration, vertical inheritance, higher-level individuality, and eukaryogenesis. Those ideas may well motivate the study, but they are not demonstrated by the present model. I therefore still think the claims need to be narrowed. The paper would be stronger if it stayed closer to what it actually shows: the ecological conditions under which a costly consortium-forming mutant can invade and persist when hosts suffer self-inhibition and attachment reduces that inhibition locally.

8. Minor issues

Several of the minor issues are now handled satisfactorily. The notation is clearer, the role of kappa is more explicit, the treatment of Omega is easier to follow, and the figures appear improved. Reproducibility is also better supported through the supplementary computational material.

My main remaining minor concern is that the local stability analysis of E_P is still largely deferred to the code supplement rather than presented directly in the SI narrative. The revised appendix now provides explicit analytical stability statements for E_2 and E_M, which improves the presentation substantially, but for E_P it still states that the stability conditions are difficult to obtain analytically and refers the reader to S2 for the numerical local stability analysis. A second remaining issue is empirical parameterization. The authors respond that the main conclusions are analytical and therefore do not depend on the exact numerical values used in the illustrations. That is reasonable as far as internal model logic is concerned, but it does not fully answer the interpretive point. A brief discussion of whether the explored parameter regions are at least broadly plausible for microbial systems would still strengthen the manuscript.

Overall assessment

In sum, the revised manuscript is better and the revision is not superficial. The authors have responded thoughtfully to several of my comments, and the paper is now clearer in a number of respects. However, I do not think the most important remaining issues are minor. The two central problems are, first, the continued mismatch between the ecological analysis actually performed and the stronger evolutionary and endosymbiotic claims that are still made in the text, and second, the fact that the inheritance and transmission problem remains only partially explored despite being central to the biological interpretation of stable ectosymbiosis. In addition, some robustness questions were answered by defending the original assumptions rather than by testing the requested relaxations.

For these reasons, I recommend Major Revision.

**********

**Have the authors made all data and (if applicable) computational code underlying the findings in their manuscript fully available?**

The PLOS Data policy requires authors to make all data and code underlying the findings described in their manuscript fully available without restriction, with rare exception (please refer to the Data Availability Statement in the manuscript PDF file). The data and code should be provided as part of the manuscript or its supporting information, or deposited to a public repository. For example, in addition to summary statistics, the data points behind means, medians and variance measures should be available. If there are restrictions on publicly sharing data or code —e.g. participant privacy or use of data from a third party—those must be specified.

Reviewer #1: Yes

**********

PLOS authors have the option to publish the peer review history of their article (what does this mean?). If published, this will include your full peer review and any attached files.

If you choose “no”, your identity will remain anonymous but your review may still be made public.

**Do you want your identity to be public for this peer review?**  For information about this choice, including consent withdrawal, please see our Privacy Policy.

Reviewer #1: No

[NOTE: If reviewer comments were submitted as an attachment file, they will be attached to this email and accessible via the submission site. Please log into your account, locate the manuscript record, and check for the action link "View Attachments". If this link does not appear, there are no attachment files.]

**Figure resubmission:**

After uploading your figures to PLOS’s NAAS tool - https://ngplosjournals.pagemajik.ai/artanalysis, NAAS will process the files provided and display the results in the "Uploaded Files" section of the page as the processing is complete. If the uploaded figures meet our requirements (or NAAS is able to fix the files to meet our requirements), the figure will be marked as "fixed" above. If NAAS is unable to fix the files, a red "failed" label will appear above. When NAAS has confirmed that the figure files meet our requirements, please download the file via the download option, and include these NAAS processed figure files when submitting your revised manuscript.

**Reproducibility:**

To enhance the reproducibility of your results, we recommend that authors of applicable studies deposit laboratory protocols in protocols.io, where a protocol can be assigned its own identifier (DOI) such that it can be cited independently in the future. Additionally, PLOS ONE offers an option to publish peer-reviewed clinical study protocols. Read more information on sharing protocols at https://plos.org/protocols?utm_medium=editorial-email&utm_source=authorletters&utm_campaign=protocols

# Author comment: Author response to Decision Letter 2

**DOI:** 10.1371/journal.pcbi.1014699.r004

18 May 2026

Attachment Submitted filename: Response_to_Reviewers_Second.pdf

# Peer review: Decision Letter 2

**DOI:** 10.1371/journal.pcbi.1014699.r005

**Authors:** Virginia E. Pitzer, Christopher Rao

22 Jul 2026

PCOMPBIOL-D-25-01620R2

Host-initiated microbial association leads to stable ectosymbiosis in an ecological model

PLOS Computational Biology

Dear Dr. Krishnan,

Thank you for submitting your manuscript to PLOS Computational Biology. Based on the reviewer's comments, the manuscript is significant improved. However, the reviewer still had a few comments that need to be addressed. Assuming these are addressed, then the manuscript does not need to go out for review again.

Please submit your revised manuscript by Sep 21 2026 11:59PM. If you will need more time than this to complete your revisions, please reply to this message or contact the journal office at ploscompbiol@plos.org. When you're ready to submit your revision, log on to https://www.editorialmanager.com/pcompbiol/ and select the 'Submissions Needing Revision' folder to locate your manuscript file.

Please include the following items when submitting your revised manuscript:

* A letter that responds to each point raised by the editor and reviewer(s). You should upload this letter as a separate file labeled 'Response to Reviewers'. This file does not need to include responses to formatting updates and technical items listed in the 'Journal Requirements' section below.

* A marked-up copy of your manuscript that highlights changes made to the original version. You should upload this as a separate file labeled 'Revised Manuscript with Track Changes'.

* An unmarked version of your revised paper without tracked changes. You should upload this as a separate file labeled 'Manuscript'.

If you would like to make changes to your financial disclosure, competing interests statement, or data availability statement, please make these updates within the submission form at the time of resubmission. Guidelines for resubmitting your figure files are available below the reviewer comments at the end of this letter.

As the corresponding author, your ORCID iD is verified in the submission system and will appear in the published article. PLOS supports the use of ORCID, and we encourage all coauthors to register for an ORCID iD and use it as well. Please encourage your coauthors to verify their ORCID iD within the submission system before final acceptance, as unverified ORCID iDs will not appear in the published article. Only the individual author can complete the verification step; PLOS staff cannot verify ORCID iDs on behalf of authors.

We look forward to receiving your revised manuscript.

Kind regards,

Christopher Rao

Academic Editor

PLOS Computational Biology

Virginia Pitzer

Editor-in-Chief

PLOS Computational Biology

**Additional Editor Comments (if provided):**

**Journal Requirements:**

If the reviewer comments include a recommendation to cite specific previously published works, please review and evaluate these publications to determine whether they are relevant and should be cited. There is no requirement to cite these works unless the editor has indicated otherwise.

1) We noticed that you used the phrase 'data not shown' in the manuscript. We do not allow these references, as the PLOS data access policy requires that all data be either published with the manuscript or made available in a publicly accessible database. Please amend the supplementary material to include the referenced data or remove the references.

**Reviewers' comments:**

Reviewer's Responses to Questions

**Comments to the Authors:**

**Please note here if the review is uploaded as an attachment.**

Reviewer #1: Manuscript: "Host-initiated microbial association leads to stable ectosymbiosis in an ecological model" (PCOMPBIOL-D-25-01620R2)

Authors: Nandakishor Krishnan, Istvan Zachar, Adam Kun, Chaitanya S. Gokhale, Jozsef Garay

Summary

The manuscript has improved substantially across the revision rounds. The previously requested additions are now present and internally consistent: the diffusion-boundary-layer rationale for inhibition reduction, the separation of cost and benefit into independent parameters, the b_Z != b_X sensitivity analysis (Appendix A4.4, Fig A4), the imperfect-vertical-inheritance analysis (Appendix A4.3, Fig A3), a simplified constant-symbiont-supply control (fixed symbiont density y=1, S2 Code, section 2.4), and the numerical Jacobians and eigenvalues for all fixed points including E_P (S2 Code). The remaining technical concerns are therefore addressed. What is left is not mathematical but interpretive: the manuscript still overstates what the model demonstrates about evolution and eukaryogenesis, and it under-reports one dependence that is central to its own headline claim.

Recommendation: Minor Revision

I do not request further modeling; the requested changes are presentation and claim-calibration issues.

1. Claim calibration. The manuscript still uses "prove/proves" (Author Summary l. 56, Results l. 188, Discussion l. 349) and forward-looking statements such as "evolutionary path ... toward obligate endosymbiosis" and "could have eventually led to the endosymbiotic origin of mitochondria" (l. 61). A feasibility model shows possibility under assumptions, not proof, and does not analyze a mutation sequence, trait substitution, or evolutionary stability. Replace "prove" with "show", "demonstrate within the model", or "identify conditions under which", and phrase the trajectory and eukaryogenesis statements as hypotheses. The ecological-selection framing ("ecologically selected for", invasion as a first step) is acceptable; only the unhedged claims need softening, consistently in the Abstract, Author Summary, Results, and Discussion.

2. Dependence on ectosymbiont retention should be visible in the main text. Two separate sensitivity analyses bear on this. In the imperfect-inheritance model (Appendix A4.3), consortial persistence requires theta > theta_critical (approx 0.8 for the illustrative parameters), and both free-living types go extinct only at perfect inheritance (theta = 1); in the ectosymbiont-release model (Appendix A4.2), the consortium-only outcome E_M is likewise recovered only in the limit of zero escape (tau = 0), with small positive tau approaching it while retaining a low free-living symbiont density. This dependence is central, not a side caveat, and sits uneasily with the Abstract's claim that population-level obligate ectosymbiosis emerges "under a wide range of conditions". Add a sentence in the Results near E_M stating it, and calibrate the Abstract to distinguish invasion and facultative ectosymbiosis, which hold over a range, from the obligate outcome, which requires these ideal-retention limits.

3. Scope of "obligate". Keep E_M described as population-level ecological exclusion throughout. The statement that free-living extinction "effectively ensures" that symbionts cannot replicate without the host (l. 459-461) holds only within the ecological closure of the model; it is not genotype-level obligacy or a mechanistic loss of autonomy, and should be stated as such.

4. E_P stability presentation. The E_P Jacobian and eigenvalues are computed in S2 Code, but the appendix narrative still only states the existence and necessary conditions and defers local stability to the code. Add one worked example in Appendix A3: the parameter set, the E_P fixed point, and its eigenvalues, so the result is verifiable without running the notebook.

5. Parameter-region interpretation. Appendix A4 already notes that the Table 1 values are arbitrary and that empirical data on the costs and benefits of prokaryotic consortia are limited. Bring one sentence to this effect into the main text, clarifying that the explored regions are internally consistent feasibility regions rather than empirically calibrated ranges. The issue is visibility in the manuscript, not absence from the supplement.

Minor points

- State the independence of s and p_Z (benefit vs cost of surface coverage) briefly in the Results, not only in Methods, as it is central to interpretation.

- Ensure the s / p_Z renaming (formerly s_Z) and the definition kappa = (1 - n p_Z)(1 - n k_YZ) are consistent across the main text, appendix, and code, and check "consortium"/"consortia" usage.

Overall, the model is sound and the central result is publishable. The revision needed is a final calibration of the evolutionary and eukaryogenesis claims and greater visibility of the inheritance dependence; once these are done, the manuscript should be acceptable.

**********

**Have the authors made all data and (if applicable) computational code underlying the findings in their manuscript fully available?**

The PLOS Data policy requires authors to make all data and code underlying the findings described in their manuscript fully available without restriction, with rare exception (please refer to the Data Availability Statement in the manuscript PDF file). The data and code should be provided as part of the manuscript or its supporting information, or deposited to a public repository. For example, in addition to summary statistics, the data points behind means, medians and variance measures should be available. If there are restrictions on publicly sharing data or code —e.g. participant privacy or use of data from a third party—those must be specified.

Reviewer #1: Yes

**********

PLOS authors have the option to publish the peer review history of their article (what does this mean?). If published, this will include your full peer review and any attached files.

If you choose “no”, your identity will remain anonymous but your review may still be made public.

**Do you want your identity to be public for this peer review?**  For information about this choice, including consent withdrawal, please see our Privacy Policy.

Reviewer #1: No

[NOTE: If reviewer comments were submitted as an attachment file, they will be attached to this email and accessible via the submission site. Please log into your account, locate the manuscript record, and check for the action link "View Attachments". If this link does not appear, there are no attachment files.]

**Figure resubmission:**

**Reproducibility:**

To enhance the reproducibility of your results, we recommend that authors of applicable studies deposit laboratory protocols in protocols.io, where a protocol can be assigned its own identifier (DOI) such that it can be cited independently in the future. Additionally, PLOS ONE offers an option to publish peer-reviewed clinical study protocols. Read more information on sharing protocols at https://plos.org/protocols?utm_medium=editorial-email&utm_source=authorletters&utm_campaign=protocols

# Author comment: Author response to Decision Letter 3

**DOI:** 10.1371/journal.pcbi.1014699.r006

30 Jul 2026

Attachment Submitted filename: Response_to_reviewers_Third.pdf

# Editor report: Decision Letter 3

**DOI:** 10.1371/journal.pcbi.1014699.r007

**Authors:** Virginia E. Pitzer, Christopher Rao

10 Aug 2026

Dear Mr. Krishnan,

We are pleased to inform you that your manuscript 'Host-initiated microbial association leads to stable ectosymbiosis in an ecological model' has been provisionally accepted for publication in PLOS Computational Biology.

Before your manuscript can be formally accepted you will need to complete some formatting changes, which you will receive in a follow up email. A member of our team will be in touch with a set of requests.

Please note that your manuscript will not be scheduled for publication until you have made the required changes, so a swift response is appreciated.

IMPORTANT: The editorial review process is now complete. PLOS will only permit corrections to spelling, formatting or significant scientific errors from this point onwards. Requests for major changes, or any which affect the scientific understanding of your work, will cause delays to the publication date of your manuscript.

Should you, your institution's press office or the journal office choose to press release your paper, you will automatically be opted out of early publication. We ask that you notify us now if you or your institution is planning to press release the article. All press must be co-ordinated with PLOS.

Thank you again for supporting Open Access publishing; we are looking forward to publishing your work in PLOS Computational Biology.

Best regards,

Christopher Rao

Academic Editor

PLOS Computational Biology

Virginia Pitzer

Editor-in-Chief

PLOS Computational Biology

***********************************************************

# Editor report: Acceptance letter

**DOI:** 10.1371/journal.pcbi.1014699.r008

**Authors:** Virginia E. Pitzer, Christopher Rao

PCOMPBIOL-D-25-01620R3

Host-initiated microbial association leads to stable ectosymbiosis in an ecological model

Dear Dr Krishnan,

I am pleased to inform you that your manuscript has been formally accepted for publication in PLOS Computational Biology. Your manuscript is now with our production department and you will be notified of the publication date in due course.

The corresponding author will soon be receiving a typeset proof for review, to ensure errors have not been introduced during production. Please review the PDF proof of your manuscript carefully, as this is the last chance to correct any errors. Please note that major changes, or those which affect the scientific understanding of the work, will likely cause delays to the publication date of your manuscript.

Soon after your final files are uploaded, unless you have opted out, the early version of your manuscript will be published online. The date of the early version will be your article's publication date. The final article will be published to the same URL, and all versions of the paper will be accessible to readers.

For Research, Software, and Methods articles, you will receive an invoice from PLOS for your publication fee after your manuscript has reached the completed accept phase. If you receive an email requesting payment before acceptance or for any other service, this may be a phishing scheme. Learn how to identify phishing emails and protect your accounts at https://explore.plos.org/phishing.

Thank you again for supporting PLOS Computational Biology and open-access publishing. We are looking forward to publishing your work!

With kind regards,

Janani Seenivasan

PLOS Computational Biology | Carlyle House, Carlyle Road, Cambridge CB4 3DN | United Kingdom ploscompbiol@plos.org | Phone +44 (0) 1223-442824 | ploscompbiol.org | @PLOSCompBiol
